# TDP-43 prevents retrotransposon activation in the *Drosophila* motor system through regulation of Dicer-2 activity

**DOI:** 10.1186/s12915-020-00816-1

**Published:** 2020-07-03

**Authors:** Giulia Romano, Raffaella Klima, Fabian Feiguin

**Affiliations:** International Centre for Genetic Engineering and Biotechnology, Padriciano 99, 34149 Trieste, Italy

**Keywords:** Neurodegeneration, Retrotransposon, TDP-43, Motoneurons, Dicer-2, siRNA, *Drosophila*

## Abstract

**Background:**

Mutations in the small RNA-binding protein TDP-43 lead to the formation of insoluble cytoplasmic aggregates that have been associated with the onset and progression of amyotrophic lateral sclerosis (ALS), a neurodegenerative disorder affecting homeostasis of the motor system which is also characterized by aberrant expression of retrotransposable elements (RTEs). Although the TDP-43 function was shown to be required in the neurons and glia to maintain the organization of neuromuscular synapses and prevent denervation of the skeletal muscles, the molecular mechanisms involved in physiological dysregulation remain elusive. Here, we address this issue using a null mutation of the TDP-43 *Drosophila* homolog, TBPH.

**Results:**

Using genome-wide gene expression profiles, we detected a strong upregulation of RTE expression in TBPH-null *Drosophila* heads, while the genetic rescue of the TDP-43 function reverted these modifications. Furthermore, we found that TBPH modulates the small interfering RNA (siRNA) silencing machinery responsible for RTE repression. Molecularly, we observed that TBPH regulates the expression levels of Dicer-2 by direct protein-mRNA interactions in vivo. Accordingly, the genetic or pharmacological recovery of Dicer-2 activity was sufficient to repress retrotransposon activation and promote motoneuron axonal wrapping and synaptic growth in TBPH-null *Drosophila*.

**Conclusions:**

We identified an upregulation of RTE expression in TBPH-null *Drosophila* heads and demonstrate that defects in the siRNA pathway lead to RTE upregulation and motoneuron degeneration. Our results describe a novel physiological role of endogenous TDP-43 in the prevention of RTE-induced neurological alterations through the modulation of Dicer-2 activity and the siRNA pathway.

## Background

Amyotrophic lateral sclerosis (ALS) is a devastating disease that affects the homeostasis of the motor system, defined by motoneurons and the associated glia, leading to muscle denervation, progressive paralysis, and neurodegeneration. Regarding the pathological mechanisms of the disease, studies performed in brain tissues obtained from deceased patients revealed the presence of insoluble aggregates of the small ribonuclear protein TDP-43 distributed along the cytoplasm and outside the cell nucleus [1–3]. These modifications strongly correlate with the symptoms of the disease and were observed in the great majority of the sporadic and familial cases of ALS [4]. However, it is still a matter of debate how histological alterations in TDP-43 lead to neurodegeneration. In this direction, experiments performed in transgenic animals indicated that TDP-43 is an aggregation-prone protein that induces neurodegeneration when overexpressed in neuronal tissues [5–10]. Moreover, analogous research lines showed that TDP-43 variants carrying mutations linked to familial cases of ALS were more predisposed to form aggregates and, in addition, more neurotoxic [11–16]. On the other hand, the formation of insoluble aggregates may also disrupt the physiological function of the endogenous protein and lead to neurodegeneration through mechanisms related with the absence of TDP-43 function in the nucleus. In relationship with these observations, we demonstrated that the suppression of the TDP-43 homolog protein in *Drosophila* (TBPH) faithfully reproduced in flies the main characteristics of the human disease alike paralysis, motoneuron degeneration, and reduced life span [17, 18]. Moreover, we described that TBPH function is permanently required in the neurons and glia to maintain the molecular organization of the neuromuscular synapses as well as prevent the denervation of the skeletal muscles [19, 20], supporting the idea that deficiencies in TBPH function may conduct to ALS by interfering with the physiological regulation of critical metabolic pathways inside the motor system. In order to identify these molecules, we performed a transcriptome comparison of gene expression profiles between wild-type and TBPH-null mutant adult head tissues. Intriguingly, we observed that the absence of TBPH provoked the upregulation of notorious families of conserved retrotransposons that included the endogenous retrovirus (ERV) *gypsy.* In addition, we found that the genetic recovery of TBPH activity prevented the activation of these elements, revealing that the endogenous function of TBPH is required for retrotransposon repression. In the present study, we tested the hypotheses described above and explored the mechanisms regulated by TBPH in retrotransposons silencing. Moreover, we investigated the neurological consequences of ERV activation in TBPH-null flies and examined if similar regulatory pathways are conserved in human neuroblastoma cells. Finally, we tested novel pharmacological compounds and therapeutic strategies to compensate for the defects of TBPH loss of function in the repression of retrotransposon activation. We hope that our results will provide novel arguments to understand the disease process and facilitate the way to novel curative interventions in ALS.

## Results

### The lack of TBPH induces the expression of retrotransposons in *Drosophila*

**Fig. 1:**
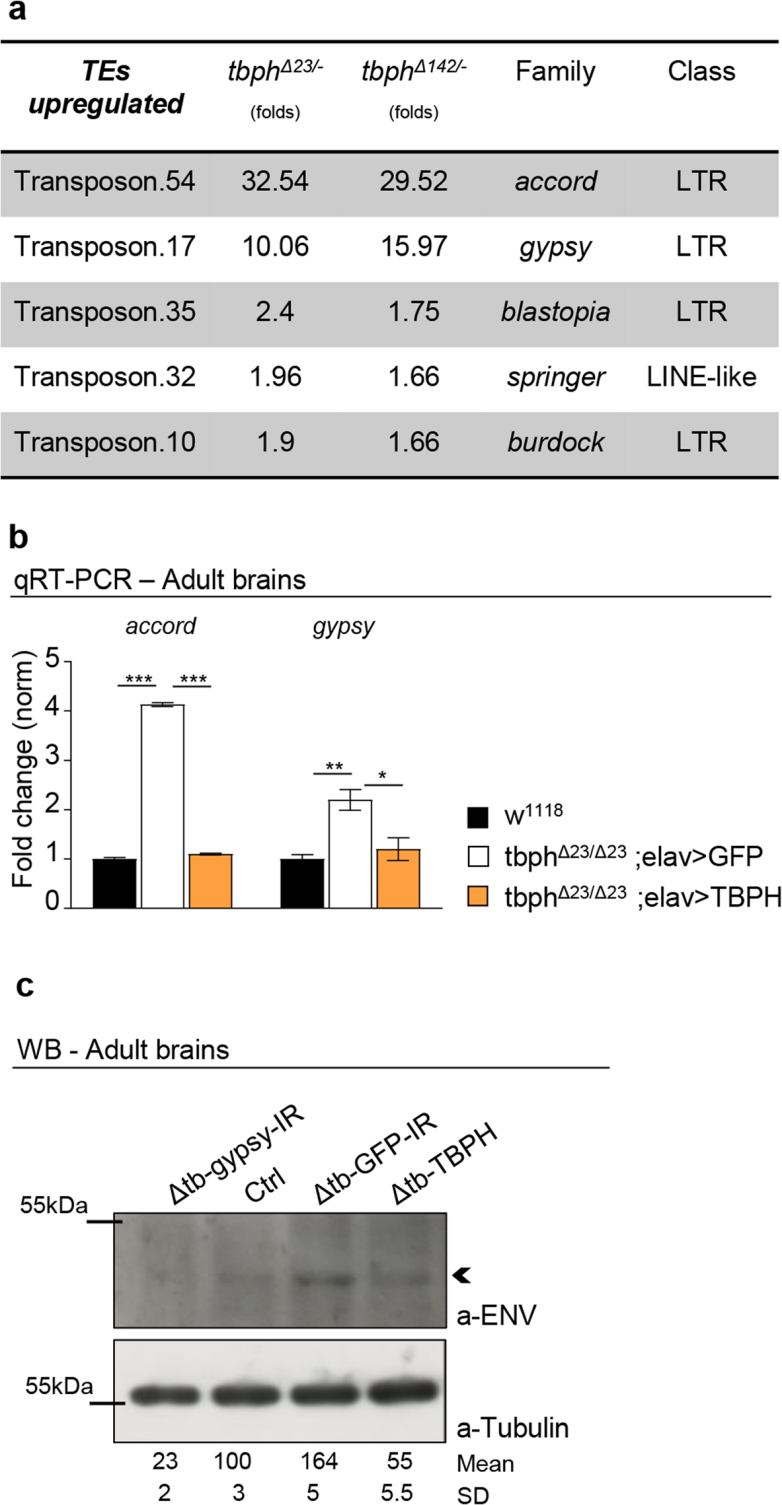
RTEs are upregulated in TBPH mutants. **a** Microarray results showing upregulated TEs in TBPH-null mutants: the fold changes are reported for both tbph mutant alleles (Δ23 and Δ142) and referred to *w*^1118^; TE family and class were also indicated. *n* = 3 (biological replicates). **b** Real-time quantitative PCR reveals *accord* and *gypsy* transcript levels normalized on *Rpl11* (housekeeping) in *w*^1118^ - tbph^Δ23^,elav-GAL4/tbph^Δ23^; UAS-GFP/+ and tbph^Δ23^,*elav*-GAL4/tbph^Δ23^,UAS-TBPH. *n* = 3 (biological replicates, with 3 technical replicates for each), error bars SEM. **c** Western blot analysis of *Drosophila env* levels in Δtb-gypsy-IR (tbph^Δ23^,*elav*-GAL4/tbph^Δ23^; UAS-gypsy-IR/+), ctrl (tbph^Δ23^,*elav*-GAL4/+), Δtb-GFP-IR (tbph^Δ23^,*elav*-GAL4/tbph^Δ23^; UAS-GFP-IR/+), and Δtb-TBPH (tbph^Δ23^,*elav*-GAL4/tbph^Δ23^,UAS-TBPH). Adult brains, 1 day old, were probed with anti-ENV and alpha-tubulin antibodies. The same membrane was probed with the two antibodies and the bands of interest were cropped. Quantification of normalized amounts and SD was reported below each lane. *n* = 2 (biological replicates). *p < 0.05, **p < 0.01, ***p < 0.001 calculated by one-way ANOVA, error bars SEM. Individual data values are provided in Additional file 6: Individual Data Values.pdf

We have previously indicated that the molecular function of TBPH is permanently required in *Drosophila* motoneurons to prevent muscle denervation, locomotive defects, and early neurodegeneration [19]. In order to identify the molecules involved in the neurodegenerative process initiated by the absence of TBPH, we utilized *Drosophila melanogaster* to analyze the differences in the patterns of gene expression between wild-type and TBPH-minus flies. For these experiments, the mRNAs expressed in adult heads of TBPH-null alleles (tbph^Δ23^ and tbph^Δ142^) and wild-type controls were isolated to hybridize GeneChip Drosophila Genome 2.0 Arrays (Additional file 1 Table 1 “w11118 vrs tbphD23”, Additional file 2 Table 2 “W1118 vrs tbphD142”). Intriguingly, the statistical analysis of these experiments revealed that 12 out of the 79 transposons, present in the microarray, appeared dysregulated in TBPH-minus alleles compared to wild type (Fig. 1a and Additional file 3 Fig. S1a-b). In this fashion, we observed that the great majority of the altered transposons belonged to the long terminal repeat (LTR) family of retrotransposons. In particular, we found that *accord* and *gypsy* were the LTRs that presented the highest levels of upregulation in TBPH-mutant heads (Fig. 1a). The modifications described in the microarray were independently confirmed by quantitative RT-PCR (qRT-PCR) using different combinations of primers against the RNA sequences transcribed from these elements (Fig. 1b). In addition, we detected that the glycoprotein *env*, codified by *gypsy* [21], appeared upregulated in TBPH-minus heads compared to controls confirming through different methodologies that the activity of the retrotransposons was increased in TBPH-mutant tissues (Fig. 1c). More importantly, we found that the genetic expression of the TBPH protein was able to repress the activation of *accord* and *gypsy* in TBPH-mutant backgrounds demonstrating that the role of TBPH in the repression of these elements was rather specific (Fig. 1b, c).

### The activation of retrotransposons causes motoneuron degeneration in TBPH-null flies

**Fig. 2:**
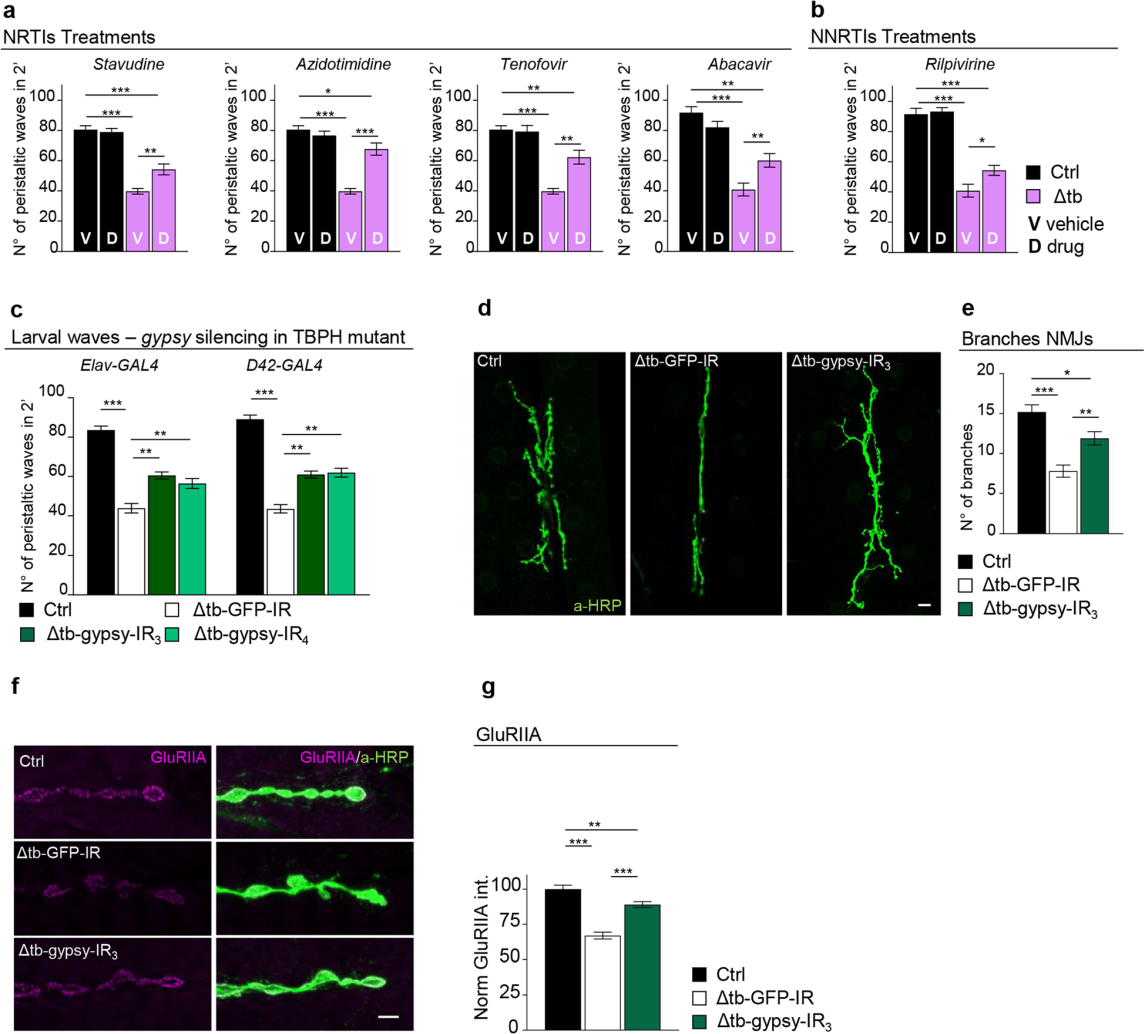
Pharmacological and genetic suppression of RTEs revert TBPH mutant phenotypes. **a** Number of peristaltic waves of Ctrl (*w*^1118^) and Δtb (tbph^Δ23^/tbph^Δ23^) fed with NRTI drugs (D) compared to vehicle only (V). *n* = 20. **b** Number of peristaltic waves of Ctrl (*w*^1118^) and Δtb (tbph^Δ23^/tbph^Δ23^) fed with NNRTI drugs (D) compared to vehicle only (V). *n* = 20. **c** Number of peristaltic waves of Ctrl (*w*^1118^), Δtb-GFP-IR (tbph^Δ23^,*elav*-GAL4/tbph^Δ23^; UAS-GFP-IR/+), Δtb-gypsy-IR_3_ (tbph^Δ23^,*elav*-GAL4/tbph^Δ23^; UAS-gypsy-IR_3_/+), and Δtb-gypsy-IR_4_ (tbph^Δ23^,*elav*-GAL4/tbph^Δ23^; UAS-gypsy-IR_4_/+). *n* = 20. **d** Confocal images of third instar NMJ terminals in muscle 6/7 second segment stained with anti-HRP (in green) in Ctrl, Δtb-GFP-IR, and Δtb-gypsy-IR_3_. **e** Quantification of branch number. *n* = 15. **f** Confocal images of third instar NMJ terminals in muscle 6/7 second segment stained with anti-HRP (in green) and anti-GluRIIA (in magenta) in Ctrl, Δtb-GFP-IR, and Δtb-gypsy-IR_3_. **g** Quantification of GluRIIA intensity. *n* > 200 boutons. *p < 0.05, **p < 0.01, ***p < 0.001 calculated by one-way ANOVA, error bars SEM. Scale bar 10 μm (**d**) and 5 μm (**f**)

The observations related above indicate that the endogenous function of the TBPH might be required to prevent the activation of retrotransposons in vivo. Furthermore, the data also suggests that the mobilization of these elements may contribute to the phenotypes provoked by the absence of TBPH function in *Drosophila* neurons. To test these possibilities, we treated TBPH-null flies with different combinations of nucleoside and non-nucleoside revert transcriptase inhibitors (NRTI and NNRTI) [22]. As a result, we observed that the oral administration of the NRTIs: stavudine, azidotimidine, tenofovir, and abacavir, together with the NNRTI rilpivirine was able to revert the locomotive defects described in TBPH-minus third instar larvae, indicating that the activation of RTEs contributed to these phenotypes (Fig. 2a, b and Additional file 4 Fig. S2a). In this direction, we decided to analyze more in detail the neurological consequences of *gypsy* upregulation in TBPH-minus *Drosophila*. This is because *gypsy* is a very active retrotransposon in *Drosophila*, responsible for the majority of the spontaneous mutations described in flies [23], and more significantly, the upregulation of this retrotransposon was already associated with the neurodegenerative phenotypes induced by the overexpression of human TDP-43 in flies [24]. Additionally, *gypsy* presents strong similarities with the viral protein HERV-K, a human endogenous retrovirus recently detected in patients with ALS [25–27]. Therefore, to test the role of *gypsy* in TBPH-null phenotypes, we decided to silence the expression of this element in tbph^Δ23^ homozygous flies. For these experiments, we utilized transgenic flies carrying RNAi constructs against the endogenous mRNA sequence of *gypsy* (*gypsy*-IR) cloned in UAS expression vectors [28]. Consequently, we found that the neuronal expression of two independent RNAi lines against *gypsy* (*gypsy*-IR_3_ and IR_4_) utilizing the pan-neuronal driver *elav-GAL4* or the more restricted motoneuronal promoter *D42-GAL4* were able to significantly revert the locomotive phenotypes observed in TBPH-minus third instar larvae (tbph^Δ23^/tbph^Δ23^; *elav-GAL4* or *D42-GAL4*/*gypsy*-IR_3_-IR_4_) compared to control flies expressing an RNAi against GFP (tbph^Δ23^/tbph^Δ23^; *elav-GAL4* or *D42-GAL4*/GFP-IR) (Fig. 2c). Surprisingly, we noticed that the genetic rescue of the locomotive behaviors induced by the suppression of *gypsy* in TBPH-null backgrounds was correlated with the continuous growth of the presynaptic terminals (Fig. 2d, e) and the formation of the glutamate receptor clusters present at the postsynaptic membranes (Fig. 2f, g), demonstrating that the abnormal activation of *gypsy* negatively contributes to the assemble of the neuromuscular synapses and impairs muscle innervation. Subsequently, we noticed that the suppression of *gypsy* in glial cells, using *repo-GAL4* (tbph^Δ23^/tbph^Δ23^; *repo-GAL4*/*gypsy*-RI_3_), was not able to rescue the locomotive phenotypes occasioned by the absence of TBPH (Additional file 4 Fig. S2b), suggesting that the repression of *gypsy* in the glia may not be sufficient to revert the neurological defects induced by the lack of TBPH function in *Drosophila* brains.

### TBPH controls the silencing of retrotransposons by regulating the expression levels of Dicer-2

**Fig. 3:**
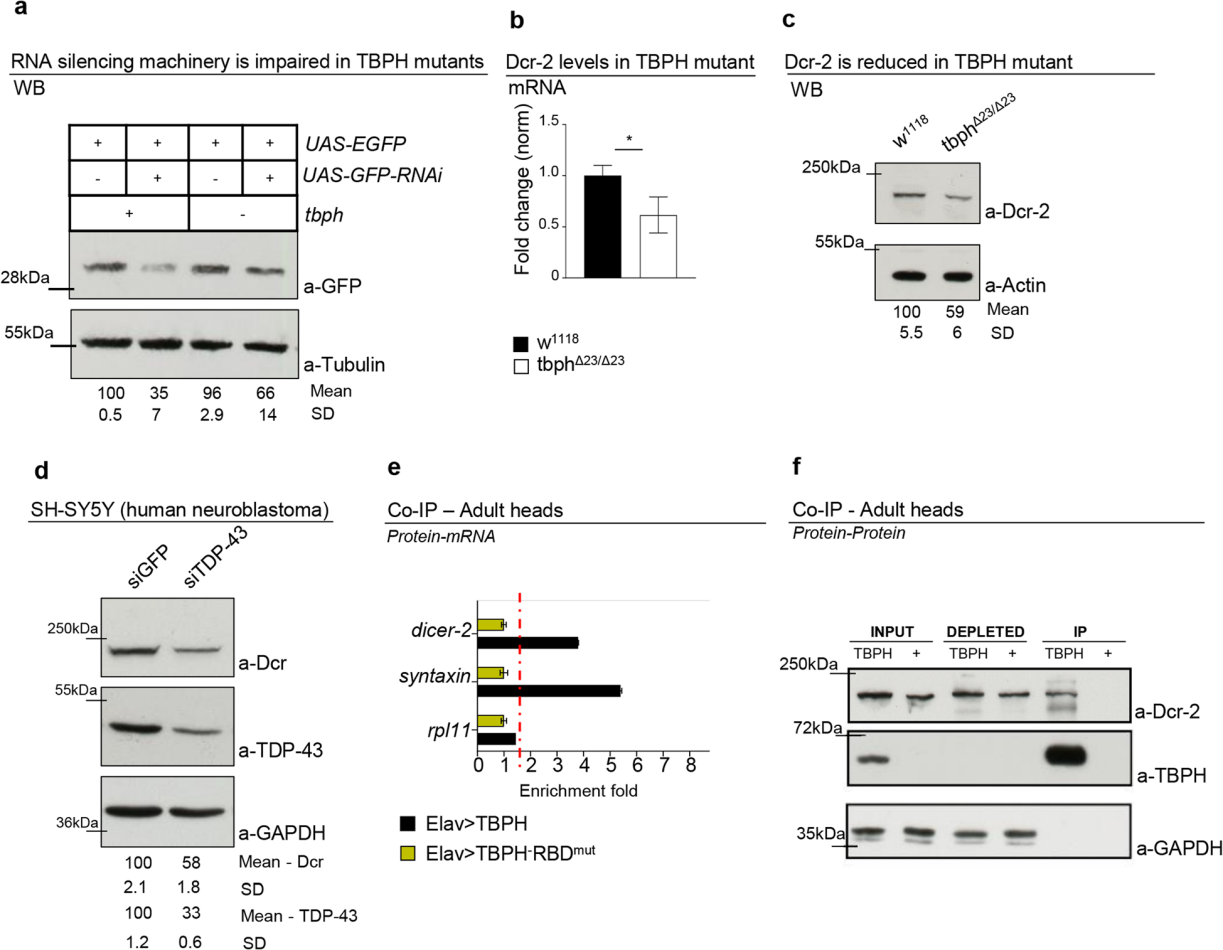
TBPH physically interacts and influences *Dcr-2* levels. **a** Western blot analysis of lane 1 (+/+;D42-GAL4,UAS-EGFP/+), lane 2 (+/+;D42-GAL4,UAS-EGFP/UAS-GFP-IR), lane 3 (tbph^Δ23^/tbph^Δ23^; D42-GAL4,UAS-EGFP/+), and lane 4 (tbph^Δ23^/tbph^Δ23^; D42-GAL4,UAS-EGFP/UAS-GFP-IR). Adult brains, 1 day old, were probed with anti-GFP and alpha-tubulin antibodies. The same membrane was probed with the two antibodies, and the bands of interest were cropped. Quantification of normalized amounts was reported below each lane. *n* = 2 (independent biological replicates). **b** Real-time PCR of *Dcr-2* transcript levels normalized on *Rpl11* (housekeeping) in adult heads of *w*^1118^ and tbph^Δ23^/tbph^Δ23^. *n* = 3 (independent biological replicates, with 2 technical replicates for each), *p < 0.05, calculated by two-tailed *t* test, error bars SEM. **c** Western blot analysis of *w*^1118^ and tbph^Δ23^/tbph^Δ23^ adult brains probed with anti-Dicer and anti-actin antibodies. The same membrane was probed with the two antibodies, and the bands of interest were cropped. Quantification of normalized amounts and SD was reported below each lane. *n* = 4 (independent biological replicates), *p < 0.05, calculated by two-tailed *t* test. **d** Western blot analysis on human neuroblastoma (SH-SY5Y) cell line probed for anti-Dicer, anti-GAPDH, and anti-TDP-43 in siGFP (GFP ctrl) and siTDP-43 (TDP-43 silenced). The same membrane was probed with the three antibodies, and the bands of interest were cropped. Quantification of normalized protein amount and SD was reported below each lane. *n* = 3 (independent biological replicates), **p < 0.01, calculated by two-tailed *t* test. **e** qRT-PCR analysis of mRNAs immunoprecipitated by FLAG-tagged TBPH (Elav>TBPH) and its mutant variants TBPH-RBD^mut^ (Elav>TBPH-RBD^mut^). The *dicer-2* enrichment folds were referred to as *rpl-11* (negative control), and syntaxin has been used as a positive control. *n* = 2 (biological replicates). **f** Western blot analysis of TBPH (GMR-GAL4/UAS-TBPH) and + (GMR-GAL4/+). Input, depleted, and immunoprecipitated (IP) materials were analyzed, probing the membrane with anti-TBPH, anti-Dicer, and anti-GAPDH. *n* = 4 (biological replicates). Individual data values are provided in Additional file 6: Individual Data Values.pdf

The retrotransposons have the capacity to transcribe themselves through RNA intermediates [23, 29]. In physiological conditions, the expression of these elements is maintained under repression through the synthesis of small interference RNAs (siRNAs). These molecules present a typical size of 21–23 nucleotides and mediate the post-transcriptional repression of the retrotransposons through the formation of RNA-induced silencing complexes (RISC) [30]. The conservation of the siRNAs sequences among different species is very high, as well as their expression patterns that include different somatic tissues and the brain [31, 32]. Regarding to that and considering that the expression levels of retrotransposons were upregulated in TBPH-null flies, we decided to test whether the siRNA silencing machinery was affected in TBPH-mutant flies compared to wild-type controls. To test this hypothesis, we took advantage of a previously described methodology based on the co-expression of a GFP-IR construct together with a GFP reporter in transgenic flies [24, 33]. Along these lines, differences in the expression levels of the GFP reporter (quantified by western blot) would reflect the efficiency of the RNA silencing machinery in the different genetic backgrounds. Accordingly, we utilized *D42-GAL4* to express the constructs described above and observed that wild-type motoneurons were able to silence the GFP reporter in a more efficient manner compared to TBPH-minus flies (Fig. 3a), indicating that the activity of TBPH is required for the normal functioning of the siRNA machinery in vivo. In order to identify the molecular mechanisms behind these alterations, we investigated whether the expression levels of the different components of the siRNA machinery were affected in TBPH-mutant heads. For these experiments, we utilized qRT-PCR technics to test the brain levels of the principal constituents of the RISC complex like Dicer-2, loquacious, and Argonaute 2. A different group of genes, previously associated with the silencing of LTR such as piwi, pasha, and homeless, was similarly analyzed [34]. Interestingly, we found that the RNase Dicer-2 (*Dcr-2*) was the only transcript that presented mRNA levels significantly downregulated in TBPH-null alleles (Fig. 3b and Additional file 5 Fig. S3). Furthermore, we observed that the protein levels of *Dcr-2* were equally downregulated in mutant fly heads compared to controls (Fig. 3c). More relevant, we found that the suppression of TDP-43 in human neuroblastoma SH-SY5Y cells produced a similar reduction in the expression levels of the human Dicer protein (Fig. 3d) suggesting that these modifications are, apparently, conserved among the species [35]. In order to understand how TBPH may regulate the levels of *Dcr-2* expression, we decided to explore if these molecules were able to physically interact in vivo. In support of this idea, we identified the presence of TBPH putative binding sites in the coding sequence of *Dcr-2* mRNA, indicating that these molecules could interact in vivo. For these experiments, we expressed a FLAG-tagged isoform of TBPH in *Drosophila* neurons and performed pull-down assays from fly head tissues [18, 19]. In this manner, we found that the mRNA of *Dcr-2* appeared highly enriched in TBPH immunoprecipitated samples compared to similar experiments performed utilizing a modified variant of this protein that is unable to bind the RNA (TBPH-RBD^mut^), [36, 37], confirming that these molecules physically interact in vivo (Fig. 3e). In addition, we observed that TBPH was also capable to interact with *Dcr-2* at the protein level demonstrating that these molecules form part of the same protein complex in *Drosophila* neurons (Fig. 3f). Altogether, our results reveal that TBPH may modulate the efficiency of the siRNA machinery through the regulation of *Dcr-2* expression levels in *Drosophila* neurons. Furthermore, TBPH could also have a role in *Dcr-2* activity throughout the formation of RISC complexes by direct protein-protein interactions.

### The re-establishment of *Dcr-2* levels in TBPH-minus motoneurons or glial cells promotes synaptic growth and muscle innervation

**Fig. 4:**
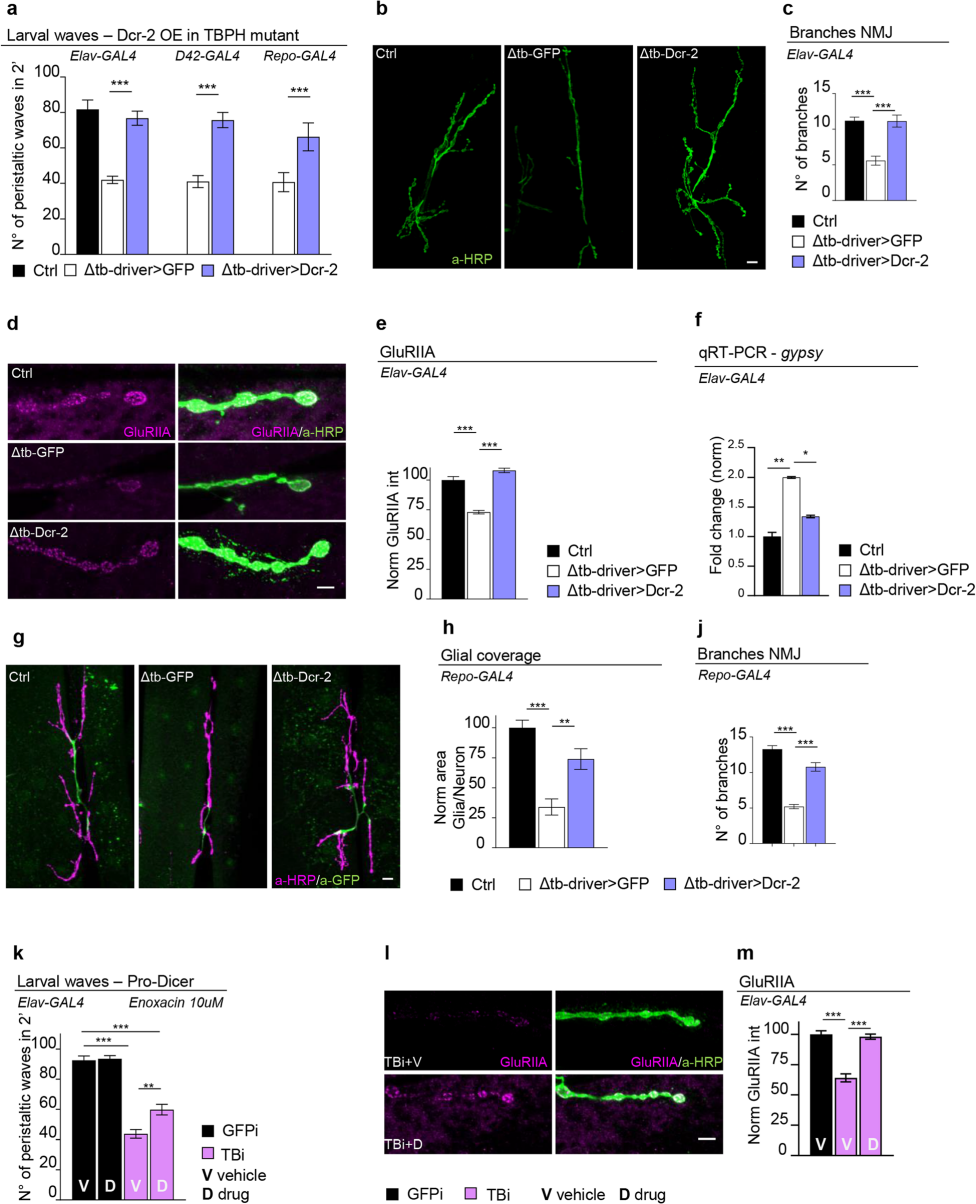
Genetic rescue of *Dcr-2* expression recovers TBPH mutant pathological phenotypes. **a** Number of peristaltic waves of Ctrl (*w*^1118^), Δtb-driver>GFP (tbph^Δ23^/tbph^Δ23^;driver-GAL4/UAS-GFP), and Δtb-driver>Dcr-2 (UAS-Dcr-2/+;tbph^Δ23^/tbph^Δ23^;driver-GAL4/+). *elav*-GAL4, D42-GAL4, and *repo*-GAL4 were used as reported on the figure. *n* = 20. **b** Confocal images of third instar NMJ terminals in muscle 6/7 second segment stained with anti-HRP (in green) in Ctrl, Δtb-driver>GFP, and Δtb-driver>Dcr-2, using elav-GAL4. **c** Quantification of branch number. *n* = 15. **d** Confocal images of third instar NMJ terminals in muscle 6/7 second segment stained with anti-HRP (in green) and anti-GluRIIA (in magenta) in Ctrl, Δtb-driver>GFP, and Δtb-driver>Dcr-2, using *elav*-GAL4. **e** Quantification of GluRIIA intensity. *n* > 200 boutons. **f** Real-time PCR of *gypsy* transcript levels normalized on *Rpl11* (housekeeping) in Ctrl, Δtb-driver>GFP, and Δtb-driver>Dcr-2, using *elav*-GAL4. *n* = 2, error bars SEM. **g** Confocal images of third instar NMJ terminals in muscle 6/7 second segment stained with anti-HRP (in magenta) for presynaptic membrane and anti-GFP (in green) for peripheral glia in Ctrl (+/+;*repo*-GAL4,UAS-GFP/+), Δtb-GFP (tbph^Δ23^/tbph^Δ23^;*repo*-GAL4,UAS-GFP/+), and Δtb-Dcr-2 (UAS-Dcr-2/+;tbph^Δ23^/tbph^Δ23^;*repo*-GAL4,UAS-GFP/+). **h** Quantification of glial area. *n* = 10 larvae. **j** Quantification of branch number. *n* = 10. **k** Number of peristaltic waves of GFPi (Dcr-2/+; tbph^Δ23^,*elav*-GAL4/+;UAS-GFP-IR/+) and TBi (Dcr-2/+; tbph^Δ23^,*elav*-GAL4/+;UAS-TBPH-IR/+) fed with 10 μM enoxacin (D) compared to vehicle only (V). *n* = 20. **l** Confocal images of third instar NMJ terminals in muscle 6/7 second segment stained with anti-HRP (in green) and anti-GluRIIA (in magenta) in GFPi and TBi. **m** Quantification of GluRIIA intensity. *n* > 200 boutons. *p < 0.05, **p < 0.01, ***p < 0.001 calculated by one-way ANOVA, error bars SEM. Scale bar 10 μm (**b**, **g**) and 5 μm (**d**, **l**)

The data described above indicates that the endogenous TBPH protein is physiologically required to prevent the activation of retrotransposons by mechanisms related with the regulation of *Dcr-2* levels in *Drosophila* brains. In order to test this hypothesis, we decided to recover the expression levels of *Dcr-2* in TBPH-mutant backgrounds. For these experiments, transgenic flies containing the *Dcr-2* gene cloned under UAS regulatory sequences (UAS-*Dcr-2*) were crossed against insects carrying the pan-neuronal driver *elav-GAL4* or the more constrained motoneurons promoter *D42-GAL4*. Strikingly, we observed that the expression of UAS-*Dcr-2* in neurons or motoneurons was sufficient to revert the serious locomotive problems shown in TBPH-null larvae (tbph^Δ23^/tbph^Δ23^; *elav-GAL4* or *D42-GAL4*/UAS-*Dcr-2*) compared to identical flies expressing the unrelated protein GFP (tbph^Δ23^/tbph^Δ23^; *elav-GAL4* or *D42-GAL4*/UAS-GFP) (Fig. 4a). Moreover, we found that the recovery of the fly locomotion was complemented by the outgrowth of the motoneuron synaptic terminals and the innervation of the underlying muscles (Fig. 4b, c). These modifications were followed by the reorganization of the glutamate receptor clusters at the postsynaptic membranes (Fig. 4d, e). Furthermore, we detected that the expression of UAS-*Dcr-2* was able to prevent the augmented expression of *gypsy* in TBPH-mutant brains (Fig. 4f), demonstrating that the alterations in *Dcr-2* activity were responsible for the pathological activation of *gypsy* and the neurological phenotypes associated with the absence of TBPH. In the same direction, we found that the expression of *Dcr-2* in glial cells with *repo*-GAL4 (tbph^Δ23^/tbph^Δ23^; *repo-GAL4*/UAS-*Dcr-2*) was likewise sufficient to rescue the locomotive deficiencies observed in TBPH-minus flies (Fig. 4a). At the cellular level, we detected that the expression of *Dcr-2* stimulated the glial wrapping of the motoneurons axons through the enlargement of the cytoplasmic area covered by the peripherical glia at the NMJs (Fig. 4g, h). In addition, the expression of *Dcr-2* in glial tissue was able to induce the non-autonomous growth of the neuromuscular synapses (Fig. 4j) revealing the unexpected role of *Dcr-2* in these tissues to sustain synaptic development. Taken together, the results described above predict that therapeutic interventions aimed to potentiate *Dcr-2* activity, along with the siRNA machinery, would be beneficial to prevent the neurological problems triggered by alterations in TBPH function. In support of this idea, we observed that TBPH-minus larvae treated with enoxacin [38] were able to significantly recover their locomotive capacities and motoneuron innervation patterns (Fig. 4k–m) confirming, through a different approach, the role of *Dcr-2* in TBPH-minus phenotypes and proposing that similar therapeutic strategies could be beneficial in patients with ALS.

## Discussion

The aberrant activation of retrotransposons (RTEs) was observed in brain tissues obtained from individuals affected by distinctive neurodegenerative diseases and described in patients carrying familial or sporadic mutations in TDP-43, insinuating that this RNA-binding protein might be involved in the mechanisms responsible for RTE repression. In agreement with this hypothesis, Krug et al. have previously demonstrated that the overexpression of human TDP-43 induce neurodegeneration through the activation of RTEs by mechanisms related with the disruption of the siRNA machinery in *Drosophila* [24]. However, these experiments did not elucidate if these phenotypes correspond to the endogenous function of TDP-43 or rather represent the dominant interference of this protein with its numerous mRNA targets and/or protein partners. In that respect, it remains a matter of debate whether the physiological function TDP-43 is required to maintain the repressed status of RTEs and the molecular mechanisms involved. Regarding that, we performed a genome-wide analysis using DNA microchips hybridized with head tissues obtained from null alleles of TBPH, the TDP-43 homolog protein *Drosophila*. As a result, we found a number of RTEs that appeared consistently dysregulated in tbph^Δ23^- and tbph^Δ142^-mutant flies, and these positive hits were further confirmed by quantitative RT-PCR. Furthermore, we observed that one of the most upregulated RTEs was *gypsy* and confirmed that the glycoprotein *env*, codified for this retrotransposon, was also upregulated in TBPH-mutant heads. In addition, we showed that the genetic rescue of the missing copies of TBPH was able to repress the activation of RTEs and revert the upregulation of the *env* protein observed in TBPH-null backgrounds, demonstrating that these molecular alterations were specific.

### The activation of RTEs provokes motoneuron degeneration in TBPH-null flies

Regarding the biological implications of the results described above, several lines of investigation have suggested that the mobilization of the RTEs provokes neuronal decline and degeneration [24, 39, 40]. On the contrary, parallel studies have reported that the activation of the retrotransposons drives genomic heterogeneity and promotes neurogenesis [41]. Taking into consideration these possible scenarios, we found that the suppression of retrotransposons transcription, through the administration of revert transcriptase inhibitors and/or nucleoside revert transcriptase inhibitors, was able to ameliorate the locomotive problems described in TBPH-minus flies. More specifically, we observed that the suppression of *gypsy* in neurons or motoneurons was sufficient to revert locomotive defects and promote motoneuron synaptic growth and muscle innervation in *Drosophila* TBPH-null mutants. These results imply that TBPH is physiologically required to prevent the deleterious activation of these transposable elements in neurons and, more restrictedly, in motoneurons. The fact that alterations in the loss and gain of TDP-43/TBPH function similarly promote the activation of RTEs correlates very well with the pathological scenario observed in ALS brains. Accordingly, the vast majority of patients present defects in the nuclear localization of this protein with an increased accumulation of TDP-43 forming insoluble aggregates in the cytoplasm making the pathological interpretation of these alterations difficult, if not, how to distinguish between defects occasioned from the loss of the nuclear function of TDP-43 and effects due to the toxic gain of function of this protein in the cytoplasm. These possibilities are a matter of strong debate in the ALS field, and in that respect, our studies demonstrate that alterations in the nuclear role of TBPH are sufficient to provoke the pathological activation of RTEs and suggest that a tight regulation of TDP-43 activity or `cellular distribution is required to prevent retrotransposon activation in the affected brains.

### TBPH prevents RTE-mediated neurodegeneration via the regulation of *Dcr-2* levels

At the molecular level, we found that the RNA silencing activity of the siRNA machinery was reduced in TBPH-null neurons. Additionally, we detected that *Dcr-2*, one of the principal components of the siRNA pathway, was downregulated in TBPH-mutant heads suggesting that defects in the activity of this endoribonuclease might be responsible for the alterations in the repression of RTEs as described above. In agreement with these hypotheses, we observed that the genetic rescue of *Dcr-2* expression levels was able to prevent the activation of the *gypsy* and support motoneuron axon terminal growth in TBPH loss of function *Drosophila*. Regarding that, we found that the expression of *Dcr-2* in glial tissues was similarly able to revert the motility defects specified in TBPH-minus flies, the glial wrapping of motoneuron axons, and the non-autonomous formations of new synaptic branches (Fig. 4a, g–j) suggesting that the activation of the siRNA machinery positively contributed to the proper functioning of the glia, most probably, via the repression of different retrotransposons and/or by playing a role in the organization of the heterochromatin [42, 43]. Regarding the molecular mechanisms, besides TBPH-mediated *Dcr-2* regulation, we found that TBPH forms molecular complexes with *Dcr-2* through physical interactions with the mRNA and the protein itself. Despite the meaning of these interactions is not well known, TBPH might be required for the intracellular localization and/or transport of the RISC complex inside or outside the nucleus or contribute to the binding and recognition of the RNA targets. Nevertheless, the formation of similar protein complexes, together with Dicer and Drosha, was described for human TDP-43 suggesting that these mechanisms might be conserved and present in ALS [35]. In agreement with this idea, we found that the suppression of TDP-43 induces the downregulation of Dicer in human neuroblastoma cell lines signifying that the TDP-43 function is required to prevent defects in Dicer protein expression or stability.

### Pharmacological treatments aimed to enhance the siRNA silencing activity were able to rescue motoneuron defects in TBPH-null flies

Finally, our experiments demonstrated that TBPH physically interacts with *Dcr-2* in protein complexes signifying that TBPH may act as a regulatory component of the RNA-induced silencing complexes (RISC) in *Drosophila* neurons. In consonance with these findings, we uncovered that pharmacological treatments utilizing enoxacin, a compound capable to activate the siRNA pathway, were able to restore the locomotive behaviors and the formation of neuromuscular synapsis in TBPH-deficient flies. These therapeutic interventions, either alone or in combination with NRTIs and NNRTIs, may help to control the activation of RTEs and, hopefully, the progress of the disease in familial or sporadic ALS.

## Conclusions

In this study, we performed a genome-wide analysis of gene expression profiles and identify a number of RTEs highly upregulated in TBPH-null *Drosophila* heads. Moreover, we demonstrated that the activation of RTEs provokes motoneuron degeneration in TBPH-minus flies due to the defects in the *Dcr-2* activity and the disruption of the siRNA silencing machinery. Additionally, we uncovered that pharmacological treatments aimed to inhibit transcription of RTEs or activate the siRNA pathway were able to restore the locomotive behaviors and the formation of neuromuscular synapsis in TBPH-mutant flies, suggesting that similar therapeutic interventions might be beneficial in patients with familial or sporadic ALS.

## Methods

### Fly strains and maintenance

All flies were maintained at 25 °C, with a 12:12-h light:dark cycle, on standard cornmeal food (agar 6.25 g/L, yeast 62.5 g/L, sugar 41.6 g/L, flour 29 g/L, propionic acid 4.1 mL/L).

The genotypes of the flies used in this work are indicated as follows:

*w*^1118^ - w;tbph^Δ23^/CyO^GFP^ - w;tbph^Δ142^/CyO^GFP^ - w;*elav*-GAL4/CyO^GFP^ - w;;D42-GAL4 - *repo*-GAL4/TM3,Sb - GMR-GAL4/CyO - UAS-Dcr-2 - w;;UAS-EGFP - w;UAS-TBPH - w;;UAS-TBPH-RBD^mut^ - UAS-gypsy-IR insertion 3 and 4 (gifted by Professor Peng Jin) - UAS-TBPH-RNAi/TM6b (#ID38377, VDRC) - UAS-mCD8:GFP - UAS-GFP-IR (#9330 Bloomington). All fly lines used in this work were backcrossed with *w*^1118^ strain in order to minimize the differences in the genetic background.

### Larval movement

Peristaltic waves of third instar larvae were performed as already described in [17]. Briefly, larvae, after genotype selection, were rinsed in water and transferred to a 0.7% agarose dish (94 mm diameter), and peristaltic waves were counted for a period of 2 min. A minimum of 20 animals were analyzed for each genotype to reach a statistically representative population.

### Drug treatment of larvae

Parental fly crosses were settled on standard cornmeal added of the below-listed drugs with the reported final concentration: stavudine 10 μM, azidotimidine 10 μM, tenofovir 10 μM, abacavir (#SML0089 Sigma) 10 μM, rilpivirine (#10410 Sigma) 10 μM, enoxacin (#AB143281 Abcam) 10 μM, and lamivudine (#L1295 Sigma) 10 μM. For each drug, a vehicle-only control group was arranged. Parental flies have been maintained 24 h in the tubes to allow the embryo laying. Synchronized embryos were grown to obtain third instar larvae to be tested for mobility or to be analyzed for NMJ morphology.

### RNA extraction and microarray analysis

RNA, both from adult dissected brains and adult heads, was extracted with the RNeasy Microarray tissue kit (QIAGEN #73304). Gene expression analysis was performed on three independent biological replicates by GenoSplice company on the Affymetrix platform using Gene Chip Drosophila Genome 2.0 Array. RNAs extracted from *Drosophila* adult heads, 1 day aged and sex-matched, of both wild-type and TBPH-null alleles (*tbph*^Δ23^ and *tbph*^Δ142^) were subjected to quality control tests before chip hybridization. The min-fold change for both upregulated and downregulated genes was settled to 1.5.

### Immunohistochemistry, confocal, and acquisition

Third instar larvae bodies were dissected and stained as previously described [19]. Larvae were dissected in HL-3, fixed in 4% paraformaldehyde 20 min (5 min in methanol for anti-GluRIIA), and subsequently blocked in 5% normal goat serum (Vector laboratories #S-1000) in PBS and 0.1% Tween 20. Primary antibody incubations were performed overnight at 4 °C, while secondary antibodies were incubated at room temperature for 2 h. Dilutions of the antibodies used are reported as follows: anti-HRP (Jackson ImmunoResearch 1:150), anti-HRP-Cy3 (Jackson ImmunoResearch 1:150), anti-GluRIIA 8B4D2c (DSHB 1:15), anti-GFP (Life Technology 1:200), Alexa-Fluor® 488 (mouse or rabbit 1:500), and Alexa- Fluor® 555 (mouse or rabbit 1:500).

Stained larvae were mounted with SlowFade Gold (#S36936 Thermo Fisher Scientific), and images of muscles 6 and 7 of the second abdominal segments were gained on a Zeiss LSM880 laser scanning microscope (× 63 oil lens). All acquisitions performed in these experiments were simultaneously processed using the same microscope settings and subsequently analyzed by ImageJ (Wayne Rasband, NIH) and Prism (GraphPad, USA) software.

### Quantification of confocal images

Animals analyzed for these experiments were processed simultaneously and images acquired with the same settings. For the GluRIIA quantification, samples were double labeled with anti-GluRIIA and anti-HRP: the mean intensity of both was quantified and a ratio calculated [19, 44]. To label the peripheral glia that covers the presynaptic terminals at the NMJ level, a membrane-tethered green fluorescent protein (UAS—mCD8-GFP) was expressed using Repo-GAL4 driver. The quantification of glial area was calculated analyzing the ratio between the area occupied by glial tissue and the area of the presynaptic terminal [20]. All the data were normalized on the control.

### Cell culture and RNA interference

SH-SY5Y neuroblastoma cell line was cultured in DMEM-GlutaMAX (#31966-021, Thermo Fisher Scientific) supplemented with 10% fetal bovine serum and 1× antibiotic-antimycotic solution (#A5955; Sigma). For RNA interference, 2–4 × 10^5^ cells were seeded in a 60-mm plate in 2 mL of medium containing 10% fetal serum. Two rounds of silencing, for a total of 48 h silencing, were carried out. HiPerfect Transfection Reagent (#301705, Qiagen) and Opti-MEM I reduced serum medium (#51985-026, Thermo Fisher Scientific) were used with a 200-nM final concentration of siRNA, (TDP43: 5′-gcaaagccaagaugagccu-3′ and EGFP control: 5′-gcaccaucuucuucaagga-3′; Sigma). Silenced cells were collected by trypsinization, lysed in RIPA buffer, and immunoblotted.

### Immunoblot

*Drosophila* adult heads or brains were homogenized in lysis buffer 1× (10 mM Tris, 150 mM NaCl, 5 mM EDTA, 5 mM EGTA, 10% glycerol, 50 mM NaF, 5 mM DTT, 4 M urea, pH 7.4, plus protease inhibitors and protein content quantified with Quant-iT Protein Assay Kit (#Q33211 Thermo Fisher Scientific). SH-SY5Y cells were homogenized in RIPA buffer (NaCl 150 mM, NP-40 1%, sodium deoxycholate 0.5%, SDS 0.1%, EDTA 2 mM, Tris 50 mM, pH 8.0) added to protease inhibitors, and protein lysates were quantified by Pierce™ BCA Protein Assay Kit (#23225, Thermo Fisher Scientific). Lysates were separated on SDS-PAGE and wet-transferred to nitrocellulose membranes (#NBA083C, Whatman). The primary antibody used were anti-Env (1:100 gifted by Prof. Christophe Terzian), anti-Dcr-2 (1:300 #ab4732, Abcam), anti-h-Dicer (1:3000, #PA5-78446, Thermo Fisher Scientific), anti-hTDP (1:4000, #12892-1-AP, ProteinTech), anti-GFP (1:3000, #A11122, Thermo Fisher Scientific), anti-TBPH (1:4000, homemade, [17], anti-GAPDH (1:1000 #sc-25778, Santa Cruz), anti-tubulin (1:2000, #CP06, Calbiochem), anti-actin (1:3000, Sigma).

### Immunoprecipitation for protein-protein interaction

Approximately one hundred *Drosophila* heads for each genotype (GMR-GAL4/UAS-TBPH and GMR-GAL4/+) were collected by flash freezing and homogenized in immunoprecipitation buffer (20 mM Tris pH 7.5, 110 mM NaCl, 0.5% Triton X-100, and protease inhibitors (Roche #11836170001)) with a Dounce homogenizer. Lysates were subjected to 0.4*g* centrifugation for 5 min to remove the largest debris and protein content quantified by BCA (#23225, Thermo Fisher Scientific). Equal protein amounts were added to protein G magnetic beads (#10003D, Thermo Fisher Scientific) coated with anti-FLAG-M2 antibody (#F3165, Sigma). After an overnight incubation on rototor at 4 °C, beads were subjected to washes and finally heated 70 °C for 10 min in 1× SDS-PAGE loading dye to elute immunoprecipitated proteins that were subsequently immunoblotted with anti-TBPH, anti-Dicer2, anti-GAPDH, and anti-Actin.

### Immunoprecipitation for RNA enrichment

*Drosophila* heads collected by flash freezing in liquid nitrogen (elav-GAL4/UAS-TBPH and elav-GAL4/+;UAS-TBPH-RBD^mut^/+) were homogenized in immunoprecipitation buffer (20 mM HEPES, 150 mM NaCl, 0.5 mM EDTA, 10% glycerol, 0.1% Triton X-100, and 1 mM DTT plus protease inhibitors (Roche #11836170001)) with a Dounce homogenizer and the lysate subjected to 0.4*g* centrifugation for 5 min to remove the largest debris. Cleared lysates were added to protein G magnetic beads (#10003D, Thermo Fisher Scientific) coated with anti-FLAG-M2 antibody (#F3165, Sigma) and incubated 4 °C for half an hour. After five washes with immunoprecipitation buffer, beads were TRIzol (#15596-026, Ambion) treated to extract RNA.

### qRT-PCR

RNA was DNAse treated with the TURBO DNA-*free*™ Kit (#AM1907, Thermo Fisher Scientific) and retrotranscribed with Oligo (dT)_20_ Primer (#18418020, Thermo Fisher Scientific) and Superscript III Reverse Transcriptase (#18080-093, Thermo Fisher Scientific). Real-time PCR was performed with Platinum SYBR Green qPCR SuperMIX UDG (#11733-038, Thermo Fisher Scientific) on a Bio-Rad CFX96 qPCR System. Below are the used primers:

**Table:** 

Target	fw-primer	rv-primer
RpL11	5′-CCATCGGTATCTATGGTCTGGA-3′	5′-CATCGTATTTCTGCTGGAACCA-3′
Dcr-2	5′-GCTTTTATGTGGGTGAACAGGG-3′	5′-GGCTGTGCCAACAAGAACTT-3′
Syn	5′-TGTTCACGCAGGGCATCATC-3′	5′-GCCGTCTGCACATAGTCCATAG-3′
Accord	5′-GGCCTCTTAGGCATGGATCT-3′	5′-AGTGGAAGCCTTACCTTGCT-3′
Blastopia	5′-AGCTGTCTTCAGACGAACCG-3′	5′-TCGGGTGTACATCTTGGTGC-3′
Springer	5′-CATGGCGTGCAACAAAGTCA-3′	5′-GTTGCCCCTGGTGTTATGGA-3′
Burdock	5′-CCTTGTTGCGAACCCATGAC-3′	5′-TTCCCATACTGCCAACCTGG-3′
Gypsy	5′-GGCTCCACCGAAATCAAACA-3′	5′-GGCCTGTGTTAACAGGTCCA-3′
Homeless	5′-TGATCGGCACCGACTATGTCA-3′	5′-CTTGGCGTAGATGGACAAGTT-3′
Ago2	5′-GCTGGGCGATAGGCCATTTT-3′	5′-GGAGGCGTGTAAACCACATTA-3′
Loq	5′-GGCGGATCGGGCTTACAAG-3′	5′-CGTTTCGCTGACGAACTTTAAGG-3′
Piwi	5′-GTGCGCTCAGATCCCAAACT-3′	5′-AAGGCTACGGTTCTTGGTCG-3′
Pasha	5′-TGATGGTGACGGCGAAGAATA-3′	5′-ATCCCTCGGGTAGGACTTCAA-3′

### Statistical analysis

All statistical analysis was performed with Prism (GraphPad, USA) version 6.0. One-way ANOVA with Bonferroni correction and two-tailed *t* test was applied as a statistical test. In all figures, all the values were displayed as the mean and the standard error of the mean (SEM). Statistical significance was displayed as *p < 0.05, **p < 0.01, and ***p < 0.001.

## Supplementary information

**Additional file 1 **: **Table S1.** List of the 1253 Regulated Probesets (Fold-change ≥ 1,5; *P*-Value ≤ 0,05).

**Additional file 2 **: **Table S2.** List of the 1408 Regulated Probesets (Fold-change ≥ 1,5; P-Value ≤ 0,05).

**Additional file 3 **: **Fig. S1**. **a** Real time quantitative PCR of *blastopia*, *burdock* and *springer* transcript levels normalized on *Rpl11* (housekeeping) in *w*^1118^ - tbph^Δ23^,elav-GAL4/tbph^Δ23^; UAS-GFP/+ and tbph^Δ23^,elav-GAL4/tbph^Δ23^,UAS-TBPH. (2biological replicates, with 3 technical replicates for each), error bars SEM. **b** Microarray results of downregulated TEs in TBPH mutants: the fold change of TEs was reported for both tbph mutant alleles (Δ23 and Δ142) referred to *w*^1118^; TEs family and class were also indicated.

**Additional file 4 **: **Fig. S2. a** Number of peristaltic waves of Ctrl (*w*^1118^) and Δtb (tbph^Δ23^/ tbph^Δ23^) fed with NRTIs drugs (D) compared to vehicle only (V). *n*=20. **b** Number of peristaltic waves of Ctrl (*w*^1118^), Δtb-GFP-IR (tbph^Δ23^ /tbph^Δ23^; Repo-GAL4/UAS-GFP-IR) and Δtb-gypsy-IR_3_ (tbph^Δ23^/tbph^Δ23^; Repo-GAL4/UAS-gypsy-IR_3_). *n*=20. ns=not significant, ***p<0.001 calculated by one-way ANOVA, error bars SEM.

**Additional file 5 **: **Fig. S3**. Real time PCR of *Dicer-2* (*Dcr-2), Argonaute 2 (Ago2), Pasha, Piwi, Loquacious (Loq)* and *Homeless* transcript levels normalized on *Rpl11* (housekeeping) in adult heads of *w*^1118^, tbph^Δ23^/tbph^Δ23^ and tbph^Δ142^/tbph^Δ142^. *n*=2, error bars SEM.

**Additional file 6.** : Individual data values.

## Data Availability

All data generated or analyzed during this study are included in this published article and its supplementary information files.
