# Supplementary material for: TDP-43 prevents retrotransposon activation in the Drosophila motor system through regulation of Dicer-2 activity
Source: BMC Biol. 2020 Jul 3;18:82. doi: 10.1186/s12915-020-00816-1 (PMC7334854; doi:10.1186/s12915-020-00816-1)
Supplement: Supplementary file 1 — Additional file 1 : Table S1. List of the 1253 Regulated Probesets (Fold-change ≥ 1,5; P-Value ≤ 0,05). [file 12915_2020_816_MOESM1_ESM.pdf]

GenoSplice technology

Customer name:	Fabian Feiguin
Project name:	W1118 vs. Delta 23
Chip:	Affymetrix Drosophila Genome 2.0 Array
Organism:	Drosophilia
Analysis level:	Gene

Data Overview: Regulated probesets

	Up-regulated	Down-regulated	All
Number of regulated probesets	585	668	1253
Min fold-change	1.50	1.50	1.50
Average fold-change	3.30	4.12	3.74
Median of fold-change	1.92	1.95	1.93
Max fold-change	191.34	462.37	462.37
Gene with the max fold-change	CG10178	CG16713	CG16713

List of the 1253 Regulated Probesets (Fold-change ≥ 1,5; P-Value ≤ 0,05)

Affymetrix Probeset ID	Gene Symbol	Gene Name	Regulation	Fold-Change	P-Value	Representative Public ID	FlyBase ID	Entrez Gene ID (NCBI)	Intensity Delta 23	Intensity W1118
1628950_at	CG10178	---	up	191.34	1.97E-02	CG10178-RA	FBgn0032684	35105	7.75	1483.30
1628052_at	Cyp6a17	Cyp6a17	up	164.76	3.00E-04	CG10241-RA	FBgn0015714	45556	8.04	1323.83
1639594_at	CG40485	---	up	54.56	1.66E-02	CG40485-RB	FBgn0069973	3355165	84.30	4599.29
1634209_at	pim	pimples	up	41.89	1.71E-04	CG5052-RA	FBgn0003087	34404	2.02	84.57
1638338_a_at	Fbp1	Protein-1	up	30.48	2.36E-02	CG17285-RA	FBgn0000639	39566	11.98	365.11
1628238_at	Tektin-C	TEKTIN C	up	25.42	6.90E-04	CG10541-RA	FBgn0035638	38653	46.43	1180.34
1626774_s_at	robo	Roundabout 1	up	21.11	4.90E-03	CG13521-RA	FBgn0005631	37603	39.27	828.95
1627096_s_at	CG16778	BTB-protein-III	up	19.64	1.14E-03	CG16778-RA	FBgn0003715	38007	22.15	434.95
1639164_at	CG9486	---	up	18.14	8.68E-04	CG9486-RA	FBgn0031791	33874	18.07	327.77
1630728_a_at	phr	photolyase	up	15.81	1.02E-03	CG11205-RA	FBgn0003082	35735	43.75	691.90
1636757_at	CG14107	---	up	12.25	3.31E-04	CG14107-RA	FBgn0036351	39498	7.78	95.23
1633481_at	CG14394	Ninjurin C	up	12.14	2.49E-02	CG14394-RA	FBgn0038079	41568	107.72	1307.46
1623655_at	Yp2	yolk protein 2	up	11.07	9.84E-04	CG2979-RA	FBgn0005391	31938	1560.91	17278.86
1640853_at	CG15020	---	up	10.99	1.92E-02	CG15020-RA	FBgn0035543	38544	6.11	67.16
1640448_x_at	---	---	up	10.86	2.58E-04	Transposon.39	---	---	42.87	465.44
1637590_at	CG42329	CG14343	up	10.32	6.20E-03	CG5156-RA	FBgn0259229	33312	24.07	248.33
1623256_at	GstE1	glutathione-S-transferase	up	8.50	6.88E-07	CG5164-RA	FBgn0034335	37106	748.62	6365.75
1625915_at	CG4375	---	up	7.81	6.39E-03	CG4375-RA	FBgn0031295	33278	6.75	52.70
1632045_at	CG5697	---	up	7.63	3.65E-02	CG5697-RA	FBgn0038846	42483	93.79	715.67
1627324_at	lola	longitudinals lacking	up	7.61	7.98E-06	CG12052-RH	FBgn0005630	44548	47.81	363.89
1640035_at	Fbp2	fat body protein P6	up	7.32	2.45E-03	CG3763-RA	FBgn0000640	34259	19.70	144.13
1624864_s_at	dock	dreadlocks	up	7.19	1.65E-03	CG3727-RA	FBgn0010583	33262	162.64	1169.38
1638772_at	CG18107	---	up	7.06	4.96E-04	CG18107-RA	FBgn0034330	37101	2122.98	14978.95
1638869_at	Cpr51A	CG10112	up	6.92	2.11E-03	CG10112-RA	FBgn0033942	36613	99.65	690.04
1629479_a_at	fok	fledgling of Klp38B	up	6.63	2.65E-03	CG10746-RB	FBgn0020303	44380	1080.82	7164.52
1632527_at	CG13705	---	up	6.25	3.20E-03	CG13705-RA	FBgn0035582	38587	134.63	841.74
1623410_at	Pxd	Peroxidase	up	6.23	5.45E-03	CT11695	FBgn0004577	2768671	502.91	3135.03
1633780_at	CG13793	---	up	6.11	4.08E-03	CG13793-RA	FBgn0031935	34047	17.19	105.05
1635312_at	---	---	up	6.02	6.67E-03	SD06617	---	---	17.12	103.01
1630458_at	CheA7a	CheA7a	up	5.96	9.09E-06	CG15033-RA	FBgn0029948	31672	193.31	1151.98
1640666_at	CG31205	---	up	5.94	8.98E-04	CG31205-RA	FBgn0051205	318626	453.74	2694.07
1627841_at	CG11170	---	up	5.88	3.98E-05	CG11170-RB	FBgn0034705	37533	57.06	335.36
1634091_at	Cp19	Shell-19	up	5.84	2.07E-04	CG6524-RA	FBgn0000358	39000	53.13	310.28
1625433_at	Scp1	calcium biding protein	up	5.57	1.93E-02	CG15848-RA	FBgn0020908	3355102	842.53	4693.57
1633120_at	---	---	up	5.55	9.33E-03	HDC06051	---	---	11.18	62.09
1623000_at	CG33093	---	up	5.33	4.65E-03	CG33093-RA	FBgn0053093	42654	38.60	205.82
1641746_at	Cpr11B	CG2555	up	4.94	2.08E-05	CG2555-RA	FBgn0030398	32203	278.74	1376.52
1638374_at	Obp19b	Odorant-binding protein 19b	up	4.88	3.31E-07	CG1670-RA	FBgn0031110	33038	181.92	887.33
1628617_at	dro4	drosomycin-F	up	4.76	2.11E-02	CG32282-RA	FBgn0052282	317954	2369.02	11286.86
1640681_at	CG4950	---	up	4.68	1.35E-02	CG4950-RA	FBgn0036587	39783	161.81	756.81
1626086_at	CG10621	selenocysteine methyltransferase	up	4.56	6.91E-03	CG10621-RA	FBgn0032726	35152	368.19	1680.61
1630927_x_at	Pxd	Peroxidase	up	4.56	2.72E-02	CT11695	FBgn0004577	2768671	391.37	1783.04
1640684_at	CG6980	---	up	4.52	3.87E-03	CG6980-RA	FBgn0039228	42955	20.52	92.84
1640870_s_at	CG17322	---	up	4.32	1.56E-04	CG17322-RD	FBgn0027070	35139	98.68	426.62
1639982_at	CG15369	---	up	4.26	2.79E-02	CG15369-RA	FBgn0030105	31862	26.16	111.46
1635276_at	CG11437	---	up	4.21	1.70E-03	CG11437-RA	FBgn0037165	40470	42.06	177.05
1629545_at	Yp1	yolk protein 1	up	4.16	3.85E-03	CG2985-RA	FBgn0004045	31939	5914.61	24625.34
1624704_at	CG4741	---	up	4.10	8.78E-03	CG4741-RA	FBgn0035040	37939	55.65	228.20
1641317_at	squ	Squash	up	4.03	7.13E-04	HDC02844	FBgn0002652	2768920	21.91	88.30
1626830_at	CG6044 /// Lcp1	--- /// cuticle p. I	up	4.00	1.45E-02	CG11650-RA	FBgn0002531	35817 /// 37558	42.92	171.71
1637123_a_at	CG33281	---	up	3.98	1.42E-02	CG15407-RB	FBgn0053281	2768938	39.47	157.23
1632856_s_at	Vmat	Vesicular monoamine transporter	up	3.96	1.85E-03	CG6139-RB	FBgn0259816	3346192	23.69	93.89
1638475_a_at	CG16885	---	up	3.95	1.17E-02	CG16885-RB	FBgn0032538	34811	701.33	2769.32
1632705_at	ninaD	neither inactivation nor afterpotent	up	3.93	6.23E-03	CG31783-RA	FBgn0002939	326160	120.21	471.84
1627281_at	CG3117	---	up	3.92	1.38E-02	CG3117-RA	FBgn0031471	33484	25.07	98.27
1630085_s_at	Peritrophin-A	peritrophin A	up	3.90	1.07E-03	CG17058-RA	FBgn0022770	33023	415.30	1621.16
1640857_at	CG10208	---	up	3.89	1.63E-06	CG10208-RA	FBgn0039118	42814	326.53	1270.42
1631432_at	CG42749	---	up	3.83	2.26E-02	CG15786-RA	FBgn0029749	---	75.24	288.00
1624195_at	sug	sugarbabe	up	3.78	1.82E-02	CG3850-RA	FBgn0033782	36424	213.43	805.70
1623710_at	CG5847	---	up	3.70	5.77E-03	CG5847-RA	FBgn0036985	40255	66.28	245.48
1635131_at	CG4525	---	up	3.70	2.13E-03	CG4525-RA	FBgn0038358	41905	27.29	100.90
1631779_s_at	ptr	proximal to raf	up	3.66	5.75E-06	CG2841-RB	FBgn0003159	44529	98.73	361.79
1625314_at	CG2022	---	up	3.66	2.21E-03	CG2022-RA	FBgn0037292	40615	241.55	883.29
1632860_at	Cpr64Aa	CG15006	up	3.64	9.65E-05	CG15006-RA	FBgn0035510	38508	328.78	1195.25
1641481_at	Ugt86Dd	Ugt86Dd	up	3.62	3.38E-03	CG6633-RA	FBgn0040256	53507	160.95	582.08
1628555_at	Osi19	Osiris	up	3.56	2.09E-02	CG15189-RA	FBgn0037429	40776	33.20	118.03
1626392_s_at	Mef2	Complementation group C	up	3.47	8.91E-03	Transposon.2	---	36032	2154.60	7478.33
1624830_at	Act79B	79B Actin	up	3.45	1.93E-02	CG7478-RA	FBgn0000045	40444	1168.73	4037.36
1624732_at	GstE5	Glutathione S transferase E5	up	3.43	1.38E-04	CG17527-RA	FBgn0063495	37110	114.92	393.93
1638301_at	CG8157	---	up	3.41	5.71E-03	CG8157-RA	FBgn0034010	36700	246.45	840.47
1631728_s_at	PRL-1	PRL-1	up	3.39	3.25E-03	CG4993-RB	FBgn0024734	34952	1056.53	3585.46
1639098_s_at	CG2837	---	up	3.39	1.38E-04	CG2837-RB	FBgn0031646	33696	149.98	507.83
1623276_at	CG31663	---	up	3.38	5.23E-03	CG31663-RA	FBgn0051663	33363	142.17	480.80
1636811_at	Oatp33Ea	Organic anion transporting polype	up	3.36	2.12E-02	CG5427-RA	FBgn0032433	34660	265.15	891.94
1623834_at	CG18641	---	up	3.33	9.17E-03	CG18641-RA	FBgn0031426	33429	35.85	119.54
1630171_at	Cpr97Ea	CG6131	up	3.32	3.58E-02	CG6131-RA	FBgn0039480	43258	2232.35	7409.45
1636015_s_at	CG32850	---	up	3.30	1.84E-02	Transposon.97	FBgn0052850	318246	2408.86	7951.61
1630577_at	CG17378	---	up	3.28	4.57E-03	CG17378-RA	FBgn0031858	33951	24.03	78.90
1632870_at	CG32364	---	up	3.28	2.44E-02	CG32364-RA	FBgn0052364	317996	72.91	239.20
1635258_s_at	---	---	up	3.22	8.94E-03	Transposon.46	---	---	958.73	3083.20
1636853_at	Cpr47Ef	CG13214	up	3.21	1.80E-02	CG13214-RA	FBgn0033603	36194	255.90	820.84
1637536_at	CG14303	---	up	3.19	3.63E-02	CG14303-RA	FBgn0038633	42236	26.87	85.84
1632775_at	CG42254	CR30478	up	3.19	1.86E-02	CG12854-RA	FBgn0259112	36644	45.38	144.92
1628043_s_at	---	---	up	3.19	1.57E-02	HDC19168	---	---	19.98	63.65
1637914_at	Obp83g	Odorant-binding protein 83g	up	3.14	1.25E-02	CG31558-RA	FBgn0046875	170878	579.07	1817.22
1627588_at	CG12483	---	up	3.13	8.69E-05	CG12483-RA	FBgn0040688	50161	194.19	608.02
1634123_at	CG14304	---	up	3.13	3.41E-02	CG14304-RA	FBgn0038629	42232	137.08	429.02
1629609_at	GRHR	hormone receptor	up	3.12	4.09E-02	CG11325-RB	FBgn0025595	33942	20.99	65.54
1625792_s_at	HipHop	lethal (3) neo26	up	3.12	8.66E-04	CG6874-RA	FBgn0036815	40047	113.54	354.28
1631628_s_at	CG12896 /// Prx2540-1 /// Prx2540-2	AOP2-related /// peroxiredoxin 25	up	3.10	2.05E-02	CG11765-RA	/// FBgn0033520 ///	246663 /// 36098 /// 36101	5200.07	16123.93
1631302_at	CG16885	---	up	3.06	3.75E-03	CG16885-RA	FBgn0032538	34811	5418.80	16600.88
1635039_at	MED16	Mediator complex subunit 16	up	3.05	1.04E-02	CG5465-RA	FBgn0034707	37535	30.68	93.70
1636170_at	CG13607	---	up	3.01	3.46E-03	CG13607-RA	FBgn0039151	42859	191.69	576.89
1623524_at	CG7031	---	up	2.99	6.39E-03	CG7031-RA	FBgn0039027	42704	43.25	129.28
1635072_at	CG7214	---	up	2.96	7.53E-03	CG7214-RA	FBgn0031940	34053	5115.90	15126.56
1635483_at	CG16884 /// DsimCG16884	--- /// ---	up	2.95	1.98E-03	CG16884-RA	FBgn0028544	34812	4186.84	12342.13
1639209_at	---	---	up	2.93	4.33E-02	CG32143-RB	---	---	15.25	44.75
1628696_at	CG12643	---	up	2.92	2.36E-03	CG12643-RA	FBgn0040942	50416	1553.91	4545.16
1638221_at	Obp83cd	Odorant-binding protein 83d	up	2.92	2.80E-03	CG15582-RA	FBgn0046878	40746	69.40	202.58
1624211_at	CG9005	---	up	2.91	4.18E-03	CG9005-RA	FBgn0033638	36243	1055.38	3075.81
1627856_at	Hus1-like	Hus1-like	up	2.90	4.10E-02	CG2525-RA	FBgn0026417	40598	104.45	302.97
1641242_at	Ugt86Di	Ugt86Di	up	2.90	2.30E-03	CG6658-RA	FBgn0040251	53502	55.19	159.94
1625407_at	---	---	up	2.88	1.95E-02	CG40336-RA	FBgn0058336	---	12.36	35.54
1636676_at	CG2150	---	up	2.87	4.79E-03	CG2150-RA	FBgn0003065	43745	89.08	255.71
1639225_at	CG13711	---	up	2.85	2.26E-02	CG13711-RA	FBgn0035572	38576	13.29	37.87
1630558_a_at	Rgk2	Rgk2	up	2.84	1.71E-02	CG15069-RA	FBgn0085419	37132	70.81	200.80
1626891_at	CG15414	---	up	2.82	2.15E-02	CG15414-RA	FBgn0031542	33570	599.55	1689.59
1626353_at	CG15738	---	up	2.81	3.60E-04	CG15738-RA	FBgn0030352	32151	315.57	885.43
1633719_at	Ugt86Dg	Ugt86Dg	up	2.77	5.08E-03	CG17200-RA	FBgn0040253	53504	14.48	40.08
1634351_at	CG7860	---	up	2.77	2.62E-02	CG7860-RA	FBgn0030653	32488	220.29	609.61
1625884_at	CG13059	---	up	2.75	3.22E-02	CG13059-RA	FBgn0036607	39803	207.40	569.69
1629846_at	CG11619	---	up	2.74	1.34E-03	CG11619-RA	FBgn0036836	40072	95.04	260.11
1634019_at	CG2064	---	up	2.73	8.96E-05	CG2064-RA	FBgn0033205	35708	187.34	511.77
1637781_at	ald	monopolar spindles 1	up	2.73	4.09E-02	CG7643-RA	FBgn0000063	42126	20.80	56.79
1624409_a_at	Npc2h	CG11315	up	2.73	1.36E-02	CG11315-RB	FBgn0039801	43649	901.66	2460.47
1627801_at	CG9497	---	up	2.70	4.35E-04	CG9497-RA	FBgn0031800	33884	330.63	892.34
1637366_at	nimC4	CG16876	up	2.70	2.18E-03	CG16876-RA	FBgn0260011	34824	233.64	629.80
1637900_at	CG11852	---	up	2.69	3.57E-02	CG11852-RA	FBgn0039297	43035	1844.98	4963.60
1634462_at	CG3822	---	up	2.67	2.86E-04	CG3822-RA	FBgn0038837	42473	201.47	537.97
1628843_at	Ipod	Interaction partner of Dnmt2	up	2.67	1.35E-03	CG2961-RA	FBgn0030187	31954	42.42	113.23
1625583_at	CG16926	---	up	2.62	2.69E-03	CG16926-RA	FBgn0040732	50205	686.37	1801.45
1626674_at	CG34199	---	up	2.61	2.03E-02	HDC07468	FBgn0085228	5740128	25.42	66.38
1637562_at	TwdlT	GCR(ich)	up	2.60	2.18E-03	CG5812-RA	FBgn0029170	44790	363.07	945.57
1636311_at	Gpdh	5' gene	up	2.60	1.99E-02	CG9042-RA	FBgn0001128	33824	4940.37	12863.46
1641366_at	eIF4E-3	eIF4E-3	up	2.60	4.31E-02	CG8023-RA	FBgn0035860	38923	27.65	71.84
1628318_at	Spc105R	CG11451	up	2.59	3.40E-02	CG11451-RA	FBgn0037025	40300	26.75	69.20
1635129_at	CG6332	---	up	2.57	5.56E-03	CG6332-RA	FBgn0038921	42585	45.17	116.19
1626727_at	Mct1	Monocarboxylate transporter 1	up	2.56	3.09E-02	CG3456-RA	FBgn0023549	31198	162.96	417.91
1635263_at	CG11825	---	up	2.56	6.73E-03	CG11825-RA	FBgn0033519	36099	101.67	260.01
1629727_at	CG14258	---	up	2.54	4.49E-04	CG14258-RA	FBgn0039482	43260	89.66	227.33
1624982_s_at	CG5080	---	up	2.53	1.72E-02	CG5080-RB	FBgn0031313	33299	2272.97	5742.13
1627855_at	yellow-e	yellow-e	up	2.50	2.49E-04	CG9792-RA	FBgn0041711	41653	858.47	2149.11
1627927_at	CG8443	clueless	up	2.50	6.59E-03	CG8443-RA	FBgn0034087	36793	635.94	1590.88
1626588_a_at	CG5325	Peroxisomal farnesylated protein	up	2.50	4.71E-03	CG5325-RB	FBgn0032407	34630	400.00	999.82
1630256_at	lectin-24Db	Lectin24Db	up	2.49	3.20E-02	CG2958-RA	FBgn0040102	53550	88.50	220.42
1625773_at	CG6289	---	up	2.48	3.62E-02	CG6289-RA	FBgn0036970	40236	26.08	64.68
1626835_at	Syb	synaptobrevin	up	2.48	1.27E-02	CG12210-RB	FBgn0003660	36080	632.68	1567.07
1636888_a_at	Aph-4	Alkaline phosphatase	up	2.47	2.84E-03	CG1462-RA	FBgn0016123	43671	991.07	2445.55
1624606_at	CG13062	---	up	2.46	2.49E-02	CG13062-RA	FBgn0036603	39799	91.21	224.34
1635524_at	CG30427	---	up	2.46	1.39E-04	CG30427-RC	FBgn0043792	37986	419.39	1029.84
1635512_at	CG11893	---	up	2.45	9.31E-04	CG11893-RA	FBgn0039316	43056	336.32	823.82
1627943_at	Ilp3	Drosophila insulin-like peptide 3	up	2.44	2.17E-02	CG14167-RA	FBgn0044050	39151	260.71	636.35
1637055_s_at	---	---	up	2.44	3.94E-02	Transposon.52	---	---	48.64	118.62
1623757_at	Cry	drosocrystallin	up	2.41	1.75E-02	CG16963-RA	FBgn0005664	34604	7301.11	17618.46
1641365_s_at	---	---	up	2.40	4.95E-06	SD27140	---	---	1549.31	3721.83
1625063_a_at	CG31606	---	up	2.40	1.01E-02	CG31606-RB	FBgn0051606	318842	39.38	94.60
1628261_at	CG13067	---	up	2.40	9.99E-03	CG13067-RA	FBgn0036589	39785	1787.24	4280.55
1636118_at	CG13065	---	up	2.39	2.58E-02	CG13065-RA	FBgn0036590	39786	491.24	1174.66
1628655_at	CG4476	---	up	2.38	3.47E-02	CG4476-RB	FBgn0035969	39056	65.32	155.72
1623180_a_at	Hr46	Complementation group D	up	2.37	4.03E-02	CG33183-RC	FBgn0000448	36073	129.62	307.53
1639400_at	CG31207	---	up	2.37	6.13E-03	CG31207-RA	FBgn0051207	326125	1245.31	2947.00
1623372_at	CG5873	---	up	2.36	2.71E-02	CG5873-RA	FBgn0038511	42100	317.03	748.53
1633690_at	CG8323	---	up	2.35	1.60E-02	CG8323-RA	FBgn0033903	36566	299.46	704.43
1627872_at	CG3770	---	up	2.35	1.56E-02	CG3770-RA	FBgn0035085	37991	149.03	350.44
1639466_s_at	CG30101	---	up	2.35	3.67E-02	CT34188	FBgn0050101	36950	161.35	379.04
1626933_s_at	CG2469	---	up	2.35	1.70E-03	CG2469-RB	FBgn0035205	38149	891.72	2093.35
1636706_at	Myd88	krapfen	up	2.34	3.18E-02	CG2078-RA	FBgn0033402	35956	266.58	623.80
1639902_at	ventrally-expressed-protein-D	ventrally-expressed-protein-D	up	2.34	2.03E-04	CG33200-RA	FBgn0053200	117331	198.25	463.72
1640386_at	wbl	windbeutel	up	2.33	5.63E-03	CG7225-RA	FBgn0004003	37206	76.17	177.50
1634418_at	CG33281	---	up	2.33	1.24E-02	CG15407-RB	FBgn0053281	2768938	70.31	163.80
1626642_at	CG6486	---	up	2.32	1.04E-02	CG6486-RA	FBgn0035922	38994	48.59	112.93
1636131_at	lig3	DNA ligase III	up	2.32	3.76E-04	CG17227-RA	FBgn0038035	41518	304.65	707.98

1630632_s_at	CG2982	---	up	2.32	4.30E-02	CG2982-RB	FBgn0029704	31374	523.98	1216.20
1624907_at	Tsp42Ee	tetraspanin 42E	up	2.32	3.13E-02	CG10106-RA	FBgn0029506	35614	2113.87	4905.29
1637391_at	Cpr76Bd	CG9299	up	2.31	1.50E-02	CG9299-RA	FBgn0036881	40123	356.16	824.19
1629530_at	IM23	Immune induced molecule 23	up	2.31	1.89E-02	CG15066-RA	FBgn0034328	37099	8318.55	19240.46
1632986_a_at	CG15088	---	up	2.31	1.11E-03	CG15088-RB	FBgn0034381	37157	235.03	542.03
1637145_at	CG14787	---	up	2.30	2.19E-02	CG14787-RA	FBgn0027793	31096	87.46	201.37
1626967_at	CalpB	Calpain B	up	2.30	5.35E-05	CG8107-RA	FBgn0025866	39165	1720.39	3948.86
1640126_at	CG8249	---	up	2.29	5.80E-03	CG8249-RA	FBgn0034045	36742	155.44	356.31
1628184_at	Tsp3A	Tetraspanin 3A	up	2.29	1.98E-03	CG10742-RA	FBgn0040334	31244	92.79	212.34
1639142_s_at	---	---	up	2.29	1.37E-02	CR_tc_GH14469	FBgn0259936	---	136.37	311.94
1634742_at	Sodh-2	Sorbitol dehydrogenase-2	up	2.28	5.31E-04	CG4649-RA	FBgn0022359	41313	346.87	790.37
1637939_at	Spn43Aa	Serine protease inhibitor 1	up	2.28	2.63E-02	CG12172-RA	FBgn0024294	45041	29.22	66.54
1624745_at	Ilp5	Drosophila insulin-like peptide 5	up	2.26	4.98E-03	HDC09365	FBgn0044048	2768992	534.47	1208.44
1635028_s_at	CG11714 /// Fuca	--- /// alpha-L-fucosidase	up	2.26	8.09E-03	CG33047-RA	0036169 /// FBgn003	3772566 /// 3772574	131.15	296.03
1629333_at	---	---	up	2.25	2.30E-02	HDC18269	---	---	20.35	45.79
1640833_at	CG42237	phospholipase A2	up	2.25	1.31E-02	CG4346-RA	FBgn0250862	32261	208.64	469.02
1640397_at	CG34360	CG12802	up	2.25	1.27E-02	CG32469-RA	FBgn0250818	41217	79.17	177.84
1639460_a_at	CG17218	---	up	2.24	6.27E-03	CG17218-RA	FBgn0032421	34646	535.55	1201.72
1633097_at	CG14770 /// DsimCG14770	--- /// ---	up	2.24	4.11E-02	CG14770-RA	FBgn0029573	31085	430.81	965.27
1625365_at	CG17005	---	up	2.24	2.07E-02	CG17005-RA	FBgn0032109	34249	49.33	110.34
1640295_a_at	RhoGAP71E	RhoGAP71E	up	2.23	3.57E-02	CG32149-RB	FBgn0036518	39693	154.90	345.07
1641389_at	ckd	cracked	up	2.22	5.48E-04	CG14959-RA	FBgn0035427	38402	186.71	414.00
1627793_s_at	CG9134	---	up	2.21	1.45E-02	CG9134-RB	FBgn0035199	38141	145.78	322.16
1623706_at	CG9498	---	up	2.21	8.27E-04	CG9498-RA	FBgn0031801	33885	571.70	1263.26
1637490_at	CG13314	---	up	2.21	1.65E-02	CG13314-RA	FBgn0035949	39030	116.53	257.39
1633268_s_at	Nha1	serine protease	up	2.21	1.37E-02	CG10806-RB	FBgn0031865	33961	53.38	117.83
1629475_at	nudC	nudC	up	2.21	8.31E-03	CG9710-RA	FBgn0021768	39879	843.57	1860.17
1639999_at	CG2662	shrunken nuts	up	2.20	1.33E-02	CG2662-RA	FBgn0024993	31254	101.12	222.38
1632403_at	CG12263	---	up	2.20	4.95E-03	CG12263-RA	FBgn0034346	37118	166.83	366.87
1625389_at	CG8209	---	up	2.20	1.18E-02	CG8209-RA	FBgn0035830	38888	381.61	839.17
1632664_at	Osi12	Osiris	up	2.20	4.25E-03	CG1154-RA	FBgn0037419	40766	52.61	115.67
1628190_at	CG33981 /// fab1	BEST:GH01256 /// PtdIns(3)P 5-ki	up	2.19	3.12E-02	CG6355-RA	0028741 /// FBgn025	37033 /// 3885654	40.22	88.14
1632683_s_at	copiaGIP	gag-int-pol	up	2.18	5.70E-05	Transposon.11	---	---	13098.33	28605.91
1637077_s_at	CG3777	---	up	2.18	6.35E-03	CG3777-RB	FBgn0024989	30979	874.77	1908.84
1627502_at	CG9304	---	up	2.17	1.18E-02	CG9304-RA	FBgn0034674	37500	288.86	627.74
1635449_s_at	Dip-B	Dipeptidase B	up	2.17	6.64E-03	CG9285-RA	FBgn0000454	48450	1837.35	3992.11
1637723_at	mod(mdg4)	Modifier67.2	up	2.17	3.82E-04	CG32491-RW	---	49228	492.67	1069.79
1641567_at	lcs /// sesB	la costa /// lethal(1)9Ed	up	2.16	3.69E-02	CG12794-RA	FBgn0028583	32007 /// 33120	224.00	484.34
1641464_s_at	CG32850	---	up	2.16	7.20E-04	CG32850-RA	FBgn0052850	318246	2886.29	6240.05
1640489_at	CG18522 /// DsecCG18522	--- /// ---	up	2.16	1.07E-03	CG18522-RA	FBgn0038347	41894	464.28	1003.74
1635531_at	rost	rolling stone	up	2.16	1.44E-03	CG9552-RA	FBgn0011705	34222	689.84	1490.12
1636208_a_at	CG15097	---	up	2.16	1.89E-02	CG15097-RB	FBgn0034396	37172	287.26	619.77
1639734_at	CG4017	---	up	2.16	3.25E-02	CG4017-RA	FBgn0032143	34296	48.23	104.03
1630224_at	CG6410	---	up	2.15	2.91E-02	CG6410-RA	FBgn0034265	37015	194.77	418.16
1626184_at	CG16978	---	up	2.15	3.15E-03	CG16978-RA	FBgn0040972	50446	347.07	744.81
1637097_at	CG7497	---	up	2.14	4.56E-02	CG7497-RA	FBgn0036742	39966	606.57	1299.10
1634343_at	Rpt6R	---	up	2.14	2.97E-02	CG2241-RA	FBgn0039788	43635	247.30	528.95
1628885_at	CG32698	---	up	2.14	1.30E-02	CG32698-RA	FBgn0052698	31915	81.88	174.82
1629715_at	Gie	CG7891	up	2.13	1.85E-05	CG7891-RA	FBgn0037551	40961	684.08	1459.73
1623811_at	mRpL18	mitochondrial ribosomal protein L1	up	2.13	2.29E-03	CG12373-RA	FBgn0026741	36406	338.85	722.76
1641055_at	Hsp23	Protein 23	up	2.13	1.43E-02	CG4463-RA	FBgn0001224	39077	959.68	2045.35
1633741_at	---	---	up	2.13	1.22E-02	CG31032-RA	FBgn0051032	---	46.80	99.70
1634671_a_at	CG41284 /// CG42598	--- /// ---	up	2.13	2.98E-02	CG40386-RB	FBgn0085599	5740851 /// 8674042	32.38	68.96
1624797_at	CG1637	---	up	2.12	4.12E-02	CG1637-RA	FBgn0030245	32019	122.36	259.48
1629907_at	CG14989	---	up	2.12	1.31E-02	CG14989-RB	FBgn0035495	38485	2265.32	4801.11
1640795_at	CG13280	---	up	2.11	4.71E-02	CG13280-RA	FBgn0032609	35015	31.04	65.64
1630038_at	pyd3	pyd3	up	2.11	1.01E-02	CG3027-RA	FBgn0037513	40916	1076.54	2276.21
1632568_at	CG3223	---	up	2.11	3.19E-02	CG3223-RA	FBgn0037538	40947	970.59	2046.55
1639440_at	CG13285	---	up	2.11	4.12E-02	CG13285-RA	FBgn0035611	38623	160.57	338.30
1635161_at	---	---	up	2.11	4.28E-02	CG7532-RA	FBgn0028915	---	4242.76	8934.70
1625908_a_at	TppII	tripeptidyl peptidase II	up	2.10	4.00E-02	CG3991-RD	FBgn0020370	36444	3104.06	6522.92
1629039_at	asrij	asrij	up	2.09	1.28E-02	CG13533-RA	FBgn0034793	37637	159.67	333.96
1623666_at	CG10516	---	up	2.09	1.08E-03	CG10516-RA	FBgn0036549	39738	558.23	1164.99
1628699_at	Lsp1beta	Larval serum protein 1 beta	up	2.09	4.85E-02	CG4178-RA	FBgn0002563	33274	464.77	969.79
1638896_at	Cpr62Bc	CG1919	up	2.09	2.71E-03	CG1919-RA	FBgn0035281	38241	5606.41	11698.15
1638567_at	CG1092	---	up	2.07	4.14E-03	CG1092-RA	FBgn0037228	40538	220.06	455.51
1638621_at	CG31648	Complex IV assembly protein CO)	up	2.07	1.17E-02	CG31648-RA	FBgn0051648	33776	184.02	380.47
1633861_at	CG8145	---	up	2.06	4.27E-03	CG8145-RA	FBgn0037617	41038	129.36	267.11
1626749_a_at	CG18135	---	up	2.06	4.97E-02	CG18135-RB	FBgn0036837	40073	3523.84	7275.04
1639621_at	CG10916	---	up	2.06	6.22E-03	CG10916-RA	FBgn0034312	37081	341.27	704.56
1636797_at	Aats-ser	Seryl-tRNA synthetase	up	2.06	4.56E-03	CG4938-RA	FBgn0021750	41862	166.27	342.96
1640365_s_at	fusl	CG14021	up	2.06	7.17E-03	CG14021-RB	FBgn0031702	33765	426.46	878.88
1637051_at	RhoGAP71E	RhoGAP71E	up	2.06	3.59E-02	CG32149-RA	FBgn0036518	39693	110.00	226.60
1636045_at	---	---	up	2.06	2.18E-02	S.C3L001763	---	---	35.14	72.27
1628584_at	Cyp305a1	Cyp305a1	up	2.06	1.48E-03	CG8733-RA	FBgn0036910	40161	430.97	886.07
1626613_at	CG11236	---	up	2.06	7.72E-03	CG11236-RA	FBgn0031860	33955	264.03	542.75
1634580_at	bsk	Jun kinase	up	2.05	1.18E-02	CG5680-RC	---	44801	31.99	65.68
1624448_at	CG6356	---	up	2.05	1.62E-02	CG6356-RA	FBgn0039178	42893	440.95	902.84
1634341_a_at	CG6129	---	up	2.05	4.07E-03	CG6129-RB	FBgn0039152	42860	77.38	158.36
1633887_at	13857 /// DmauCG13857 /// DsimCG13	--- /// --- /// ---	up	2.04	2.88E-02	CG13857-RA	FBgn0038958	42627	53.39	109.17
1634809_s_at	CG30194	---	up	2.04	2.60E-02	CG30194-RA	FBgn0050194	37657	43.14	88.06
1636572_at	Duox	Dual oxidase	up	2.04	8.04E-03	CG3131-RA	FBgn0031464	33477	384.61	784.78
1625584_at	CG8654	---	up	2.04	2.23E-03	CG8654-RA	FBgn0034479	37275	1258.35	2566.03
1628935_at	CG10962	---	up	2.04	1.35E-02	CG10962-RB	FBgn0030073	31824	40.97	83.52
1635413_at	CG32266	---	up	2.03	1.06E-03	CG32266-RA	FBgn0052266	317945	99.57	202.37
1628915_s_at	---	---	up	2.03	2.83E-02	CG3799-RB	FBgn0027593	---	1349.48	2742.51
1623308_at	CG33784	---	up	2.03	8.72E-03	HDC12790	FBgn0053784	3772181	53.04	107.54
1638033_at	CG6055	---	up	2.02	2.34E-02	CG6055-RA	FBgn0031918	34027	66.52	134.68
1633939_at	CG13563	---	up	2.02	9.61E-03	CG13563-RA	FBgn0034966	37845	176.04	356.18
1632565_at	Bin1	Sin3A-associated polypeptide p18	up	2.02	9.14E-03	CG6046-RA	FBgn0024491	41965	439.24	888.28
1633166_at	CG12942	---	up	2.02	6.88E-03	CG12942-RA	FBgn0033569	36158	78.92	159.12
1638974_at	CG14963	---	up	2.01	1.00E-02	CG14963-RA	FBgn0035409	38383	32.69	65.79
1641125_at	CG1942	---	up	2.01	4.20E-02	CG1942-RA	FBgn0033215	35719	99.45	199.74
1625949_at	Gpdh	5' gene	up	2.01	9.62E-03	CG9042-RB	FBgn0001128	33824	1464.61	2939.27
1640224_s_at	CG13796	---	up	2.00	3.01E-02	CG13796-RB	FBgn0031939	34051	986.53	1977.46
1626341_at	Smyd4	CG14122	up	2.00	7.32E-03	CG14122-RA	FBgn0036282	39414	42.52	85.13
1626198_at	Cyp4d8	Cytochrome P450-4d8	up	2.00	7.95E-03	CG4321-RA	FBgn0015033	38841	160.62	321.49
1636338_at	CG15021	---	up	2.00	9.09E-03	CG15021-RA	FBgn0035544	38546	1276.07	2548.21
1629940_at	CG13895	---	up	2.00	2.37E-02	CG13895-RA	FBgn0035158	38087	128.30	256.04
1628289_at	ems	empty spiracles	up	1.99	3.76E-03	CG2988-RA	FBgn0000576	41697	63.68	126.85
1634062_at	CG14218	---	up	1.98	1.92E-02	CG14218-RA	FBgn0031031	32949	37.64	74.66
1629923_at	CG7371	---	up	1.98	3.01E-03	CG7371-RA	FBgn0031710	33774	221.93	439.65
1638724_at	CG18507	---	up	1.98	1.44E-02	CG18507-RA	FBgn0028527	34775	418.38	828.79
1624891_at	CG13183 /// DsecCG13183	--- /// ---	up	1.98	2.09E-03	CG13183-RA	FBgn0033667	36275	517.70	1023.40
1631954_at	Ranbp9	Ranbp9	up	1.98	2.70E-03	CG5252-RA	FBgn0037894	41367	210.14	415.37
1629638_at	conv	convoluted	up	1.97	2.50E-02	CG8561-RA	FBgn0033920	36588	260.77	514.68
1625710_at	CG31717	---	up	1.97	4.85E-02	CG31717-RA	FBgn0051717	318911	1198.46	2361.30
1634701_at	CG3713	---	up	1.97	1.88E-02	CG3713-RA	FBgn0040343	50365	677.54	1334.86
1635259_at	Mov34	Mov34	up	1.97	4.56E-02	CG3416-RA	FBgn0002787	37894	2946.70	5800.95
1624592_at	CG5188	---	up	1.97	1.94E-03	CG5188-RA	FBgn0032247	34428	142.27	279.80
1625678_at	Pi3K59F	dPI 3-kinase	up	1.97	6.81E-03	CG5373-RA	FBgn0015277	37733	130.84	257.21
1627302_at	Fmo-2	Flavin-containing monooxygenase	up	1.96	2.51E-03	CG3174-RA	FBgn0033079	35561	435.74	854.22
1630204_a_at	CG8136	---	up	1.96	4.41E-02	CG8136-RA	FBgn0037616	41037	60.60	118.55
1629803_a_at	scb	volado	up	1.96	2.10E-03	CG8095-RB	FBgn0003328	36692	649.67	1270.31
1629161_at	Obp56f	Odorant-binding protein 56f	up	1.95	4.09E-02	CG30450-RA	FBgn0043533	246668	53.87	105.30
1628834_a_at	SelD	Selenide,water dikinase	up	1.95	3.46E-03	CG8553-RB	FBgn0020615	36587	308.16	601.75
1627100_s_at	drm	drumstick	up	1.95	2.13E-04	CG10016-RB	FBgn0024244	49638	145.59	284.09
1628398_at	CG5853	---	up	1.95	1.15E-02	CG5853-RA	FBgn0032167	34322	871.95	1700.52
1624032_at	CG3887	---	up	1.95	3.47E-03	CG3887-RA	FBgn0031670	33725	1719.25	3351.39
1632373_s_at	CG34015	---	up	1.95	2.25E-02	Dm.X.57281.0	FBgn0054015	3885570	279.74	545.01
1641633_at	CG15544	---	up	1.95	1.63E-02	CG15544-RA	FBgn0039804	43655	266.33	518.18
1623066_at	Irk3	Inwardly rectifying potassium char	up	1.94	4.24E-03	CG10369-RA	FBgn0032706	35131	109.95	213.83
1635196_at	CG6836	---	up	1.94	1.31E-02	CG6836-RA	FBgn0036834	40070	379.52	737.83
1622908_a_at	CG10962	---	up	1.94	5.75E-03	CG10962-RA	FBgn0030073	31824	116.66	226.52
1632693_at	Lcp65Ac	Lcp65Ac	up	1.94	3.70E-02	CG6956-RA	FBgn0020642	38708	505.81	979.45
1626660_at	CG15024 /// Pdh	--- /// pigment cell dehydrogenase	up	1.93	7.20E-03	CG15024-RA	FBgn0040699	39812 /// 50172	390.48	755.56
1637195_at	CG31321	---	up	1.93	1.66E-02	CG31321-RB	FBgn0051321	318681	506.36	979.73
1628613_a_at	CG30492 /// CG30494	--- /// ---	up	1.93	7.44E-03	CG30492-RE	FBgn0050492	35698 /// 35699	562.54	1085.01
1628662_at	CG13531	---	up	1.93	5.28E-03	CG13531-RB	FBgn0034786	37627	499.91	964.13
1632278_s_at	---	---	up	1.93	2.92E-02	CG30193-RA	0050190 /// FBgn005	---	261.10	503.33
1641105_at	MED20	Mediator complex subunit 20	up	1.92	2.29E-02	CG18780-RA	FBgn0013531	43952	112.80	217.10
1636842_at	CG8460	---	up	1.91	8.20E-04	CG8460-RA	FBgn0031996	34115	155.64	297.27
AFFX-r2-Dros-GAPDH-3_at	---	---	up	1.91	1.06E-02	AFFX-Dm-Gpdh-3	---	---	1562.97	2985.21
1633991_at	CG31445	---	up	1.90	2.78E-03	CG31445-RA	FBgn0051445	326140	359.95	685.38
1633946_at	CG31955	---	up	1.90	4.75E-03	CG31955-RA	FBgn0051955	319045	265.64	505.59
1638616_at	mthl9	Mth-like 9	up	1.89	3.70E-02	CG17084-RA	FBgn0035131	38056	116.44	220.59
1627532_at	Rpb4	Rpb4	up	1.89	4.11E-04	CG31237-RA	FBgn0053520	326128	363.62	686.39
1640637_at	MED19	Mediator complex subunit 19	up	1.89	1.05E-02	CG5546-RA	FBgn0036761	39987	468.70	884.49
1631724_s_at	CG17746	---	up	1.89	3.28E-02	CG17746-RA	FBgn0035425	38400	3539.85	6678.71
1628712_at	CG17754	---	up	1.88	2.98E-03	CG17754-RC	FBgn0030114	31873	4148.40	7807.36
1630955_at	CG9870	---	up	1.88	6.60E-03	CG9870-RA	FBgn0031422	33425	71.33	134.17
1627207_at	CG14352	---	up	1.88	1.32E-02	CG14352-RA	FBgn0031351	33341	50.60	94.89
1638876_at	Rpn2	Rpn2	up	1.88	1.45E-02	CG11888-RA	FBgn0028692	43449	4661.31	8740.31
1635636_at	to	Takeout	up	1.87	3.85E-02	CG11853-RA	FBgn0039298	43036	250.47	467.81
1628458_at	milt	milton	up	1.87	4.29E-03	CG13777-RC	FBgn0022201	45683	1247.55	2329.64
1624423_at	CG5945	---	up	1.86	1.59E-03	CG5945-RA	FBgn0032494	34729	7894.96	14711.09
1623088_at	CG10855	---	up	1.86	3.15E-02	CG10855-RA	FBgn0035461	38443	123.84	230.66
1635321_at	CG14688	---	up	1.86	3.40E-02	CG14688-RA	FBgn0037819	41275	1750.08	3257.46
1623761_at	CG15347	---	up	1.86	1.89E-03	CG15347-RA	FBgn0030040	31779	60.52	112.63
1640358_at	CG4968	---	up	1.86	2.80E-02	CG4968-RA	FBgn0032214	34384	1161.36	2160.71
1626481_a_at	Taz	tafazzins	up	1.86	1.95E-02	CG8766-RA	FBgn0026619	36405	2420.58	4500.62
1636756_at	CG31627	---	up	1.86	3.89E-02	CG31627-RA	FBgn0051627	318860	57.08	106.10
1626694_at	---	---	up	1.86	3.07E-02	CG1674-RA	FBgn0039897	---	117.38	218.12
1630765_at	CG15547	---	up	1.85	4.17E-03	CG15547-RA	FBgn0039809	43661	92.26	170.79
1638484_at	Hsp67Bc	Gene 3	up	1.85	3.93E-03	CG4190-RA	FBgn0001229	39071	702.92	1300.92
AFFX-r2-Dros-GAPDH-M_a_at	---	---	up	1.85	3.64E-04	AFFX-Dm-Gpdh-M	---	---	5928.57	10971.69
1631307_at	CG11438	---	up	1.85	8.93E-03	CG11438-RA	FBgn0037164	40469	271.49	501.93
1637412_a_at	sas	responsible for dominant phenotyp	up	1.85	1.77E-04	CG2507-RB	FBgn0002306	40861	434.46	802.62
1637349_at	Rpn1	Rpn1	up	1.85	3.35E-02	CG7762-RA	FBgn0028695	40174	2477.47	4573.34
1623151_a_at	CG2010	---	up	1.85	8.63E-04	CG2010-RB	FBgn0039667	43475	343.79	634.52
1640952_at	CG1632	---	up	1.84	4.37E-03	CG1632-RA	FBgn0030027	31763	412.20	760.44
1639130_a_at	kkv	Chitin Synthase B	up	1.84	1.49E-02	CG2666-RA	FBgn0001311	45884	1958.45	3607.80
1631311_at	---	---	up	1.84	2.69E-02	CG14351-RA	FBgn0031349	---	49.67	91.45
1633624_s_at	alphaTub84D	alpha-Tubulin	up	1.84	3.25E-02	CG2512-RA	FBgn0003885	40904	78.02	143.56
1634468_at	CG13397	---	up	1.84	2.87E-03	CG13397-RA	FBgn0014417	46386	217.14	398.88
1638227_at	uif	SP1070	up	1.84	2.33E-03	CG9138-RA	FBgn0031879	33983	278.09	510.30
1623957_s_at	GstS1	glutathione-S-transferase	up	1.83	1.91E-04	CG8938-RB	FBgn0010226	36927	3133.87	5738.00
1633987_at	---	---	up	1.83	2.28E-02	CT35439	---	---	22.00	40.24
1641208_s_at	---	---	up	1.83	4.44E-02	SD10988	FBgn0046756	---	31.38	57.31
1622933_at	CG10344	---	up	1.82	4.78E-02	CG10344-RB	FBgn0034729	37565	29.14	53.17
1625522_at	---	---	up	1.82	1.02E-02	CG32551-RA	---	---	96.01	175.12
1631620_at	GlyP	glycogen phosphorylase b	up	1.82	6.94E-04	CG7254-RA	FBgn0004507	33386	6225.80	11347.55
1635208_at	CG9934	---	up	1.82	4.79E-02	CG9934-RA	FBgn0032467	34699	2997.98	5461.66
1639747_at	CG30291 /// grau	--- /// grauzone	up	1.82	8.25E-03	CG30291-RA	FBgn0050291	246534 /// 45871	233.35	423.90
1636750_s_at	CG13737	---	up	1.81	5.99E-03	CG13737-RA	FBgn0036382	39531	227.72	413.07
1635874_at	CG2794	---	up	1.81	3.62E-02	CG2794-RA	FBgn0031265	33233	108.57	196.84
1631874_at	---	---	up	1.81	1.94E-02	CG40295-RA	/// FBgn0085492 ///	---	128.12	231.82
1631112_at	Epac	Epac	up	1.81	8.07E-04	CG3427-RA	FBgn0085421	35588	405.25	732.28
AFFX-r2-Dros-GAPDH-3_a_at	---	---	up	1.80	2.26E-02	AFFX-Dm-Gpdh-3	---	---	8215.72	14807.01

1626534_at	tko	mitochondrial ribosomal protein S1	up	1.80	7.45E-03	CG7925-RB	FBgn0003714	31228	725.40	1307.00
1637161_s_at	myoglianin	Myoglianin	up	1.80	1.08E-02	CG1838-RA	FBgn0026199	43811	53.78	96.90
1624205_at	Tom40	Translocase of outer membrane 4	up	1.80	1.07E-02	CG12157-RA	FBgn0016041	44978	1212.00	2182.27
1616608_a_at	Gpdh	5' gene	up	1.80	2.52E-02	CG9042-RB	FBgn0001128	33824	8126.45	14622.93
1640710_at	Rpt4	Rpt4	up	1.79	4.73E-02	CG3455-RA	FBgn0028685	31567	4781.53	8575.65
1633264_at	CG13024	---	up	1.79	1.05E-02	CG13024-RA	FBgn0036665	39880	882.77	1580.70
1628087_s_at	CG10200	---	up	1.79	4.43E-02	CG10200-RB	FBgn0033968	36648	1507.98	2697.28
1632512_at	CG11820	---	up	1.78	8.78E-03	CG11820-RA	FBgn0039270	43004	311.81	556.20
1628245_at	sens-2	senseless-2	up	1.78	8.84E-03	CG31632-RA	FBgn0051632	33957	49.45	88.14
1630919_at	pip	pipe	up	1.78	1.68E-02	CG9614-RA	FBgn0003089	40104	174.41	310.88
1634162_at	CG40198	---	up	1.78	2.23E-03	CG40198-RA	FBgn0058198	3354922	2026.23	3608.27
1624074_at	Obp56a	Odorant-binding protein 56a	up	1.78	9.85E-03	CG11797-RA	FBgn0034468	37264	62.88	111.94
1638472_at	Cpr62Bb	CG13935	up	1.78	3.12E-02	CG13935-RA	FBgn0035280	38240	3144.84	5591.84
1630201_a_at	CG11321	---	up	1.77	1.73E-03	CG11321-RA	FBgn0031857	33950	284.66	504.92
1637545_at	prtp	pretaporter	up	1.77	2.59E-02	CG1837-RA	FBgn0030329	32124	2069.90	3670.29
1627389_at	---	---	up	1.77	2.43E-02	HDC07756	---	---	37.59	66.64
1635057_s_at	CG15445	---	up	1.77	2.48E-02	CG15445-RB	FBgn0031161	33094	1707.78	3024.99
1641169_s_at	CG11050	---	up	1.77	9.33E-03	CG11050-RA	FBgn0031836	33924	2475.77	4382.77
1631387_at	CG30344	---	up	1.77	3.65E-02	CG30344-RA	FBgn0050344	246552	1483.52	2623.52
1637936_at	CG32512	---	up	1.77	3.15E-03	CG32512-RA	FBgn0052512	33085	155.03	273.80
1626970_at	Ugt35b	UDP-glycosyltransferase	up	1.76	1.59E-02	CG6649-RA	FBgn0026314	41333	1716.07	3027.67
1626712_at	---	---	up	1.76	8.03E-03	S.C3L000077	---	---	29.45	51.90
1625140_at	NtR	NtR	up	1.76	3.52E-02	CG6698-RA	FBgn0029147	43935	265.09	467.02
1629889_s_at	regucalcin	regucalcin	up	1.76	5.59E-03	CG1803-RA	FBgn0030362	32165	2225.70	3919.09
1629559_s_at	Atet	ABC transporter expressed in trac	up	1.76	3.25E-04	CG2969-RA	FBgn0020762	33636	1491.64	2624.71
1633718_at	CG5355	---	up	1.76	1.35E-02	CG5355-RA	FBgn0032242	34421	918.83	1616.72
1627833_at	Cralbp	Cellular retinaldehyde binding prot	up	1.76	8.06E-03	CG10546-RA	FBgn0035636	38651	178.26	313.55
1631614_at	CG5190	---	up	1.76	1.98E-02	CG5190-RA	FBgn0034351	37124	140.30	246.63
1635006_at	/// DgriCG7077 /// DmojCG7077 /// Dvi	--- /// --- /// --- /// ---	up	1.75	1.15E-02	CG7077-RA	FBgn0038946	42614	2418.21	4234.51
1636046_at	Cda4	CG32499	up	1.75	1.69E-02	CG32499-RA	FBgn0052499	33144	892.15	1561.12
1641688_at	CG18136	---	up	1.74	8.38E-03	CG18136-RA	FBgn0036839	40075	139.71	243.66
1630669_at	CG7299	---	up	1.74	8.04E-03	CG7299-RA	FBgn0032282	34468	1651.45	2879.19
1635363_a_at	CG1832 /// Jupiter	--- /// Jupiter	up	1.74	3.34E-02	CG31363-RD	FBgn0051363	35445 /// 41392	3143.72	5476.81
1636158_at	CG8492	---	up	1.74	1.51E-02	CG8492-RA	FBgn0035813	38866	30.57	53.16
1625958_at	---	---	up	1.74	4.18E-03	HDC06189	---	---	33.47	58.18
1631671_at	CG13827	---	up	1.73	6.74E-03	CG13827-RA	FBgn0039068	42751	240.06	416.42
1625359_at	Tpl94D	transition protein-like[94D]	up	1.73	1.01E-02	CG31281-RA	FBgn0051281	318658	29.03	50.32
1624310_s_at	CG4753	---	up	1.73	1.35E-02	CG4753-RB	FBgn0036622	39819	383.36	664.09
1636668_at	spz5	CG9972	up	1.73	2.37E-02	CG9972-RA	FBgn0035379	38350	510.71	884.08
1623851_at	CG9400	---	up	1.73	9.57E-03	CG9400-RA	FBgn0030562	32385	2684.83	4638.58
1638562_a_at	Cyp6d5	Cyp6d5	up	1.73	6.88E-03	CG3050-RB	FBgn0038194	41706	4055.49	7001.69
1625417_s_at	CG14374 /// CG14377	--- /// ---	up	1.73	1.08E-02	CG14377-RA	0038148 /// FBgn004	41649 /// 50026	146.29	252.43
1639118_a_at	Hrb87F	Heterogeneous nuclear ribonucle	up	1.72	3.55E-03	CG12749-RA	FBgn0004237	48535	1242.38	2142.14
1634952_s_at	CG15094	---	up	1.72	1.48E-03	CG15094-RA	FBgn0034392	37168	345.18	594.17
1640429_a_at	CG6145	---	up	1.72	3.07E-03	CG6145-RA	FBgn0033853	36506	2586.21	4446.88
1631571_a_at	spir	spire	up	1.72	1.99E-02	CG10076-RD	FBgn0003475	45931	282.51	484.78
1623558_at	Cpr49Ae	CG8505	up	1.71	8.03E-04	CG8505-RA	FBgn0033728	36351	5965.53	10227.90
1637620_s_at	CG9186	---	up	1.71	3.94E-03	CG9186-RB	FBgn0035206	38150	1249.88	2139.19
1639140_at	casp	casper	up	1.71	1.59E-02	CG8400-RA	FBgn0034068	36769	1194.89	2044.14
1623298_at	CG6067	---	up	1.71	2.93E-02	CG6067-RA	FBgn0029828	31529	573.82	981.43
1639452_at	CG14984	---	up	1.71	4.41E-02	CG14984-RA	FBgn0035480	38467	1177.50	2012.55
1640616_at	CG17974	---	up	1.71	3.60E-02	CG17974-RA	FBgn0034624	37441	208.89	356.68
1634733_at	CG3831 /// DmirCG3831	--- /// ---	up	1.71	3.60E-02	CG3831-RA	FBgn0034804	37648	165.78	282.92
1626787_s_at	CG17556 /// CG3678	--- /// ---	up	1.71	3.24E-02	CG17556-RA	0038461 /// FBgn003	42045 /// 42046	1827.75	3117.82
1628013_at	CG3597	---	up	1.71	2.91E-02	CG3597-RA	FBgn0031417	33420	924.01	1575.73
1640280_at	lola	longitudinals lacking	up	1.70	5.62E-03	CG12052-RC	FBgn0005630	44548	713.93	1216.31
1623959_at	CG1532	---	up	1.70	1.82E-02	CG1532-RA	FBgn0031143	33076	776.71	1322.18
1624328_a_at	set-01	septin	up	1.70	2.35E-02	CG1403-RB	FBgn0011710	33114	94.06	160.07
1641564_a_at	Rb97D	Ribonuclear protein at 97D	up	1.70	3.97E-03	CG6354-RA	FBgn0004903	43231	429.15	729.53
1640462_at	Ho	Heme oxygenase	up	1.70	2.85E-02	CG14716-RA	FBgn0037933	41407	8320.50	14137.44
1630968_at	CG13907	---	up	1.70	1.70E-02	CG13907-RA	FBgn0035173	38104	2308.15	3919.51
1631549_s_at	CG18278 /// CG30059	--- /// ---	up	1.69	1.22E-02	CG18278-RA	0033836 /// FBgn005	246425 /// 36487	77.86	131.78
1633379_s_at	cnc	cap and collar	up	1.69	4.50E-02	CG17894-RC	FBgn0000338	42743	3432.50	5802.72
1625122_at	Obp99c	Odorant-binding protein 99c	up	1.69	1.65E-03	CG7584-RA	FBgn0039682	43494	12326.09	20824.01
1634503_at	CG15695	---	up	1.69	4.32E-02	CG15695-RA	FBgn0038832	42468	154.75	261.41
1641446_s_at	CG5171	---	up	1.69	5.47E-04	CG5171-RB	FBgn0031907	34016	1073.46	1812.99
1627999_s_at	barr	barren	up	1.69	2.16E-02	CG10726-RA	FBgn0014127	35287	127.79	215.73
1634630_at	CG5707	---	up	1.69	3.24E-02	CG5707-RA	FBgn0026593	48422	369.05	622.94
1628271_at	CG3609	---	up	1.69	4.35E-04	CG3609-RA	FBgn0031418	33421	972.16	1640.87
1627783_at	CG18599	---	up	1.69	2.87E-02	CG18599-RA	FBgn0038592	42191	62.97	106.28
1634874_at	AR-2	Allatostatin Receptor 2	up	1.69	2.50E-03	CG10001-RA	FBgn0039595	43393	72.29	121.94
1637814_s_at	Jafrac2	jafrac2	up	1.68	9.47E-03	CG1274-RA	FBgn0040308	53577	121.99	205.53
1635110_at	Cyp6a13	Cyp6a13	up	1.68	1.03E-03	CG2397-RA	FBgn0033304	35837	261.01	439.41
1634388_at	CG30197	---	up	1.68	2.59E-02	CG30197-RA	FBgn0050197	246513	13794.37	23217.16
1640405_at	l(2)01810	lethal (2) 01810	up	1.68	2.25E-03	CG5304-RA	FBgn0010497	47253	198.98	334.88
1623288_at	Pros26	Proteasome subunit 20S	up	1.68	4.39E-02	CG4097-RA	FBgn0002284	39855	4622.31	7767.48
1636915_at	CG11380	---	up	1.68	1.66E-02	CG11380-RA	FBgn0040359	31065	224.15	376.59
1627068_at	Cyp4aa1	Cyp4aa1	up	1.68	4.83E-02	CG8302-RA	FBgn0034053	36752	111.89	187.49
1630370_at	CG9796	---	up	1.68	1.73E-03	CG9796-RA	FBgn0038149	41650	2280.47	3820.37
1632166_at	Plap	Phospholipase A2 activator protei	up	1.67	3.92E-02	CG5105-RA	FBgn0024314	43978	407.52	679.81
1635318_at	Odc1	ornithine decarboxylase	up	1.67	1.22E-03	CG8721-RA	FBgn0013307	35766	325.05	541.84
1641699_at	TXBP181-like	TXBP181-like	up	1.66	2.31E-03	CG2072-RA	FBgn0026326	35954	321.19	534.78
1629641_s_at	---	---	up	1.66	3.82E-02	Transposon.65	---	---	132.97	221.23
1638324_s_at	bond	james bond	up	1.66	4.47E-03	CG6921-RB	FBgn0038985	42657	2171.10	3612.03
1623020_at	CG31937	---	up	1.66	1.31E-03	CG31937-RA	FBgn0031360	326177	554.10	921.85
1638469_s_at	CG3857	---	up	1.66	1.26E-02	Transposon.36	FBgn0023520	2768908	1279.01	2126.72
1632699_s_at	Adh /// Adhr	alcohol dehydrogenase /// Adh-rel	up	1.66	1.61E-02	CG32954-RA	0000055 /// FBgn000	3771877 /// 3772432	225.94	375.16
1640901_at	CG6753	---	up	1.66	4.95E-02	CG6753-RA	FBgn0038070	41557	113.29	188.08
1633106_at	CG6662	---	up	1.66	2.07E-02	CG6662-RA	FBgn0035907	38975	204.40	339.26
1641558_at	CG31005	---	up	1.66	9.61E-03	CG31005-RA	FBgn0051005	43697	1157.12	1918.88
1627399_at	CG17266	---	up	1.66	4.15E-02	CG17266-RA	FBgn0033089	35571	57.54	95.36
1641740_at	CG34370	CG13499	up	1.66	1.08E-02	CG30408-RA	FBgn0085399	5740565	154.80	256.29
1638778_at	CG10352	---	up	1.65	1.13E-03	CG10352-RA	FBgn0030348	32147	266.94	441.75
1626666_s_at	RhoGAP16F	RhoGAP16F	up	1.65	4.45E-02	CG7122-RB	FBgn0030893	32790	66.09	109.35
1639834_at	G14291 /// DpseGA12883 /// DvirGJ141	--- /// GA12883 /// CG14291	up	1.65	1.19E-02	CG14291-RA	FBgn0038660	42266	177.97	294.40
1641579_at	mRpS26	mitochondrial ribosomal protein S2	up	1.65	2.41E-02	CG7354-RA	FBgn0036774	40001	340.31	562.91
1630289_at	CG6435	---	up	1.65	1.39E-02	CG6435-RA	FBgn0034165	36894	708.68	1170.65
1638522_at	CG12123 /// DsimCG12123	--- /// ---	up	1.65	3.10E-02	CG12123-RA	FBgn0030039	31777	300.64	496.44
1636909_at	CG4078	---	up	1.65	2.87E-02	CG4078-RA	FBgn0029798	31497	124.40	205.33
1635390_s_at	CG9331	---	up	1.65	1.81E-02	CG9331-RB	FBgn0032889	35347	2702.05	4458.83
1627678_at	CG18302	---	up	1.65	2.36E-02	CG18302-RA	FBgn0032266	34452	551.28	909.08
1631167_at	CG5023	---	up	1.65	4.32E-02	CG5023-RA	FBgn0038774	42400	1160.82	1913.88
1637293_at	CG17707	---	up	1.65	2.88E-02	CG17707-RA	FBgn0025835	5740408	385.66	635.80
1631217_a_at	TpnC73F	troponin C	up	1.65	2.15E-02	CG7930-RA	FBgn0010424	39916	126.61	208.65
1633830_at	CG10195	---	up	1.65	2.52E-02	CG10195-RA	FBgn0032787	35228	169.32	278.86
1635838_at	CG13545	---	up	1.65	2.43E-02	CG13545-RA	FBgn0034828	37675	101.64	167.36
1626326_at	CG31436	---	up	1.65	9.33E-03	CG31436-RA	FBgn0051436	318734	542.10	892.48
1630399_at	Cad99C	Cad99C	up	1.65	1.96E-02	CG31009-RA	FBgn0039709	43528	365.97	602.13
1633303_at	Cht6	CG2989	up	1.65	2.16E-02	CG2989-RA	FBgn0030171	31935	527.61	867.98
1639597_at	Obp44a	Odorant-binding protein 44a	up	1.64	3.42E-02	CG2297-RA	FBgn0033268	35789	18343.41	30163.94
1641035_a_at	/// DgriCG5854 /// DmojCG5854 /// Dvi	--- /// --- /// --- /// ---	up	1.64	4.00E-04	CG5854-RA	FBgn0039130	42831	705.10	1159.19
1623934_at	blp	black pearl	up	1.64	1.16E-02	CG5268-RA	FBgn0038387	41937	504.26	828.89
1623103_at	CG14946	---	up	1.64	3.19E-02	CG14946-RA	FBgn0032405	34627	573.32	940.57
1625757_at	gfzf	GST-containing FLYWCH zinc-fing	up	1.64	7.57E-04	CG31492-RA	FBgn0250732	40858	1081.69	1774.55
1626990_s_at	Mipp1	Multiple inositol polyphosphate ph	up	1.64	9.27E-03	CG4123-RB	FBgn0026061	39841	1174.87	1924.70
1630584_at	CG10657	retinaldehyde-binding related prot	up	1.64	2.15E-03	CG10657-RA	FBgn0036289	39423	623.38	1020.82
1632590_at	CG31320	---	up	1.63	1.35E-02	CG31320-RA	FBgn0051320	318680	74.18	121.20
1636718_s_at	CG4502	---	up	1.63	3.38E-05	CG4502-RB	FBgn0031896	34002	3627.14	5925.85
1625665_at	mRpL39	mitochondrial ribosomal protein L3	up	1.63	5.44E-03	CG17166-RA	FBgn0036462	39627	475.69	777.08
1639636_at	Cht7	CG1869	up	1.63	1.81E-02	CG1869-RA	FBgn0035398	38370	1526.53	2493.32
1632280_at	mRpS35	mitochondrial ribosomal protein S3	up	1.63	1.46E-04	CG2101-RA	FBgn0035374	38345	432.25	705.25
1639396_s_at	CDase	Ceramidase	up	1.63	2.06E-02	CG1471-RB	FBgn0039774	43618	743.23	1211.67
AFFX-r2-Dros-GAPDH-5_a_at	---	---	up	1.63	3.74E-02	AFFX-Dm-Gpdh-5	---	---	293.19	477.88
1624029_at	CG3608	---	up	1.63	5.12E-03	CG3608-RA	FBgn0035039	37938	184.40	300.29
1624017_at	Ahcy89E	AdoHcyase-like	up	1.62	1.08E-02	CG8956-RA	FBgn0015011	42043	420.38	682.86
1640352_at	CG13779	---	up	1.62	1.72E-02	CG13779-RA	FBgn0040954	50428	5064.94	8227.04
1630258_at	GstD2	Glutathione S transferase D2	up	1.62	3.72E-02	CG4181-RA	FBgn0010038	48335	257.62	418.37
1626979_at	Gr92a	Gustatory receptor 92a	up	1.62	4.82E-02	CG31208-RA	FBgn0045471	117473	34.16	55.47
1641432_a_at	Prosalpha5	20s proteasome	up	1.62	4.92E-02	CG10938-RA	FBgn0016697	36951	5785.47	9391.19
1634125_at	CG30440	---	up	1.62	1.58E-02	CG30440-RA	FBgn0050440	35496	213.77	346.94
1638365_at	CG3244	---	up	1.62	6.19E-03	CG3244-RA	FBgn0031629	33678	5930.28	9616.81
1635298_at	CG13458	---	up	1.62	3.51E-02	CG13458-RA	FBgn0036479	39646	155.44	251.75
1622984_at	Sod2	mitochodnrial SOD	up	1.62	2.27E-03	CG8905-RA	FBgn0010213	36878	2813.69	4555.81
1637036_s_at	pr	purple	up	1.62	4.99E-02	CG16784-RC	FBgn0003141	35292	7226.04	11698.49
1634165_at	CG11378	---	up	1.62	1.41E-03	CG11378-RA	FBgn0040364	31060	1148.17	1857.36
1624658_at	CG33158	---	up	1.61	7.94E-03	CG33158-RB	FBgn0053158	39834	140.83	227.36
1627277_s_at	CG2930	---	up	1.61	8.72E-03	CG2930-RA	FBgn0028491	31341	310.95	501.82
1629523_at	lola	longitudinals lacking	up	1.61	9.26E-03	CG12052-RS	FBgn0005630	44548	205.16	331.04
1638975_at	CG13876	---	up	1.61	1.76E-02	CG13876-RA	FBgn0035109	38031	108.02	174.04
1626204_at	CG42246	---	up	1.61	4.25E-02	S.CX000803	FBgn0259098	7354415	180.43	290.65
1639981_at	Ssb-c31a	Single stranded-binding protein c3	up	1.61	2.53E-03	CG8396-RA	FBgn0015299	34120	846.95	1363.73
1633523_at	Pbgs	Porphobilinogen synthase	up	1.61	2.41E-02	CG10335-RA	FBgn0036271	39402	200.75	322.72
1640131_a_at	CG5270	---	up	1.61	6.29E-03	CG5270-RB	FBgn0037897	41370	376.89	605.75
1624553_a_at	Rcd7	Reduction in Cnn Dots 7	up	1.61	4.12E-02	CG14098-RA	FBgn0036884	40126	61.52	98.84
1639256_at	CG5676	---	up	1.61	1.92E-04	CG5676-RA	FBgn0032200	34368	1847.90	2968.87
1628008_a_at	TER94	ter94	up	1.60	1.74E-02	CG2331-RA	FBgn0024923	36040	6691.82	10732.70
1630593_at	NLaz	Neural Lazarillo	up	1.60	1.08E-02	CG33126-RA	FBgn0053126	33324	2571.31	4121.32
1625587_at	Jheh3	Juvenile hormone epoxide hydrola	up	1.60	3.68E-03	CG15106-RA	FBgn0034406	37182	207.84	332.96
1624919_at	CG8525	---	up	1.60	3.21E-02	CG8525-RA	FBgn0033735	36358	262.52	420.26
1625503_at	CG32425	---	up	1.60	3.44E-02	CG32425-RB	FBgn0052425	40273	1940.24	3105.73
1635062_at	Smg1	FRAP-related	up	1.60	4.71E-03	CG32743-RA	FBgn0052743	31625	363.51	580.20
1641042_at	CG16820	---	up	1.59	8.29E-03	CG16820-RA	FBgn0032495	34730	635.37	1012.89
1634707_s_at	Gfat1	Glutamine:fructose-6-phosphate a	up	1.59	2.36E-02	CG12449-RA	FBgn0027341	3354921	3321.09	5290.26
1626846_s_at	CG5973	---	up	1.59	1.22E-03	CG5973-RA	FBgn0031914	34023	1964.10	3125.59
1631330_at	CG3408	---	up	1.59	2.84E-02	CG3408-RA	FBgn0036008	39107	132.84	211.33
1640610_at	CG6613	---	up	1.59	2.55E-02	CG6613-RA	FBgn0034694	37520	249.39	396.63
1631487_s_at	CG32626	---	up	1.59	2.93E-02	CT36016	FBgn0052626	32352	124.41	197.84
1623740_at	CG32154	---	up	1.59	4.89E-02	CG32154-RA	FBgn0052154	39778	128.26	203.92
1625195_s_at	shn	schnurri	up	1.59	1.68E-02	Transposon.24	---	36171	2283.81	3630.07
1633770_at	CG11967	---	up	1.59	6.43E-03	CG11967-RA	FBgn0037646	41070	427.68	679.25
1637638_s_at	CG10702	---	up	1.59	2.45E-02	CG10702-RB	FBgn0032752	35181	480.39	762.46
1636837_at	CG32177	---	up	1.59	1.23E-03	CG32177-RA	FBgn0052177	326197	1053.60	1672.23
1622973_at	CG8031	---	up	1.59	2.74E-02	CG8031-RA	FBgn0038110	41601	808.69	1282.70
1630168_at	CG31636	---	up	1.58	4.94E-02	CG31636-RA	FBgn0051636	318865	100.37	159.08
1624463_s_at	pr-set7	pr-set7	up	1.58	2.34E-02	CG3307-RC	FBgn0011474	41743	285.78	452.58
1624689_at	---	---	up	1.58	9.94E-03	HDC08982	---	---	108.49	171.61
1635272_a_at	CG10205	---	up	1.58	1.49E-02	CG10205-RB	FBgn0033970	36650	1628.41	2572.08
1641703_at	CG2147	---	up	1.58	1.12E-02	CG2147-RA	FBgn0030025	31759	313.94	495.65
1640884_at	CG15784	---	up	1.58	2.04E-02	CG15784-RA	FBgn0029766	31461	936.13	1476.67
1624861_at	AlkB	AlkB	up	1.58	1.41E-02	AlkB-RA	FBgn0065035	2768870	123.76	195.19
1634983_s_at	CG32521	---	up	1.58	2.00E-03	CG32521-RA	FBgn0052521	33098	3430.80	5408.83
1628466_s_at	Rpn6	Proteasome p44.5 subunit	up	1.58	5.83E-03	CG10149-RA	FBgn0028689	36638	7088.43	11165.93
1623555_at	CG10131	---	up	1.57	3.03E-02	CG10131-RA	FBgn0033949	36624	172.70	271.78
1636845_at	CG8885	Sco1 protein homolog	up	1.57	1.46E-04	CG8885-RA	FBgn0031656	33711	545.47	858.03
1628599_at	Psf2	Psf2	up	1.57	2.27E-04	CG18013-RA	FBgn0031599	33641	160.17	251.42
1629428_at	CG7342	---	up	1.57	2.47E-02	CG7342-RA	FBgn0038716	42335	115.21	180.82
1634668_at	Ice	Ice	up	1.57	5.46E-03	CG7788-RA	FBgn0019972	43514	1194.76	1873.39
1634002_at	CG13315	---	up	1.57	4.36E-03	CG13315-RA	FBgn0040827	50300	8430.36	13205.54
1628353_at	GstD1	CG10045 Glutathione S-transfera	up	1.57	3.58E-03	CG10045-RA	FBgn0001149	41503	8333.10	13052.02

1631718_at	mRpL12	mitochondrial ribosomal protein L1	up	1.57	2.68E-03	CG5012-RA	FBgn0011787	44326	844.12	1321.27
1626059_at	knrl	knirps-related	up	1.57	2.41E-02	CG4761-RA	FBgn0001323	40285	192.07	300.61
1640159_at	---	---	up	1.56	6.23E-03	CG13377-RA	FBgn0040369	---	996.10	1558.88
1627342_at	CG15251	---	up	1.56	8.83E-03	CG15251-RA	FBgn0030166	31930	96.22	150.52
1637027_s_at	CG10165	---	up	1.56	7.53E-03	CG10165-RA	FBgn0032801	35242	228.48	357.36
1630233_at	CG5399	---	up	1.56	1.92E-02	CG5399-RA	FBgn0038353	41900	212.64	332.51
1630679_at	CG42249	CG1961	up	1.56	4.26E-02	CG1961-RA	FBgn0259101	32043	77.30	120.88
1625297_at	CG8128	---	up	1.56	8.54E-03	CG8128-RA	FBgn0030668	32504	1660.59	2594.04
1632390_at	cer	crammer	up	1.56	8.73E-03	CG10460-RA	FBgn0034443	37229	1933.82	3019.98
1628536_s_at	CG11880	---	up	1.56	1.88E-02	CG11880-RA	FBgn0039637	43440	1344.24	2093.69
1635683_at	CG6136	---	up	1.56	1.81E-02	CG6136-RA	FBgn0038332	41877	75.93	118.22
1638112_at	CG1908	---	up	1.56	4.99E-02	CG1908-RA	FBgn0030274	32055	1101.88	1715.06
1634606_a_at	mmy	cabrio	up	1.56	6.21E-04	CG9535-RA	FBgn0259749	33903	1700.45	2644.40
1624616_at	Trh	Tryptophan hydroxylase	up	1.56	1.28E-04	CG9122-RA	FBgn0035187	38121	155.97	242.55
1637736_a_at	GalNAc-T2	polypeptide GalNAc transferase 7	up	1.55	8.51E-03	CG6394-RA	FBgn0030930	32836	196.69	305.37
1624818_s_at	Sply	Sphingosine-1-phosphate lyase	up	1.55	1.86E-02	CG8946-RA	FBgn0010591	46059	1127.83	1746.20
1629822_a_at	CG15506	---	up	1.55	3.25E-02	CG15506-RA	FBgn0039686	43498	336.82	520.87
1639388_a_at	CG12567	---	up	1.54	2.35E-02	CG12567-RB	FBgn0039958	3355094	707.98	1092.25
1629070_at	CG1208	---	up	1.54	1.71E-02	CG1208-RA	FBgn0037386	40727	773.32	1192.62
1631273_at	CG5262	---	up	1.54	2.64E-02	CG5262-RA	FBgn0036988	40258	441.34	680.43
1629843_s_at	Ubc-E2H	Ubc-E2H	up	1.54	2.81E-02	CG2257-RA	FBgn0029996	31728	2628.32	4049.79
1622999_at	set-01	septin	up	1.54	3.26E-02	CG1403-RA	FBgn0011710	33114	708.25	1091.12
1629023_s_at	CG40191	---	up	1.54	1.97E-02	CG40191-RC	FBgn0058191	3354930	1098.51	1691.90
1639253_at	CG11242	---	up	1.54	2.45E-02	CG11242-RA	FBgn0034451	37244	1282.43	1974.22
1627023_at	CG4884	---	up	1.54	3.12E-02	CG4884-RA	FBgn0039565	43357	223.47	343.83
1633153_at	cdc14	CG7134	up	1.54	4.19E-02	CG7134-RA	FBgn0031952	34067	169.28	260.37
1637038_at	tex	tex	up	1.54	2.88E-02	CG9615-RA	FBgn0037569	40983	81.17	124.84
1624695_at	CG3999 /// DsecCG3999	--- /// ---	up	1.54	4.43E-02	CG3999-RA	FBgn0037801	41253	487.67	749.85
1625312_at	CG10553	---	up	1.54	2.49E-02	CG10553-RA	FBgn0039324	43064	1592.08	2446.90
1623497_at	HLHm3	split locus enhancer protein m3	up	1.53	3.92E-02	CG8346-RA	FBgn0002609	43156	194.31	298.24
1641307_at	CG14419	---	up	1.53	4.24E-02	CG14419-RA	FBgn0029639	31276	1662.39	2551.39
1630280_s_at	CG42353 /// CG42354	--- /// ---	up	1.53	3.66E-02	CG9216-RA	0259699 /// FBgn025	32576 /// 7354437	5323.35	8169.82
1627641_s_at	CG17494	---	up	1.53	1.59E-02	CG17494-RA	FBgn0040011	3355127	618.97	948.08
1635163_at	CG5731	---	up	1.53	1.12E-02	CG5731-RA	FBgn0032192	34355	335.02	512.84
1641454_at	CG1461	---	up	1.53	1.82E-02	CG1461-RA	FBgn0030558	32381	2869.65	4392.63
1628792_at	Obp56g	Odorant-binding protein 56g	up	1.53	3.30E-02	CG13873-RA	FBgn0034474	37270	2863.43	4381.81
1624981_a_at	CG10576	proliferation-associated protein	up	1.53	3.64E-03	CG10576-RB	FBgn0035630	38645	840.22	1285.31
1638730_at	Hsp67Ba	Gene 1	up	1.53	3.93E-02	CG4167-RA	FBgn0001227	39076	111.38	170.07
1633092_at	cup	cup	up	1.53	2.45E-02	CG11181-RA	FBgn0000392	33934	950.38	1449.69
1637127_at	CG10958	---	up	1.52	7.78E-03	CG10958-RA	FBgn0030004	31736	106.91	163.03
1627835_a_at	Cda5	CG31973	up	1.52	4.89E-02	CG31973-RA	FBgn0051973	33158	1904.73	2900.49
1627693_at	CG1965	---	up	1.52	7.26E-04	CG1965-RA	FBgn0037466	40842	498.70	759.08
1635975_s_at	---	---	up	1.52	2.68E-02	RE54004	FBgn0047095	---	1158.74	1763.40
1630729_at	tefu	telomere fusion	up	1.52	2.11E-02	CG6535-RA	FBgn0045035	41839	123.34	187.70
1641108_at	CG17928	---	up	1.52	2.39E-02	CG17928-RA	FBgn0032603	35009	618.65	941.31
1633701_at	CG16985	---	up	1.52	9.82E-05	CG16985-RA	FBgn0035355	38323	596.68	906.82
1623537_at	---	---	up	1.52	2.67E-02	CT35952	---	---	83.40	126.72
1636151_at	CG32196	---	up	1.52	3.55E-03	CG32196-RA	FBgn0052196	317908	206.74	313.65
1631925_at	Mdr65	P-glycoprotein65-Dm	up	1.52	1.23E-03	CG10181-RA	FBgn0004513	38726	1742.16	2642.94
1634170_a_at	jbug	filamin	up	1.52	4.23E-02	CG30092-RB	FBgn0028371	43997	501.29	760.22
1636440_at	Sip1	SRY interacting protein 1	up	1.52	2.79E-03	CG10939-RA	FBgn0010620	44155	1106.01	1677.13
1638276_at	Obp56e	Odorant-binding protein 56e	up	1.51	9.95E-03	CG8462-RA	FBgn0034471	37267	10012.24	15161.37
1638795_at	sec6	sec6	up	1.51	4.68E-02	CG5341-RA	FBgn0034367	37142	231.64	350.64
1630348_a_at	CG5059	---	up	1.51	3.20E-02	CG5059-RA	FBgn0037007	40278	5403.38	8165.26
1636075_at	pch2	CG31453	up	1.51	1.63E-02	CG31453-RA	FBgn0051453	41013	95.41	144.15
1625683_at	CG5112	---	up	1.51	7.30E-03	CG5112-RA	FBgn0039341	43083	690.99	1043.43
1632839_a_at	CG8312	---	up	1.51	6.68E-03	CG8312-RA	FBgn0037720	41163	655.58	987.69
1640738_a_at	spn-A	Spindle-A	up	1.50	2.84E-02	CG7948-RA	FBgn0003479	43577	143.14	215.19
1630408_s_at	CG7033	---	up	1.50	2.09E-03	CG7033-RC	FBgn0030086	31838	1338.02	2011.03
1628652_at	Myo10A	Myosin XV	up	1.50	1.33E-02	CG2174-RA	FBgn0030252	32028	501.42	753.52
1626586_at	Obp57a	Odorant-binding protein 57a	up	1.50	3.25E-02	CG30141-RA	FBgn0043535	246670	722.57	1085.34
1641400_at	CG9170	---	up	1.50	2.81E-02	CG9170-RA	FBgn0030716	32563	1013.62	1522.18
1633684_at	CG32444	aldose 1-epimerase	up	1.50	1.74E-03	CG32444-RA	FBgn0043783	40406	4357.18	6542.02
1634087_at	CG1311	---	up	1.50	6.18E-04	CG1311-RA	FBgn0035523	38523	469.84	705.33
1632298_s_at	mfas	Midline Fasciclin	up	1.50	2.39E-02	CG3359-RB	FBgn0024211	41455	2215.74	3324.12
1624057_at	CG16713	---	down	462.37	4.23E-02	CG16713-RA	FBgn0031560	33591	4607.77	9.97
1626088_at	CG2177	---	down	119.00	2.77E-04	CG2177-RA	FBgn0039902	43786	1031.33	8.67
1633386_s_at	mthl8	Mth-like 8	down	118.15	7.30E-03	CT37020	FBgn0052475	38013	391.21	3.31
1625965_a_at	Osi2	Osiris	down	75.94	2.03E-02	CG1148-RB	FBgn0037410	40756	726.70	9.57
1629362_at	mthl8	Mth-like 8	down	71.80	3.25E-03	CG32475-RA	FBgn0052475	38013	481.40	6.70
1627551_s_at	AttA /// AttB	attacin /// attacin	down	50.28	2.12E-04	CG18372-RA	0012042 /// FBgn004	36636 /// 36637	18401.06	365.98
1632719_at	CecC	Cecropin	down	48.93	4.82E-02	CG1373-RA	FBgn0000279	43599	2938.79	60.07
1634633_s_at	---	---	down	32.54	8.71E-04	Transposon.54	---	---	8123.52	249.62
1631805_at	CG3397	---	down	26.55	2.23E-04	CG3397-RA	FBgn0037975	41454	272.39	10.26
1626530_at	CecB	Cecropin B	down	19.09	2.92E-02	CG1878-RA	FBgn0000278	43598	2056.03	107.68
1641419_at	AttC	attacin	down	18.48	2.23E-02	CG4740-RA	FBgn0041579	36484	25310.48	1369.88
1625124_at	AttA	attacin	down	17.43	4.11E-03	CG10146-RA	FBgn0012042	36636	4509.97	258.81
1638687_at	CG40298	---	down	17.17	1.04E-03	CG40298-RA	FBgn0058298	3355149	419.97	24.46
1634310_at	TpnC4	TpnC4	down	16.51	2.00E-04	CG12408-RA	FBgn0033027	35498	222.00	13.45
1631962_at	MtnD	Metallothionein D	down	15.85	3.66E-02	CG33192-RA	FBgn0053192	326270	1834.55	115.71
1634551_at	CG6188	---	down	14.17	6.11E-03	CG6188-RA	FBgn0038074	41561	4443.01	313.61
1624393_at	w	white	down	13.39	1.29E-02	CG2759-RA	FBgn0003996	31271	272.84	20.38
1633545_at	PGRP-SD	PGRP-SD	down	12.93	1.88E-02	CG7496-RA	FBgn0035806	38858	4593.00	355.13
1631693_at	CG9616	---	down	12.26	2.08E-02	CG9616-RA	FBgn0038214	41731	641.09	52.30
1640955_s_at	---	---	down	12.25	5.63E-03	Transposon.34	---	---	93.25	7.61
1638235_at	DptB	diptericin-like protein	down	11.57	2.81E-02	CG10794-RA	FBgn0034407	37184	19133.68	1654.31
1641499_at	CG3259	---	down	10.79	3.69E-03	CG3259-RA	FBgn0038221	41739	674.65	62.54
1627759_at	CG30080	---	down	10.68	2.26E-03	CG30080-RA	FBgn0050080	246441	3079.46	288.41
1633812_at	CG9080	---	down	10.44	2.30E-02	CG9080-RA	FBgn0033593	36183	4862.15	465.86
1624819_s_at	---	---	down	10.06	8.00E-06	Transposon.17	---	---	2929.61	291.32
1627736_at	Actbeta	activin-beta	down	9.56	1.40E-04	CG11062-RA	FBgn0024913	43826	1000.20	104.66
1639402_a_at	scrib	Scribble	down	9.45	2.35E-03	CG5462-RC	FBgn0026178	44448	112.59	11.92
1638021_at	CG4757	---	down	9.24	2.11E-02	CG4757-RA	FBgn0027584	41330	8855.18	957.94
1629242_x_at	---	---	down	8.31	7.21E-03	Transposon.33	---	---	488.67	58.82
1635331_at	Cpr49Af	CG8510	down	8.14	5.94E-04	CG8510-RA	FBgn0033729	36352	136.10	16.73
1638087_at	CG33523	MSP protein	down	7.99	2.05E-03	CG32410-RA	FBgn0053523	38668	69.21	8.67
1636490_at	PGRP-SB1	PGRP-SB1	down	7.72	1.64E-02	CG9681-RA	FBgn0043578	39870	14076.17	1823.33
1627613_at	Mtk	Metchnikowin	down	7.38	1.33E-02	CG8175-RA	FBgn0014865	36708	31641.60	4284.80
1641338_at	fru	fruitless	down	7.04	2.36E-03	CG14307-RA	FBgn0004652	42226	54.80	7.78
1630633_at	CG13130	---	down	6.81	4.37E-03	CG13130-RA	FBgn0040965	50439	290.29	42.62
1630375_at	Mec2	Mec2	down	6.42	1.07E-02	CG7635-RA	FBgn0030993	32905	653.97	101.80
1622906_at	CG9027	---	down	6.30	2.48E-05	CG13200-RA	FBgn0033631	36232	697.80	110.83
1638428_at	---	---	down	6.16	3.19E-03	Transposon.33	---	---	101.35	16.47
1626147_s_at	---	---	down	6.13	4.90E-06	CG12891-RA	FBgn0027842	---	3991.61	651.59
1626695_at	CG9518	---	down	6.11	2.31E-03	CG9518-RA	FBgn0030590	32416	75.66	12.38
1628387_s_at	CG30080	---	down	5.94	7.29E-05	CT32157	FBgn0050080	246441	3041.75	512.33
1629072_at	CG42259 /// su(w[a])	--- /// suppressor of white-apricot	down	5.81	3.28E-02	CG3019-RA	0003638 /// FBgn025	31054 /// 7354475	2524.13	434.76
1633062_at	CG32791	---	down	5.69	2.31E-03	CG32791-RA	FBgn0052791	31322	245.51	43.15
1636330_at	CG17107	---	down	5.54	1.23E-02	CG17107-RA	FBgn0032281	34467	1368.48	246.85
1628806_at	Rh5	Rhodopsin5	down	5.53	2.73E-03	CG5279-RA	FBgn0014019	34615	3382.72	612.14
1628758_at	CG1774	---	down	5.50	1.41E-03	CG1774-RA	FBgn0039856	43722	633.57	115.16
1630749_at	CG11052	---	down	5.43	1.79E-04	CG11052-RA	FBgn0040524	49997	368.43	67.88
1634409_at	CG31775 /// CG42586	--- /// ---	down	5.38	1.88E-02	CG31775-RA	FBgn0028537	318935 /// 8673977	1522.99	282.92
1629269_at	CG32204	---	down	5.37	9.63E-04	CG32204-RA	FBgn0052204	317913	4551.97	848.11
1630804_at	Oatp33Eb	Organic anion transporting polype	down	5.36	2.24E-05	CG6417-RA	FBgn0032435	34662	118.50	22.10
1631871_at	ACXC	ACXC	down	5.33	2.59E-02	CG5983-RA	FBgn0040508	34689	44.56	8.37
1636946_at	CG6687	Dm-serpin-88Eb	down	5.32	1.86E-02	CG6687-RA	FBgn0038299	41829	3314.33	622.56
1635188_at	CG6675	---	down	5.23	3.64E-03	CG6675-RA	FBgn0032973	35439	3398.53	650.21
1625724_at	Pde1c	Phosphodiesterase 1c	down	5.22	1.07E-04	CG14940-RA	FBgn0259225	34594	1063.77	203.82
1639971_at	CG12224	---	down	5.18	5.61E-03	CG12224-RA	FBgn0037974	41453	55.51	10.71
1637061_at	flower	flower	down	5.00	2.43E-04	CG6151-RB	FBgn0036533	---	179.98	36.01
1627287_at	CG5966	---	down	4.91	1.71E-02	CG5966-RA	FBgn0029831	31532	2304.62	469.35
1632975_at	CG4398	---	down	4.89	1.00E-02	CG4398-RA	FBgn0034126	36838	122.98	25.17
1637652_a_at	CG30222	---	down	4.85	2.27E-03	CG30222-RD	FBgn0050222	50129	36.69	7.56
1628639_at	CG5791	---	down	4.85	1.59E-02	CG5791-RA	FBgn0040582	50055	17851.35	3683.38
1637275_a_at	CG13335	---	down	4.69	6.23E-04	CG13335-RA	FBgn0033857	36511	2539.76	541.24
1629125_at	gp210	gp210	down	4.67	2.31E-02	CT34163	FBgn0033039	35512	94.29	20.18
1636007_at	Ance-2	Ance-2	down	4.64	4.31E-03	CG16869-RA	FBgn0032535	34806	182.18	39.30
1627251_s_at	CG42343	CG12564	down	4.56	3.40E-03	CG12565-RA	FBgn0259245	33125	105.68	23.16
1640377_s_at	Rala	anon-fast-evolving-1H4	down	4.45	3.28E-04	CG2849-RB	FBgn0015286	31332	1318.17	296.16
1629853_at	CG3699	---	down	4.42	1.11E-02	CG3699-RA	FBgn0040349	31046	1775.75	401.42
1631697_at	---	---	down	4.41	4.88E-03	CG10816-RA	FBgn0010388	---	688.16	155.93
1629569_at	Fbxl4	CG1839	down	4.40	6.24E-03	CG1839-RA	FBgn0030555	32378	2477.79	562.85
1629671_at	CG14193	---	down	4.38	1.33E-02	CG14193-RA	FBgn0030994	32906	79.26	18.10
1623852_at	CG10407	---	down	4.35	1.28E-02	CG10407-RA	FBgn0038395	41949	1473.77	338.81
1630553_at	alphaPS5	alphaPS5	down	4.35	9.58E-03	CG5372-RA	FBgn0034880	37732	207.20	47.68
1634786_at	lectin-28C /// ninaE	Lectin28C /// rhodopsin	down	4.27	1.58E-02	CG7106-RA	FBgn0040099	42367 /// 53542	698.57	163.64
1626328_at	CG32259	---	down	4.18	9.14E-03	CG32259-RB	FBgn0052259	317942	81.47	19.48
1626629_s_at	---	---	down	4.14	2.32E-02	HDC02628	---	---	66.04	15.95
1637772_at	CG4726	---	down	4.13	5.84E-03	CG4726-RA	FBgn0031307	33292	294.96	71.39
1627958_at	CG30269	---	down	4.06	1.10E-03	CG30269-RA	FBgn0050269	37600	1364.41	335.88
1640085_at	CG9733	POAE-like Serine protease	down	4.05	4.37E-04	CG9733-RA	FBgn0039759	43601	381.90	94.22
1636148_at	CG6553	---	down	4.05	3.49E-05	CG6553-RA	FBgn0033880	36537	297.44	73.52
1633465_at	Cp7Fa	Chorion protein a at 7F	down	3.98	8.59E-03	CG15349-RA	FBgn0014464	3885650	55.12	13.86
1626503_at	CG2254 /// DsecGM11216	--- /// ---	down	3.94	8.32E-03	CG2254-RA	FBgn0029994	31726	4800.04	1219.79
1630913_at	Shroom	CG13942	down	3.90	6.35E-03	CT33491	FBgn0085408	36592	82.61	21.16
1639224_at	RPA2	CG9273	down	3.89	4.03E-03	CG9273-RA	FBgn0032906	35364	311.93	80.28
1631215_at	CG32132	---	down	3.88	6.73E-04	CG32132-RA	FBgn0052132	317877	677.54	174.45
1639730_at	CG34054	---	down	3.86	9.54E-04	HDC05792	FBgn0054054	3885601	564.21	146.23
1635936_at	CG13822	---	down	3.84	1.88E-02	CG13822-RA	FBgn0039098	42787	437.70	113.98
1633727_s_at	wnd	CG8789	down	3.80	1.27E-03	CG8789-RA	FBgn0036896	40143	1513.84	398.08
1639442_a_at	Tsf1	Transferrin 1	down	3.73	3.90E-02	CG6186-RB	FBgn0022355	32821	2100.72	563.39
1631165_at	CG4688	---	down	3.70	1.19E-05	CG4688-RA	FBgn0033817	36467	1743.56	470.92
1630934_at	---	---	down	3.69	3.54E-04	Transposon.30	---	---	3141.26	851.07
1640553_at	CG13063	---	down	3.66	6.46E-03	CG13063-RA	FBgn0036601	39797	290.56	79.29
1633220_at	CG16739	---	down	3.63	5.09E-03	CG16739-RA	FBgn0034505	37307	70.15	19.31
1630365_at	CG33330 /// CG3563	--- /// ---	down	3.63	1.94E-03	CG3563-RA	FBgn0038259	2768667 /// 41788	349.68	96.35
1634690_at	tum	tumbleweed	down	3.62	9.23E-04	CG13345-RA	FBgn0086356	36538	185.92	51.40
1641382_at	Pms2	Pms2	down	3.59	4.67E-03	CG8169-RA	FBgn0011660	36705	160.06	44.56
1623813_at	CG33307	---	down	3.55	2.00E-03	CG16887-RA	FBgn0053307	2768914	940.52	264.64
1635213_at	CG40470	---	down	3.55	4.22E-02	CG40470-RA	FBgn0058470	3355093	65.53	18.44
1632185_s_at	scpr-A /// scpr-B /// scpr-C	SCP-containing protein A /// SCP-(	down	3.54	1.63E-04	CG5207-RA	/// FBgn0037888 ///	41348 /// 41357 /// 41358	179.35	50.62
1639838_at	CG8483	---	down	3.54	4.23E-04	CG8483-RA	FBgn0038126	41623	6766.89	1913.09
1636213_at	---	---	down	3.53	3.17E-04	RE73276	---	---	160.20	45.43
1632392_s_at	Slob	giant slob	down	3.51	1.64E-04	CG6772-RB	FBgn0024290	34039	2654.48	756.38
1638330_at	Dot	Dorothy	down	3.50	1.88E-04	CG2788-RA	FBgn0015663	44058	271.16	77.47
1635189_at	Drs	drosomycin	down	3.49	1.49E-02	CG10810-RA	FBgn0010381	38419	21827.06	6250.09
1629194_s_at	ade2	adenosine2	down	3.49	4.07E-03	CG9127-RA	FBgn0000052	33847	1665.09	477.07
1624269_at	gkt	glaikit	down	3.49	7.49E-03	CG8825-RA	FBgn0031506	33530	268.68	76.99
1623364_at	CG4250	---	down	3.49	4.02E-02	CG4250-RA	FBgn0034761	37599	6065.82	1740.30
1627735_at	CG13908	---	down	3.47	3.99E-04	CG13908-RA	FBgn0035178	38110	1044.63	300.93
1635507_at	CG30031 /// CG4269	--- /// ---	down	3.44	1.78E-02	CG4269-RA	FBgn0034741	246404 /// 37578	1335.57	388.03
1628585_at	Gyc-89Da	Guanylyl cyclase at 89Da	down	3.43	3.83E-03	CG14885-RA	FBgn0038435	42001	64.50	18.83
1638424_at	CG10514	---	down	3.40	6.87E-03	CG10514-RA	FBgn0039312	43052	229.68	67.51
1628884_at	PGRP-SA	semmelweis	down	3.38	2.52E-02	CG11709-RA	FBgn0030310	32099	1921.02	568.22
1624849_at	Prosap	Prosap	down	3.36	1.39E-04	CG30483-RA	FBgn0040752	50225	2143.58	637.10
1632964_at	pirk	CG15678	down	3.35	3.47E-05	CG15678-RA	FBgn0034647	37468	454.21	135.42
1626248_at	CG18547	---	down	3.35	3.45E-03	CG18547-RA	FBgn0037973	41452	447.48	133.44
1625139_at	Gadd45	Gadd45	down	3.35	1.80E-02	CG11086-RA	FBgn0033153	35646	310.44	92.80

1628757_at	Glut1	glucose transporter 1	down	3.34	3.47E-04	CG1086-RA	FBgn0025593	38109	538.97	161.13
1624631_x_at	---	---	down	3.34	9.53E-03	Transposon.44	---	---	550.23	164.58
1634729_at	l(2)37Cd	CG10563	down	3.34	9.10E-03	CG10563-RA	FBgn0086445	35193	197.63	59.24
1631115_at	Obp8a	Odorant-binding protein 8a	down	3.30	1.39E-02	CG12665-RA	FBgn0030103	31860	336.06	101.73
1633921_at	CG30050	---	down	3.30	6.78E-03	CG30050-RA	FBgn0050050	246417	657.90	199.36
1640778_at	---	---	down	3.29	8.57E-04	LP09838	---	---	451.19	137.10
1640540_at	CG30273	---	down	3.26	4.20E-03	CG30273-RA	FBgn0050273	246520	409.84	125.54
1625067_at	CG32260	---	down	3.26	3.21E-02	CG32260-RA	FBgn0052260	317943	122.56	37.57
1641729_at	CG13043	---	down	3.22	8.13E-03	CG13043-RA	FBgn0036600	39796	4444.88	1378.97
1631468_a_at	tim	TIMELESS	down	3.20	1.21E-03	CG3234-RC	FBgn0014396	33571	139.12	43.41
1629013_at	CG11436	---	down	3.20	1.55E-03	CG11436-RA	FBgn0029713	31386	281.95	88.01
1639170_at	Cyp4d21	Cyp4d21	down	3.20	4.41E-02	CG6730-RA	FBgn0031925	34036	775.97	242.68
1633825_at	CG14221	---	down	3.19	1.49E-03	CG14221-RA	FBgn0031042	32961	174.84	54.77
1640775_a_at	Nmdmc	NAD-dependent methylenetetrahy	down	3.19	1.85E-03	CG18466-RB	FBgn0010222	47895	3590.03	1125.74
1641062_at	Ady43A	Ady43A	down	3.16	2.92E-04	CG1851-RA	FBgn0026602	44750	610.32	193.36
1628174_at	nimB1	CG33119	down	3.15	4.48E-04	CG33119-RA	FBgn0027929	34813	198.09	62.98
1627784_at	---	---	down	3.12	2.10E-03	GM07606	---	---	527.06	168.96
1626364_at	CG13000	---	down	3.10	1.10E-02	CG13000-RA	FBgn0030807	32685	47.10	15.21
1640029_s_at	CG14669	---	down	3.08	1.50E-02	HDC11911	FBgn0037326	40652	32.05	10.40
1635591_at	NijA	ninjurin A	down	3.08	3.72E-02	CG6449-RA	FBgn0036101	39215	2089.25	678.61
1630555_at	CG33346	---	down	3.08	9.60E-03	CG33346-RA	FBgn0053346	2768690	104.77	34.06
1638645_at	CG9662	---	down	3.04	3.53E-04	CG9662-RA	FBgn0031529	33554	348.34	114.73
1626492_s_at	Sop2	Suppressor of profilin 2	down	3.03	1.84E-03	CG8978-RB	FBgn0001961	34793	1574.72	518.88
1633796_at	CG11425	---	down	3.03	1.28E-04	CG11425-RA	FBgn0037167	40472	1776.72	586.94
1641657_s_at	CG12424	---	down	3.02	3.40E-03	CG12424-RC	FBgn0033987	36673	997.55	330.45
1628839_at	CG17110	---	down	3.02	1.91E-02	CG17110-RA	FBgn0039050	42729	65.31	21.65
1628015_at	cngl	CNG channel-like	down	3.02	4.38E-03	CG9176-RC	FBgn0029090	32468	247.03	81.93
1638611_at	---	---	down	3.00	7.34E-05	RE24931	---	---	177.94	59.35
1627271_at	CG13422 /// CG7655	--- /// ---	down	2.99	1.81E-02	CG13422-RA	FBgn0034511	37313 /// 42128	19741.97	6599.45
1639180_at	Arc1	CG12505	down	2.98	2.58E-02	CG12505-RA	FBgn0033926	36595	8545.51	2865.25
1640144_at	CG18067	---	down	2.96	1.42E-02	CG18067-RA	FBgn0034512	37314	31243.19	10546.43
1629784_a_at	Myo28B1	myosin VII 28B1-B4	down	2.96	4.90E-03	CG6976-RC	FBgn0040299	53515	254.15	85.99
1639697_at	---	---	down	2.94	1.77E-03	HDC10028	---	---	91.41	31.07
1626319_a_at	IM10	Immune induced molecule 10	down	2.94	4.03E-04	CG18279-RB	---	36486	2022.79	688.08
1635968_at	CG3604	---	down	2.93	2.25E-02	CG3604-RA	FBgn0031562	33593	421.05	143.61
1625074_at	CG12486	---	down	2.93	2.14E-02	CG12486-RA	FBgn0036541	39729	289.92	98.92
1630255_at	CG31676	---	down	2.92	7.79E-03	CG31676-RA	FBgn0051676	35351	1460.35	499.59
1626966_s_at	---	---	down	2.92	7.83E-03	Transposon.82	---	---	365.38	125.16
1630651_at	---	---	down	2.91	3.18E-02	S.C3R000941	---	---	55.42	19.01
1637662_at	msta	msta	down	2.89	3.11E-03	CG32800-RA	FBgn0053548	31200	4510.66	1560.14
1624688_at	CG9360 /// DsimCG9360	--- /// ---	down	2.87	4.04E-02	CG9360-RA	FBgn0030332	32128	1523.06	529.95
1626578_at	CG5195	---	down	2.87	8.67E-03	CG5195-RA	FBgn0036995	40266	144.82	50.43
1634256_a_at	CG11566 /// stg1	--- /// CG33670	down	2.87	3.22E-02	CG32514-RA	0031159 /// FBgn006	318064 /// 3772393	160.96	56.16
1634406_at	CG32982	---	down	2.85	7.23E-03	CG32982-RB	FBgn0052982	34237	384.64	135.12
1627191_a_at	ena	enabled	down	2.83	6.14E-03	CG15112-RC	FBgn0000578	37201	946.44	333.98
1624076_at	CG5849	---	down	2.83	2.64E-03	CG5849-RA	FBgn0038897	42556	190.35	67.33
1624953_at	Cyp12c1	Cyp12c1	down	2.82	2.03E-02	CG4120-RA	FBgn0036806	40037	651.54	230.94
1639374_s_at	Amy-d /// Amy-p	Amylase /// Amylase	down	2.81	1.30E-02	CG18730-RA	0000078 /// FBgn000	36932 /// 47764	1123.52	399.67
1636934_at	zormin	D-Titin	down	2.80	1.48E-03	CG32307-RA	FBgn0052311	2769001	418.65	149.73
1641619_at	CG34057	---	down	2.80	2.90E-02	CG13904-RA	FBgn0054057	3885645	127.52	45.62
1629753_at	Kr	Kruppel	down	2.80	1.51E-02	CG3340-RA	FBgn0001325	38012	55.99	20.03
1636071_a_at	Cbp80	cap binding protein 80	down	2.79	7.16E-05	CG7035-RA	FBgn0022942	44409	387.84	138.89
1635283_at	nimB2	CG31839	down	2.79	1.66E-02	CG31839-RA	FBgn0028543	260645	2347.77	841.51
1633419_s_at	CG7341	---	down	2.78	2.88E-02	CG7341-RA	FBgn0036777	40004	69.93	25.16
1627376_at	Rel	relish	down	2.78	9.03E-03	CG11992-RA	FBgn0014018	41087	4197.15	1511.38
1636409_at	CG11034	---	down	2.78	2.09E-02	CG11034-RA	FBgn0031741	33810	60.84	21.91
1629740_at	CG33828 /// His1:CG33831 /// His1:CG	His1:CG31617 /// His1:CG33801 /	down	2.78	3.67E-02	CG31617-RA	/// FBgn0053831 ///	71912 /// 3771981 /// 37719	780.35	281.09
1627489_a_at	CG10433	---	down	2.77	5.21E-03	CG10433-RA	FBgn0034638	37458	6730.30	2428.61
1636073_at	CG32344	---	down	2.75	6.28E-03	CG32344-RA	FBgn0052344	326208	201.12	73.15
1638581_at	Ubqn	CG14224	down	2.74	5.05E-05	CG14224-RA	FBgn0031057	32977	1420.56	518.00
1640199_at	Hcs	CG14670	down	2.74	1.30E-02	CG14670-RA	FBgn0037332	40659	945.94	345.78
1623218_s_at	Alh	Alhambra	down	2.72	7.37E-03	CG1070-RA	FBgn0037471	40850	171.57	63.02
1632478_a_at	Pkc53E	protein kinase C	down	2.69	5.71E-03	CG6622-RB	FBgn0003091	48311	2137.66	793.84
1623109_at	CG4593	---	down	2.69	3.76E-03	CG4593-RA	FBgn0029929	31649	250.37	92.99
1633936_a_at	sca	scabrous	down	2.68	1.59E-02	CG17579-RA	FBgn0003326	36411	208.58	77.91
1626061_at	CG34123	---	down	2.68	7.80E-03	CG30078-RA	FBgn0083959	36694	189.90	70.94
1639530_at	CG14528	---	down	2.68	1.83E-03	CG14528-RA	FBgn0039611	43410	400.06	149.48
1623951_at	---	---	down	2.67	3.74E-02	CG14068-RA	FBgn0053507	---	157.52	59.01
1630524_at	---	---	down	2.66	2.33E-02	LP09564	---	---	1171.71	440.32
1631534_at	sfl	diet-wingless	down	2.66	1.37E-03	CG8339-RA	FBgn0020251	38736	279.62	105.13
1628173_at	CG17189	---	down	2.66	6.58E-03	CG17189-RA	FBgn0039485	43263	1360.70	512.21
1637432_at	CG3097	---	down	2.64	2.18E-03	CG3097-RA	FBgn0029804	31503	753.62	285.52
1631635_at	CG7294	---	down	2.63	3.93E-02	CG7294-RA	FBgn0032284	34470	338.62	128.52
1623266_at	CG14180	---	down	2.63	3.04E-02	CG14180-RA	FBgn0036003	39101	233.47	88.66
1638636_at	CG12512	---	down	2.63	6.65E-03	CG12512-RA	FBgn0031703	33766	697.26	264.92
1638504_s_at	---	---	down	2.60	6.80E-04	CT38759	---	---	231.91	89.18
1630520_s_at	---	---	down	2.59	1.05E-02	RH09485	FBgn0062961	---	2686.54	1035.62
1635844_at	CG15887	---	down	2.59	2.68E-02	CG15887-RA	FBgn0038132	41631	154.63	59.74
1628942_at	CG15385	---	down	2.58	7.06E-03	CG15385-RA	FBgn0031397	33396	266.25	103.00
1630549_at	---	---	down	2.58	4.87E-02	HDC16354	---	---	45.29	17.52
1636293_at	CG2217	---	down	2.57	7.31E-05	CG2217-RA	FBgn0027544	43607	3450.46	1343.27
1632430_at	Tsf1	Transferrin 1	down	2.56	3.38E-02	CG6186-RA	FBgn0022355	32821	16254.26	6348.19
1628313_at	CG41452	---	down	2.56	4.72E-02	HDC11607	FBgn0084015	4379915	81.43	31.83
1630441_at	CG12716	---	down	2.55	8.16E-04	CG12716-RA	FBgn0030439	32248	83.99	32.93
1632761_at	beat-IIIa /// CG34106	beat-IIIa /// ---	down	2.54	8.81E-04	HDC02919	FBgn0083942	35035 /// 4379891	257.77	101.29
1627091_at	CG30287	---	down	2.54	2.75E-02	CG30287-RA	FBgn0050287	246530	44.13	17.39
1634438_at	Pph13	Munster	down	2.53	5.01E-04	CG2819-RA	FBgn0023489	33239	815.39	321.86
1628560_at	CG9344	---	down	2.53	3.95E-03	CG9344-RA	FBgn0034564	37372	366.38	144.87
1624836_at	CG13795 /// DsecCG13795	--- /// ---	down	2.53	4.12E-03	CG13795-RA	FBgn0031937	34049	1630.26	644.76
1638915_at	Ets21C	Ets at 21C	down	2.53	4.92E-02	CG2914-RA	FBgn0005660	33229	52.66	20.83
1631627_at	CG5335 /// Fbp1	--- /// Protein-1	down	2.53	6.63E-04	CG5335-RA	FBgn0034365	37140 /// 39566	366.13	144.88
1638452_at	per	period	down	2.53	4.60E-02	CG2647-RA	FBgn0003068	31251	490.64	194.20
1638696_a_at	CG3552	---	down	2.53	1.06E-02	CG3552-RA	FBgn0035999	39097	820.48	324.83
1631370_at	CG30026	---	down	2.51	4.43E-03	CG30026-RA	FBgn0050026	246399	1518.90	604.51
1639866_at	---	---	down	2.51	1.92E-02	S.C3L002717	---	---	60.63	24.14
1638314_at	CG34360	CG12802	down	2.49	7.71E-03	CG12418-RA	0040536 /// FBgn025	41217	624.23	250.64
1629398_at	CG10383	---	down	2.48	1.47E-03	CG10383-RA	FBgn0032699	35123	1278.97	515.55
1635273_s_at	kek5	kekkon-like	down	2.45	3.41E-03	CG12199-RC	FBgn0031016	32930	547.41	223.12
1641280_at	Ance-4	Ance-4	down	2.45	2.50E-02	CG8196-RA	FBgn0033366	35909	755.64	308.19
1637658_at	mthl3	mth-like 3	down	2.45	3.73E-02	CG6530-RA	FBgn0028956	36961	50.68	20.68
1624802_at	CG31686	---	down	2.45	4.93E-02	CG31686-RA	FBgn0051686	318885	70.04	28.64
1637462_at	CG13833	---	down	2.44	2.53E-02	CG13833-RA	FBgn0039040	42718	553.28	226.68
1640758_at	CG32613	---	down	2.44	3.81E-03	CG32613-RA	FBgn0052613	318117	254.92	104.56
1628296_at	pgant4	polypeptide GalNAc transferase 4	down	2.43	4.31E-02	CG31956-RA	FBgn0051956	261610	47.46	19.49
1625954_at	CG2681	---	down	2.42	2.99E-03	CG2681-RA	FBgn0024997	31258	354.81	146.35
1640757_at	CG33462	---	down	2.42	4.03E-02	CG30086-RA	FBgn0053462	2768841	93.99	38.86
1633145_at	PGRP-LF	PRGP-like	down	2.41	3.44E-03	CG4437-RA	FBgn0035977	39064	785.61	326.62
1629565_s_at	CG30160 /// Tsp42Eb	--- /// tetraspanin 42E	down	2.40	1.99E-02	CG30160-RA	0042086 /// FBgn005	246490 /// 59177	152.76	63.63
1639729_s_at	---	---	down	2.40	5.83E-03	Transposon.35	---	---	3621.75	1509.33
1623676_at	Doa	Darkener of apricot	down	2.39	1.81E-02	CG31049-RA	FBgn0259220	43415	98.00	41.08
1639223_a_at	sr	Stripe	down	2.39	4.53E-04	CG7847-RA	FBgn0003499	42162	303.64	127.30
1632204_at	Mmp1	Matrix metalloproteinase 1	down	2.38	2.27E-02	CG4859-RB	FBgn0035049	37949	1217.97	512.53
1636134_at	Or83b	Odorant receptor 83b	down	2.37	2.02E-02	CG10609-RA	FBgn0037324	40650	147.06	62.15
1624101_at	Cyp6a23	Cyp6a23	down	2.36	2.66E-03	CG10242-RA	FBgn0033978	36661	700.43	296.29
1636169_at	---	---	down	2.36	6.77E-03	HDC17695	---	---	68.95	29.19
1638393_at	---	---	down	2.36	1.18E-02	HDC12224	---	---	299.65	127.14
1640978_at	CG14567	---	down	2.35	3.05E-03	CG14567-RA	FBgn0037126	40420	1692.77	719.39
1625050_s_at	---	---	down	2.35	2.11E-02	Transposon.3	---	---	1607.60	685.02
1634188_a_at	tim	TIMELESS	down	2.34	1.14E-02	CG3234-RA	FBgn0014396	33571	4466.66	1908.52
1632961_at	Shaw	Shaker cognate w	down	2.34	1.15E-03	CG2822-RB	FBgn0003386	33599	197.23	84.28
1623871_at	CG18563	---	down	2.34	4.96E-02	CG18563-RA	FBgn0032639	35050	239.33	102.42
1640206_at	CG4660	---	down	2.33	4.60E-03	CG4660-RA	FBgn0029839	31542	1366.81	587.34
1629678_a_at	Aplip1	APP-like protein interacting protei	down	2.32	2.37E-02	CG1200-RA	FBgn0040281	53472	1264.59	545.99
1631225_at	CG8317	---	down	2.32	1.18E-02	CG8317-RA	FBgn0034140	36853	2474.28	1068.34
1625153_at	p24-2	p24-related-2	down	2.31	3.64E-02	CG33105-RA	FBgn0053105	318890	78.95	34.15
1626605_at	CG14075	---	down	2.31	7.90E-03	CG14075-RA	FBgn0036835	40071	113.64	49.23
1630825_at	CG5798	---	down	2.27	6.18E-03	CG5798-RA	FBgn0038862	42509	1861.32	820.27
1628265_a_at	CG42540	CG13719	down	2.26	2.40E-03	CG32245-RB	FBgn0052245	38562	890.15	393.41
1631714_a_at	SP71	SP71	down	2.26	1.44E-04	CG17131-RB	FBgn0029128	3354987	561.47	248.93
1625428_at	CG13707	---	down	2.25	6.33E-04	CG13707-RA	FBgn0035578	38583	447.04	198.30
1624540_at	CG14642	---	down	2.24	2.22E-02	CG14642-RB	FBgn0037222	40532	69.71	31.13
1627814_at	---	---	down	2.23	3.47E-02	CG14067-RA	FBgn0053507	---	51.71	23.16
1626665_at	CG7408	---	down	2.22	4.26E-02	CG7408-RB	FBgn0036765	39991	127.61	57.46
1625778_at	CG4562	---	down	2.22	1.67E-03	CG4562-RA	FBgn0038740	42362	211.80	95.46
1625756_at	CG6656	---	down	2.21	3.39E-04	CG6656-RA	FBgn0038912	42576	2855.44	1289.94
1634398_a_at	Dl	Delta	down	2.21	8.68E-03	CG3619-RB	FBgn0000463	42313	347.60	157.06
1634977_at	Tsp42Er	tetraspanin 42E	down	2.21	1.17E-03	CG12837-RA	FBgn0033139	35629	132.16	59.74
1637795_at	CG7778	---	down	2.21	1.14E-02	CG7778-RA	FBgn0032025	34147	1520.16	687.14
1636104_at	---	---	down	2.21	2.93E-02	HDC19315	---	---	164.24	74.38
1638238_at	CG5778	---	down	2.21	1.36E-02	CG5778-RA	FBgn0038930	42598	12178.13	5521.95
1634831_at	antdh	antdh	down	2.20	2.39E-02	CG1386-RA	FBgn0026268	32058	1059.99	481.08
1629749_a_at	CG13870	---	down	2.20	1.77E-02	CG13870-RB	FBgn0034506	37308	53.62	24.40
1629747_at	Cpr49Ag	CG8511	down	2.19	3.68E-03	CG8511-RA	FBgn0033730	36353	5599.61	2551.91
1625697_at	Cyp6g1	CYP6-like	down	2.17	2.73E-02	CG8453-RA	FBgn0025454	36316	2211.65	1019.30
1639819_at	CG6385	---	down	2.17	3.24E-02	CG6385-RA	FBgn0034276	37026	501.68	231.54
1631400_at	Cpr66D	CG32029	down	2.17	1.81E-02	CG32029-RA	FBgn0052029	38990	184.16	85.01
1630619_at	Spn1	serpin 1	down	2.16	3.29E-02	CG9456-RA	FBgn0028988	49808	1626.11	752.85
1625860_s_at	CG18542 /// CG32939	--- /// ---	down	2.16	1.79E-04	CG32939-RA	0037731 /// FBgn005	318260 /// 41176	1014.55	470.29
1628986_at	gp210	gp210	down	2.16	4.97E-03	CG7897-RA	FBgn0033039	35512	611.11	283.29
1626540_at	CG14077 /// Ir75d	--- /// CG14076	down	2.16	9.49E-03	CG14076-RA	0036829 /// FBgn003	40065 /// 40066	53.13	24.65
1623415_at	dl	Dorsal	down	2.15	1.78E-03	CG6667-RC	FBgn0000462	35047	735.75	341.61
1635558_s_at	CG17724 /// seq	--- /// sequoia	down	2.15	6.47E-03	LD12453	0028991 /// FBgn003	246682 /// 3772396	585.84	272.83
1627436_s_at	Pdp1	PAR-domain protein 1	down	2.14	5.50E-03	CG17888-RF	FBgn0016694	45588	3527.13	1650.91
1635113_at	CG34104	---	down	2.14	4.37E-02	CG12102-RA	FBgn0083940	31945	84.37	39.51
1630886_at	SelR	methionine-R-sulfoxide reductase	down	2.13	3.11E-03	CG6584-RD	FBgn0037847	41309	740.28	347.26
1641245_a_at	CG40115	---	down	2.13	4.36E-02	CG40115-RB	---	---	275.72	129.45
1632404_at	rtet	tetracycline resistance	down	2.13	2.41E-03	CG5760-RA	FBgn0028468	42500	270.59	127.34
1626888_at	CG9426	---	down	2.12	4.34E-03	CG9426-RA	FBgn0032485	34719	911.88	430.26
1625432_at	ths	thisbe	down	2.12	2.26E-02	CG12443-RA	FBgn0033652	36258	77.34	36.53
1623932_at	Ent3	Equilibrative nucleoside transporte	down	2.11	2.76E-03	CG11010-RA	FBgn0036319	39461	1021.71	483.21
1627669_at	CG1791	---	down	2.11	1.20E-03	CG1791-RA	FBgn0030163	31927	760.80	359.87
1623355_at	v	vermilion	down	2.11	1.15E-02	CG2155-RA	FBgn0003965	32026	1168.27	554.86
1633963_a_at	nAcRalpha-30D	Dalpha6	down	2.10	5.42E-04	CG4128-RA	FBgn0032151	34304	2659.01	1266.12
1638136_at	CG15765	---	down	2.10	2.13E-02	CG15765-RA	FBgn0029814	31513	345.93	164.74
1629899_at	cact	cactus	down	2.10	4.61E-02	CG5848-RC	FBgn0000250	34969	1694.25	807.30
1636998_at	sca	scabrous	down	2.09	9.73E-04	CG17579-RB	FBgn0003326	36411	351.65	168.41
1637963_at	14191 /// DmauCG14191 /// DsimCG14	--- /// --- /// ---	down	2.09	2.13E-03	CG14191-RA	FBgn0030981	32893	275.48	132.04
1633049_at	611159 /// DmirCG11159 /// DpseGA108	--- /// --- /// GA10804	down	2.08	3.53E-03	CG11159-RA	FBgn0034539	37341	240.85	115.62
1630036_at	CG13594	---	down	2.08	2.06E-02	CG13594-RA	FBgn0035041	37940	517.80	248.73
1636333_a_at	mthl2	Mth-like 2	down	2.08	4.55E-02	CG17795-RA	FBgn0035623	38636	165.51	79.76
1624020_at	mod(mdg4)	Modifier67.2	down	2.07	9.95E-03	CG32491-RO	FBgn0002781	49228	253.74	122.47
1633152_at	mod(mdg4)	Modifier67.2	down	2.06	3.00E-02	CG32491-RA	FBgn0002781	49228	87.30	42.29
1631772_a_at	MTA1-like	MTA-like	down	2.06	9.98E-04	CG2244-RA	FBgn0027951	40693	325.18	157.76
1627321_x_at	---	---	down	2.06	9.37E-03	CG6835-RA	FBgn0030882	---	72.86	35.37
1637997_at	CG32155	---	down	2.06	7.23E-03	CG32155-RA	FBgn0052155	317887	368.47	178.89
1641224_s_at	CG3812	---	down	2.06	8.78E-04	CG3812-RA	FBgn0030421	32230	81.14	39.41
1633237_at	Idgf1	Imaginal disc growth factor1	down	2.06	3.65E-02	CG4472-RA	FBgn0020416	34978	1499.64	729.58
1629771_at	Cyp12a5	Cyp12a5	down	2.05	1.73E-02	CG11821-RA	FBgn0038680	42293	296.93	144.66
1641398_s_at	LBR	Lamin B receptor	down	2.05	1.11E-02	CG17952-RB	FBgn0034657	37482	110.63	53.91

1632431_s_at	Ance	anon-fast-evolving-3D10	down	2.05	3.40E-03	CG8827-RA	FBgn0012037	34805	1906.44	929.09
1624108_at	CG34114	---	down	2.05	2.88E-02	CG4695-RA	FBgn0083950	4379854	62.38	30.41
1640947_at	CG15376	---	down	2.05	9.68E-03	CG15376-RA	FBgn0029692	31354	110.48	53.99
1639537_at	CG4587	---	down	2.05	1.53E-02	CG4587-RA	FBgn0028863	34921	145.74	71.26
1629469_s_at	CG10960	---	down	2.04	7.22E-03	CG10960-RC	FBgn0036316	39458	3956.29	1936.77
1638414_a_at	Bmcp	Bmcp	down	2.04	9.30E-03	CG7314-RA	FBgn0036199	39322	904.65	443.21
1636616_at	Or63a	Odorant receptor 63a	down	2.04	1.75E-02	CG9969-RA	FBgn0035382	38354	224.65	110.06
1623770_at	CG1673	---	down	2.04	5.95E-03	CG1673-RA	FBgn0030482	32297	2083.97	1021.13
1624595_a_at	Rbp2	anon-fast-evolving-1B9	down	2.04	2.79E-03	CG4429-RA	FBgn0010256	49809	340.40	166.85
1624911_at	Cyp28d2	Cyp28d2	down	2.04	1.88E-03	CG6081-RA	FBgn0031688	33748	200.23	98.25
1625563_s_at	CG14253	---	down	2.04	2.12E-03	CG14253-RB	FBgn0039467	43243	341.74	167.72
1638230_at	CG3160	---	down	2.04	2.62E-03	CG3160-RA	FBgn0029789	31487	369.57	181.38
1636061_at	---	---	down	2.03	5.02E-04	GM08821	---	---	2231.70	1096.94
1623875_at	CG3434	---	down	2.03	1.06E-03	CG3434-RA	FBgn0036000	39098	420.82	207.05
1629695_at	CG4686 /// DpseGA18356	--- /// GA18356	down	2.03	3.81E-02	CG4686-RA	FBgn0038739	42361	1310.28	645.29
1636682_at	CG31778	---	down	2.03	1.39E-02	CG31778-RA	FBgn0051778	261616	865.51	427.23
1635416_at	CG31100	---	down	2.03	9.25E-05	CG31100-RA	FBgn0051100	41075	921.80	455.17
1631783_at	CG4267	---	down	2.02	1.78E-02	CG4267-RA	FBgn0031405	33405	996.95	492.68
1624466_a_at	CG15117	---	down	2.01	6.25E-04	CG15117-RA	FBgn0034417	37197	366.78	182.07
1632884_at	CG33967	---	down	2.01	4.40E-04	CG12600-RA	FBgn0053967	41783	94.07	46.70
1628264_a_at	RecQ5	Drosophila homolog of RecQ	down	2.01	5.06E-03	CG4879-RA	FBgn0027375	39594	308.24	153.33
1640674_at	Ir25a	CG15627	down	2.01	4.13E-02	CG15627-RA	FBgn0031634	33683	104.72	52.19
1639019_s_at	CG33470 /// IM10	--- /// Immune induced molecule 10	down	2.00	1.14E-02	Dm.2R.12921.0	0033835 /// FBgn005	2768831 /// 36486	23187.79	11615.07
1623080_at	CG18410	---	down	2.00	1.47E-04	CG18410-RA	FBgn0039226	42953	911.33	456.69
1628572_s_at	stops	suppressor (rdgB) 69	down	1.99	1.07E-03	CG31006-RB	FBgn0086704	43683	2396.26	1203.33
1631315_at	CG12730	---	down	1.98	1.22E-03	CG12730-RA	FBgn0029771	31466	1971.47	995.90
1636762_a_at	scf	supercoiling factor	down	1.98	2.18E-02	CG9148-RA	FBgn0025682	38145	1472.40	744.48
1641322_at	Pde9	Phosphodiesterase 9	down	1.98	3.80E-02	CG32648-RA	FBgn0259171	32233	212.04	107.26
1625162_at	CG4611	---	down	1.97	3.49E-04	CG4611-RA	FBgn0035591	38601	275.78	139.85
1639136_s_at	psq	pipsqueak	down	1.97	8.95E-03	CG2368-RB	FBgn0004399	36118	446.89	226.81
1633254_at	CG7787	---	down	1.97	6.18E-03	CG7787-RA	FBgn0032020	34142	529.57	268.81
1641415_at	---	---	down	1.97	1.14E-02	RH47344	---	---	75.92	38.57
1639550_a_at	CG9650	---	down	1.97	5.68E-03	CG9650-RA	FBgn0029939	31660	234.61	119.32
1636107_at	CG14440	---	down	1.96	1.57E-02	CG14440-RA	FBgn0029894	31610	294.55	150.02
1626113_at	CG4080	---	down	1.96	1.64E-03	CG4080-RA	FBgn0035983	39079	898.39	457.59
1624543_s_at	---	---	down	1.96	1.16E-02	Transposon.32	---	---	2596.96	1323.28
1632100_s_at	sina	anon-fast-evolving-2H7	down	1.96	9.40E-03	CG9949-RA	FBgn0003410	39884	1069.66	546.16
1623194_at	CG7637	---	down	1.95	4.38E-02	CG7637-RA	FBgn0033548	36135	889.13	455.00
1627007_at	CG4306	---	down	1.95	1.90E-02	CG4306-RA	FBgn0036787	40017	695.01	356.22
1627055_at	CG14164 /// CG6709	--- /// ---	down	1.95	3.17E-02	CG6709-RA	0036056 /// FBgn003	39166 /// 39167	389.45	199.81
1635725_a_at	unc-115	unc-115	down	1.95	1.93E-03	CG31332-RC	FBgn0037733	41178	4339.11	2230.24
1628847_a_at	CG17167	---	down	1.95	2.85E-02	CG40146-RA	FBgn0039941	3354991	165.40	85.01
1632437_at	CR31846	---	down	1.94	2.82E-02	CG31846-RA	FBgn0051846	318979	594.90	306.16
1630609_s_at	CG2225	---	down	1.94	2.50E-02	CG2225-RB	FBgn0032957	35421	501.47	258.23
1639174_at	---	---	down	1.94	1.08E-02	CG32552-RA	---	---	77.60	40.00
1635284_a_at	HDAC4	HDAC4	down	1.94	2.10E-03	CG1770-RA	FBgn0041210	32278	1071.31	552.63
1622925_at	---	---	down	1.93	4.63E-03	CG15319-RB	FBgn0015624	---	580.84	300.24
1635575_at	---	---	down	1.93	8.67E-03	CG40032-RA	---	---	108.41	56.17
1635166_at	CG34400	CG17368	down	1.93	2.74E-02	CG32131-RA	FBgn0085429	39533	278.39	144.26
1640976_at	Ykt6	Ykt6	down	1.93	6.32E-04	CG1515-RA	FBgn0260858	31706	773.84	401.09
1630614_s_at	---	---	down	1.93	7.41E-04	HDC11981	---	---	3280.69	1703.75
1638683_at	---	---	down	1.92	1.32E-02	HDC17231	---	---	152.76	79.39
1641220_at	prom	prominin	down	1.92	9.85E-03	CG30165-RA	FBgn0259210	246493	214.92	111.75
1629467_at	pan	pangolin	down	1.92	1.91E-04	CG32005-RA	FBgn0085432	43769	354.51	184.35
1633218_a_at	UGP	UTP-G-1-P uridylyltransferase	down	1.92	1.12E-02	CG4347-RC	FBgn0035978	39065	265.56	138.63
1638078_at	Side	similar to Deadpan	down	1.91	7.73E-03	CG10446-RA	FBgn0032741	35168	725.20	379.37
1624440_at	---	---	down	1.91	3.44E-02	AM01482	---	---	389.26	203.73
1629051_at	---	---	down	1.91	4.32E-02	CG40396-RA	---	---	148.96	78.18
1623923_a_at	gl	glass	down	1.90	6.58E-03	CG7672-RB	FBgn0004618	42210	626.34	328.92
1629877_at	CG31683	---	down	1.90	4.79E-02	CG31683-RA	FBgn0051683	261623	407.91	214.32
1626345_at	IM4	Immune induced molecule 4	down	1.90	2.68E-03	CG15231-RA	FBgn0040653	50126	20282.52	10676.04
1623016_at	CG1299	---	down	1.90	5.00E-02	CG1299-RA	FBgn0035501	38496	1350.19	711.69
1633959_s_at	CG30345 /// CG9766 /// DsimCG9766	--- /// --- /// ---	down	1.90	7.98E-03	Transposon.10	FBgn0037229	246553 /// 40539	1880.90	991.83
1634669_at	CG32333	---	down	1.90	4.89E-02	CG32333-RA	FBgn0052333	38142	355.64	187.67
1633619_at	---	---	down	1.88	4.89E-02	GH12056	---	---	93.60	49.66
1638366_at	---	---	down	1.88	6.08E-04	HDC20114	---	---	225.57	119.77
1624378_at	east	enhanced adult sensory threshold	down	1.88	2.25E-03	CG4399-RB	FBgn0010110	46006	881.04	468.24
1623459_at	Spn4	neuroserpin	down	1.88	2.60E-04	CG9453-RC	FBgn0028985	49805	1061.65	564.45
1632997_at	CG34127	Drosokinin receptor	down	1.88	4.44E-02	CG32465-RB	FBgn0083963	40912	282.55	150.26
1638675_at	CG10680	---	down	1.88	1.06E-02	CG10680-RA	FBgn0032836	35282	6538.10	3479.55
1628076_at	Lip3	Lip3	down	1.88	1.21E-02	CG8823-RA	FBgn0023495	41643	45.73	24.35
AFFX-r2-Dros-Act5C-5_x_at	---	---	down	1.87	3.77E-02	AFFX-Dm-Act5C-5	---	---	1247.31	665.41
1634075_at	dp	dumpy	down	1.87	3.59E-03	CG33196-RB	FBgn0053196	318824	1297.83	693.11
1629703_at	npf	neuropeptide F	down	1.86	1.31E-02	CG10342-RA	FBgn0027109	42018	849.03	455.72
1630088_at	CG16743	---	down	1.85	2.33E-02	CG16743-RA	FBgn0032322	34517	1176.23	634.59
1627854_at	CG9914 /// DsecCG9914	Enoyl-CoA hydratase mitochondria	down	1.85	9.13E-03	CG9914-RA	FBgn0030737	32592	5585.41	3018.72
1640217_at	CG30154	---	down	1.85	2.69E-04	CG30154-RA	FBgn0050154	246487	1015.02	548.78
1627367_at	CG5653	---	down	1.85	3.21E-03	CG5653-RA	FBgn0035943	39024	526.78	284.99
1623327_at	---	---	down	1.85	4.05E-02	S.C2R001709	---	---	3637.07	1969.51
1638305_at	Mip	drostatin-B2	down	1.84	1.45E-02	CG6456-RA	FBgn0036713	39933	761.91	412.99
1627653_at	l(1)G0469	lethal (1) G0469	down	1.84	1.14E-02	CG32597-RA	FBgn0040153	32371	219.57	119.16
1633558_s_at	CG14997	---	down	1.84	8.89E-04	CG14997-RB	FBgn0035515	38514	787.18	427.74
1625022_a_at	CG6125	---	down	1.84	1.00E-04	CG6125-RB	FBgn0038337	41882	707.01	384.25
1639582_at	Pde11	Phosphodiesterase 11	down	1.84	1.22E-02	CG10231-RA	FBgn0085370	35107	1094.13	595.74
1624375_at	CG3227	insensitive	down	1.83	3.93E-02	CG3227-RA	FBgn0031434	33441	128.47	70.16
1632980_at	CG3902	---	down	1.83	3.53E-03	CG3902-RA	FBgn0036824	40059	3960.95	2168.92
1627279_at	nwk	nervous wreck	down	1.83	4.23E-02	CG4684-RA	FBgn0035966	39052	236.96	129.77
1631371_s_at	Pde8	Phosphodiesterase 8	down	1.82	6.45E-03	CG5411-RA	FBgn0034886	37741	238.14	130.62
1626789_at	CG13618	---	down	1.82	3.47E-02	CG13618-RA	FBgn0039203	42924	194.93	106.94
1636217_at	CG30503 /// kappaB-Ras	--- /// kappaB-Ras	down	1.82	1.90E-03	CG30503-RA	0040513 /// FBgn005	246656 /// 35667	1480.18	813.13
1638225_a_at	inx7	innexin7	down	1.81	3.86E-02	CG2977-RB	FBgn0027106	33027	86.90	48.00
1641570_s_at	CG18858 /// CG31683	--- /// ---	down	1.80	9.94E-05	CG31683-RA	0042175 /// FBgn005	261623 /// 326109	2643.23	1468.45
1625745_at	CG31643	---	down	1.80	1.70E-02	CG31643-RA	FBgn0051643	318867	174.93	97.35
1623265_at	CG10513	---	down	1.80	1.80E-02	CG10513-RA	FBgn0039311	43051	645.64	359.43
1625170_at	CG8001	---	down	1.79	1.13E-02	CG8001-RA	FBgn0035268	38224	381.32	212.46
1635367_at	fs(1)h	rancor	down	1.79	2.37E-02	CG2252-RA	FBgn0004656	31722	96.38	53.79
1636053_s_at	s-cup	stanley-cup	down	1.79	2.02E-02	CT32394	FBgn0050044	36371	65.28	36.48
1622949_at	peb	hindsight	down	1.79	2.33E-02	CG12212-RA	FBgn0003053	31391	639.53	357.44
1627150_at	CG3056	---	down	1.79	8.71E-04	CG3056-RA	FBgn0024987	31086	1404.75	785.19
1633017_at	Tsf3	Transferrin 3	down	1.78	3.78E-02	CG3666-RA	FBgn0034094	36800	531.44	298.08
1640746_at	CG18542 /// CG32939	--- /// ---	down	1.78	2.11E-03	CG18542-RA	0037731 /// FBgn005	318260 /// 41176	2437.52	1370.46
1625636_s_at	pan	pangolin	down	1.77	3.26E-02	CG17964-RE	FBgn0085432	43769	170.86	96.26
1623137_s_at	dpr8	dpr8	down	1.77	6.92E-03	CT36309	FBgn0052600	32387	217.62	122.68
1630042_at	Ca-alpha1T	Ca-alpha1T	down	1.77	1.40E-02	CG15899-RB	FBgn0029846	31550	129.21	72.85
1627752_s_at	SK	small conductance calcium-activat	down	1.77	6.03E-04	CG10706-RA	FBgn0029761	31456	1243.51	701.94
1632087_at	CG9928	---	down	1.77	1.02E-02	CG9928-RA	FBgn0032472	34704	10443.44	5896.76
1640276_a_at	Flo	flotillin-1	down	1.77	1.59E-02	CG8200-RA	FBgn0024754	36726	1922.45	1085.67
1636437_at	CG31638	---	down	1.77	6.10E-03	CG31638-RA	FBgn0051638	33910	173.56	98.07
1634235_at	CG34388	CG14842	down	1.77	3.42E-02	CG14843-RA	FBgn0085417	5740700	241.02	136.20
1636407_at	gol	goliath	down	1.77	7.98E-03	CG2679-RB	FBgn0004919	38006	3139.60	1776.02
1631382_at	CG13085	---	down	1.76	2.52E-02	CG13085-RA	FBgn0032780	35219	90.42	51.27
1639113_at	CG7705	---	down	1.76	1.64E-04	CG7705-RA	FBgn0038639	42243	584.61	331.65
1640898_at	DNaseII	acid DNase	down	1.76	2.31E-02	CG7780-RA	FBgn0000477	48228	1675.48	950.57
1632752_at	Ast	drostatin-A4	down	1.76	6.52E-03	CG13633-RA	FBgn0015591	42947	1028.70	584.31
1625325_s_at	simj	simjang	down	1.76	1.97E-02	CG32067-RB	FBgn0010762	39212	279.65	158.85
1628856_at	---	---	down	1.76	2.05E-02	CG31181-RA	---	---	377.46	214.54
1625652_s_at	CG12428	---	down	1.76	7.52E-03	CG12428-RB	FBgn0039543	43332	2074.17	1179.16
1630380_at	CG3837	---	down	1.76	1.12E-03	CG3837-RA	FBgn0038279	41809	686.69	390.49
1633534_s_at	mura	murashka	down	1.76	1.12E-02	CG9381-RA	FBgn0037705	41145	1074.22	611.00
1637516_at	CG15449	---	down	1.76	1.33E-02	CG15449-RA	FBgn0031146	33079	99.94	56.89
1624053_at	CG33967	---	down	1.76	3.40E-02	CG7552-RA	FBgn0053967	41783	281.83	160.51
1640395_a_at	dos	Suppressor of sevenless-S11	down	1.76	2.77E-02	CG1044-RA	FBgn0016794	38321	599.40	341.41
1633211_a_at	CG7795	---	down	1.75	4.75E-02	CG7795-RA	FBgn0032019	34141	59.80	34.09
1631127_at	CG10424	---	down	1.75	6.26E-03	CG10424-RA	FBgn0036848	40085	331.17	188.90
1631355_at	mRpS28	mitochondrial ribosomal protein S2	down	1.75	4.14E-04	CG5497-RA	FBgn0034361	37136	686.85	392.03
1623091_at	CG12644	---	down	1.75	1.34E-02	CG12644-RA	FBgn0040940	50414	117.16	66.89
1630844_at	CG15293	---	down	1.75	2.95E-02	CG15293-RA	FBgn0028526	34828	9699.15	5539.25
1625856_at	dm	diminutive	down	1.75	3.79E-02	CG10798-RA	FBgn0000472	31310	1567.71	897.35
1627571_at	CrebA	Box B-binding factor 2	down	1.75	4.23E-02	CG7450-RA	FBgn0004396	39682	505.37	289.40
1630248_at	CG5346	---	down	1.74	2.41E-02	CG5346-RA	FBgn0038981	42653	566.61	324.74
1625486_a_at	PGRP-LA	Peptidoglycan recognition protein	down	1.74	6.31E-03	CG32042-RB	FBgn0035975	39062	1498.93	859.37
1641448_a_at	Dg	Dystroglycan	down	1.74	3.32E-02	CG18250-RA	FBgn0034072	36773	601.68	345.24
1628101_s_at	CG32581 /// CG8974	--- /// ---	down	1.74	1.09E-04	CG32581-RA	0030693 /// FBgn005	318098 /// 32532	3457.98	1985.36
1640590_at	CG32195	---	down	1.74	4.94E-02	CG32195-RA	FBgn0052195	317907	272.15	156.57
1638205_s_at	futsch	Futsch	down	1.74	2.84E-04	GH26828	FBgn0259108	5740544	4457.55	2565.16
1639346_at	frj	Farjavit	down	1.74	3.94E-03	CG9526-RA	FBgn0031815	33900	1871.61	1077.05
1624161_at	---	---	down	1.73	3.18E-02	GH05702	---	---	435.04	250.94
1624497_at	---	---	down	1.73	3.46E-05	CR31541-RA	FBgn0037309	---	842.69	486.31
1626723_at	Takr86C	Neuropeptide receptor	down	1.73	2.64E-02	CG6515-RA	FBgn0004841	41286	125.55	72.52
1629745_at	CG6439	Isocitrate dehydrogenase	down	1.73	1.46E-03	CG6439-RA	FBgn0038922	42586	3547.65	2050.35
1631043_x_at	---	---	down	1.73	4.27E-02	HDC20230	---	---	463.16	267.99
1624241_at	NaCP60E	put. sodium channel gene	down	1.73	3.73E-02	CG18506-RA	FBgn0085434	37981	961.32	556.42
1623376_s_at	Nmda1	N-methyl-D-aspartate receptor-ass	down	1.73	1.29E-02	CG3798-RF	FBgn0013305	36419	5005.43	2897.88
1624523_at	CG32683	---	down	1.73	4.33E-03	CG32683-RA	FBgn0052683	31970	648.24	375.66
1629115_s_at	scrib	Scribble	down	1.73	2.69E-02	CG5462-RA	FBgn0026178	44448	329.44	190.93
1637130_at	CG30159	---	down	1.72	9.32E-04	CG30159-RA	FBgn0050159	35611	1309.19	760.02
1625219_at	ninaC	Droninac	down	1.72	4.51E-02	CG5125-RA	FBgn0002938	34012	210.91	122.48
1637853_a_at	Hk	Hyperkinetic	down	1.72	9.73E-04	CG32688-RB	FBgn0001203	31955	1708.69	992.79
1626946_at	oc	orthodenticle	down	1.72	4.17E-03	CG12154-RA	FBgn0004102	31802	294.06	170.87
1638603_at	CG31909	---	down	1.72	3.60E-02	CG31909-RA	FBgn0051909	319020	90.48	52.59
1637714_a_at	Ance-3	Ance-3	down	1.72	1.59E-03	CG31766-RA	FBgn0032536	34808	941.96	547.75
1627925_at	spict	CG12292	down	1.72	2.09E-03	CG12292-RA	FBgn0032451	34681	1498.35	871.31
1634186_a_at	CG17119	---	down	1.72	6.46E-03	CG17119-RA	FBgn0039045	42723	269.21	156.67
1632772_at	CG15203	---	down	1.72	1.77E-02	CG15203-RA	FBgn0030261	32038	344.63	200.58
1631149_s_at	La	ribonucleoprotein La	down	1.72	1.64E-02	CG10922-RB	FBgn0011638	35305	662.86	385.85
1634295_at	hoe2	hoepel2	down	1.72	5.37E-05	CG15624-RA	FBgn0031649	33700	1306.45	760.65
1634184_a_at	jp	junctophilin	down	1.71	4.18E-02	CG4405-RB	FBgn0032129	34274	501.92	293.30
1624189_at	CG12236	---	down	1.71	4.31E-03	CG12236-RB	FBgn0029822	31523	751.40	439.26
1627465_at	Mes4	NF-YC-like	down	1.71	2.66E-03	CG11301-RA	FBgn0034726	37560	481.05	281.30
1626574_at	CG9935	---	down	1.71	6.13E-03	CG9935-RA	FBgn0039916	43806	1739.71	1017.49
1641068_a_at	spz	Spaetzle	down	1.71	8.05E-03	CG6134-RA	FBgn0003495	43256	1041.65	610.05
1633487_at	CG30109	---	down	1.71	4.93E-03	CG30109-RA	FBgn0050109	246461	395.98	231.92
1623223_at	---	---	down	1.71	1.97E-02	GH25761	---	---	142.78	83.63
1635362_at	CG13654	---	down	1.71	3.98E-02	CG13654-RA	FBgn0039290	43028	231.46	135.60
1636059_at	CG9689	---	down	1.71	3.91E-03	CG9689-RA	FBgn0030159	31922	1461.15	856.51
1636953_a_at	---	---	down	1.71	3.48E-03	CG32171-RB	FBgn0036672	---	8394.75	4922.50
1639732_s_at	salr	sal-related	down	1.70	5.59E-03	CG4881-RA	FBgn0000287	34568	238.49	140.02
1625819_at	sno	Strawberry Notch	down	1.70	4.93E-03	CG1903-RB	FBgn0005410	32273	2191.48	1287.50
1626988_at	CG6805	---	down	1.70	9.77E-04	CG6805-RA	FBgn0034179	36911	1309.96	770.46
1626972_at	CG11874	---	down	1.70	5.75E-04	CG11874-RA	FBgn0039634	43436	1315.74	774.06
1628913_at	CG32365	---	down	1.70	2.24E-02	CG32365-RA	FBgn0052365	38876	180.09	106.21
1628709_at	CG34265	---	down	1.69	4.62E-03	HDC08233	FBgn0085294	5740186	404.97	239.00
1631722_at	---	---	down	1.69	1.67E-02	GM02743	---	---	452.99	267.51
1630127_at	---	---	down	1.69	1.50E-03	HL01468	---	---	272.08	160.78
1635488_at	CG31660	---	down	1.69	3.02E-02	CG31660-RB	FBgn0051660	33690	851.44	503.18
1629808_at	CG12004	anon-fast-evolving-3A8	down	1.69	3.46E-02	S.C3L000206	FBgn0035236	38185	332.79	196.81
1634436_at	Ast-C	drostatin-C	down	1.69	3.25E-03	CG14919-RA	FBgn0032336	34537	3132.88	1854.87
1625146_a_at	RN-tre	tre oncogene-related protein	down	1.69	6.85E-04	CG8085-RA	FBgn0020620	36554	154.75	91.62
1623899_at	Takr99D	Tachykinin-like receptor at 99D	down	1.69	2.47E-02	CG7887-RA	FBgn0004622	43551	200.51	118.97

1640335_at	CG42788	---	down	1.69	6.71E-04	CG31304-RA	FBgn0051304	---	635.21	376.96
1639489_at	CG3700 /// DmirCG3700 /// MED23	--- /// --- /// Mediator complex subu	down	1.68	5.63E-03	CG3700-RA	0034795 /// FBgn003	37639 /// 37640	691.96	411.17
1627837_at	CG5527	---	down	1.68	4.59E-02	CG5527-RA	FBgn0039564	43356	381.43	226.66
1640822_s_at	qkr54B	quaking related 54B	down	1.68	1.30E-02	CG4816-RA	FBgn0022987	36966	710.30	422.35
1624385_at	CG42574	CG17735	down	1.68	6.89E-04	CG17735-RA	---	40596	1545.55	919.64
1634856_at	Hdc	histidine decarboxylase	down	1.68	1.13E-03	CG3454-RA	FBgn0005619	36076	2954.38	1758.66
1635657_s_at	SK	small conductance calcium-activat	down	1.68	2.59E-02	CT36054	FBgn0029761	31456	114.28	68.05
1624556_s_at	bol	boule	down	1.68	3.18E-02	CG4760-RB	FBgn0011206	39049	320.26	190.90
1623517_at	G4962 /// DmauCG4962 /// DsimCG496	--- /// --- /// ---	down	1.68	2.00E-04	CG4962-RA	FBgn0036597	39793	3042.36	1814.87
1625624_at	CG14105	---	down	1.67	6.15E-05	CG14105-RA	FBgn0036359	39507	469.23	280.55
1631392_at	CG11089	---	down	1.67	3.42E-03	CG11089-RA	FBgn0039241	42973	8686.26	5193.86
1629898_s_at	CG10737	Gene 1	down	1.67	6.94E-04	CG10737-RD	FBgn0034420	37202	1183.07	707.97
1641156_at	dpr3	dpr3	down	1.67	1.92E-03	CG15379-RA	FBgn0053516	3346208	98.06	58.75
1623960_s_at	---	---	down	1.67	4.57E-02	Transposon.40	---	---	248.57	148.93
1637037_at	ade3	Complementation group D	down	1.67	8.42E-03	CG31628-RA	FBgn0000053	33986	1957.66	1173.62
1626965_at	CG31743 /// CG34313	--- /// ---	down	1.67	3.30E-04	CG31743-RA	0032618 /// FBgn008	326156 /// 5740766	2176.60	1305.38
1630514_at	Gprk1	rhodopsin kinase	down	1.67	4.04E-04	CG40129-RA	FBgn0004833	3355013	1618.35	971.78
1628659_at	Mdr49	Multi drug resistance 49	down	1.67	1.51E-02	CG3879-RA	FBgn0004512	36428	1276.78	766.77
1628160_a_at	dom	domino	down	1.66	9.91E-04	CG9696-RD	FBgn0020306	45655	612.08	367.72
1624377_s_at	be	E protein	down	1.66	1.63E-02	Transposon.84	FBgn0052594	32430	1133.69	681.11
1629590_at	Gr64c	Gustatory receptor 64c	down	1.66	1.73E-02	CG32256-RA	FBgn0045477	117479	75.79	45.54
1627828_s_at	CG34315 /// lat	--- /// Origin recognition complex s	down	1.66	4.36E-02	CG4088-RA	0005654 /// FBgn008	36454 /// 5740282	57.46	34.55
1629619_at	Aats-trp /// sage	Tryptophanyl-tRNA synthetase /// s	down	1.66	2.92E-02	CG9735-RA	FBgn0010803	41105 /// 45399	183.47	110.65
1641500_at	---	---	down	1.65	4.10E-02	Dm.X.56707.0	---	---	124.04	74.97
1641069_at	CG10703	---	down	1.65	2.70E-02	CG10703-RA	FBgn0037881	41350	303.42	183.84
1640667_at	Cyp4ae1	transcription unit I	down	1.65	4.71E-02	CG10755-RA	FBgn0015036	31193	212.75	129.00
1639525_at	king-tubby	Tubby-like	down	1.65	3.39E-02	CG9398-RA	FBgn0015721	37400	223.64	135.65
1627082_at	CG15465	---	down	1.65	1.08E-02	CG15465-RA	FBgn0029746	31434	727.73	441.44
1631964_at	CG18262	---	down	1.65	1.68E-02	CG18262-RA	FBgn0030012	31745	124.54	75.57
1641723_a_at	PGRP-LC	totem	down	1.65	1.23E-02	CG4432-RB	FBgn0035976	39063	792.23	480.82
1635858_s_at	Nhe2	Nhe2	down	1.65	1.77E-02	CG9256-RA	FBgn0040297	35376	82.12	49.84
1632958_a_at	CG42365	---	down	1.65	1.48E-02	CG15675-RA	FBgn0259711	37469	336.78	204.44
1623565_at	CG17278	---	down	1.64	3.54E-03	CG17278-RA	FBgn0046763	50050	1455.00	884.64
1625276_a_at	Eip71CD	methionine-S-sulfoxide reductase	down	1.64	3.10E-02	CG7266-RC	FBgn0000565	39675	1117.22	680.23
1630733_s_at	CG42322	CG15690	down	1.64	4.02E-02	CG31203-RA	FBgn0259222	42455	1145.78	697.87
1628496_at	dpr16	dpr16	down	1.64	2.56E-02	CG12591-RA	FBgn0037295	40619	179.45	109.37
1641284_a_at	CG6995	---	down	1.64	1.86E-02	CG6995-RA	FBgn0039229	42958	1002.19	611.09
1625666_at	---	---	down	1.64	1.78E-02	HDC10777	---	---	635.87	388.36
1633565_at	CG7741	---	down	1.64	1.97E-03	CG7741-RA	FBgn0033615	36208	282.01	172.25
1630696_at	CG12972	---	down	1.64	1.57E-03	CG12972-RA	FBgn0037076	40363	130.19	79.59
1626232_at	Camta	Calmodulin-binding transcription a	down	1.64	1.67E-02	CG8809-RA	FBgn0259234	35974	319.50	195.35
1638010_at	cngl	CNG channel-like	down	1.63	1.36E-02	CG9176-RB	FBgn0029090	32468	282.02	172.86
1624520_a_at	ftz-f1	Ftz interacting protein 1	down	1.63	4.63E-02	CG4059-RB	FBgn0001078	40045	394.85	242.03
1633181_at	osm-1	osm-1	down	1.63	4.45E-02	CG13809-RA	FBgn0035317	38282	73.20	44.89
1631364_at	CG33696	---	down	1.63	2.39E-02	CG32047-RA	FBgn0053696	39127	137.17	84.11
1629649_at	siz	schizo	down	1.63	9.99E-03	CT32172	FBgn0026179	40327	64.46	39.54
1636974_at	dlp	Dally-like	down	1.63	7.37E-04	CG32146-RA	FBgn0041604	39596	1138.89	698.72
1630309_s_at	---	---	down	1.63	4.15E-03	GM03661	---	---	790.43	485.03
1628688_at	CG12054	---	down	1.63	1.99E-02	CG12054-RA	FBgn0039831	43694	1023.97	628.47
1633036_s_at	CG32495 /// GS	--- /// Glutathione Synthetase	down	1.63	6.54E-04	CG32495-RA	0030882 /// FBgn005	318053 /// 32775	1506.39	924.84
1626812_at	CG14132	---	down	1.63	1.99E-02	CG14132-RA	FBgn0040817	50290	2361.89	1450.38
1633994_at	CG34402	CG17793	down	1.63	3.85E-02	CG4940-RA	FBgn0085431	41513	129.82	79.76
1624998_at	---	---	down	1.63	1.74E-02	HDC19722	---	---	97.84	60.15
1640924_a_at	CG3558	---	down	1.63	6.15E-03	CG3558-RA	FBgn0025681	48421	1465.54	900.96
1623209_at	CG8833	---	down	1.63	7.25E-03	CG8833-RA	FBgn0036386	39535	179.86	110.61
1637582_a_at	Nedd4	Nedd4	down	1.62	3.29E-02	CG7555-RC	FBgn0259174	39958	1325.79	815.93
1637206_at	CG8405	---	down	1.62	1.47E-03	CG8405-RA	FBgn0034071	36772	261.99	161.56
1628594_at	rut	rutabaga	down	1.62	2.61E-03	CG9533-RA	FBgn0003301	32406	330.02	203.65
1632003_a_at	Ptp99A	Protein tyrosine phosphatase 99A	down	1.62	7.10E-03	CG2005-RB	FBgn0004369	43469	152.59	94.19
1627632_at	CG17777	---	down	1.62	2.27E-03	CG17777-RA	FBgn0040900	50374	512.46	316.65
1625218_at	CG11399	---	down	1.62	3.68E-02	CG11399-RB	FBgn0037021	40295	471.85	291.75
1638368_at	CG31674	---	down	1.62	2.59E-02	CG31674-RA	FBgn0051674	318878	201.47	124.64
1630217_at	CG13650	---	down	1.62	9.30E-03	CG13650-RA	FBgn0039277	43014	1587.24	982.13
1634223_at	---	---	down	1.61	3.98E-02	S.C3R000885	---	---	44.38	27.50
1625613_at	CG11899 /// DsimGD21426	--- /// CG11899	down	1.61	2.38E-03	CG11899-RA	FBgn0014427	46391	2653.35	1644.55
1633293_at	CG10157	---	down	1.61	2.03E-02	CG10157-RA	FBgn0039099	42788	2537.40	1573.12
1630065_at	CG6912	---	down	1.61	1.04E-02	CG6912-RA	FBgn0038290	41820	675.95	419.16
1625620_a_at	CG14425 /// Sxl	--- /// sex-lethal	down	1.61	7.94E-03	CG33070-RC	0003659 /// FBgn002	3771983 /// 3772180	2117.78	1313.25
1639473_at	CG12541	---	down	1.61	1.52E-02	HDC17784	FBgn0029930	31650	223.27	138.48
1641362_at	CG13759	---	down	1.61	3.19E-02	CG13759-RA	FBgn0040376	31236	166.69	103.44
1636576_s_at	norpA	phospholipase C	down	1.61	5.65E-03	CG3620-RB	FBgn0004625	31376	13977.12	8677.25
1627300_at	para	paralytic	down	1.61	1.72E-02	CG9907-RA	FBgn0003036	32619	681.26	423.20
1624373_at	Crg-1	Circadianly Regulated Gene	down	1.61	4.54E-02	CG32788-RB	0021738 /// FBgn008	43900	183.16	113.85
1638927_at	Vti1	Vti1	down	1.61	2.53E-02	CG3279-RA	FBgn0035156	38085	875.82	544.76
1628009_at	fas	faint sausage	down	1.61	5.14E-03	CG17716-RA	FBgn0000633	36521	1037.26	645.18
1638751_a_at	CG4004	---	down	1.61	3.24E-02	CG4004-RB	FBgn0030418	32226	250.01	155.57
1636848_at	CG6024	---	down	1.61	3.66E-03	CG6024-RA	FBgn0036202	39325	1350.40	840.98
1638280_at	---	---	down	1.61	3.26E-02	CT22789	---	---	563.38	350.97
1632245_a_at	Spn4	neuroserpin	down	1.60	2.33E-02	CG9453-RB	FBgn0028985	49805	1469.44	916.07
1634131_s_at	Mnt	Mnt	down	1.60	7.44E-03	CG13316-RC	FBgn0023215	31331	946.51	590.26
1629423_at	ics	Ras suppressor-1	down	1.60	1.09E-03	CG9031-RA	FBgn0028546	34774	1471.53	918.51
1631704_at	CG5805	---	down	1.60	1.61E-03	CG5805-RA	FBgn0039223	42950	1325.65	827.82
1632409_a_at	Pka-C1	protein kinase A	down	1.60	1.50E-02	CG4379-RA	FBgn0000273	34284	373.29	233.11
1623036_a_at	CG10249	---	down	1.60	6.01E-03	CG10249-RC	FBgn0027596	36668	2416.99	1509.56
1641662_at	nAcRalpha-34E	Dalpha5	down	1.60	4.97E-02	CG32975-RA	FBgn0028875	34826	807.60	506.06
1625974_at	ird5	LPS-responsive kinase	down	1.59	9.10E-03	CG4201-RA	FBgn0024222	44432	403.42	252.94
1635713_at	CG13185	---	down	1.59	1.55E-02	CG13185-RA	FBgn0033661	36268	293.54	184.09
1635623_at	Cpr73D	CG9665	down	1.59	5.60E-04	CG9665-RA	FBgn0036680	39897	827.60	519.25
1634789_at	CG30151	---	down	1.59	3.60E-03	CG30151-RA	FBgn0050151	246484	531.76	334.00
1626339_at	garz	gartenzwerg	down	1.59	1.22E-02	CG8487-RB	FBgn0033714	36337	1102.41	692.58
1638476_s_at	mnb	minibrain	down	1.59	2.97E-02	LD34846	FBgn0259168	32771	374.74	235.45
1636742_at	CG13575 /// DmirCG13575 /// nord	--- /// --- /// nord	down	1.59	2.80E-02	CG30418-RA	0034996 /// FBgn005	37882 /// 37883	414.08	260.54
1636239_at	---	---	down	1.59	4.66E-02	HDC10913	---	---	53.54	33.72
1626187_at	Brf	Brf	down	1.59	1.25E-02	CG31256-RA	FBgn0038499	42087	373.75	235.48
1633011_at	crp	cropped	down	1.59	2.23E-02	CG7664-RA	FBgn0001994	34956	225.01	141.86
1638325_at	Hn	Henna	down	1.58	1.38E-02	CG7399-RA	FBgn0001208	38871	9221.14	5818.79
1638048_at	HdacX	CG31119	down	1.58	4.63E-02	CG31119-RA	FBgn0051119	326120	155.47	98.17
1641473_at	Fas3	Fasciclin III	down	1.58	3.85E-02	CG5803-RA	FBgn0000636	35097	120.89	76.37
1626805_s_at	gish	gilgamesh	down	1.58	7.01E-04	CG6963-RD	FBgn0250823	49701	2039.57	1288.86
1635130_a_at	SamDC	S-adenosylmethionine decarboxyl	down	1.58	7.16E-03	CG5029-RB	FBgn0019932	34396	729.39	460.96
1638114_at	CG13022	---	down	1.58	2.43E-02	CG13022-RA	FBgn0036679	39896	274.04	173.50
1631662_a_at	---	---	down	1.58	4.34E-05	CG18768-RC	FBgn0085445	---	2926.35	1855.58
1634318_at	CG33110	---	down	1.58	3.35E-02	CG33110-RA	FBgn0053110	42659	2102.15	1333.62
1628677_at	Pkcdelta	protein kinase C	down	1.58	7.42E-04	CG10524-RA	FBgn0259680	32191	476.66	302.49
1633775_at	CG31665	fibropellin	down	1.58	9.05E-03	CG31665-RA	FBgn0051665	33361	2281.85	1448.69
1631723_at	CG5498	transcript 2	down	1.58	3.18E-02	CG5498-RB	FBgn0027565	40250	663.72	421.41
1626897_at	HisCl1	Histamine-gated chloride channel	down	1.57	3.37E-02	CG14723-RA	FBgn0037950	41426	509.51	323.56
1624389_at	CG7526	---	down	1.57	3.39E-03	CG7526-RA	FBgn0035798	38850	84.81	53.91
1633798_at	yps	Ypsilon Schachtel	down	1.57	1.20E-02	CG5654-RA	FBgn0022959	39377	2276.52	1447.02
1632415_s_at	Dcp2	CG6169	down	1.57	3.28E-03	CG6169-RA	FBgn0036534	39722	309.46	196.71
1626518_at	seq	sequoia	down	1.57	4.64E-02	LD12453	FBgn0028991	3772396	271.34	172.48
1624913_s_at	oaf	transcript-near-decapentaplegic	down	1.57	1.27E-02	CG9884-RC	FBgn0011818	33435	265.35	168.71
1641071_at	CG8343	Incilarin-like	down	1.57	2.42E-02	CG8343-RA	FBgn0040502	35544	19914.77	12662.54
1636174_at	GstD9	Glutathione S transferase D9	down	1.57	1.27E-02	CG10091-RA	FBgn0038020	41502	772.86	491.50
1629347_at	klu	klumphuss	down	1.57	1.46E-02	CG12296-RA	FBgn0013469	39228	176.22	112.26
1626894_at	CG42700	---	down	1.57	4.14E-02	CG13165-RA	FBgn0033707	---	92.84	59.15
1641492_at	---	---	down	1.57	3.87E-03	GH12319	---	---	215.42	137.28
1629319_at	CG10428	---	down	1.57	1.03E-03	CG10428-RA	FBgn0032724	35150	787.69	502.45
1637491_s_at	CG3835	---	down	1.57	3.94E-05	CG3835-RB	FBgn0023507	31184	2277.29	1453.94
1640799_at	dsx	doublesex	down	1.57	3.26E-02	CG11094-RA	FBgn0000504	40940	385.29	246.04
1626659_at	CG4980	---	down	1.57	6.26E-03	CG4980-RA	FBgn0039558	43348	102.29	65.35
1624619_s_at	CG18477 /// CG31780	--- /// ---	down	1.56	3.37E-02	CG31780-RB	0028864 /// FBgn005	318937 /// 34922	340.86	217.86
1627233_at	CG14047	---	down	1.56	1.17E-02	CG14047-RA	FBgn0040390	31224	574.53	368.49
1633612_at	---	---	down	1.56	4.42E-02	CG8144-RB	FBgn0026188	---	309.63	198.90
1631055_at	Pka-C1	protein kinase A	down	1.56	2.50E-03	CG4379-RB	FBgn0000273	34284	1976.03	1269.58
1625012_s_at	vir-1	virus-induced RNA 1	down	1.56	8.91E-03	CG31764-RB	FBgn0043841	34652	3422.86	2199.52
1637174_s_at	CG13430 /// CG18065	--- /// ---	down	1.55	9.78E-04	CG33008-RA	0034518 /// FBgn003	3772419 /// 3772580	367.13	236.12
1640032_a_at	CG3814 /// TTLL3B	--- /// CG11201	down	1.55	3.56E-02	CG3814-RB	FBgn0025692	33946 /// 36418	156.62	100.73
1623171_s_at	lilli	lilliputian	down	1.55	1.04E-02	GM02019	FBgn0041111	33496	184.09	118.43
1632748_at	CG15084	---	down	1.55	2.32E-02	CG15084-RA	FBgn0034402	37178	139.02	89.46
1632213_s_at	CG31292 /// CG3303	--- /// ---	down	1.55	2.40E-02	CG31292-RA	0038381 /// FBgn005	318665 /// 41931	128.84	82.91
1623885_at	alpha-Est1	fragment A	down	1.55	1.53E-02	CG1031-RA	FBgn0015568	40909	1148.87	739.38
1638171_s_at	CG1753	---	down	1.55	2.99E-02	CG1753-RA	FBgn0031148	33081	568.41	366.02
1639968_at	Ciao1	Ciao1	down	1.55	4.17E-03	CG12797-RA	FBgn0033972	36655	1049.10	675.76
1636451_a_at	Atf-2	Activating transcription factor-2	down	1.55	7.49E-03	CG30420-RA	FBgn0050420	37978	1116.21	719.80
1626566_at	CG12262	---	down	1.55	3.96E-02	CG12262-RA	FBgn0035811	38864	4429.91	2856.92
1628080_at	GIIIspla2	phospholipase A[[2]]	down	1.55	2.91E-02	CG1583-RA	FBgn0030013	31747	2880.06	1857.47
1626099_s_at	fray	Ste20-like protein kinase	down	1.55	3.45E-02	CG7693-RA	FBgn0023083	44233	2033.28	1311.85
1639966_a_at	CG3262	---	down	1.55	1.70E-03	CG3262-RA	FBgn0032986	35452	1158.97	748.13
1639600_at	CG13919	---	down	1.55	1.42E-02	CG13919-RA	FBgn0035248	38200	427.59	276.10
1636276_at	Unr	Upstream of N-ras	down	1.55	1.35E-03	CG7015-RA	FBgn0035895	38963	662.73	428.13
1635633_s_at	bin3	bicoid-interacting protein 3	down	1.55	2.26E-04	CG8276-RB	FBgn0033073	35552	1341.70	867.11
1632753_a_at	Cep97	CG3980	down	1.55	1.64E-03	CG3980-RA	FBgn0031575	33610	737.58	477.10
1636759_at	CG8303	---	down	1.55	5.70E-03	CG8303-RA	FBgn0034143	36858	587.39	380.01
1625080_at	tral	trailer hitch	down	1.54	6.18E-04	CG10686-RA	FBgn0041775	39430	1934.65	1253.06
1641232_s_at	CG32706 /// CG6999	--- /// ---	down	1.54	9.71E-03	CG6999-RA	0030085 /// FBgn005	318160 /// 31837	675.28	437.75
1628243_at	dbr	debra	down	1.54	4.60E-02	CG11371-RB	FBgn0067779	33161	1191.70	772.55
1635236_at	CG42322	CG15690	down	1.54	1.01E-02	CG15690-RA	FBgn0259222	42455	264.61	171.64
1624699_s_at	Bx	Beadex	down	1.54	3.45E-02	CG6500-RA	FBgn0000242	32846	833.87	541.17
1629776_a_at	Esyt2	---	down	1.54	3.92E-03	CG6643-RA	FBgn0039208	42929	2503.22	1626.66
1636385_at	CG11317	---	down	1.54	5.85E-04	CG11317-RA	FBgn0039816	43676	820.20	533.07
1641434_at	CG32695	---	down	1.54	4.04E-02	CG32695-RA	FBgn0052695	31918	1562.71	1017.08
1633817_at	DJ-1alpha	DJ-1alpha	down	1.54	1.75E-02	CG6646-RA	FBgn0033885	36543	183.12	119.23
1626802_a_at	Sh	Shaker	down	1.54	8.80E-03	CG12348-RA	FBgn0003380	32780	4160.10	2709.07
1629473_at	cdi	center divider	down	1.54	1.91E-02	CG6027-RA	FBgn0004876	42289	1713.44	1116.21
1625707_s_at	Ccp84Aa /// Ccp84Ab	cuticle cluster 8 /// cuticle cluster 7	down	1.53	1.12E-02	CG2360-RA	0004782 /// FBgn000	40824 /// 40825	736.09	479.84
1639106_at	Grip	Glutamate receptor binding protei	down	1.53	4.84E-03	CG14447-RA	FBgn0029830	50391	353.27	230.46
1628331_at	Ca-beta	calcium channel beta subunit	down	1.53	4.96E-03	CG6320-RB	FBgn0259822	34557	641.15	418.74
1629681_x_at	CG4068	---	down	1.53	5.71E-04	CG4068-RA	FBgn0029738	31424	1219.14	796.42
1640163_at	Fas2	Fasciclin II	down	1.53	4.37E-04	CG3665-RA	FBgn0000635	31364	534.14	349.04
1625642_at	CG11509	---	down	1.53	7.58E-03	CG11509-RA	---	---	759.10	496.95
1633198_a_at	5-HT2	Serotonin receptor 2	down	1.53	7.57E-03	CG1056-RA	FBgn0087012	40575	1107.10	725.27
1631051_at	CG9323	---	down	1.53	1.55E-02	CG9323-RA	FBgn0032883	35339	221.91	145.38
1630756_at	Mipp2	Multiple inositol polyphosphate ph	down	1.53	1.51E-02	CG4317-RA	FBgn0026060	31544	1113.28	729.44
AFFX-r2-Dros-Act5C-5_at	---	---	down	1.53	1.28E-02	AFFX-Dm-Act5C-5	---	---	949.00	621.92
1639504_at	CG14855	---	down	1.53	2.84E-02	CG14855-RA	FBgn0038260	41789	108.49	71.14
1625245_at	CG3759	laccase-like	down	1.52	5.08E-03	CG3759-RA	FBgn0032116	34258	258.81	169.74
1627912_at	CG5493	---	down	1.52	3.94E-03	CG5493-RA	FBgn0034364	37139	1789.59	1174.10
1627354_at	CG11779 /// CG5835 /// DanaCG11779	--- /// --- /// ---	down	1.52	1.11E-03	CG5835-RA	0038682 /// FBgn003	42296 /// 42298	594.21	389.85
1623495_at	I-2	Inhibitor-2	down	1.52	5.56E-03	CG10574-RA	FBgn0028429	39156	1014.39	666.06
1638312_at	CG13189	---	down	1.52	1.84E-02	CG13189-RA	FBgn0033665	36273	312.41	205.39
1637749_at	D2R	Dopamine 2-like receptor	down	1.52	3.70E-02	CG17004-RB	FBgn0053517	33007	690.15	453.80
1624577_at	Ace	acetylcholine esterase	down	1.52	1.31E-02	CG17907-RA	FBgn0000024	41625	1769.87	1164.08
1632907_a_at	CG13204	---	down	1.52	2.51E-03	CG13204-RA	FBgn0033627	36227	448.69	295.42
1623069_s_at	CG17544	---	down	1.52	1.60E-02	CG17544-RA	FBgn0032775	35213	669.77	441.38
1631680_a_at	CG42367	---	down	1.52	9.33E-03	CG31878-RB	FBgn0259713	318998	200.03	131.98
1626218_s_at	oaf	transcript-near-decapentaplegic	down	1.51	1.44E-03	CG9884-RB	FBgn0011818	33435	841.32	555.41
1624103_at	CG7246	---	down	1.51	4.14E-02	CG7246-RA	FBgn0030081	31833	114.86	76.06
1638669_at	CG11395	---	down	1.51	1.76E-02	CG11395-RA	FBgn0034200	36937	3127.60	2072.41

1640366_at	CG5608	---	down	1.51	3.78E-02	CG5608-RA	FBgn0038058	41543	392.41	260.08
1629484_s_at	chinmo	Chronologically inappropriate mor	down	1.51	4.35E-02	CG31666-RD	FBgn0086758	33343	513.49	340.48
1638999_at	rno	rhinoceros	down	1.51	6.55E-03	CG7036-RA	FBgn0035106	38027	221.96	147.30
1637110_at	CG3618	---	down	1.51	2.00E-02	CG3618-RA	FBgn0037028	40303	1749.64	1161.27
1635360_at	CG10830	---	down	1.51	9.74E-03	CG10830-RA	FBgn0038839	42475	3063.63	2033.44
1633082_at	CG8449	---	down	1.51	7.49E-03	CG8449-RA	FBgn0038129	41628	655.13	434.88
1637636_at	DopEcR	DopEcR	down	1.51	3.92E-02	CG18314-RA	FBgn0035538	38539	1846.35	1226.13
1634106_at	CG11686	---	down	1.51	3.11E-02	CG11686-RA	FBgn0040551	50024	1096.65	728.62
1639892_at	Sodh-1	Sorbitol dehydrogenase like	down	1.50	4.89E-02	CG1982-RA	FBgn0024289	40836	2791.37	1855.23
1633498_at	CG7188	---	down	1.50	3.69E-02	CG7188-RB	FBgn0035871	38936	73.95	49.18
1634083_at	Nf1	neurofibromatosis type 1	down	1.50	1.54E-03	CG8318-RB	FBgn0015269	43149	722.02	480.14
1624405_a_at	CG7059	---	down	1.50	5.10E-03	CG7059-RA	FBgn0038957	42626	678.44	451.47
1623424_a_at	babo	Activin A receptor 45A	down	1.50	1.26E-02	CG8224-RB	FBgn0011300	35900	633.00	421.25
1637626_at	CG15412	---	down	1.50	1.94E-02	CG15412-RA	FBgn0031528	33553	214.97	143.09
1625158_at	Teh4	tipE homolog 4	down	1.50	2.55E-02	CG15003-RA	FBgn0035504	38501	148.92	99.15
1625873_at	CG15661	---	down	1.50	2.44E-02	CG15661-RA	FBgn0034605	37421	781.37	520.35
1635714_s_at	fdl	fused lobes	down	1.50	8.20E-03	CG8824-RC	FBgn0045063	250735	998.48	665.48
