# Supplementary material for: TDP-43 prevents retrotransposon activation in the Drosophila motor system through regulation of Dicer-2 activity
Source: BMC Biol. 2020 Jul 3;18:82. doi: 10.1186/s12915-020-00816-1 (PMC7334854; doi:10.1186/s12915-020-00816-1)
Supplement: Supplementary file 2 — Additional file 2 : Table S2. List of the 1408 Regulated Probesets (Fold-change ≥ 1,5; P-Value ≤ 0,05). [file 12915_2020_816_MOESM2_ESM.pdf]

Customer name:	Fabian Feiguin
Project name:	W1118 vs. Delta 142
Chip:	Affymetrix Drosophila Genome 2.0 Array
Organism:	Drosophilia
Analysis level:	Gene

Data Overview: Regulated probesets

	Up-regulated	Down-regulated	All
Number of regulated probesets	777	631	1408
Min fold-change	1.50	1.50	1.50
Average fold-change	3.12	5.51	4.19
Median of fold-change	1.97	2.00	1.98
Max fold-change	378.25	609.05	609.05
Gene with the max fold-change	CG10178	CG16713	CG16713

List of the 1408 Regulated Probesets (Fold-change ≥ 1,5; P-Value ≤ 0,05)

Affymetrix Probeset ID	Gene Symbol	Gene Name	Regulation	Fold-Change	P-Value	Representative Public ID	FlyBase ID	Entrez Gene ID (NCBI)	Intensity Delta 142	Intensity W1118
1628950_at	CG10178	---	up	378.25	1.96E-02	CG10178-RA	FBgn0032684	35105	3.92	1483.30
1628238_at	Tektin-C	TEKTIN C	up	54.05	4.22E-04	CG10541-RA	FBgn0035638	38653	21.84	1180.34
1628052_at	Cyp6a17	Cyp6a17	up	48.93	1.62E-04	CG10241-RA	FBgn0015714	45556	27.06	1323.83
1639594_at	CG40485	---	up	34.84	1.65E-02	CG40485-RB	FBgn0069973	3355165	132.01	4599.29
1639164_at	CG9486	---	up	25.87	1.46E-03	CG9486-RA	FBgn0031791	33874	12.67	327.77
1630728_a_at	phr	photolyase	up	16.76	2.00E-03	CG11205-RA	FBgn0003082	35735	41.27	691.90
1627096_s_at	CG16778	BTB-protein-III	up	16.58	4.56E-04	CG16778-RA	FBgn0003715	38007	26.24	434.95
1626774_s_at	robo	Roundabout 1	up	15.03	1.64E-03	CG13521-RA	FBgn0005631	37603	55.14	828.95
1638338_a_at	Fbp1	Protein-1	up	14.60	2.23E-02	CG17285-RA	FBgn0000639	39566	25.01	365.11
1635512_at	CG11893	---	up	13.22	4.98E-04	CG11893-RA	FBgn0039316	43056	62.31	823.82
1631639_at	TpnC47D	troponin C	up	12.76	4.73E-02	CG9073-RA	FBgn0010423	36195	141.67	1807.93
1640448_x_at	---	---	up	10.97	7.31E-05	Transposon.39	---	---	42.41	465.44
1632856_s_at	Vmat	Vesicular monoamine transporter	up	10.91	2.06E-04	CG6139-RB	FBgn0260964	3346192	8.61	93.89
1627324_at	lola	longitudinals lacking	up	8.99	1.83E-05	CG12052-RH	FBgn0005630	44548	40.47	363.89
1635312_at	---	---	up	8.73	5.91E-03	SD06617	---	---	11.80	103.01
1627841_at	CG11170	---	up	8.56	2.01E-05	CG11170-RB	FBgn0034705	37533	39.20	335.36
1635594_at	CG5255	---	up	8.32	7.57E-03	CG5255-RA	FBgn0038485	42073	5.69	47.30
1623410_at	Pxd	Peroxidase	up	7.70	1.21E-02	CT11695	FBgn0004577	2768671	407.26	3135.03
1631311_at	---	---	up	7.08	1.06E-03	CG14351-RA	---	---	12.91	91.45
1629479_a_at	fok	fledgling of Klp38B	up	6.99	2.65E-03	CG10746-RB	FBgn0020303	44380	1025.21	7164.52
1637590_at	CG42329	CG14343	up	6.67	7.76E-03	CG5156-RA	FBgn0259229	33312	37.22	248.33
1633780_at	CG13793	---	up	6.66	7.40E-03	CG13793-RA	FBgn0031935	34047	15.77	105.05
1623256_at	GstE1	glutathione-S-transferase	up	6.59	6.18E-05	CG5164-RA	FBgn0034335	37106	966.42	6365.75
1638869_at	Cpr51A	CG10112	up	6.52	7.55E-04	CG10112-RA	FBgn0033942	36613	105.78	690.04
1624864_s_at	dock	dreadlocks	up	6.26	1.28E-03	CG3727-RA	FBgn0010583	33262	186.86	1169.38
1630085_s_at	Peritrophin-A	peritrophin A	up	6.03	1.68E-03	CG17058-RA	FBgn0022770	33023	268.90	1621.16
1632045_at	CG5697	---	up	5.95	3.44E-02	CG5697-RA	FBgn0038846	42483	120.30	715.67
1635758_s_at	TBPH	TBPH	up	5.81	3.60E-04	CG10327-RD	FBgn0025790	37781	146.08	848.92
1633481_at	CG14394	Ninjurin C	up	5.59	3.07E-02	CG14394-RA	FBgn0038079	41568	234.08	1307.46
1634091_at	Cp19	Shell-19	up	5.50	3.65E-04	CG6524-RA	FBgn0000358	39000	56.43	310.28
1641317_at	squ	Squash	up	5.45	1.34E-03	HDC02844	FBgn0002652	2768920	16.20	88.30
1630927_x_at	Pxd	Peroxidase	up	5.44	3.10E-02	CT11695	FBgn0004577	2768671	327.57	1783.04
1623372_at	CG5873	---	up	5.31	1.81E-02	CG5873-RA	FBgn0038511	42100	140.91	748.53
1628617_at	dro4	drosomycin-F	up	5.07	2.11E-02	CG32282-RA	FBgn0052282	317954	2227.41	11286.86
1630010_a_at	pnt	pointed	up	5.03	6.21E-03	CG17077-RB	FBgn0003118	42757	28.28	142.30
1630458_at	CheA7a	CheA7a	up	5.02	2.75E-05	CG15033-RA	FBgn0029948	31672	229.51	1151.98
1638772_at	CG18107	---	up	4.94	5.70E-04	CG18107-RA	FBgn0034330	37101	3032.16	14978.95
1639225_at	CG13711	---	up	4.79	5.13E-03	CG13711-RA	FBgn0035572	38576	7.91	37.87
1623438_at	CG9505	---	up	4.73	3.42E-02	CG9505-RA	FBgn0031805	33889	27.30	129.13
1625884_at	CG13059	---	up	4.73	3.23E-02	CG13059-RA	FBgn0036607	39803	120.51	569.69
1640853_at	CG15020	---	up	4.72	1.31E-02	CG15020-RA	FBgn0035543	38544	14.23	67.16
1637562_at	TwdlT	GCR(ich)	up	4.72	1.45E-03	CG5812-RA	FBgn0029170	44790	200.45	945.57
1634343_at	Rpt6R	---	up	4.66	1.31E-02	CG2241-RA	FBgn0039788	43635	113.59	528.95
1639734_at	CG4017	---	up	4.64	2.71E-02	CG4017-RA	FBgn0032143	34296	22.40	104.03
1638856_at	CG2650	0.9kb transcript	up	4.62	4.49E-02	CG2650-RA	FBgn0000092	31252	1522.69	7028.32
1626735_at	CG13551	---	up	4.51	4.66E-02	CG13551-RC	FBgn0040660	50133	17.84	80.55
1637900_at	CG11852	---	up	4.51	3.12E-02	CG11852-RA	FBgn0039297	43035	1101.04	4963.60
1632527_at	CG13705	---	up	4.50	2.08E-03	CG13705-RA	FBgn0035582	38587	187.20	841.74
1625433_at	Scp1	calcium biding protein	up	4.47	2.04E-02	CG15848-RA	FBgn0020908	3355102	1051.02	4693.57
1630649_at	CG12520	---	up	4.40	2.06E-02	CG12520-RA	FBgn0036324	39466	17.65	77.68
1623834_at	CG18641	---	up	4.35	2.46E-03	CG18641-RA	FBgn0031426	33429	27.49	119.54
1637777_at	CG13982	---	up	4.28	1.48E-02	CG13982-RA	FBgn0031811	33895	16.03	68.65
1640681_at	CG4950	---	up	4.26	1.73E-02	CG4950-RA	FBgn0036587	39783	177.65	756.81
1635258_s_at	---	---	up	4.26	8.28E-03	Transposon.46	---	---	723.91	3083.20
1631432_at	CG42749	---	up	4.21	2.25E-02	CG15786-RA	FBgn0261803	---	68.42	288.00
1637123_a_at	CG33281	---	up	4.19	5.40E-03	CG15407-RB	FBgn0053281	2768938	37.51	157.23
1627801_at	CG9497	---	up	4.19	1.99E-04	CG9497-RA	FBgn0031800	33884	212.94	892.34
1631628_s_at	CG12896 /// Prx2540-1 /// Prx2540-2	AOP2-related /// peroxiredoxin 25	up	4.18	1.93E-02	CG11765-RA	/// FBgn0033520 ///	246663 /// 36098 /// 36101	3857.05	16123.93
1638475_a_at	CG16885	---	up	4.12	9.40E-03	CG16885-RB	FBgn0032538	34811	671.67	2769.32
1634209_at	pim	pimples	up	4.10	2.04E-02	CG5052-RA	FBgn0003087	34404	20.65	84.57
1627856_at	Hus1-like	Hus1-like	up	4.05	2.09E-02	CG2525-RA	FBgn0026417	40598	74.74	302.97
1640857_at	CG10208	---	up	4.05	1.36E-06	CG10208-RA	FBgn0039118	42814	314.04	1270.42
1626830_at	CG6044 /// Lcp1	--- /// cuticle p. I	up	4.04	9.30E-03	CG11650-RA	0002531 /// FBgn003	35817 /// 37558	42.55	171.71
1626891_at	CG15414	---	up	4.01	2.13E-02	CG15414-RA	FBgn0031542	33570	420.94	1689.59
1633097_at	CG14770 /// DsimCG14770	--- /// ---	up	4.00	3.25E-02	CG14770-RA	0029573 /// FBgn025	31085	241.51	965.27
1633120_at	---	---	up	3.96	1.52E-02	HDC06051	---	---	15.68	62.09
1624195_at	sug	sugarbabe	up	3.95	3.01E-02	CG3850-RA	FBgn0033782	36424	203.96	805.70
1638374_at	Obp19b	Odorant-binding protein 19b	up	3.94	3.28E-04	CG1670-RA	FBgn0031110	33038	224.98	887.33
1639098_s_at	CG2837	---	up	3.94	4.49E-04	CG2837-RB	FBgn0031646	33696	129.03	507.83
1639902_at	ventrally-expressed-protein-D	ventrally-expressed-protein-D	up	3.91	9.33E-05	CG33200-RA	FBgn0053200	117331	118.57	463.72
1637660_at	CG5150	---	up	3.91	4.76E-02	CG5150-RA	FBgn0035620	38633	11.49	44.88
1624982_s_at	CG5080	---	up	3.90	1.57E-02	CG5080-RB	FBgn0031313	33299	1474.00	5742.13
1627683_at	CG34296	---	up	3.89	2.74E-02	HDC16265	FBgn0085325	5740662	9.72	37.77
1630558_a_at	Rgk2	Rgk2	up	3.88	8.56E-03	CG15069-RA	FBgn0085419	37132	51.71	200.80
1632860_at	Cpr64Aa	CG15006	up	3.86	2.24E-04	CG15006-RA	FBgn0035510	38508	309.40	1195.25
1637366_at	nimC4	CG16876	up	3.85	1.47E-05	CG16876-RA	FBgn0260011	34824	163.39	629.80
1623522_at	CG11668	---	up	3.82	3.20E-02	CG11668-RA	FBgn0038113	41606	73.47	280.99
1624891_at	CG13183 /// DsecCG13183	--- /// ---	up	3.80	8.21E-05	CG13183-RA	0033667 /// FBgn026	36275	269.25	1023.40
1630171_at	Cpr97Ea	CG6131	up	3.75	2.59E-02	CG6131-RA	FBgn0039480	43258	1974.19	7409.45
1640900_at	Arc42	Arc42	up	3.71	1.03E-02	CG4703-RA	FBgn0038742	42364	124.03	460.63
1636015_s_at	CG32850	---	up	3.71	1.50E-02	Transposon.97	FBgn0052850	318246	2142.96	7951.61
1634123_at	CG14304	---	up	3.71	3.35E-02	CG14304-RA	FBgn0038629	42232	115.72	429.02
1635276_at	CG11437	---	up	3.70	1.06E-03	CG11437-RA	FBgn0037165	40470	47.87	177.05
1626882_at	lush	lush	up	3.70	2.62E-02	CG8807-RA	FBgn0020277	40136	125.80	464.89
1625061_at	CG17298	---	up	3.68	4.19E-02	CG17298-RA	FBgn0038879	42529	928.63	3416.24
1641125_at	CG1942	---	up	3.66	1.66E-03	CG1942-RA	FBgn0033215	35719	54.63	199.74
1624211_at	CG9005	---	up	3.64	5.04E-03	CG9005-RA	FBgn0033638	36243	843.97	3075.81
1632870_at	CG32364	---	up	3.61	3.71E-02	CG32364-RA	FBgn0052364	317996	66.35	239.20
1624602_at	CG13235	---	up	3.57	1.84E-02	CG13235-RA	FBgn0040766	50239	12.96	46.29
1635524_at	CG30427	---	up	3.57	1.15E-03	CG30427-RC	FBgn0043792	37986	288.51	1029.84
1624732_at	GstE5	Glutathione S transferase E5	up	3.55	1.72E-04	CG17527-RA	FBgn0063495	37110	110.90	393.93
1638240_s_at	CG8785	---	up	3.55	3.44E-02	CG8785-RA	FBgn0033760	36391	378.03	1341.35
1638554_at	CG4115	---	up	3.50	4.46E-02	CG4115-RA	FBgn0038017	41499	687.63	2404.62
1626513_at	MESR6	Misexpression suppressor of ras 6	up	3.49	3.99E-02	CG6818-RA	FBgn0036846	40083	496.30	1732.77
1627588_at	CG12483	---	up	3.47	8.06E-04	CG12483-RA	FBgn0040688	50161	175.10	608.02
1623276_at	CG31663	---	up	3.47	6.90E-03	CG31663-RA	FBgn0051663	33363	138.73	480.80
1640224_s_at	CG13796	---	up	3.46	2.30E-02	CG13796-RB	FBgn0031939	34051	571.12	1977.46
1628885_at	CG32698	---	up	3.43	5.12E-03	CG32698-RA	FBgn0052698	31915	50.93	174.82
1641567_at	lcs /// sesB	la costa /// lethal(1)9Ed	up	3.42	1.79E-02	CG12794-RA	0003360 /// FBgn002	32007 /// 33120	141.51	484.34
1624704_at	CG4741	---	up	3.42	1.57E-02	CG4741-RA	FBgn0035040	37939	66.67	228.20
1627927_at	CG8443	clueless	up	3.40	6.84E-03	CG8443-RA	FBgn0034087	36793	468.15	1590.88
1626933_s_at	CG2469	---	up	3.36	2.94E-03	CG2469-RB	FBgn0035205	38149	622.17	2093.35
1640035_at	Fbp2	fat body protein P6	up	3.36	8.55E-03	CG3763-RA	FBgn0000640	34259	42.89	144.13
1624830_at	Act79B	79B Actin	up	3.34	2.37E-02	CG7478-RA	FBgn0000045	40444	1207.11	4037.36
1635039_at	MED16	Mediator complex subunit 16	up	3.32	3.26E-02	CG5465-RA	FBgn0034707	37535	28.22	93.70
1624606_at	CG13062	---	up	3.32	1.98E-02	CG13062-RA	FBgn0036603	39799	67.59	224.34
1627793_s_at	CG9134	---	up	3.31	1.16E-02	CG9134-RB	FBgn0035199	38141	97.21	322.16
1640666_at	CG31205	---	up	3.27	3.24E-03	CG31205-RA	FBgn0051205	318626	822.87	2694.07
1632705_at	ninaD	neither inactivation nor afterpotent	up	3.26	8.83E-03	CG31783-RA	FBgn0002939	326160	144.62	471.84
1637914_at	Obp83g	Odorant-binding protein 83g	up	3.25	1.47E-02	CG31558-RA	FBgn0046875	170878	558.91	1817.22
1636676_at	CG2150	---	up	3.23	4.81E-03	CG2150-RA	FBgn0003065	43745	79.05	255.71
1631724_s_at	CG17746	---	up	3.21	2.24E-02	CG17746-RA	FBgn0035425	38400	2079.48	6678.71
1634351_at	CG7860	---	up	3.20	2.82E-02	CG7860-RA	FBgn0030653	32488	190.74	609.61
1628655_at	CG4476	---	up	3.18	1.22E-02	CG4476-RB	FBgn0035969	39056	48.97	155.72
1629475_at	nudC	nudC	up	3.18	1.26E-02	CG9710-RA	FBgn0021768	39879	585.49	1860.17
1638484_at	Hsp67Bc	Gene 3	up	3.14	4.10E-03	CG4190-RA	FBgn0001229	39071	414.68	1300.92
1623710_at	CG5847	---	up	3.14	1.01E-02	CG5847-RA	FBgn0036985	40255	78.29	245.48
1633540_at	CG8147	---	up	3.13	4.97E-04	CG8147-RA	FBgn0043791	41135	134.14	419.83
1635483_at	CG16884 /// DsimCG16884	--- /// ---	up	3.11	5.19E-04	CG16884-RA	0028544 /// FBgn008	34812	3967.32	12342.13
1625792_s_at	HipHop	lethal (3) neo26	up	3.11	2.52E-03	CG6874-RA	FBgn0036815	40047	114.05	354.28
1638327_a_at	CG17754	---	up	3.11	3.66E-02	CG17754-RC	FBgn0030114	31873	1325.48	4116.40
1636170_at	CG13607	---	up	3.10	5.12E-03	CG13607-RA	FBgn0039151	42859	186.37	576.89
1634418_at	CG33281	---	up	3.08	1.91E-02	CG15407-RB	FBgn0053281	2768938	53.19	163.80
1626198_at	Cyp4d8	Cytochrome P450-4d8	up	3.07	9.98E-04	CG4321-RA	FBgn0015033	38841	104.73	321.49
1624525_at	CG12998	---	up	3.07	4.07E-02	CG12998-RA	FBgn0030829	32710	1198.92	3676.19
1631302_at	CG16885	---	up	3.06	1.34E-03	CG16885-RA	FBgn0032538	34811	5418.18	16600.88
1638054_at	CG13056	---	up	3.05	2.61E-03	CG13056-RA	FBgn0040794	50267	1276.96	3895.14
1635131_at	CG4525	---	up	3.04	4.51E-03	CG4525-RA	FBgn0038358	41905	33.18	100.90
1641464_s_at	CG32850	---	up	3.04	3.70E-04	CG32850-RA	FBgn0052850	318246	2053.78	6240.05
1637097_at	CG7497	---	up	3.03	2.37E-02	CG7497-RA	FBgn0036742	39966	428.57	1299.10
1633882_at	Rgk1	Rgk1	up	3.02	4.41E-02	CG9811-RA	FBgn0034434	37219	444.60	1343.44
1636811_at	Oatp33Ea	Organic anion transporting polype	up	3.02	2.07E-02	CG5427-RA	FBgn0032433	34660	295.60	891.94
1638221_at	Obp83cd	Odorant-binding protein 83d	up	3.00	1.93E-03	CG15582-RA	FBgn0046878	40746	67.44	202.58
1628318_at	Spc105R	CG11451	up	3.00	1.21E-02	CG11451-RA	FBgn0037025	40300	23.07	69.20
1626727_at	Mct1	Monocarboxylate transporter 1	up	2.99	1.20E-02	CG3456-RA	FBgn0023549	31198	139.87	417.91
1641242_at	Ugt86Di	Ugt86Di	up	2.95	2.11E-03	CG6658-RA	FBgn0040251	53502	54.25	159.94
1629822_a_at	CG15506	---	up	2.95	1.03E-02	CG15506-RA	FBgn0039686	43498	176.75	520.87
1625915_at	CG4375	---	up	2.94	4.49E-02	CG4375-RA	FBgn0031295	33278	17.92	52.70
1625389_at	CG8209	---	up	2.93	9.40E-03	CG8209-RA	FBgn0035830	38888	286.78	839.17
1640870_s_at	CG17322	---	up	2.92	7.49E-05	CG17322-RD	FBgn0027070	35139	145.93	426.62
1626353_at	CG15738	---	up	2.92	1.50E-03	CG15738-RA	FBgn0030352	32151	303.07	885.43
1636915_at	CG11380	---	up	2.91	1.70E-03	CG11380-RA	FBgn0040359	31065	129.29	376.59
1627185_at	---	---	up	2.90	3.41E-02	HDC07868	---	---	26.56	77.07
1625312_at	CG10553	---	up	2.90	2.32E-04	CG10553-RA	FBgn0039324	43064	844.36	2446.90
1639440_at	CG13285	---	up	2.88	2.49E-02	CG13285-RA	FBgn0035611	38623	117.53	338.30
1640770_a_at	Eip78C	nuclear hormone receptor	up	2.87	2.39E-02	CG18023-RA	FBgn0004865	40345	121.14	347.16
1626392_s_at	Mef2	Complementation group C	up	2.85	9.17E-03	Transposon.2	FBgn0011656	36032	2620.42	7478.33
1640684_at	CG6980	---	up	2.84	1.25E-02	CG6980-RA	FBgn0039228	42955	32.63	92.84
1636046_at	Cda4	CG32499	up	2.83	1.06E-02	CG32499-RA	FBgn0052499	33144	551.51	1561.12
1625314_at	CG2022	---	up	2.82	3.52E-04	CG2022-RA	FBgn0037292	40615	313.04	883.29
1626028_at	CG4783	---	up	2.82	4.53E-02	CG4783-RA	FBgn0038756	42381	16.62	46.80
1628660_at	CG7130	---	up	2.81	2.95E-02	CG7130-RA	FBgn0037151	40450	157.62	443.13
1639130_a_at	kkv	Chitin Synthase B	up	2.80	1.25E-02	CG2666-RA	FBgn0001311	45884	1287.11	3607.80
1638033_at	CG6055	---	up	2.80	1.01E-03	CG6055-RA	FBgn0031918	34027	48.11	134.68
1628062_at	CG34048	---	up	2.79	2.89E-04	S.C2L001366	FBgn0054048	3885615	12.21	34.09
1628915_s_at	---	---	up	2.76	1.83E-02	CG3799-RB	---	---	994.74	2742.51
1624971_at	CG5254	---	up	2.74	3.66E-02	CG5254-RA	FBgn0040383	31020	3339.99	9161.11
1640833_at	CG42237	phospholipase A2	up	2.74	6.43E-03	CG4346-RA	FBgn0250862	32261	171.23	469.02
1629845_at	CG34375	CG13834	up	2.73	1.88E-02	CG13835-RA	FBgn0085404	42715	43.75	119.37
1634062_at	CG14218	---	up	2.72	4.27E-02	CG14218-RA	FBgn0031031	32949	27.45	74.66
1635371_at	CG31323	---	up	2.72	2.16E-02	CG31323-RA	FBgn0051323	318682	37.86	102.98
1630632_s_at	CG2982	---	up	2.72	3.67E-02	CG2982-RB	FBgn0029704	31374	447.79	1216.20

1635161_at	---	---	up	2.71	2.88E-02	CG7532-RA	---	---	3295.81	8934.70
1638768_at	Cpr11A	CG2560	up	2.71	8.03E-03	CG2560-RA	FBgn0030394	32198	107.59	291.20
1632568_at	CG3223	---	up	2.70	2.44E-02	CG3223-RA	FBgn0037538	40947	757.10	2046.55
1637293_at	CG17707	---	up	2.69	1.19E-02	CG17707-RA	FBgn0025835	5740408	236.05	635.80
1637246_a_at	CG32627	---	up	2.69	6.45E-03	CG32627-RA	FBgn0052627	32329	1796.51	4837.30
1641740_at	CG34370	CG13499	up	2.69	1.47E-03	CG30408-RA	FBgn0085399	5740565	95.32	256.29
1641365_s_at	---	---	up	2.68	5.12E-06	SD27140	---	---	1388.47	3721.83
1641270_at	CG8745	---	up	2.67	3.35E-03	CG8745-RA	FBgn0036381	39530	1229.76	3287.02
1637939_at	Spn43Aa	Serine protease inhibitor 1	up	2.67	1.84E-02	CG12172-RA	FBgn0024294	45041	24.95	66.54
1624398_at	CG8630	---	up	2.66	6.82E-03	CG8630-RA	FBgn0038130	41629	1688.81	4497.24
1639400_at	CG31207	---	up	2.65	5.41E-03	CG31207-RA	FBgn0051207	326125	1110.76	2947.00
1625908_a_at	TppII	tripeptidyl peptidase II	up	2.65	2.59E-02	CG3991-RD	FBgn0020370	36444	2460.58	6522.92
1639907_at	CG11951	---	up	2.64	2.68E-02	CG11951-RA	FBgn0039656	43463	183.67	484.76
1634519_at	CG34217	---	up	2.64	6.21E-03	HDC05165	FBgn0085246	5740254	1523.37	4017.15
1623811_at	mRpL18	mitochondrial ribosomal protein L1	up	2.64	3.50E-04	CG12373-RA	FBgn0026741	36406	274.12	722.76
1630577_at	CG17378	---	up	2.63	6.98E-03	CG17378-RA	FBgn0031858	33951	30.02	78.90
1633089_a_at	lola	longitudinals lacking	up	2.63	6.99E-05	CG12052-RJ	FBgn0005630	44548	47.69	125.26
1629676_at	Tbp-1	Proteasome 26S regulatory subun	up	2.62	4.61E-02	CG10370-RA	FBgn0028684	42805	3054.97	7997.98
1628531_at	Rpn11	yippee interacting protein 5	up	2.62	3.55E-02	CG18174-RA	FBgn0028694	33738	3785.87	9905.27
1629530_at	IM23	Immune induced molecule 23	up	2.62	1.51E-02	CG15066-RA	FBgn0034328	37099	7354.76	19240.46
1634820_at	Pros29	Proteasome 20S alpha3 subunit	up	2.61	3.78E-02	CG9327-RA	FBgn0261394	37378	2357.82	6163.44
1637531_at	CG1440 /// DsimGD16898	--- /// ---	up	2.61	4.61E-02	CG1440-RA	0030038 /// FBgn008	31776	1104.14	2880.82
1636338_at	CG15021	---	up	2.61	4.58E-03	CG15021-RA	FBgn0035544	38546	977.17	2548.21
1626184_at	CG16978	---	up	2.59	8.29E-04	CG16978-RA	FBgn0040972	50446	287.25	744.81
1629803_a_at	scb	volado	up	2.59	8.70E-04	CG8095-RB	FBgn0003328	36692	490.91	1270.31
1640107_at	obst-A	CG17052	up	2.58	6.48E-05	CG17052-RA	FBgn0031097	33022	1325.39	3420.22
1626481_a_at	Taz	tafazzins	up	2.58	8.60E-03	CG8766-RA	FBgn0026619	36405	1746.27	4500.62
1628696_at	CG12643	---	up	2.57	3.12E-03	CG12643-RA	FBgn0040942	50416	1769.36	4545.16
1629266_s_at	CG13349	---	up	2.57	4.68E-02	CG13349-RA	FBgn0033886	36545	2339.05	6005.57
1631050_at	CG5384	---	up	2.57	4.87E-02	CG5384-RA	FBgn0032216	34387	3940.02	10110.94
1629641_s_at	---	---	up	2.55	1.53E-02	Transposon.65	---	---	86.63	221.23
1627872_at	CG3770	---	up	2.54	7.51E-03	CG3770-RA	FBgn0035085	37991	137.94	350.44
1626660_at	CG15024 /// Pdh	--- /// pigment cell dehydrogenase	up	2.53	7.30E-03	CG15024-RA	0011693 /// FBgn004	39812 /// 50172	298.13	755.56
1628843_at	Ipod	Interaction partner of Dnmt2	up	2.53	1.49E-03	CG2961-RA	FBgn0030187	31954	44.69	113.23
1635208_at	CG9934	---	up	2.53	3.42E-02	CG9934-RA	FBgn0032467	34699	2156.23	5461.66
1637349_at	Rpn1	Rpn1	up	2.53	2.35E-02	CG7762-RA	FBgn0028695	40174	1807.34	4573.34
1641389_at	ckd	cracked	up	2.53	7.93E-04	CG14959-RA	FBgn0035427	38402	163.64	414.00
1639217_s_at	brat	Brain Tumor	up	2.52	4.46E-02	CG10719-RA	FBgn0010300	35197	59.19	148.91
1633592_a_at	CREG	cellular repressor of E1A-stimulate	up	2.51	8.90E-03	CG5413-RB	FBgn0025456	42092	1941.57	4869.33
1639812_at	CG5555	---	up	2.51	2.34E-02	CG5555-RA	FBgn0038686	42302	730.91	1831.82
1640389_at	Obp56c	Odorant-binding protein 56c	up	2.50	2.24E-02	CG30128-RA	FBgn0046879	170882	23.25	58.21
1638472_at	Cpr62Bb	CG13935	up	2.50	2.25E-02	CG13935-RA	FBgn0035280	38240	2240.39	5591.84
1625190_at	CG31105	---	up	2.49	4.84E-02	CG31105-RA	FBgn0051105	318593	34.74	86.64
1635259_at	Mov34	Mov34	up	2.49	3.56E-02	CG3416-RA	FBgn0002787	37894	2331.35	5800.95
1624448_at	CG6356	---	up	2.48	6.23E-03	CG6356-RA	FBgn0039178	42893	363.41	902.84
1631249_at	CG12713	---	up	2.48	4.28E-02	CG12713-RA	FBgn0036536	39724	47.66	118.17
1629638_at	conv	convoluted	up	2.48	1.78E-02	CG8561-RA	FBgn0261269	36588	207.60	514.68
1624035_a_at	verm	helmsman	up	2.48	1.75E-02	CG8756-RB	FBgn0261341	40149	1705.40	4222.15
1623000_at	CG33093	---	up	2.46	7.97E-03	CG33093-RA	FBgn0053093	42654	83.61	205.82
1628261_at	CG13067	---	up	2.46	1.14E-02	CG13067-RA	FBgn0036589	39785	1739.11	4280.55
1633594_at	Rpn7	Rpn7	up	2.46	3.80E-02	CG5378-RA	FBgn0028688	42641	2101.19	5162.18
1628458_at	milt	milton	up	2.45	2.27E-03	CG13777-RC	FBgn0022201	45683	949.05	2329.64
1633690_at	CG8323	---	up	2.45	7.10E-03	CG8323-RA	FBgn0033903	36566	287.01	704.43
1636853_at	Cpr47Ef	CG13214	up	2.44	2.13E-02	CG13214-RA	FBgn0033603	36194	336.13	820.84
1628584_at	Cyp305a1	Cyp305a1	up	2.44	1.07E-03	CG8733-RA	FBgn0036910	40161	363.09	886.07
1637077_s_at	CG3777	---	up	2.44	4.22E-03	CG3777-RB	FBgn0024989	30979	782.90	1908.84
1640397_at	CG34360	CG12802	up	2.43	8.21E-03	CG32469-RA	FBgn0250818	41217	73.16	177.84
1637421_at	Cht2	Chitinase 2	up	2.43	1.63E-02	CG2054-RA	FBgn0022702	38223	444.38	1079.89
1632328_at	serp	Serpentine	up	2.43	2.31E-02	CG32209-RB	FBgn0260653	40150	3021.51	7327.76
1639636_at	Cht7	CG1869	up	2.42	4.61E-03	CG1869-RA	FBgn0035398	38370	1029.42	2493.32
1624011_at	Cpr76Bc	CG9295	up	2.42	3.10E-02	CG9295-RB	FBgn0036880	40122	72.80	176.31
1635449_s_at	Dip-B	Dipeptidase B	up	2.42	9.99E-03	CG9285-RA	FBgn0000454	48450	1649.83	3992.11
1623773_at	CG32082	---	up	2.42	3.97E-03	CG32082-RA	FBgn0052082	39258	83.43	201.73
1638616_at	mthl9	Mth-like 9	up	2.42	2.26E-02	CG17084-RA	FBgn0035131	38056	91.26	220.59
1640126_at	CG8249	---	up	2.41	1.11E-02	CG8249-RA	FBgn0034045	36742	147.56	356.31
1632278_s_at	---	---	up	2.41	2.88E-02	CG30193-RA	---	---	208.46	503.33
1633718_at	CG5355	---	up	2.41	7.93E-03	CG5355-RA	FBgn0032242	34421	669.66	1616.72
1626588_a_at	CG5325	Peroxisomal farnesylated protein	up	2.41	3.42E-03	CG5325-RB	FBgn0032407	34630	415.26	999.82
1640462_at	Ho	Heme oxygenase	up	2.40	1.46E-02	CG14716-RA	FBgn0037933	41407	5879.04	14137.44
1633939_at	CG13563	---	up	2.40	3.67E-03	CG13563-RA	FBgn0034966	37845	148.27	356.18
1629731_at	CG14565	---	up	2.39	3.17E-02	CG14565-RA	FBgn0037129	40423	70.39	168.11
1632683_s_at	copiaGIP	gag-int-pol	up	2.38	3.18E-05	Transposon.11	FBgn0013437	---	12011.23	28605.91
1639460_a_at	CG17218	---	up	2.38	3.26E-03	CG17218-RA	FBgn0032421	34646	505.08	1201.72
AFFX-r2-Dros-GAPDH-M_a_at	---	---	up	2.37	7.55E-04	AFFX-Dm-Gpdh-M	---	---	4628.95	10971.69
1627855_at	yellow-e	yellow-e	up	2.37	7.19E-05	CG9792-RA	FBgn0041711	41653	908.23	2149.11
1634226_at	CG17249	---	up	2.36	5.79E-03	CG17249-RA	FBgn0035249	38201	63.86	150.70
1638227_at	uif	SP1070	up	2.36	8.36E-04	CG9138-RA	FBgn0031879	33983	216.47	510.30
1628712_at	CG17754	---	up	2.35	2.90E-04	CG17754-RC	FBgn0030114	31873	3317.59	7807.36
1637161_s_at	myoglianin	Myoglianin	up	2.35	6.11E-03	CG1838-RA	FBgn0026199	43811	41.21	96.90
1641055_at	Hsp23	Protein 23	up	2.35	1.79E-02	CG4463-RA	FBgn0001224	39077	870.35	2045.35
1625584_at	CG8654	---	up	2.35	3.08E-03	CG8654-RA	FBgn0034479	37275	1093.02	2566.03
1635057_s_at	CG15445	---	up	2.34	1.48E-02	CG15445-RB	FBgn0031161	33094	1290.09	3024.99
1630289_at	CG6435	---	up	2.34	9.81E-03	CG6435-RA	FBgn0034165	36894	500.06	1170.65
1635573_at	---	---	up	2.34	1.58E-02	CG12488-RA	---	---	65.20	152.40
1639934_at	CG31272	---	up	2.34	1.35E-03	CG31272-RA	FBgn0051272	326130	36.24	84.66
1637490_at	CG13314	---	up	2.33	1.50E-02	CG13314-RA	FBgn0035949	39030	110.28	257.39
1633887_at	13857 /// DmauCG13857 /// DsimCG13	--- /// --- /// ---	up	2.33	2.45E-02	CG13857-RA	/// FBgn0260196 ///	42627	46.81	109.17
1638610_at	CG7802	---	up	2.33	3.44E-02	CG7802-RA	FBgn0039704	43523	48.93	114.10
1630333_at	Cpr50Ca	CG13338	up	2.33	1.01E-02	CG13338-RA	FBgn0033867	36523	27.22	63.38
1630072_a_at	CG10096 /// CG10097	--- /// ---	up	2.33	4.04E-02	CG32919-RA	0038032 /// FBgn003	3771756 /// 3772183	35.06	81.62
1640358_at	CG4968	---	up	2.33	1.81E-02	CG4968-RA	FBgn0032214	34384	928.62	2160.71
1634019_at	CG2064	---	up	2.33	1.36E-03	CG2064-RA	FBgn0033205	35708	219.97	511.77
1636311_at	Gpdh	5' gene	up	2.32	1.74E-02	CG9042-RA	FBgn0001128	33824	5533.04	12863.46
1637341_at	Rpt3	Rpt3	up	2.32	4.21E-02	CG16916-RA	FBgn0028686	32047	3025.43	7024.33
1626326_at	CG31436	---	up	2.32	2.69E-03	CG31436-RA	FBgn0051436	318734	384.43	892.48
1626488_s_at	Rpn9	Nobody	up	2.32	3.14E-02	CG10230-RB	FBgn0028691	42802	4086.43	9479.37
1635636_at	to	Takeout	up	2.32	2.47E-02	CG11853-RA	FBgn0039298	43036	201.76	467.81
1638884_at	CG4238	---	up	2.31	1.29E-02	CG4238-RA	FBgn0031384	33377	84.57	195.67
1623103_at	CG14946	---	up	2.31	2.14E-02	CG14946-RA	FBgn0032405	34627	407.28	940.57
1628555_at	Osi19	Osiris	up	2.31	2.42E-02	CG15189-RA	FBgn0037429	40776	51.11	118.03
1636131_at	lig3	DNA ligase III	up	2.31	4.55E-04	CG17227-RA	FBgn0038035	41518	306.80	707.98
1633329_at	yellow-h	yellow-h	up	2.31	2.91E-02	CG1629-RA	FBgn0039896	43779	648.76	1496.10
1623740_at	CG32154	---	up	2.30	3.55E-03	CG32154-RA	FBgn0052154	39778	88.69	203.92
1628398_at	CG5853	---	up	2.30	2.64E-03	CG5853-RA	FBgn0032167	34322	739.72	1700.52
1640616_at	CG17974	---	up	2.30	1.21E-02	CG17974-RA	FBgn0034624	37441	155.28	356.68
1636572_at	Duox	Dual oxidase	up	2.29	1.86E-03	CG3131-RA	FBgn0031464	33477	342.56	784.78
1629597_a_at	Asph	Aspartyl beta-hydroxylase	up	2.29	4.55E-02	CG8421-RB	FBgn0034075	36778	4670.13	10695.06
1623524_at	CG7031	---	up	2.29	1.07E-02	CG7031-RA	FBgn0039027	42704	56.54	129.28
1635793_at	CG11413	---	up	2.28	4.17E-02	CG11413-RA	FBgn0035022	37920	7940.48	18134.35
1639452_at	CG14984	---	up	2.28	2.45E-02	CG14984-RA	FBgn0035480	38467	882.10	2012.55
1628087_s_at	CG10200	---	up	2.28	3.09E-02	CG10200-RB	FBgn0033968	36648	1183.22	2697.28
1636888_a_at	Aph-4	Alkaline phosphatase	up	2.28	2.64E-03	CG1462-RA	FBgn0016123	43671	1073.35	2445.55
1635321_at	CG14688	---	up	2.28	2.71E-02	CG14688-RA	FBgn0037819	41275	1430.55	3257.46
1638852_at	CHKov2	CHKov2	up	2.28	1.86E-02	CG10675-RA	FBgn0039328	43068	265.06	603.46
1631735_at	CG10445	---	up	2.28	5.24E-03	CG10445-RA	FBgn0037531	40938	24.67	56.14
1636313_at	CG4914	---	up	2.27	3.30E-02	CG4914-RA	FBgn0036436	39597	141.08	320.91
1640991_at	CG32091	---	up	2.27	7.96E-03	CG32091-RB	FBgn0052091	39307	434.79	988.58
1636706_at	Myd88	krapfen	up	2.27	3.36E-02	CG2078-RA	FBgn0033402	35956	275.16	623.80
1634795_a_at	Prosalpha7	20s proteasome	up	2.26	3.17E-02	CG1519-RB	FBgn0023175	36018	2701.59	6112.36
1634580_at	bsk	Jun kinase	up	2.26	2.85E-02	CG5680-RC	FBgn0000229	44801	29.07	65.68
1629923_at	CG7371	---	up	2.26	5.52E-04	CG7371-RA	FBgn0031710	33774	194.69	439.65
1630968_at	CG13907	---	up	2.26	1.31E-02	CG13907-RA	FBgn0035173	38104	1736.31	3919.51
1631728_s_at	PRL-1	PRL-1	up	2.25	1.29E-02	CG4993-RB	FBgn0024734	34952	1591.75	3585.46
1626086_at	CG10621	selenocysteine methyltransferase	up	2.25	6.76E-03	CG10621-RA	FBgn0032726	35152	746.98	1680.61
1627994_at	CG33296	---	up	2.25	3.38E-02	CG33296-RA	FBgn0053296	2768941	79.07	177.75
1632155_at	CG13676	---	up	2.25	1.96E-04	CG13676-RA	FBgn0035844	38906	530.57	1192.76
1638896_at	Cpr62Bc	CG1919	up	2.24	5.74E-04	CG1919-RA	FBgn0035281	38241	5211.07	11698.15
1636208_a_at	CG15097	---	up	2.24	2.32E-02	CG15097-RB	FBgn0034396	37172	276.95	619.77
1636668_at	spz5	CG9972	up	2.23	1.97E-02	CG9972-RA	FBgn0035379	38350	395.70	884.08
1624273_at	CG8034	---	up	2.23	4.54E-02	CG8034-RA	FBgn0031011	32924	247.39	551.93
1641707_at	Ufd1-like	UfD1-like	up	2.22	4.06E-02	CG6233-RA	FBgn0036136	39254	1295.10	2881.30
1639091_at	Rpn5	Rpn5	up	2.22	3.35E-02	CG1100-RA	FBgn0028690	40717	3855.29	8554.27
1624745_at	Ilp5	Drosophila insulin-like peptide 5	up	2.21	3.28E-03	HDC09365	FBgn0044048	2768992	546.17	1208.44
1624423_at	CG5945	---	up	2.21	2.69E-03	CG5945-RA	FBgn0032494	34729	6668.30	14711.09
1641432_a_at	Prosalpha5	20s proteasome	up	2.20	3.17E-02	CG10938-RA	FBgn0016697	36951	4262.81	9391.19
1639838_at	CG8483	---	up	2.20	2.36E-02	CG8483-RA	FBgn0038126	41623	868.69	1913.09
1623288_at	Pros26	Proteasome subunit 20S	up	2.19	3.06E-02	CG4097-RA	FBgn0002284	39855	3546.38	7767.48
1625710_at	CG31717	---	up	2.19	4.06E-02	CG31717-RA	FBgn0051717	318911	1078.76	2361.30
1627502_at	CG9304	---	up	2.19	1.57E-02	CG9304-RA	FBgn0034674	37500	286.91	627.74
1632650_at	CG5867	---	up	2.19	4.01E-02	CG5867-RA	FBgn0027586	34728	5896.46	12885.82
1633264_at	CG13024	---	up	2.18	3.56E-03	CG13024-RA	FBgn0036665	39880	723.89	1580.70
1635163_at	CG5731	---	up	2.18	4.59E-03	CG5731-RA	FBgn0032192	34355	234.89	512.84
1637723_at	mod(mdg4)	Modifier67.2	up	2.18	6.46E-04	CG32491-RW	FBgn0002781	49228	491.62	1069.79
1636997_at	CG18528	---	up	2.17	4.38E-02	CG18528-RA	FBgn0039189	42906	278.40	604.90
1641633_at	CG15544	---	up	2.17	7.23E-03	CG15544-RA	FBgn0039804	43655	238.88	518.18
1637918_s_at	CG16786	---	up	2.17	4.10E-02	CG16786-RA	FBgn0034974	37856	550.27	1192.03
1637391_at	Cpr76Bd	CG9299	up	2.17	8.23E-03	CG9299-RA	FBgn0036881	40123	380.56	824.19
1624907_at	Tsp42Ee	tetraspanin 42E	up	2.16	2.93E-02	CG10106-RA	FBgn0029506	35614	2267.58	4905.29
1632558_at	CG5630	---	up	2.16	6.28E-04	S.C3R002243	FBgn0038842	42478	357.55	772.77
1630224_at	CG6410	---	up	2.16	1.63E-02	CG6410-RA	FBgn0034265	37015	193.62	418.16
1638226_at	CG10562	---	up	2.16	8.00E-03	CG10562-RA	FBgn0039326	43066	784.76	1694.11
1638498_s_at	CG1600	---	up	2.16	2.53E-02	CG1600-RA	FBgn0033188	35687	6445.83	13903.60
1629120_at	alpha-Man-I	alpha Mannosidase I	up	2.15	1.09E-02	CG32682-RA	FBgn0259170	31957	448.47	965.54
1637736_a_at	GalNAc-T2	polypeptide GalNAc transferase 7	up	2.15	1.10E-03	CG6394-RA	FBgn0030930	32836	141.99	305.37
1641350_at	p47	p47	up	2.15	4.39E-02	CG11139-RA	FBgn0033179	35674	943.16	2028.01
1625761_a_at	Mmp1	Matrix metalloproteinase 1	up	2.15	1.67E-02	CG4859-RB	FBgn0035049	37949	368.86	792.36
1629727_at	CG14258	---	up	2.14	4.69E-03	CG14258-RA	FBgn0039482	43260	106.22	227.33
1623083_at	CG1698	---	up	2.14	2.78E-02	CG1698-RA	FBgn0033443	36006	523.23	1118.69
1639784_at	Pros45	Pros45	up	2.13	4.51E-02	CG1489-RA	FBgn0020369	33105	3705.52	7882.45
1624614_at	CG14894	---	up	2.13	2.37E-03	CG14894-RA	FBgn0038428	41993	284.49	605.14
1640710_at	Rpt4	Rpt4	up	2.13	3.50E-02	CG3455-RA	FBgn0028685	31567	4034.15	8575.65
1627678_at	CG18302	---	up	2.12	6.21E-03	CG18302-RA	FBgn0032266	34452	427.95	909.08
1633319_at	CG14956	---	up	2.12	4.29E-04	CG14956-RA	FBgn0035403	38377	249.54	529.07
1626787_s_at	CG17556 /// CG3678	--- /// ---	up	2.12	1.92E-02	CG17556-RA	0038461 /// FBgn003	42045 /// 42046	1471.82	3117.82
1630256_at	lectin-24Db	Lectin24Db	up	2.12	3.34E-02	CG2958-RA	FBgn0040102	53550	104.18	220.42
1628979_at	CG32396	---	up	2.11	3.62E-02	CG32396-RA	FBgn0052396	117406	29.80	62.93
1640091_at	CG13005	---	up	2.11	4.59E-02	CG13005-RA	FBgn0030794	32670	25.64	54.09
1627637_at	d	myosin 29D	up	2.11	4.69E-02	CG10595-RA	FBgn0086896	34179	143.74	303.01
1623757_at	Cry	drosocrystallin	up	2.11	2.30E-02	CG16963-RA	FBgn0005664	34604	8369.64	17618.46
1630593_at	NLaz	Neural Lazarillo	up	2.10	6.71E-03	CG33126-RA	FBgn0053126	33324	1964.36	4121.32
1635298_at	CG13458	---	up	2.09	2.23E-02	CG13458-RA	FBgn0036479	39646	120.57	251.75
1631454_a_at	pio	piopio	up	2.09	5.70E-03	CG3541-RC	FBgn0020521	37929	46.61	97.31
1628390_at	flower	flower	up	2.09	2.57E-02	CG6151-RA	FBgn0261722	---	1702.29	3552.31
1623088_at	CG10855	---	up	2.08	3.40E-03	CG10855-RA	FBgn0035461	38443	111.16	230.66
1636750_s_at	CG13737	---	up	2.07	3.88E-03	CG13737-RA	FBgn0036382	39531	199.44	413.07
1631549_s_at	CG18278 /// CG30059	--- /// ---	up	2.07	4.56E-03	CG18278-RA	0033836 /// FBgn026	246425 /// 36487	63.80	131.78
1632960_at	Eh	Eclosion hormone	up	2.07	3.46E-02	CG5400-RA	FBgn0000564	42101	239.31	494.23
1631973_at	Ote	otefin	up	2.06	3.37E-02	CG5581-RA	FBgn0003022	37090	38.10	78.65
1640352_at	CG13779	---	up	2.06	1.82E-02	CG13779-RA	FBgn0040954	50428	3985.83	8227.04

1628859_at	shi	dynamin	up	2.06	4.24E-03	CG18102-RC	FBgn0003392	45928	1407.49	2904.02
1632986_a_at	CG15088	---	up	2.06	3.25E-03	CG15088-RB	FBgn0034381	37157	263.34	542.03
1639823_at	e	ebony	up	2.06	2.19E-02	CG3331-RA	FBgn0000527	42521	1173.85	2414.67
1640877_at	Tgt	tRNA-guanine transglycosylase	up	2.05	2.07E-02	CG4947-RA	FBgn0031321	33307	38.41	78.92
1628117_at	Hsp27	heat shock protein hsp27	up	2.05	2.30E-03	CG4466-RA	FBgn0001226	39078	164.99	339.00
1626682_s_at	CG9135	---	up	2.05	9.13E-03	CG9135-RA	FBgn0031769	33850	109.66	225.27
1633946_at	CG31955	---	up	2.05	4.97E-03	CG31955-RA	FBgn0051955	319045	246.81	505.59
AFFX-r2-Dros-GAPDH-3_a_at	---	---	up	2.05	1.79E-02	AFFX-Dm-Gpdh-3	---	---	7232.36	14807.01
1638081_at	CG3119	---	up	2.05	4.87E-02	CG3119-RA	FBgn0031466	33479	33.77	69.13
1640637_at	MED19	Mediator complex subunit 19	up	2.04	5.61E-03	CG5546-RA	FBgn0036761	39987	433.07	884.49
1639747_at	CG30291 /// grau	--- /// grauzone	up	2.04	4.23E-03	CG30291-RA	0001133 /// FBgn005	246534 /// 45871	207.84	423.90
1629523_at	lola	longitudinals lacking	up	2.04	3.04E-03	CG12052-RS	FBgn0005630	44548	162.35	331.04
1638876_at	Rpn2	Rpn2	up	2.04	8.96E-03	CG11888-RA	FBgn0028692	43449	4289.47	8740.31
1637645_at	miple2	miple2	up	2.04	2.57E-02	CG18321-RA	FBgn0029002	44787	702.07	1429.91
1624724_at	b6	b6	up	2.04	3.62E-02	CG3100-RA	FBgn0024897	31124	199.85	406.92
1634125_at	CG30440	---	up	2.03	5.01E-03	CG30440-RA	FBgn0050440	35496	170.66	346.94
1616608_a_at	Gpdh	5' gene	up	2.03	2.22E-02	CG9042-RB	FBgn0001128	33824	7193.97	14622.93
1635329_at	CG7638	---	up	2.03	5.63E-03	CG7638-RA	FBgn0036133	39250	469.19	950.57
1636118_at	CG13065	---	up	2.03	3.76E-02	CG13065-RA	FBgn0036590	39786	579.92	1174.66
1638621_at	CG31648	Complex IV assembly protein COX	up	2.03	7.13E-03	CG31648-RA	FBgn0051648	33776	187.85	380.47
1638522_at	CG12123 /// DsimCG12123	--- /// ---	up	2.03	9.81E-03	CG12123-RA	0030039 /// FBgn008	31777	245.14	496.44
1639466_s_at	CG30101	---	up	2.02	4.96E-02	CT34188	FBgn0050101	36950	187.36	379.04
1625297_at	CG8128	---	up	2.02	6.88E-03	CG8128-RA	FBgn0030668	32504	1283.19	2594.04
1634707_s_at	Gfat1	Glutamine:fructose-6-phosphate a	up	2.02	9.22E-03	CG12449-RA	FBgn0027341	3354921	2620.86	5290.26
1636168_s_at	CG13101	---	up	2.02	2.68E-02	CG13101-RA	FBgn0032084	34221	204.97	413.22
1635072_at	CG7214	---	up	2.01	1.19E-02	CG7214-RA	FBgn0031940	34053	7507.59	15126.56
1633405_s_at	CG4288	---	up	2.01	6.42E-04	CG4288-RB	FBgn0038799	42426	427.86	861.75
1628613_a_at	CG30492 /// CG30494	--- /// ---	up	2.01	9.97E-04	CG30492-RE	0027604 /// FBgn005	35698 /// 35699	539.70	1085.01
1636154_at	CG30090	---	up	2.00	1.81E-02	CG30090-RA	FBgn0050090	246448	32.03	64.13
1629846_at	CG11619	---	up	2.00	1.00E-02	CG11619-RA	FBgn0036836	40072	129.91	260.11
1637545_at	prtp	pretaporter	up	2.00	1.59E-02	CG1837-RA	FBgn0030329	32124	1833.52	3670.29
1638050_s_at	Sox14	Sox box protein 14	up	2.00	4.81E-02	CG3090-RA	FBgn0005612	37822	880.69	1759.70
1626846_s_at	CG5973	---	up	2.00	4.93E-04	CG5973-RA	FBgn0031914	34023	1566.35	3125.59
1641042_at	CG16820	---	up	2.00	5.30E-04	CG16820-RA	FBgn0032495	34730	507.64	1012.89
1631954_at	Ranbp9	Ranbp9	up	1.99	2.14E-03	CG5252-RA	FBgn0037894	41367	208.27	415.37
1638324_s_at	bond	james bond	up	1.99	7.11E-03	CG6921-RB	FBgn0260942	42657	1811.48	3612.03
1624328_a_at	set-01	septin	up	1.99	1.36E-02	CG1403-RB	FBgn0011710	33114	80.30	160.07
1628466_s_at	Rpn6	Proteasome p44.5 subunit	up	1.99	9.24E-03	CG10149-RA	FBgn0028689	36638	5610.58	11165.93
1632543_at	CG9003	---	up	1.99	3.11E-02	CG9003-RA	FBgn0033639	36244	528.46	1049.83
1627068_at	Cyp4aa1	Cyp4aa1	up	1.99	1.56E-02	CG8302-RA	FBgn0034053	36752	94.39	187.49
1633166_at	CG12942	---	up	1.99	7.64E-03	CG12942-RA	FBgn0033569	36158	80.14	159.12
1633384_at	Mpp6	M-phase phosphoprotein 6	up	1.99	3.87E-02	CG9250-RA	FBgn0032921	35382	291.42	578.55
1632904_at	Rat1	CG10354	up	1.98	3.93E-02	CG10354-RA	FBgn0031868	33964	147.70	292.99
1634701_at	CG3713	---	up	1.98	2.13E-02	CG3713-RA	FBgn0040343	50365	675.74	1334.86
1625279_a_at	tup	islet	up	1.98	3.70E-02	CG10619-RA	FBgn0003896	35147	76.97	152.03
1637551_at	CG1887	---	up	1.97	1.65E-02	CG1887-RA	FBgn0035290	38252	157.56	311.06
1630201_a_at	CG11321	---	up	1.97	1.05E-02	CG11321-RA	FBgn0031857	33950	255.99	504.92
1632775_at	CG42254	CR30478	up	1.97	3.67E-02	CG12854-RA	FBgn0259112	36644	73.47	144.92
1628662_at	CG13531	---	up	1.97	1.05E-03	CG13531-RB	FBgn0034786	37627	489.24	964.13
1633303_at	Cht6	CG2989	up	1.97	1.87E-02	CG2989-RA	FBgn0030171	31935	440.75	867.98
1634162_at	CG40198	---	up	1.97	4.16E-03	CG40198-RA	FBgn0058198	3354922	1832.36	3608.27
1629715_at	Gie	CG7891	up	1.97	8.37E-04	CG7891-RA	FBgn0037551	40961	742.74	1459.73
1625600_a_at	Asph	Aspartyl beta-hydroxylase	up	1.96	2.07E-02	CG8421-RE	FBgn0034075	36778	72.96	143.21
1640580_at	Ir68a	CG6185	up	1.96	4.96E-02	CG6185-RA	FBgn0036150	39269	49.70	97.53
1629559_s_at	Atet	ABC transporter expressed in trac	up	1.96	4.71E-04	CG2969-RA	FBgn0020762	33636	1338.00	2624.71
1641041_at	CG18467	---	up	1.96	3.73E-03	CG18467-RA	FBgn0034218	36958	584.12	1145.28
1631387_at	CG30344	---	up	1.96	2.33E-02	CG30344-RA	FBgn0050344	246552	1338.29	2623.52
1639140_at	casp	casper	up	1.95	8.91E-03	CG8400-RA	FBgn0034068	36769	1046.74	2044.14
1640365_s_at	fusl	CG14021	up	1.95	1.43E-02	CG14021-RB	FBgn0031702	33765	450.10	878.88
1635950_at	Su(P)	Suppressor of ref(2)P sterility	up	1.95	7.36E-04	CG4086-RA	FBgn0004465	39856	606.96	1185.13
1632432_at	CG9850	Dlg-interacting metalloprotease	up	1.95	9.34E-04	CG9850-RA	FBgn0034903	37762	851.03	1660.19
1628008_a_at	TER94	ter94	up	1.95	2.06E-02	CG2331-RA	FBgn0261014	36040	5508.70	10732.70
1624074_at	Obp56a	Odorant-binding protein 56a	up	1.95	3.24E-02	CG11797-RA	FBgn0034468	37264	57.47	111.94
1630005_at	Ubi-p5E	Ubiquitin-5E	up	1.94	5.78E-04	CG32744-RA	FBgn0086558	326237	6841.80	13301.30
1632664_at	Osi12	Osiris	up	1.94	5.97E-03	CG1154-RA	FBgn0037419	40766	59.72	115.67
1636842_at	CG8460	---	up	1.93	3.80E-02	CG8460-RA	FBgn0031996	34115	153.63	297.27
1623851_at	CG9400	---	up	1.93	1.83E-03	CG9400-RA	FBgn0030562	32385	2399.70	4638.58
AFFX-r2-Dros-GAPDH-5_a_at	---	---	up	1.93	2.55E-02	AFFX-Dm-Gpdh-5	---	---	247.34	477.88
1639621_at	CG10916	---	up	1.93	1.20E-02	CG10916-RA	FBgn0034312	37081	365.26	704.56
1635028_s_at	CG11714 /// Fuca	--- /// alpha-L-fucosidase	up	1.93	1.44E-02	CG33047-RA	0036169 /// FBgn003	3772566 /// 3772574	153.51	296.03
1639933_at	tw	Protein O-mannosyltransferase 2	up	1.93	4.18E-02	CG12311-RA	FBgn0086368	31024	102.53	197.68
1628190_at	CG33981 /// fab1	BEST:GH01256 /// PtdIns(3)P 5-ki	up	1.92	3.45E-02	CG6355-RA	0028741 /// FBgn025	37033 /// 3885654	45.85	88.14
1626893_at	Gasp	Gasp	up	1.92	6.69E-03	CG10287-RA	FBgn0026077	40745	8532.38	16392.06
1638724_at	CG18507	---	up	1.92	2.13E-02	CG18507-RA	FBgn0028527	34775	431.60	828.79
1641039_at	rtv	retroactive	up	1.92	7.45E-03	CG1397-RA	FBgn0261277	32056	142.57	273.58
1626967_at	CalpB	Calpain B	up	1.92	2.17E-04	CG8107-RA	FBgn0025866	39165	2059.08	3948.86
1641105_at	MED20	Mediator complex subunit 20	up	1.92	1.51E-02	CG18780-RA	FBgn0013531	43952	113.27	217.10
1636797_at	Aats-ser	Seryl-tRNA synthetase	up	1.91	3.23E-03	CG4938-RA	FBgn0021750	41862	179.15	342.96
1632565_at	Bin1	Sin3A-associated polypeptide p18	up	1.91	6.76E-03	CG6046-RA	FBgn0024491	41965	464.13	888.28
1636718_s_at	CG4502	---	up	1.91	1.75E-05	CG4502-RB	FBgn0031896	34002	3101.31	5925.85
1623666_at	CG10516	---	up	1.91	4.45E-02	CG10516-RA	FBgn0036549	39738	609.77	1164.99
1629794_at	iHog	interference Hedgehog	up	1.91	1.96E-02	CG9211-RA	FBgn0031872	33972	114.86	219.43
AFFX-r2-Dros-GAPDH-3_at	---	---	up	1.91	1.71E-02	AFFX-Dm-Gpdh-3	---	---	1563.09	2985.21
1635196_at	CG6836	---	up	1.91	2.84E-04	CG6836-RA	FBgn0036834	40070	386.78	737.83
1625949_at	Gpdh	5' gene	up	1.91	1.20E-02	CG9042-RB	FBgn0001128	33824	1542.68	2939.27
1630348_a_at	CG5059	---	up	1.90	1.18E-03	CG5059-RA	FBgn0037007	40278	4287.55	8165.26
1638365_at	CG3244	---	up	1.90	2.48E-03	CG3244-RA	FBgn0031629	33678	5051.18	9616.81
1632898_at	CG33978	---	up	1.90	2.17E-02	HDC16673	FBgn0053978	3885646	427.29	812.60
1641558_at	CG31005	---	up	1.90	5.88E-03	CG31005-RA	FBgn0051005	43697	1011.43	1918.88
1631768_at	qkr58E-1	KH-domain	up	1.90	4.13E-02	CG3613-RA	FBgn0022986	37561	171.93	326.04
1635683_at	CG6136	---	up	1.90	4.96E-03	CG6136-RA	FBgn0038332	41877	62.36	118.22
1640489_at	CG18522 /// DsecCG18522	--- /// ---	up	1.89	1.89E-03	CG18522-RA	0038347 /// FBgn026	41894	529.74	1003.74
1640473_at	CG34120	---	up	1.89	3.01E-02	CG1819-RA	FBgn0083956	318066	375.30	710.77
1633834_at	CG9701	---	up	1.89	2.79E-03	CG9701-RA	FBgn0036659	39872	734.93	1389.32
1624032_at	CG3887	---	up	1.89	6.29E-03	CG3887-RA	FBgn0031670	33725	1774.67	3351.39
1633759_s_at	---	---	up	1.89	1.15E-02	CG14066-RD	---	---	170.42	321.24
1635363_a_at	CG1832 /// Jupiter	--- /// Jupiter	up	1.88	2.44E-02	CG31363-RD	0032979 /// FBgn005	35445 /// 41392	2906.07	5476.81
1625582_at	CG32187	---	up	1.88	7.11E-04	CG32187-RA	FBgn0052187	39980	77.95	146.90
1632011_at	CG31274 /// MESK4	--- /// Misexpression suppressor of	up	1.88	2.34E-02	CG31447-RA	0043069 /// FBgn005	318655 /// 64872	470.95	887.45
1629039_at	asrij	asrij	up	1.88	9.16E-03	CG13533-RA	FBgn0034793	37637	177.43	333.96
1635764_at	314696 /// DanaGF16171 /// DvarCG146	--- /// --- /// ---	up	1.88	3.43E-02	CG14696-RA	/// FBgn0066150 ///	41317	239.21	450.22
1629876_at	Cks85A	Cyclin-dependent kinase subunit 8	up	1.88	4.00E-02	CG9790-RA	FBgn0037613	41034	454.79	854.94
1629070_at	CG1208	---	up	1.88	1.56E-02	CG1208-RA	FBgn0037386	40727	635.69	1192.62
1640059_at	CG6040	---	up	1.88	2.29E-03	CG6040-RA	FBgn0038679	42292	1040.85	1951.80
1631701_a_at	Cpr49Ac	CG8502	up	1.87	2.15E-02	CG8502-RC	FBgn0033725	36348	49.18	92.08
1637195_at	CG31321	---	up	1.87	1.40E-02	CG31321-RB	FBgn0051321	318681	523.45	979.73
1640235_at	W	head involution defect	up	1.87	3.61E-02	CG5123-RA	FBgn0003997	40009	366.54	685.37
1640795_at	CG13280	---	up	1.87	4.07E-02	CG13280-RA	FBgn0032609	35015	35.13	65.64
1626970_at	Ugt35b	UDP-glycosyltransferase	up	1.86	5.44E-03	CG6649-RA	FBgn0026314	41333	1625.47	3027.67
1623984_s_at	Mical	mical	up	1.86	5.95E-04	CG33208-RB	FBgn0053208	41225	518.19	965.07
1623066_at	Irk3	Inwardly rectifying potassium char	up	1.86	2.46E-03	CG10369-RA	FBgn0032706	35131	114.95	213.83
1639351_at	Cpr97Eb	CG15884	up	1.86	7.55E-04	CG15884-RA	FBgn0039481	43259	2988.87	5558.19
1625405_at	yellow-c	yellow-c	up	1.86	8.27E-04	CG4182-RA	FBgn0041713	34879	2483.46	4615.12
1640526_at	CG1103	---	up	1.86	2.40E-02	CG1103-RA	FBgn0037235	40547	985.04	1828.11
1635263_at	CG11825	---	up	1.86	4.91E-03	CG11825-RA	FBgn0033519	36099	140.17	260.01
1630399_at	Cad99C	Cad99C	up	1.85	8.66E-03	CG31009-RA	FBgn0039709	43528	324.75	602.13
1624592_at	CG5188	---	up	1.85	4.38E-02	CG5188-RA	FBgn0032247	34428	151.03	279.80
1627302_at	Fmo-2	Flavin-containing monooxygenase	up	1.85	2.32E-03	CG3174-RA	FBgn0033079	35561	461.41	854.22
1624970_s_at	Cul-3	Cullin3	up	1.85	1.55E-02	CG11861-RC	FBgn0261268	34896	606.14	1121.74
1639778_at	CG18304	---	up	1.85	3.72E-03	CG18304-RA	FBgn0031869	33969	162.53	300.27
1623957_s_at	GstS1	glutathione-S-transferase	up	1.85	1.57E-04	CG8938-RB	FBgn0010226	36927	3106.51	5738.00
1628635_at	Osi9	Osiris	up	1.85	4.82E-02	CG15592-RA	FBgn0037416	40763	106.52	196.71
1631112_at	Epac	Epac	up	1.84	1.60E-02	CG3427-RA	FBgn0085421	35588	397.38	732.28
1639253_at	CG11242	---	up	1.84	2.12E-02	CG11242-RA	FBgn0034451	37244	1072.06	1974.22
1637113_s_at	abba	CG15105	up	1.84	3.04E-02	CG15105-RB	FBgn0034412	37190	5963.59	10976.13
1632166_at	Plap	Phospholipase A2 activator protei	up	1.84	2.61E-02	CG5105-RA	FBgn0024314	43978	369.83	679.81
1628834_a_at	SelD	Selenide,water dikinase	up	1.83	7.24E-04	CG8553-RB	FBgn0261270	36587	328.86	601.75
1631062_at	CG2023	---	up	1.83	2.53E-02	CG2023-RA	FBgn0037383	40724	113.78	208.02
1631119_at	Cip4	Cip4	up	1.83	3.76E-05	CG15015-RA	FBgn0035533	38534	631.29	1153.00
1628840_at	ken	ken barbie	up	1.82	4.00E-02	CG5575-RA	FBgn0011236	37785	568.30	1037.06
1639181_at	CG14598	---	up	1.82	2.59E-02	CG14598-RA	FBgn0037503	40893	100.34	182.84
1631217_a_at	TpnC73F	troponin C	up	1.82	1.95E-03	CG7930-RA	FBgn0010424	39916	114.55	208.65
1626550_at	CG34449	---	up	1.82	3.44E-02	CG32705-RA	FBgn0085478	31887	47.53	86.54
1623706_at	CG9498	---	up	1.82	6.26E-03	CG9498-RA	FBgn0031801	33885	694.05	1263.26
1630168_at	CG31636	---	up	1.82	2.86E-02	CG31636-RA	FBgn0051636	318865	87.43	159.08
1629609_at	GRHR	hormone receptor	up	1.82	4.57E-02	CG11325-RB	FBgn0025595	33942	36.08	65.54
1627532_at	Rpb4	Rpb4	up	1.82	7.35E-05	CG31237-RA	FBgn0053520	326128	378.08	686.39
1627783_at	CG18599	---	up	1.81	1.99E-02	CG18599-RA	FBgn0038592	42191	58.56	106.28
1629940_at	CG13895	---	up	1.81	2.32E-02	CG13895-RA	FBgn0035158	38087	141.10	256.04
1638112_at	CG1908	---	up	1.81	2.57E-02	CG1908-RA	FBgn0030274	32055	945.55	1715.06
1629551_s_at	CG12268	---	up	1.81	2.13E-02	CG12268-RA	FBgn0039131	42833	300.71	543.50
1639142_s_at	---	---	up	1.81	2.51E-02	CR_tc_GH14469	---	---	172.65	311.94
1623949_s_at	CG32816	---	up	1.81	3.18E-02	CG32816-RB	FBgn0052816	318225	238.24	430.28
1627833_at	Cralbp	Cellular retinaldehyde binding prot	up	1.81	7.99E-03	CG10546-RA	FBgn0035636	38651	173.62	313.55
1637145_at	CG14787	---	up	1.80	3.63E-02	CG14787-RA	FBgn0027793	31096	111.79	201.37
1637504_at	CG17754	---	up	1.80	1.88E-02	CG17754-RD	FBgn0030114	31873	1032.61	1859.85
1638795_at	sec6	sec6	up	1.80	1.37E-02	CG5341-RA	FBgn0034367	37142	194.77	350.64
1623299_at	Mmp2	Matrix metalloproteinase 2	up	1.80	1.06E-02	CG1794-RA	FBgn0033438	35997	244.98	440.68
1625040_a_at	CG13502	---	up	1.80	4.28E-02	CG13502-RB	FBgn0034692	37518	41.13	73.95
1633153_at	cdc14	CG7134	up	1.80	1.98E-02	CG7134-RA	FBgn0031952	34067	145.03	260.37
1626016_s_at	tmod	Tropomodulin	up	1.79	8.84E-03	CG1539-RE	FBgn0082582	43633	9593.04	17160.25
1631584_at	CG31189	---	up	1.78	3.69E-02	CG31189-RA	FBgn0051189	42487	548.32	978.64
1639999_at	CG2662	shrunken nuts	up	1.78	1.07E-02	CG2662-RA	FBgn0024993	31254	124.72	222.38
1632403_at	CG12263	---	up	1.78	5.20E-03	CG12263-RA	FBgn0034346	37118	205.93	366.87
1634170_a_at	jbug	filamin	up	1.78	3.88E-02	CG30092-RB	FBgn0028371	43997	427.08	760.22
1623491_at	CG15155	---	up	1.78	1.99E-03	CG15155-RA	FBgn0032669	35087	85.53	152.02
1636837_at	CG32177	---	up	1.78	4.72E-04	CG32177-RA	FBgn0052177	326197	941.22	1672.23
1624432_at	spz3	Spz3	up	1.78	1.29E-02	CG7104-RA	FBgn0031959	34077	392.26	696.26
1626194_at	melt	melted	up	1.77	2.71E-02	CG8624-RA	FBgn0023001	38785	109.55	194.36
1638858_at	CG42235	CG31090	up	1.77	4.07E-02	CG8934-RA	FBgn0250757	43136	112.46	199.02
1624616_at	Trh	Tryptophan hydroxylase	up	1.77	1.12E-03	CG9122-RA	FBgn0035187	38121	137.19	242.55
1638079_at	l(2)05510	lethal (2) 05510	up	1.77	2.81E-02	CG13432-RA	FBgn0028622	47065	1686.02	2980.24
1636756_at	CG31627	---	up	1.77	4.95E-02	CG31627-RA	FBgn0051627	318860	60.09	106.10
1638301_at	CG8157	---	up	1.77	2.79E-02	CG8157-RA	FBgn0034010	36700	476.14	840.47
1631307_at	CG11438	---	up	1.77	1.39E-02	CG11438-RA	FBgn0037164	40469	284.36	501.93
1635639_a_at	Eip74EF	ecdysone inducible protein	up	1.76	8.99E-03	CG32180-RA	FBgn0000567	39962	1223.74	2158.87
1626109_a_at	Drip	aquaporin	up	1.76	1.99E-02	CG9023-RA	FBgn0015872	36236	362.66	639.07
1631614_at	CG5190	---	up	1.76	2.44E-02	CG5190-RA	FBgn0034351	37124	140.05	246.63
1629716_a_at	CG13284	---	up	1.76	1.57E-02	CG13284-RA	FBgn0032614	35021	239.19	421.10
1634671_a_at	CG41284 /// CG42598	--- /// ---	up	1.76	2.66E-03	CG40386-RB	0085599 /// FBgn026	5740851 /// 8674042	39.17	68.96
1636440_at	Sip1	SRY interacting protein 1	up	1.76	9.59E-04	CG10939-RA	FBgn0010620	44155	954.49	1677.13
1628271_at	CG3609	---	up	1.75	1.93E-02	CG3609-RA	FBgn0031418	33421	935.89	1640.87
1636486_at	Gdi /// Taf11	Gdi-related /// TBP-associated fac	up	1.75	1.29E-02	CG4079-RA	0004868 /// FBgn001	34264 /// 34293	237.86	416.89
1641169_s_at	CG11050	---	up	1.75	6.50E-03	CG11050-RA	FBgn0031836	33924	2500.62	4382.77
1622973_at	CG8031	---	up	1.75	9.60E-03	CG8031-RA	FBgn0038110	41601	732.29	1282.70
1636245_at	T48	48' transcript	up	1.75	1.86E-02	CG5507-RA	FBgn0004359	43235	156.03	272.77
1634121_at	Obp57c	Odorant-binding protein 57c	up	1.74	3.24E-03	CG13421-RA	FBgn0034509	37311	2678.12	4669.69
1637353_at	Dat	Arylalkylamine N-acetyltransferase	up	1.74	3.36E-03	CG3318-RA	FBgn0019643	37867	2876.33	5012.92
1633574_at	CG31820	---	up	1.74	3.77E-03	CG31820-RA	FBgn0051820	318961	23.61	41.10

1640386_at	wbl	windbeutel	up	1.74	9.81E-03	CG7225-RA	FBgn0004003	37206	102.03	177.50
1641688_at	CG18136	---	up	1.74	2.02E-02	CG18136-RA	FBgn0036839	40075	140.15	243.66
1639388_a_at	CG12567	---	up	1.74	5.88E-03	CG12567-RB	FBgn0039958	3355094	629.29	1092.25
1623238_at	CG5618	---	up	1.73	6.10E-03	CG5618-RA	FBgn0036975	40241	2532.81	4394.26
1632498_at	CG15629	---	up	1.73	3.21E-02	CG15629-RA	FBgn0031630	33679	565.98	981.61
1640639_at	CG42235	CG31090	up	1.73	3.67E-02	CG8966-RA	FBgn0250757	43136	31.49	54.61
1631874_at	---	---	up	1.73	2.18E-02	CG40295-RA	---	---	133.71	231.82
1636091_at	fj	four-jointed	up	1.73	1.35E-02	CG10917-RA	FBgn0000658	37089	158.08	274.01
1641109_at	obst-B	CG4778	up	1.73	3.95E-02	CG4778-RA	FBgn0027600	34336	621.37	1076.99
1628099_at	bor	belphegor	up	1.73	4.10E-03	CG6815-RA	FBgn0040237	53565	579.13	1003.57
1627835_a_at	Cda5	CG31973	up	1.73	2.86E-02	CG31973-RA	FBgn0051973	33158	1674.07	2900.49
1641607_at	Nc	Nedd2-like caspase	up	1.73	9.00E-04	CG8091-RA	FBgn0026404	39173	265.66	460.19
1635006_at	/// DgriCG7077 /// DmojCG7077 /// Dvir	--- /// --- /// --- /// ---	up	1.73	5.79E-03	CG7077-RA	0086919 /// FBgn008	42614	2445.68	4234.51
1627399_at	CG17266	---	up	1.73	3.47E-02	CG17266-RA	FBgn0033089	35571	55.13	95.36
1639149_at	CG15313	---	up	1.73	4.46E-02	CG15313-RA	FBgn0030173	31937	217.19	375.57
1634668_at	Ice	Ice	up	1.73	2.23E-03	CG7788-RA	FBgn0019972	43514	1083.76	1873.39
1634606_a_at	mmy	cabrio	up	1.73	3.26E-04	CG9535-RA	FBgn0259749	33903	1530.37	2644.40
1641423_at	CG6739	---	up	1.73	4.74E-02	CG6739-RA	FBgn0031926	34037	627.55	1083.63
1631330_at	CG3408	---	up	1.72	2.01E-02	CG3408-RA	FBgn0036008	39107	122.61	211.33
1631273_at	CG5262	---	up	1.72	9.05E-03	CG5262-RA	FBgn0036988	40258	394.90	680.43
1626143_a_at	CG32138	---	up	1.72	5.60E-03	CG32138-RB	FBgn0052138	39561	223.24	384.53
1636151_at	CG32196	---	up	1.72	7.86E-03	CG32196-RA	FBgn0052196	317908	182.74	313.65
1630802_at	Cyp6d4	Cyp6d4	up	1.72	2.88E-03	CG12800-RA	FBgn0039006	42682	166.59	285.92
1634952_s_at	CG15094	---	up	1.71	1.24E-03	CG15094-RA	FBgn0034392	37168	346.64	594.17
1640280_at	lola	longitudinals lacking	up	1.71	9.26E-03	CG12052-RC	FBgn0005630	44548	709.71	1216.31
1634742_at	Sodh-2	Sorbitol dehydrogenase-2	up	1.71	4.11E-03	CG4649-RA	FBgn0022359	41313	461.57	790.37
1639396_s_at	CDase	Ceramidase	up	1.71	4.45E-05	CG1471-RB	FBgn0039774	43618	707.61	1211.67
1635943_s_at	CG10947	---	up	1.71	2.42E-02	CG10947-RA	FBgn0032857	35309	81.59	139.68
1630419_a_at	CG7300	---	up	1.71	4.61E-02	CG7300-RB	FBgn0032286	34472	637.93	1090.62
1640326_at	CG30381	---	up	1.71	1.81E-02	Dm.2R.17149.0	FBgn0050381	246580	50.92	87.03
1634388_at	CG30197	---	up	1.71	1.86E-02	CG30197-RA	FBgn0050197	246513	13604.32	23217.16
1632776_at	CSN5	JUN activation domain-binding pro	up	1.71	2.58E-02	CG14884-RA	FBgn0027053	42000	546.77	933.12
1641703_at	CG2147	---	up	1.71	9.27E-03	CG2147-RA	FBgn0030025	31759	290.46	495.65
1632348_at	CG9452	---	up	1.71	3.26E-02	CG9452-RA	FBgn0036877	40119	75.52	128.80
1629318_at	CG17104	---	up	1.70	4.43E-02	CG17104-RA	FBgn0040496	34465	186.00	317.08
1636471_at	mod(mdg4)	Modifier67.2	up	1.70	5.63E-04	CG32491-RY	FBgn0002781	49228	113.42	193.32
1627721_at	CG12880	---	up	1.70	2.03E-02	CG12880-RA	FBgn0046258	43328	178.72	304.31
1635975_s_at	---	---	up	1.70	1.26E-02	RE54004	---	---	1035.85	1763.40
1640707_at	CG15772	---	up	1.70	2.88E-02	CG15772-RA	FBgn0029799	31498	528.32	898.97
1637412_a_at	sas	responsible for dominant phenotyp	up	1.70	1.11E-03	CG2507-RB	FBgn0002306	40861	471.82	802.62
1639680_at	---	---	up	1.70	4.48E-03	HDC15078	---	---	2115.34	3594.70
1634662_at	Cyp313a1	Cyp313a1	up	1.70	6.13E-03	CG3360-RA	FBgn0038236	41759	5339.55	9067.99
1628226_at	CG8539 /// DsimCG8539	--- /// ---	up	1.70	5.99E-03	CG8539-RA	0035791 /// FBgn008	38842	386.11	655.39
1634165_at	CG11378	---	up	1.70	5.31E-04	CG11378-RA	FBgn0040364	31060	1094.35	1857.36
1641579_at	mRpS26	mitochondrial ribosomal protein S2	up	1.70	8.48E-03	CG7354-RA	FBgn0036774	40001	331.68	562.91
1636716_at	Cyp4c3	Cytochrome P450-4c3	up	1.69	5.08E-03	CG1438-RA	FBgn0015032	43663	210.65	356.24
1624834_at	CG14015	---	up	1.69	3.56E-02	CG14015-RA	FBgn0031716	33780	298.89	504.01
1633290_a_at	mol	numb-interacting protein	up	1.69	3.07E-02	CG4482-RA	FBgn0086711	34872	595.04	1003.27
1640610_at	CG6613	---	up	1.68	2.07E-02	CG6613-RA	FBgn0034694	37520	235.60	396.63
1629511_at	CG5326	---	up	1.68	1.41E-02	CG5326-RA	FBgn0038983	42655	1485.96	2501.44
1625604_at	CG14259	---	up	1.68	8.25E-04	CG14259-RA	FBgn0039483	43261	2604.06	4377.04
1633523_at	Pbgs	Porphobilinogen synthase	up	1.68	4.07E-03	CG10335-RA	FBgn0036271	39402	192.28	322.72
1634465_a_at	GluClalpha	indefinite	up	1.68	4.57E-02	CG7535-RB	FBgn0024963	42350	182.20	305.45
1631853_at	CG6610	---	up	1.68	2.46E-03	CG6610-RA	FBgn0035675	38693	297.81	499.17
1626543_at	Sbp2	SECIS-binding protein 2	up	1.68	3.29E-02	CG7066-RA	FBgn0087039	38934	577.52	967.80
1631637_a_at	Cpr	P450 reductase	up	1.68	4.25E-03	CG11567-RA	FBgn0015623	33883	2455.25	4113.49
1628536_s_at	CG11880	---	up	1.68	2.16E-02	CG11880-RA	FBgn0039637	43440	1249.91	2093.69
1626597_at	CG9521	---	up	1.67	1.83E-02	CG9521-RA	FBgn0030588	32414	75.58	126.47
1625421_at	CG30377	---	up	1.67	3.48E-02	CG30377-RA	FBgn0050377	50252	83.51	139.70
1637773_s_at	Baldspot	baldspot	up	1.67	4.88E-02	CG3971-RA	FBgn0260960	39860	1660.08	2775.25
1635783_a_at	CG3119	---	up	1.67	4.14E-03	CG3119-RB	FBgn0031466	33479	52.06	87.01
1623308_at	CG33784	---	up	1.67	1.90E-02	HDC12790	FBgn0053784	3772181	64.36	107.54
1630450_s_at	Thd1	Thd1	up	1.67	1.77E-02	HDC16707	FBgn0026869	43796	131.24	219.05
1638975_at	CG13876	---	up	1.67	1.65E-02	CG13876-RA	FBgn0035109	38031	104.32	174.04
1634468_at	CG13397	---	up	1.67	8.01E-03	CG13397-RA	FBgn0014417	46386	239.36	398.88
1623505_s_at	Ubi-p5E	Ubiquitin-5E	up	1.66	3.58E-02	CG11700-RA	FBgn0086558	326237	15660.45	26070.03
1623934_at	blp	black pearl	up	1.66	4.26E-04	CG5268-RA	FBgn0038387	41937	498.11	828.89
1625587_at	Jheh3	Juvenile hormone epoxide hydrola	up	1.66	2.73E-03	CG15106-RA	FBgn0034406	37182	200.28	332.96
1630280_s_at	CG42353 /// CG42354	--- /// ---	up	1.66	3.58E-02	CG9216-RA	0259699 /// FBgn025	32576 /// 7354437	4916.93	8169.82
1630831_at	CG7695	---	up	1.66	3.22E-04	CG7695-RA	FBgn0038631	42234	971.56	1614.30
1625685_at	Invadolysin	Invadolysin	up	1.66	8.10E-03	CG3953-RA	FBgn0086359	49580	287.21	477.06
1626708_at	Cbl	Cbl	up	1.66	3.82E-03	CG7037-RA	FBgn0020224	38961	270.74	449.60
1624729_at	lola	longitudinals lacking	up	1.66	1.42E-02	CG12052-RL	FBgn0005630	44548	307.04	509.73
1625757_at	gfzf	GST-containing FLYWCH zinc-fing	up	1.66	7.42E-04	CG31492-RA	FBgn0250732	40858	1069.31	1774.55
1632105_a_at	cib	ciboulot	up	1.66	1.06E-02	CG4944-RC	FBgn0026084	31359	2081.29	3453.79
1639256_at	CG5676	---	up	1.66	1.57E-04	CG5676-RA	FBgn0032200	34368	1790.34	2968.87
1640901_at	CG6753	---	up	1.66	4.07E-02	CG6753-RA	FBgn0038070	41557	113.43	188.08
1625944_a_at	CG16838	---	up	1.66	3.02E-02	CG16838-RB	FBgn0036574	39770	111.66	185.13
1633092_at	cup	cup	up	1.66	2.02E-02	CG11181-RA	FBgn0000392	33934	874.90	1449.69
1637763_a_at	CG8298	---	up	1.66	4.10E-02	CG8298-RA	FBgn0033673	36282	140.56	232.84
1639480_at	Cat	catalase	up	1.65	3.44E-02	CG6871-RA	FBgn0000261	40048	3710.56	6138.82
1637129_at	GstE3	Glutathione S transferase E3	up	1.65	2.44E-03	CG17524-RA	FBgn0063497	37108	1728.96	2860.41
1634321_s_at	CG2051	---	up	1.65	1.10E-02	CG2051-RC	FBgn0037376	40716	105.24	173.90
1627249_a_at	l(2)35Bg	transcription unit B	up	1.65	4.57E-02	CG4180-RB	FBgn0001977	49424	467.30	771.62
1627004_at	CG7632	---	up	1.65	5.32E-03	CG7632-RA	FBgn0037071	40357	531.99	877.08
1625683_at	CG5112	---	up	1.65	1.41E-02	CG5112-RA	FBgn0039341	43083	633.74	1043.43
1624121_at	knk	knickkopf	up	1.65	2.40E-02	CG6217-RA	FBgn0001321	47137	430.82	709.21
1631474_s_at	Gpo-1	mitochondrial GPD	up	1.65	6.18E-03	CG8256-RC	FBgn0022160	47611	1735.55	2857.00
1638541_at	mRpL32	mitochondrial ribosomal protein L3	up	1.65	1.33E-02	CG12220-RA	FBgn0039835	43698	315.10	518.59
1635861_a_at	Tpr2	Tetratricopeptide repeat protein 2	up	1.64	3.90E-03	CG4599-RA	FBgn0032586	34984	1324.28	2177.28
1637874_at	Lasp	Lasp	up	1.64	1.85E-02	CG3849-RA	FBgn0063485	39864	284.89	467.68
1637620_s_at	CG9186	---	up	1.64	2.09E-03	CG9186-RB	FBgn0035206	38150	1303.31	2139.19
1625154_s_at	pot	papillote	up	1.64	5.66E-04	CG2467-RA	FBgn0250871	32154	1304.88	2141.56
1637814_s_at	Jafrac2	jafrac2	up	1.64	4.60E-02	CG1274-RA	FBgn0040308	53577	125.55	205.53
1637036_s_at	pr	purple	up	1.64	3.98E-02	CG16784-RC	FBgn0003141	35292	7146.36	11698.49
1639981_at	Ssb-c31a	Single stranded-binding protein c3	up	1.64	3.61E-03	CG8396-RA	FBgn0015299	34120	833.27	1363.73
1637993_at	MED11	Mediator complex subunit 11	up	1.64	3.98E-02	CG6884-RA	FBgn0036811	40042	254.17	415.92
1635376_at	CG9086	---	up	1.64	5.79E-03	CG9086-RA	FBgn0030809	32687	227.63	372.23
1625031_at	CG7841	---	up	1.63	5.02E-04	CG7841-RA	FBgn0036502	39672	462.18	755.17
1623396_at	CG13875	---	up	1.63	4.13E-02	CG13875-RA	FBgn0035104	38024	211.33	345.16
1629963_at	sut1	sugar transporter 1	up	1.63	4.92E-02	CG8714-RA	FBgn0028563	35774	378.30	617.63
1625283_at	mod(mdg4)	Modifier67.2	up	1.63	1.84E-02	CG32491-RM	FBgn0002781	49228	98.59	160.97
1633387_at	bves	bves	up	1.63	2.66E-02	CG32513-RA	FBgn0031150	33083	179.89	293.64
1639118_a_at	Hrb87F	Heterogeneous nuclear ribonucleo	up	1.63	1.85E-03	CG12749-RA	FBgn0004237	48535	1312.93	2142.14
1636726_at	CG17180	---	up	1.63	3.93E-03	CG17180-RA	FBgn0035140	38068	168.40	274.65
1631925_at	Mdr65	P-glycoprotein65-Dm	up	1.63	7.13E-04	CG10181-RA	FBgn0004513	38726	1621.76	2642.94
1637198_at	CG10345	---	up	1.63	1.61E-02	CG10345-RA	FBgn0027562	42017	1044.87	1702.62
1638583_at	CG31103	---	up	1.63	3.86E-03	CG31103-RA	FBgn0051103	43030	722.46	1176.75
1640429_a_at	CG6145	---	up	1.63	2.37E-03	CG6145-RA	FBgn0033853	36506	2731.48	4446.88
1626835_at	Syb	synaptobrevin	up	1.63	1.48E-02	CG12210-RB	FBgn0003660	36080	962.74	1567.07
1633989_at	CG1124	---	up	1.62	3.99E-03	CG1124-RA	FBgn0037290	40613	2454.89	3986.55
1636845_at	CG8885	Sco1 protein homolog	up	1.62	1.21E-03	CG8885-RA	FBgn0031656	33711	528.40	858.03
1626990_s_at	Mipp1	Multiple inositol polyphosphate ph	up	1.62	6.74E-03	CG4123-RB	FBgn0026061	39841	1186.36	1924.70
1638730_at	Hsp67Ba	Gene 1	up	1.62	2.97E-02	CG4167-RA	FBgn0001227	39076	104.85	170.07
1637880_at	CG13606	---	up	1.62	1.52E-02	CG13606-RA	FBgn0039161	42873	99.86	161.75
1635984_at	Neu3	Meltrin-like	up	1.62	4.77E-02	CG31385-RA	FBgn0051314	3772109	327.95	531.02
1625785_at	l(1)G0196	lethal (1) G0196	up	1.62	4.36E-02	CG14616-RA	FBgn0027279	33137	29.30	47.44
1633830_at	CG10195	---	up	1.62	3.87E-02	CG10195-RA	FBgn0032787	35228	172.25	278.86
1624689_at	---	---	up	1.62	2.39E-02	HDC08982	---	---	106.03	171.61
1631157_at	CG7365	---	up	1.62	2.32E-02	CG7365-RA	FBgn0036939	40200	461.18	746.13
1624571_s_at	CG32500 /// CG32857 /// CG33502	--- /// --- /// ---	up	1.62	6.98E-05	CG32857-RA	/// FBgn0052857 ///	768875 /// 2768879 /// 31825	1299.93	2101.34
1625140_at	NtR	NtR	up	1.62	2.91E-02	CG6698-RA	FBgn0029147	43935	288.99	467.02
1640743_at	Dip-C	Dipeptidase C	up	1.62	5.37E-04	CG5663-RA	FBgn0000455	47769	782.34	1264.01
1626223_at	CG2543	---	up	1.61	9.30E-03	CG2543-RA	FBgn0030407	32212	167.63	270.62
1623762_at	key	kenny	up	1.61	3.18E-02	CG16910-RA	FBgn0041205	37967	1068.19	1723.99
1634016_at	CG2781	---	up	1.61	4.76E-04	CG2781-RA	FBgn0037534	40943	2893.22	4669.36
1629907_at	CG14989	---	up	1.61	3.39E-02	CG14989-RB	FBgn0035495	38485	2975.38	4801.11
1623959_at	CG1532	---	up	1.61	2.87E-02	CG1532-RA	FBgn0031143	33076	819.64	1322.18
1629598_at	FK506-bp1	FK506-binding protein 1	up	1.61	8.24E-03	CG6226-RA	FBgn0013269	41860	218.59	352.21
1632298_s_at	mfas	Midline Fasciclin	up	1.61	9.72E-03	CG3359-RB	FBgn0260745	41455	2064.75	3324.12
1627043_at	CG4674	---	up	1.61	3.02E-03	CG4674-RA	FBgn0037856	41320	275.42	443.35
1641465_at	CG6153	---	up	1.61	1.17E-02	CG6153-RA	FBgn0032445	34675	360.26	579.67
1632390_at	cer	crammer	up	1.61	8.26E-03	CG10460-RA	FBgn0034443	37229	1877.53	3019.98
1635110_at	Cyp6a13	Cyp6a13	up	1.60	1.15E-03	CG2397-RA	FBgn0033304	35837	274.07	439.41
1631487_s_at	CG32626	---	up	1.60	2.16E-02	CT36016	FBgn0052626	32352	123.53	197.84
1628668_at	---	---	up	1.60	6.73E-03	CT33033	---	---	357.94	573.06
1623571_a_at	CG9328	---	up	1.60	1.69E-04	CG9328-RB	FBgn0032886	35344	1781.57	2851.09
1637676_at	CG5482	---	up	1.60	1.20E-03	CG5482-RA	FBgn0034368	37144	1023.50	1637.90
1628644_at	CG17350	---	up	1.60	3.79E-02	CG17350-RA	FBgn0032772	35210	255.10	407.25
1639439_at	Sclp	CG2471	up	1.60	3.18E-02	CG2471-RA	FBgn0030357	32156	13037.80	20805.40
1632743_a_at	dre4	supressor of Ty element 16	up	1.60	2.09E-02	CG1828-RB	FBgn0002183	38248	195.59	312.06
1626981_s_at	CG8979	---	up	1.59	2.15E-02	CG8979-RC	FBgn0033669	36277	600.83	958.21
1638008_s_at	CG2003	---	up	1.59	9.07E-03	CG2003-RA	FBgn0039886	43761	216.27	344.83
1626968_at	CG7207	---	up	1.59	3.34E-02	CG7207-RA	FBgn0027569	38928	1415.25	2255.12
1630919_at	pip	pipe	up	1.59	2.31E-02	CG9614-RA	FBgn0003089	40104	195.14	310.88
1634733_at	CG3831 /// DmirCG3831	--- /// ---	up	1.59	2.24E-02	CG3831-RA	0034804 /// FBgn008	37648	177.69	282.92
1626586_at	Obp57a	Odorant-binding protein 57a	up	1.59	2.38E-02	CG30141-RA	FBgn0043535	246670	682.06	1085.34
1632280_at	mRpS35	mitochondrial ribosomal protein S3	up	1.59	3.62E-04	CG2101-RA	FBgn0035374	38345	443.36	705.25
1641324_at	LanA	Laminin	up	1.59	4.83E-03	CG10236-RA	FBgn0002526	38723	471.33	749.59
1638469_s_at	CG3857	---	up	1.59	1.81E-02	Transposon.36	FBgn0023520	2768908	1337.56	2126.72
1639597_at	Obp44a	Odorant-binding protein 44a	up	1.59	2.89E-02	CG2297-RA	FBgn0033268	35789	18989.71	30163.94
1641400_at	CG9170	---	up	1.59	1.18E-02	CG9170-RA	FBgn0030716	32563	958.31	1522.18
1641446_s_at	CG5171	---	up	1.59	2.78E-04	CG5171-RB	FBgn0031907	34016	1141.52	1812.99
1631571_a_at	spir	spire	up	1.59	1.38E-02	CG10076-RD	FBgn0003475	45931	305.27	484.78
1622999_at	set-01	septin	up	1.59	2.86E-02	CG1403-RA	FBgn0011710	33114	687.09	1091.12
1631726_at	Dscam3	CG31190	up	1.59	4.61E-02	CG31190-RA	FBgn0261046	42103	69.94	111.02
1623298_at	CG6067	---	up	1.59	4.01E-02	CG6067-RA	FBgn0029828	31529	618.41	981.43
1631327_at	mspo	m-spondin	up	1.59	1.59E-03	CG10145-RA	FBgn0020269	36632	635.43	1007.83
1641199_a_at	CG1544	---	up	1.59	3.92E-03	CG1544-RA	FBgn0039827	43689	209.92	332.79
1639748_at	CG15922	---	up	1.58	5.37E-03	CG15922-RA	FBgn0040575	50048	530.52	840.87
1629919_at	Cpr100A	CG12045	up	1.58	1.65E-02	CG12045-RA	FBgn0039805	43657	10917.71	17279.08
1625321_a_at	mtg	mind the gap	up	1.58	2.91E-02	CG7549-RA	FBgn0260386	40970	265.92	420.80
1624463_s_at	pr-set7	pr-set7	up	1.58	1.55E-03	CG3307-RC	FBgn0011474	41743	286.26	452.58
1635413_at	CG32266	---	up	1.58	3.21E-02	CG32266-RA	FBgn0052266	317945	128.23	202.37
1624310_s_at	CG4753	---	up	1.58	2.78E-02	CG4753-RB	FBgn0036622	39819	420.89	664.09
1631671_at	CG13827	---	up	1.58	1.36E-03	CG13827-RA	FBgn0039068	42751	264.29	416.42
1633280_s_at	CG12581	---	up	1.58	8.22E-04	CG12581-RB	FBgn0037213	40522	875.46	1378.98
1641678_at	CG5319	---	up	1.57	2.05E-02	CG5319-RA	FBgn0038501	42089	97.62	153.62
1637517_s_at	KrT95D	Krueppel target at 95D	up	1.57	3.97E-03	CG5405-RB	FBgn0020647	42843	828.42	1302.40
1640159_at	---	---	up	1.57	3.58E-03	CG13377-RA	---	---	991.57	1558.88
1632373_s_at	CG34015	---	up	1.57	2.32E-02	Dm.X.57281.0	FBgn0054015	3885570	346.69	545.01
1623970_at	CG13154	---	up	1.57	5.03E-04	CG13154-RA	FBgn0033736	36359	70.21	110.32
1633477_at	CG10496	---	up	1.57	1.67E-02	CG10496-RA	FBgn0034631	37450	109.88	172.64
1634684_at	CG7272	---	up	1.57	6.87E-03	CG7272-RA	FBgn0036501	39670	1534.76	2410.57
1627749_at	CG6412	---	up	1.57	2.48E-04	CG6412-RA	FBgn0032646	35060	569.38	894.01
1624355_at	bl	bancal	up	1.57	3.61E-03	CG13425-RB	FBgn0015907	43862	1044.47	1639.63
1629023_s_at	CG40191	---	up	1.57	2.37E-02	CG40191-RC	FBgn0058191	3354930	1078.34	1691.90
1630231_at	fw	wrinkled	up	1.57	5.04E-05	CG1500-RA	FBgn0001083	32162	704.96	1104.73
1635732_at	CG6915	---	up	1.57	4.80E-02	CG6915-RA	FBgn0035898	38966	77.24	120.95

1624267_at	CG7182	---	up	1.57	1.16E-02	CG7182-RA	FBgn0035878	38944	157.50	246.60
1637008_at	CG14687	---	up	1.57	2.02E-02	CG14687-RA	FBgn0037835	41297	5181.02	8111.43
1641345_at	CG3808	---	up	1.57	3.63E-02	CG3808-RA	FBgn0036838	40074	83.28	130.33
1641699_at	TXBP181-like	TXBP181-like	up	1.56	3.74E-03	CG2072-RA	FBgn0026326	35954	341.72	534.78
1640983_at	CG5909	---	up	1.56	3.28E-02	CG5909-RA	FBgn0039495	43274	605.82	947.51
1622984_at	Sod2	mitochodnrial SOD	up	1.56	1.88E-04	CG8905-RA	FBgn0010213	36878	2913.71	4555.81
1627693_at	CG1965	---	up	1.56	8.44E-04	CG1965-RA	FBgn0037466	40842	486.47	759.08
1637936_at	CG32512	---	up	1.56	3.01E-02	CG32512-RA	FBgn0052512	33085	175.51	273.80
1629161_at	Obp56f	Odorant-binding protein 56f	up	1.56	4.44E-04	CG30450-RA	FBgn0043533	246668	67.53	105.30
1633701_at	CG16985	---	up	1.56	2.51E-02	CG16985-RA	FBgn0035355	38323	581.70	906.82
1627023_at	CG4884	---	up	1.56	2.67E-02	CG4884-RA	FBgn0039565	43357	220.70	343.83
1627971_s_at	sano	serrano	up	1.56	2.80E-02	CG12758-RB	FBgn0034408	37185	938.97	1461.67
1631620_at	GlyP	glycogen phosphorylase b	up	1.56	1.07E-02	CG7254-RA	FBgn0004507	33386	7291.16	11347.55
1628929_s_at	CG1902	---	up	1.56	7.40E-03	CG1902-RA	FBgn0033434	35993	1376.52	2140.75
1627926_at	CG8315	---	up	1.55	6.31E-03	CG8315-RA	FBgn0034058	36758	479.87	745.75
1633712_at	CG31997	---	up	1.55	1.33E-02	CG31997-RA	FBgn0051997	319064	5289.91	8218.22
1636203_at	B-H1	Bar-H1	up	1.55	2.47E-02	CG5529-RA	FBgn0011758	32724	107.96	167.70
1636745_at	Art4	Arginine methyltransferase 4	up	1.55	2.60E-02	CG5358-RA	FBgn0037770	41219	201.80	313.20
1631948_s_at	Clk	dclock	up	1.55	1.22E-02	CG7391-RA	FBgn0023076	38872	389.13	603.95
1623624_at	CG14869	---	up	1.55	7.90E-03	CG14869-RA	FBgn0038341	41887	613.49	951.77
1638237_at	CG5412	---	up	1.55	2.50E-02	CG5412-RA	FBgn0038806	42434	170.86	265.00
1634767_at	CG6126	---	up	1.55	9.31E-03	CG6126-RA	FBgn0038407	41967	1407.84	2183.30
1634303_at	---	---	up	1.55	3.37E-02	CG11228-RA	---	---	687.82	1065.88
1635456_at	CG31235	---	up	1.55	3.79E-02	CG31235-RA	FBgn0051235	42204	43.76	67.80
1629790_at	CG11267	---	up	1.55	1.52E-03	CG11267-RA	FBgn0036334	39476	2512.19	3887.71
1637076_at	CG8861	---	up	1.55	4.11E-03	CG8861-RA	FBgn0037676	41109	1001.31	1547.44
1626336_at	dnr1	defense repressor 1	up	1.54	3.87E-03	CG12489-RA	FBgn0260866	37575	600.89	925.72
1636423_at	CG8012	---	up	1.54	1.52E-02	CG8012-RA	FBgn0040832	50305	805.70	1240.66
1626350_at	alpha-Est10	Esterase-10	up	1.54	7.44E-04	CG1131-RA	FBgn0015569	40896	246.97	379.92
1628077_at	---	---	up	1.54	5.19E-03	S.C2R000437	---	---	81.71	125.65
1628705_at	PH4alphaEFB	prolyl-4-hydroxylase-alpha EFB	up	1.54	8.77E-03	CG31022-RA	FBgn0039776	43620	1506.16	2314.29
1626760_at	CG2974	---	up	1.54	3.55E-02	CG2974-RA	FBgn0030178	31944	202.69	311.39
1629868_s_at	CG3838	---	up	1.54	1.08E-02	CG3838-RA	FBgn0032130	34275	262.62	403.44
1634191_at	CG6398	---	up	1.54	3.76E-02	CG6398-RA	FBgn0030870	32761	1067.05	1638.79
1637638_s_at	CG10702	---	up	1.53	1.39E-02	CG10702-RB	FBgn0032752	35181	496.89	762.46
1636728_at	Ptr	Ptc-related Disp-like	up	1.53	3.09E-02	CG11212-RA	FBgn0033068	35546	303.87	465.89
1633997_s_at	CG5938	---	up	1.53	7.21E-03	CG5938-RB	FBgn0046247	43290	1891.38	2896.88
1632762_s_at	CG3176 /// CG32817	--- /// ---	up	1.53	4.10E-03	CG3176-RA	0029524 /// FBgn005	30990 /// 318226	249.49	381.73
1626033_at	Atg9	Autophagy-specific gene 9	up	1.53	4.55E-02	CG3615-RA	FBgn0034110	36821	621.31	949.72
1641035_a_at	/// DgriCG5854 /// DmojCG5854 /// Dvir	--- /// --- /// --- /// ---	up	1.53	3.95E-03	CG5854-RA	0086956 /// FBgn008	42831	758.45	1159.19
1634746_at	CG3589	---	up	1.53	2.26E-02	CG3589-RA	FBgn0035065	37966	413.23	630.99
1635272_a_at	CG10205	---	up	1.53	2.22E-02	CG10205-RB	FBgn0033970	36650	1685.06	2572.08
1625725_at	CG42496 /// Ppcdc	--- /// CG30290	up	1.53	1.90E-03	CG30290-RA	0050290 /// FBgn026	246533 /// 8674001	576.46	879.20
1637469_at	pen-2	presenilin enhancer	up	1.52	1.01E-02	CG33198-RA	FBgn0053198	251430	493.57	752.49
1628155_at	klar	klarsicht	up	1.52	1.04E-02	CG17046-RA	FBgn0001316	38067	1114.01	1695.48
1641496_a_at	grass	CG5896	up	1.52	1.82E-02	CG5896-RA	FBgn0039494	43273	1134.69	1726.68
1638044_a_at	CG5902	---	up	1.52	3.72E-04	CG5902-RB	FBgn0039136	42839	1005.17	1528.81
1634704_at	ATP7	ATP7	up	1.52	3.61E-02	CG1886-RA	FBgn0030343	32142	226.52	344.32
1628488_at	CG6051	---	up	1.52	3.87E-02	CG6051-RA	FBgn0039492	43271	579.74	880.45
1626376_at	ptip	CG32133	up	1.52	4.11E-02	CG32133-RA	FBgn0052133	39552	58.15	88.20
1633884_at	Rtf1	CG10955	up	1.52	3.27E-02	CG10955-RA	FBgn0034722	37554	261.86	397.14
1630957_s_at	slif	slimfast	up	1.52	2.92E-02	CG11128-RA	FBgn0037203	40510	541.12	820.69
1627106_a_at	CG10237	---	up	1.52	2.69E-02	CG10237-RA	FBgn0032783	35222	3570.22	5411.66
1623761_at	CG15347	---	up	1.52	3.24E-03	CG15347-RA	FBgn0030040	31779	74.33	112.63
1630797_at	CG32582	---	up	1.51	2.92E-02	CG32582-RA	FBgn0052582	318099	81.18	122.97
1641540_a_at	CG7668	---	up	1.51	8.26E-03	CG7668-RA	FBgn0036929	40190	793.88	1202.02
1626461_at	Hsp60B /// Ncc69	Heat shock protein 60 related /// s	up	1.51	1.61E-02	CG4357-RA	0011244 /// FBgn003	39410 /// 48572	829.02	1252.22
1626338_s_at	vvl	ventral veins lacking	up	1.51	4.54E-02	S.C3L000862	FBgn0086680	38752	57.58	86.97
1624692_at	CG6830	---	up	1.51	4.20E-03	CG6830-RA	FBgn0037934	41408	285.13	430.53
1626646_s_at	CG4729	---	up	1.51	7.09E-04	CG4729-RA	FBgn0036623	39820	2045.95	3086.36
1628795_s_at	CG9175	---	up	1.51	1.46E-02	CG9175-RA	FBgn0031779	33862	199.92	301.50
1630213_at	Fip1	CG1078	up	1.51	4.03E-02	CG1078-RA	FBgn0037255	40569	136.75	206.22
1623481_at	MSBP	CG9066	up	1.51	1.34E-02	CG9066-RA	FBgn0030703	32548	1580.41	2380.50
1625665_at	mRpL39	mitochondrial ribosomal protein L3	up	1.51	1.28E-02	CG17166-RA	FBgn0036462	39627	515.95	777.08
1632309_at	As	Drosophila Angelman syndrome	up	1.51	1.39E-04	CG6190-RA	FBgn0061469	39266	615.17	926.40
1637557_a_at	flr	flare	up	1.51	3.03E-02	CG10724-RA	FBgn0260049	39505	239.25	360.27
1635041_at	CG32225	---	up	1.51	3.77E-02	CG32225-RA	FBgn0052225	317926	555.02	835.69
1632687_at	mib1	Mind Bomb	up	1.51	3.85E-02	CG5841-RA	FBgn0036558	39750	547.15	823.58
1637038_at	tex	tex	up	1.50	3.93E-02	CG9615-RA	FBgn0037569	40983	82.99	124.84
1628953_at	CG7131	---	up	1.50	1.94E-02	CG7131-RA	FBgn0038598	42197	59.81	89.96
1629388_s_at	CG34317 /// CG7950	--- /// ---	up	1.50	1.83E-02	CG7950-RA	0085346 /// FBgn026	43579 /// 5740224	451.15	678.32
1634100_at	CG13865	---	up	1.50	7.29E-03	CG13865-RA	FBgn0039965	3355158	324.50	487.39
1622928_at	Arp5	CG7940	up	1.50	3.13E-02	CG7940-RA	FBgn0038576	42173	157.59	236.66
1636321_s_at	CG13624	---	up	1.50	2.28E-02	CG13624-RB	FBgn0039209	42930	1292.10	1940.14
1629889_s_at	regucalcin	regucalcin	up	1.50	1.85E-02	CG1803-RA	FBgn0030362	32165	2610.95	3919.09
1632699_s_at	Adh /// Adhr	alcohol dehydrogenase /// Adh-rel	up	1.50	2.09E-02	CG32954-RA	0000055 /// FBgn000	3771877 /// 3772432	250.05	375.16
1639834_at	G14291 /// DpseGA12883 /// DvirGJ141	--- /// GA12883 /// CG14291	up	1.50	2.45E-02	CG14291-RA	/// FBgn0064440 ///	42266	196.23	294.40
1634412_at	CG2135	---	up	1.50	3.38E-02	CG2135-RA	FBgn0039874	43746	95.24	142.88
1624057_at	CG16713	---	down	609.05	3.48E-03	CG16713-RA	FBgn0031560	33591	6069.55	9.97
1634366_at	CG32185	---	down	529.45	1.12E-02	CG32185-RA	FBgn0052185	317900	15879.88	29.99
1626088_at	CG2177	---	down	132.10	6.75E-04	CG2177-RA	FBgn0039902	43786	1144.81	8.67
1633386_s_at	mthl8	Mth-like 8	down	106.03	7.20E-03	CT37020	FBgn0052475	38013	351.09	3.31
1627551_s_at	AttA /// AttB	attacin /// attacin	down	59.05	3.03E-03	CG18372-RA	0012042 /// FBgn004	36636 /// 36637	21609.58	365.98
1632719_at	CecC	Cecropin	down	57.10	4.43E-02	CG1373-RA	FBgn0000279	43599	3429.57	60.07
1629362_at	mthl8	Mth-like 8	down	56.94	2.60E-03	CG32475-RA	FBgn0052475	38013	381.76	6.70
1632210_at	CG30091	---	down	51.94	9.11E-03	CG30091-RA	FBgn0050091	246449	180.38	3.47
1625965_a_at	Osi2	Osiris	down	34.69	7.64E-03	CG1148-RB	FBgn0037410	40756	331.99	9.57
1629118_at	PGRP-SC2	PGRP-SC2	down	32.92	3.90E-02	CG14745-RA	FBgn0043575	35862	4398.44	133.61
1637120_at	CG30098	---	down	30.23	1.37E-02	CG30098-RA	FBgn0050098	246456	766.84	25.37
1634633_s_at	---	---	down	29.52	1.30E-04	Transposon.54	---	---	7368.76	249.62
1625124_at	AttA	attacin	down	27.32	3.88E-02	CG10146-RA	FBgn0012042	36636	7069.77	258.81
1634551_at	CG6188	---	down	24.32	1.72E-02	CG6188-RA	FBgn0038074	41561	7625.46	313.61
1641419_at	AttC	attacin	down	23.70	5.24E-03	CG4740-RA	FBgn0041579	36484	32471.41	1369.88
1631512_at	CG9377	---	down	22.28	2.25E-03	CG9377-RA	FBgn0032507	34743	1279.60	57.43
1626526_at	TotF	Turandot F	down	20.90	1.83E-02	CG31691-RA	FBgn0044811	117461	73.60	3.52
1626429_at	Lsp1gamma	Larval serum protein 1 gamma	down	19.95	3.01E-03	CG6821-RA	FBgn0002564	38015	219.40	11.00
1638687_at	CG40298	---	down	17.54	2.15E-03	CG40298-RA	FBgn0058298	3355149	428.93	24.46
1634310_at	TpnC4	TpnC4	down	17.21	2.99E-03	CG12408-RA	FBgn0033027	35498	231.42	13.45
1624819_s_at	---	---	down	15.97	5.08E-03	Transposon.17	---	---	4652.90	291.32
1638235_at	DptB	diptericin-like protein	down	14.24	1.32E-03	CG10794-RA	FBgn0034407	37184	23551.01	1654.31
1633545_at	PGRP-SD	PGRP-SD	down	14.00	3.69E-02	CG7496-RA	FBgn0035806	38858	4972.53	355.13
1626530_at	CecB	Cecropin B	down	12.09	3.02E-03	CG1878-RA	FBgn0000278	43598	1301.93	107.68
1635331_at	Cpr49Af	CG8510	down	11.98	1.05E-02	CG8510-RA	FBgn0033729	36352	200.38	16.73
1636583_at	CG5932	---	down	11.67	1.65E-02	CG5932-RA	FBgn0036996	40267	120.59	10.33
1636490_at	PGRP-SB1	PGRP-SB1	down	10.97	2.68E-02	CG9681-RA	FBgn0043578	39870	19999.70	1823.33
1631693_at	CG9616	---	down	10.47	7.32E-03	CG9616-RA	FBgn0038214	41731	547.69	52.30
1627736_at	Actbeta	activin-beta	down	9.37	2.86E-04	CG11062-RA	FBgn0024913	43826	980.64	104.66
1627613_at	Mtk	Metchnikowin	down	9.23	1.21E-03	CG8175-RA	FBgn0014865	36708	39547.39	4284.80
1638021_at	CG4757	---	down	8.98	1.35E-04	CG4757-RA	FBgn0027584	41330	8600.94	957.94
1633812_at	CG9080	---	down	8.65	4.43E-03	CG9080-RA	FBgn0033593	36183	4028.39	465.86
1634786_at	lectin-28C /// ninaE	Lectin28C /// rhodopsin	down	7.85	7.70E-05	CG7106-RA	0002940 /// FBgn004	42367 /// 53542	1284.60	163.64
1635549_at	TotA	Turandot A	down	7.84	3.15E-02	CG31509-RA	FBgn0028396	44121	23346.16	2978.82
1629194_s_at	ade2	adenosine2	down	7.34	3.92E-03	CG9127-RA	FBgn0000052	33847	3500.15	477.07
1636946_at	CG6687	Dm-serpin-88Eb	down	7.23	2.42E-02	CG6687-RA	FBgn0038299	41829	4500.22	622.56
1628758_at	CG1774	---	down	7.18	1.80E-03	CG1774-RA	FBgn0039856	43722	826.94	115.16
1630633_at	CG13130	---	down	6.68	1.73E-03	CG13130-RA	FBgn0040965	50439	284.61	42.62
1632975_at	CG4398	---	down	6.66	1.82E-04	CG4398-RA	FBgn0034126	36838	167.66	25.17
1626578_at	CG5195	---	down	6.52	4.47E-03	CG5195-RA	FBgn0036995	40266	328.88	50.43
1629125_at	gp210	gp210	down	6.33	7.42E-03	CT34163	FBgn0033039	35512	127.76	20.18
1637061_at	flower	flower	down	6.33	3.03E-03	CG6151-RB	FBgn0261722	---	227.86	36.01
1623813_at	CG33307	---	down	6.32	5.86E-03	CG16887-RA	FBgn0053307	2768914	1673.75	264.64
1628639_at	CG5791	---	down	6.32	5.00E-03	CG5791-RA	FBgn0040582	50055	23273.43	3683.38
1637196_at	CG13237	---	down	6.20	4.15E-02	AT03263	FBgn0031202	---	46.31	7.47
1622906_at	CG9027	---	down	6.14	3.81E-03	CG13200-RA	FBgn0033631	36232	680.24	110.83
1637462_at	CG13833	---	down	5.76	1.84E-02	CG13833-RA	FBgn0039040	42718	1306.41	226.68
1626147_s_at	---	---	down	5.76	2.25E-03	CG12891-RA	---	---	3750.71	651.59
1633208_at	CG11741	---	down	5.72	3.10E-03	CT32625	FBgn0040531	5740784	127.05	22.23
1637734_at	TepI	Thiolester containing protein I	down	5.61	2.06E-03	CG18096-RA	FBgn0041183	34937	98.49	17.55
1637275_a_at	CG13335	---	down	5.58	4.71E-02	CG13335-RA	FBgn0033857	36511	3019.57	541.24
1627119_at	CG8329	---	down	5.52	2.13E-03	CG8329-RA	FBgn0036022	39123	12661.18	2292.86
1625724_at	Pde1c	Phosphodiesterase 1c	down	5.50	1.02E-02	CG14940-RA	FBgn0259225	34594	1120.11	203.82
1639730_at	CG34054	---	down	5.29	3.80E-03	HDC05792	FBgn0054054	3885601	773.52	146.23
1630804_at	Oatp33Eb	Organic anion transporting polype	down	5.28	1.32E-02	CG6417-RA	FBgn0032435	34662	116.59	22.10
1633796_at	CG11425	---	down	5.26	1.26E-04	CG11425-RA	FBgn0037167	40472	3085.42	586.94
1624393_at	w	white	down	5.21	7.00E-03	CG2759-RA	FBgn0003996	31271	106.21	20.38
1633062_at	CG32791	---	down	5.20	9.79E-04	CG32791-RA	FBgn0052791	31322	224.59	43.15
1628806_at	Rh5	Rhodopsin5	down	5.18	8.05E-07	CG5279-RA	FBgn0014019	34615	3173.55	612.14
1631115_at	Obp8a	Odorant-binding protein 8a	down	5.17	2.73E-03	CG12665-RA	FBgn0030103	31860	526.00	101.73
1638424_at	CG10514	---	down	4.84	1.27E-03	CG10514-RA	FBgn0039312	43052	327.04	67.51
1639170_at	Cyp4d21	Cyp4d21	down	4.70	1.01E-02	CG6730-RA	FBgn0031925	34036	1141.68	242.68
1626248_at	CG18547	---	down	4.67	2.44E-02	CG18547-RA	FBgn0037973	41452	622.92	133.44
1629398_at	CG10383	---	down	4.63	3.95E-02	CG10383-RA	FBgn0032699	35123	2384.79	515.55
1627958_at	CG30269	---	down	4.61	2.93E-03	CG30269-RA	FBgn0050269	37600	1547.86	335.88
1629269_at	CG32204	---	down	4.56	3.39E-02	CG32204-RA	FBgn0052204	317913	3868.28	848.11
1635936_at	CG13822	---	down	4.46	4.51E-03	CG13822-RA	FBgn0039098	42787	508.03	113.98
1630065_at	CG6912	---	down	4.45	8.23E-03	CG6912-RA	FBgn0038290	41820	1864.54	419.16
1631808_at	---	---	down	4.43	2.24E-02	LD15354	---	---	56.62	12.78
1627287_at	CG5966	---	down	4.42	1.89E-02	CG5966-RA	FBgn0029831	31532	2072.51	469.35
1630553_at	alphaPS5	alphaPS5	down	4.35	6.05E-03	CG5372-RA	FBgn0034880	37732	207.28	47.68
1637467_at	CG5245 /// DpseGA26228	--- /// ---	down	4.29	9.80E-03	CG5245-RA	0038047 /// FBgn006	41530	72.29	16.87
1632447_at	B52	Serine/arginine rich protein 55	down	4.28	2.02E-04	CG10851-RD	FBgn0004587	41670	219.48	51.33
1632228_at	CG6733	---	down	4.21	9.76E-03	CG6733-RA	FBgn0039052	42731	95.82	22.77
1623364_at	CG4250	---	down	4.18	3.32E-04	CG4250-RA	FBgn0034761	37599	7271.79	1740.30
1639971_at	CG12224	---	down	4.17	2.07E-02	CG12224-RA	FBgn0037974	41453	44.63	10.71
1631165_at	CG4688	---	down	4.17	1.48E-02	CG4688-RA	FBgn0033817	36467	1961.70	470.92
1639224_at	RPA2	CG9273	down	4.16	2.74E-03	CG9273-RA	FBgn0032906	35364	333.86	80.28
1623486_at	CG7900	---	down	4.15	6.04E-03	CG7900-RA	FBgn0037548	40957	250.24	60.36
1634729_at	l(2)37Cd	CG10563	down	4.14	6.53E-03	CG10563-RA	FBgn0086445	35193	245.23	59.24
1637772_at	CG4726	---	down	4.12	5.33E-03	CG4726-RA	FBgn0031307	33292	294.39	71.39
1641280_at	Ance-4	Ance-4	down	4.10	1.07E-03	CG8196-RA	FBgn0033366	35909	1264.35	308.19
1624076_at	CG5849	---	down	4.09	3.78E-04	CG5849-RA	FBgn0038897	42556	275.49	67.33
1635189_at	Drs	drosomycin	down	4.05	5.13E-03	CG10810-RA	FBgn0010381	38419	25324.52	6250.09
1624953_at	Cyp12c1	Cyp12c1	down	4.05	1.53E-04	CG4120-RA	FBgn0036806	40037	935.32	230.94
1639819_at	CG6385	---	down	4.02	2.54E-02	CG6385-RA	FBgn0034276	37026	930.48	231.54
1624947_x_at	---	---	down	4.00	1.66E-02	CT35233	---	---	43.16	10.80
1638969_at	CG6279	---	down	3.97	4.64E-02	CG6279-RA	FBgn0036125	39242	141.43	35.58
1636007_at	Ance-2	Ance-2	down	3.83	1.01E-02	CG16869-RA	FBgn0032535	34806	150.65	39.30
1631225_at	CG8317	---	down	3.82	2.36E-04	CG8317-RA	FBgn0034140	36853	4075.90	1068.34
1628757_at	Glut1	glucose transporter 1	down	3.81	6.57E-03	CG1086-RA	FBgn0025593	38109	613.12	161.13
1627784_at	---	---	down	3.77	3.17E-03	GM07606	---	---	636.39	168.96
1628174_at	nimB1	CG33119	down	3.77	1.34E-03	CG33119-RA	FBgn0027929	34813	237.12	62.98
1635283_at	nimB2	CG31839	down	3.74	7.63E-03	CG31839-RA	FBgn0028543	260645	3149.02	841.51
1637432_at	CG3097	---	down	3.74	2.21E-02	CG3097-RA	FBgn0029804	31503	1068.10	285.52
1639180_at	Arc1	CG12505	down	3.73	3.23E-04	CG12505-RA	FBgn0033926	36595	10693.08	2865.25
1630913_at	Shroom	CG13942	down	3.72	9.74E-03	CT33491	FBgn0085408	36592	78.64	21.16
1636213_at	---	---	down	3.72	1.74E-02	RE73276	---	---	168.83	45.43
1633237_at	Idgf1	Imaginal disc growth factor1	down	3.69	1.80E-03	CG4472-RA	FBgn0020416	34978	2691.48	729.58
1630520_s_at	---	---	down	3.68	2.15E-04	RH09485	---	---	3813.19	1035.62
1629784_a_at	Myo28B1	myosin VII 28B1-B4	down	3.67	5.84E-03	CG6976-RC	FBgn0040299	53515	315.70	85.99

1639424_at	CG6910	---	down	3.67	1.81E-02	CG6910-RA	FBgn0036262	39391	1670.66	455.47
1623852_at	CG10407	---	down	3.66	1.69E-02	CG10407-RA	FBgn0038395	41949	1240.13	338.81
1623770_at	CG1673	---	down	3.66	1.58E-02	CG1673-RA	FBgn0030482	32297	3736.75	1021.13
1639374_s_at	Amy-d /// Amy-p	Amylase /// Amylase	down	3.66	3.35E-02	CG18730-RA	0000078 /// FBgn000	36932 /// 47764	1461.98	399.67
1638238_at	CG5778	---	down	3.66	3.00E-03	CG5778-RA	FBgn0038930	42598	20198.19	5521.95
1633727_s_at	wnd	CG8789	down	3.63	5.71E-04	CG8789-RA	FBgn0036896	40143	1445.68	398.08
1631521_at	CG33469	---	down	3.62	8.09E-03	CG33469-RA	FBgn0053469	2768832	57.38	15.85
1627489_a_at	CG10433	---	down	3.61	2.92E-03	CG10433-RA	FBgn0034638	37458	8755.86	2428.61
1628884_at	PGRP-SA	semmelweis	down	3.60	4.03E-02	CG11709-RA	FBgn0030310	32099	2044.97	568.22
1638330_at	Dot	Dorothy	down	3.57	2.36E-03	CG2788-RA	FBgn0015663	44058	276.82	77.47
1640144_at	CG18067	---	down	3.54	1.53E-03	CG18067-RA	FBgn0034512	37314	37327.24	10546.43
1640553_at	CG13063	---	down	3.53	3.38E-02	CG13063-RA	FBgn0036601	39797	279.82	79.29
1635188_at	CG6675	---	down	3.53	2.13E-02	CG6675-RA	FBgn0032973	35439	2292.72	650.21
1639694_s_at	Arc1 /// CG10102	CG12505 /// ---	down	3.52	8.59E-04	CG10102-RA	0033926 /// FBgn003	36595 /// 36596	535.74	152.36
1626328_at	CG32259	---	down	3.51	2.05E-02	CG32259-RB	FBgn0052259	317942	68.42	19.48
1632392_s_at	Slob	giant slob	down	3.44	1.76E-03	CG6772-RB	FBgn0024290	34039	2604.78	756.38
1624836_at	CG13795 /// DsecCG13795	--- /// ---	down	3.44	4.12E-03	CG13795-RA	0031937 /// FBgn026	34049	2217.14	644.76
1627271_at	CG13422 /// CG7655	--- /// ---	down	3.40	5.66E-04	CG13422-RA	0034511 /// FBgn003	37313 /// 42128	22408.94	6599.45
1639896_at	---	---	down	3.39	4.19E-02	HDC10707	---	---	182.39	53.87
1624849_at	Prosap	Prosap	down	3.37	5.18E-05	CG30483-RA	FBgn0040752	50225	2149.08	637.10
1625697_at	Cyp6g1	CYP6-like	down	3.37	1.14E-03	CG8453-RA	FBgn0025454	36316	3430.94	1019.30
1632964_at	pirk	CG15678	down	3.36	1.87E-02	CG15678-RA	FBgn0034647	37468	455.21	135.42
1634256_a_at	CG11566 /// stg1	--- /// CG33670	down	3.33	3.36E-02	CG32514-RA	0031159 /// FBgn006	318064 /// 3772393	187.18	56.16
1630749_at	CG11052	---	down	3.31	2.67E-03	CG11052-RA	FBgn0040524	49997	224.89	67.88
1631627_at	CG5335 /// Fbp1	--- /// Protein-1	down	3.28	2.63E-03	CG5335-RA	0000639 /// FBgn003	37140 /// 39566	474.72	144.88
1640757_at	CG33462	---	down	3.27	2.65E-03	CG30086-RA	FBgn0053462	2768841	127.24	38.86
1631370_at	CG30026	---	down	3.27	8.61E-04	CG30026-RA	FBgn0050026	246399	1974.11	604.51
1629013_at	CG11436	---	down	3.25	9.97E-04	CG11436-RA	FBgn0029713	31386	285.81	88.01
1639866_at	---	---	down	3.24	3.67E-02	S.C3L002717	---	---	78.14	24.14
1637370_at	yellow-f	yellow f	down	3.23	2.71E-02	CG18550-RA	FBgn0041710	41595	330.87	102.28
1635937_at	CG4500 /// DsecCG4500	--- /// ---	down	3.23	2.03E-02	CG4500-RA	0028519 /// FBgn026	34822	283.47	87.75
1640540_at	CG30273	---	down	3.23	3.10E-02	CG30273-RA	FBgn0050273	246520	405.27	125.54
1630067_a_at	TepII	Thiolester containing protein II	down	3.20	2.14E-02	CG7052-RA	FBgn0041182	34044	6349.49	1982.09
1624269_at	gkt	glaikit	down	3.20	4.45E-03	CG8825-RA	FBgn0260817	33530	246.14	76.99
1640778_at	---	---	down	3.20	6.81E-03	LP09838	---	---	438.22	137.10
1637037_at	ade3	Complementation group D	down	3.18	1.90E-03	CG31628-RA	FBgn0000053	33986	3735.65	1173.62
1631215_at	CG32132	---	down	3.17	1.68E-03	CG32132-RA	FBgn0052132	317877	553.49	174.45
1627652_at	CG42492	CG11473	down	3.17	1.05E-04	CG17761-RA	FBgn0259994	8674066	169.28	53.48
1630561_at	CG13641	---	down	3.14	5.60E-04	CG13641-RA	FBgn0039239	42969	766.26	243.71
1636682_at	CG31778	---	down	3.14	9.08E-04	CG31778-RA	FBgn0051778	261616	1341.09	427.23
1631082_at	B52	Serine/arginine rich protein 55	down	3.13	1.37E-02	CG10851-RF	FBgn0004587	41670	103.31	32.98
1632430_at	Tsf1	Transferrin 1	down	3.12	6.59E-03	CG6186-RA	FBgn0022355	32821	19803.95	6348.19
1639502_at	nimC1	nimrod C1	down	3.08	8.80E-03	CG8942-RA	FBgn0259896	34816	168.73	54.78
1634690_at	tum	tumbleweed	down	3.07	3.72E-02	CG13345-RA	FBgn0086356	36538	157.73	51.40
1631760_at	CG7768	---	down	3.04	2.86E-02	CG7768-RB	FBgn0036415	39573	106.32	34.92
1633921_at	CG30050	---	down	3.03	1.11E-03	CG30050-RA	FBgn0050050	246417	604.73	199.36
1640377_s_at	Rala	anon-fast-evolving-1H4	down	3.03	6.18E-03	CG2849-RB	FBgn0015286	31332	896.16	296.16
1639442_a_at	Tsf1	Transferrin 1	down	3.01	3.73E-02	CG6186-RB	FBgn0022355	32821	1697.06	563.39
1626492_s_at	Sop2	Suppressor of profilin 2	down	3.01	5.20E-04	CG8978-RB	FBgn0001961	34793	1560.80	518.88
1630255_at	CG31676	---	down	3.00	5.83E-06	CG31676-RA	FBgn0051676	35351	1496.48	499.59
1638302_a_at	CG1090	---	down	3.00	3.33E-02	CG1090-RA	FBgn0037238	40551	577.94	192.96
1628942_at	CG15385	---	down	2.97	2.77E-02	CG15385-RA	FBgn0031397	33396	305.90	103.00
1635748_at	CG15322	---	down	2.94	3.58E-02	CG15322-RA	FBgn0031088	33012	52.30	17.79
1631468_a_at	tim	TIMELESS	down	2.92	4.17E-03	CG3234-RC	FBgn0014396	33571	126.90	43.41
1625139_at	Gadd45	Gadd45	down	2.91	8.46E-03	CG11086-RA	FBgn0033153	35646	270.30	92.80
1630825_at	CG5798	---	down	2.91	1.04E-03	CG5798-RA	FBgn0038862	42509	2383.73	820.27
1637662_at	msta	msta	down	2.90	9.87E-03	CG32800-RA	FBgn0053548	31200	4524.56	1560.14
1627735_at	CG13908	---	down	2.89	2.28E-02	CG13908-RA	FBgn0035178	38110	869.44	300.93
1640775_a_at	Nmdmc	NAD-dependent methylenetetrahy	down	2.88	3.09E-05	CG18466-RB	FBgn0010222	47895	3247.72	1125.74
1636293_at	CG2217	---	down	2.87	6.73E-03	CG2217-RA	FBgn0027544	43607	3850.12	1343.27
1638611_at	---	---	down	2.86	1.37E-02	RE24931	---	---	169.63	59.35
1633826_at	Iris	Iris	down	2.84	2.66E-03	CG4715-RA	FBgn0031305	33290	1391.64	489.89
1630524_at	---	---	down	2.84	2.60E-02	LP09564	---	---	1249.63	440.32
1631604_at	CG42369	---	down	2.83	3.50E-03	CG9511-RA	FBgn0259715	33894	3659.55	1291.63
1637516_at	CG15449	---	down	2.81	1.31E-02	CG15449-RA	FBgn0031146	33079	159.82	56.89
1627376_at	Rel	relish	down	2.81	2.80E-02	CG11992-RA	FBgn0014018	41087	4245.26	1511.38
1641657_s_at	CG12424	---	down	2.80	3.79E-02	CG12424-RC	FBgn0033987	36673	924.68	330.45
1625698_at	CG6639 /// RSG7	--- /// Regulator of G-protein signa	down	2.79	2.23E-02	CG6639-RA	0024941 /// FBgn003	32674 /// 35049	600.48	214.89
1641062_at	Ady43A	Ady43A	down	2.79	4.20E-04	CG1851-RA	FBgn0026602	44750	539.23	193.36
1629678_a_at	Aplip1	APP-like protein interacting protei	down	2.78	3.38E-03	CG1200-RA	FBgn0040281	53472	1520.00	545.99
1641729_at	CG13043	---	down	2.78	4.93E-03	CG13043-RA	FBgn0036600	39796	3832.87	1378.97
1627436_s_at	Pdp1	PAR-domain protein 1	down	2.78	3.91E-03	CG17888-RF	FBgn0016694	45588	4585.46	1650.91
1630088_at	CG16743	---	down	2.77	1.23E-03	CG16743-RA	FBgn0032322	34517	1758.78	634.59
1626789_at	CG13618	---	down	2.77	5.21E-03	CG13618-RA	FBgn0039203	42924	296.25	106.94
1629569_at	Fbxl4	CG1839	down	2.75	9.93E-04	CG1839-RA	FBgn0030555	32378	1549.49	562.85
1641382_at	Pms2	Pms2	down	2.75	4.57E-03	CG8169-RA	FBgn0011660	36705	122.42	44.56
1633220_at	CG16739	---	down	2.74	3.72E-02	CG16739-RA	FBgn0034505	37307	52.92	19.31
1638645_at	CG9662	---	down	2.74	1.70E-04	CG9662-RA	FBgn0031529	33554	314.34	114.73
1638636_at	CG12512	---	down	2.70	1.50E-02	CG12512-RA	FBgn0031703	33766	715.59	264.92
1636073_at	CG32344	---	down	2.68	1.30E-02	CG32344-RA	FBgn0052344	326208	196.14	73.15
1631748_at	---	---	down	2.68	4.70E-02	HDC17718	---	---	46.68	17.44
1638581_at	Ubqn	CG14224	down	2.67	7.79E-03	CG14224-RA	FBgn0031057	32977	1385.31	518.00
1634158_at	Npc2f	CG6164	down	2.64	7.02E-03	CG6164-RA	FBgn0039154	42864	106.46	40.38
1631392_at	CG11089	---	down	2.64	3.75E-04	CG11089-RA	FBgn0039241	42973	13690.52	5193.86
1634188_a_at	tim	TIMELESS	down	2.63	8.63E-04	CG3234-RA	FBgn0014396	33571	5019.62	1908.52
1629083_at	CG16704	---	down	2.60	1.00E-02	CG16704-RA	FBgn0031558	33589	12410.33	4768.88
1635273_s_at	kek5	kekkon-like	down	2.60	1.38E-04	CG12199-RC	FBgn0031016	32930	580.43	223.12
1623355_at	v	vermilion	down	2.60	2.45E-03	CG2155-RA	FBgn0003965	32026	1442.52	554.86
1628560_at	CG9344	---	down	2.60	1.14E-02	CG9344-RA	FBgn0034564	37372	376.23	144.87
1636145_at	Spn28D	Dm-serpin-28D	down	2.59	1.51E-02	CG7219-RA	FBgn0031973	34091	2046.55	789.05
1630619_at	Spn1	serpin 1	down	2.58	5.67E-03	CG9456-RA	FBgn0028988	49808	1945.51	752.85
1623068_at	Cyp4e3	Cytochrome P450-4e3	down	2.58	4.14E-03	CG4105-RA	FBgn0015035	34291	3709.52	1436.37
1623871_at	CG18563	---	down	2.58	2.34E-02	CG18563-RA	FBgn0032639	35050	264.46	102.42
1629469_s_at	CG10960	---	down	2.58	1.67E-03	CG10960-RC	FBgn0036316	39458	4987.95	1936.77
1636071_a_at	Cbp80	cap binding protein 80	down	2.57	1.98E-02	CG7035-RA	FBgn0022942	44409	357.30	138.89
1627594_at	---	---	down	2.57	3.89E-02	HDC07873	---	---	36.59	14.26
1628296_at	pgant4	polypeptide GalNAc transferase 4	down	2.56	5.44E-04	CG31956-RA	FBgn0051956	261610	49.94	19.49
1626611_at	---	---	down	2.56	3.54E-02	S.C2L001001	---	---	34.68	13.57
1633493_at	---	---	down	2.55	4.33E-02	S.CX001836	---	---	53.69	21.02
1628492_at	Aprt	Adenine phosphoribosyltransferas	down	2.55	5.57E-04	CG18315-RA	FBgn0000109	48224	864.01	338.42
1631962_at	MtnD	Metallothionein D	down	2.55	2.93E-02	CG33192-RA	FBgn0053192	326270	295.05	115.71
1640754_at	bmm	CG5295	down	2.55	3.14E-03	CG5295-RA	FBgn0036449	39611	4597.89	1806.57
1638452_at	per	period	down	2.54	4.82E-02	CG2647-RA	FBgn0003068	31251	492.82	194.20
1635507_at	CG30031 /// CG4269	--- /// ---	down	2.54	2.27E-02	CG4269-RA	0034741 /// FBgn005	246404 /// 37578	984.34	388.03
1634805_at	CG14642	---	down	2.53	1.70E-02	CG14642-RA	FBgn0037222	40532	97.33	38.53
1624101_at	Cyp6a23	Cyp6a23	down	2.51	6.58E-04	CG10242-RA	FBgn0033978	36661	744.36	296.29
1624736_a_at	CG5819	---	down	2.51	2.21E-04	CG5819-RA	FBgn0034717	37547	192.97	77.01
1629747_at	Cpr49Ag	CG8511	down	2.50	1.33E-04	CG8511-RA	FBgn0033730	36353	6382.05	2551.91
1627912_at	CG5493	---	down	2.50	1.49E-03	CG5493-RA	FBgn0034364	37139	2930.12	1174.10
1623951_at	---	---	down	2.48	6.06E-03	CG14068-RA	---	---	146.11	59.01
1626605_at	CG14075	---	down	2.47	3.38E-02	CG14075-RA	FBgn0036835	40071	121.67	49.23
1624466_a_at	CG15117	---	down	2.47	1.75E-02	CG15117-RA	FBgn0034417	37197	449.52	182.07
1628931_at	CG3301	---	down	2.46	5.85E-03	CG3301-RA	FBgn0038878	42528	545.17	221.59
1632639_at	Arc2	CG13941	down	2.46	4.77E-02	CG13941-RA	FBgn0033928	36597	183.84	74.83
1625052_at	CG5955	---	down	2.46	4.54E-02	CG5955-RA	FBgn0036997	40268	1351.21	550.38
1632209_at	CG7829	---	down	2.45	7.99E-03	CG7829-RA	FBgn0039703	43522	952.67	388.37
1641434_at	CG32695	---	down	2.45	1.64E-03	CG32695-RA	FBgn0052695	31918	2487.38	1017.08
1634438_at	Pph13	Munster	down	2.43	4.60E-04	CG2819-RA	FBgn0023489	33239	783.51	321.86
1641539_a_at	CG7829	---	down	2.43	3.94E-03	CG7829-RB	FBgn0039703	43522	1827.86	752.81
1639530_at	CG14528	---	down	2.43	1.92E-02	CG14528-RA	FBgn0039611	43410	362.80	149.48
1623415_at	dl	Dorsal	down	2.43	1.33E-03	CG6667-RC	FBgn0260632	35047	828.93	341.61
1637658_at	mthl3	mth-like 3	down	2.42	3.42E-02	CG6530-RA	FBgn0028956	36961	50.16	20.68
1634143_at	Cyp6w1	Cyp6w1	down	2.41	2.56E-02	CG8345-RA	FBgn0033065	35543	3084.72	1277.41
1641570_s_at	CG18858 /// CG31683	--- /// ---	down	2.40	3.99E-05	CG31683-RA	0042175 /// FBgn005	261623 /// 326109	3529.30	1468.45
1633619_at	---	---	down	2.40	1.78E-02	GH12056	---	---	118.99	49.66
1631246_at	Fmrf	FMRFamide	down	2.40	4.54E-02	CG2346-RA	FBgn0000715	36030	365.27	152.46
1641415_at	---	---	down	2.39	3.01E-03	RH47344	---	---	92.34	38.57
1632478_a_at	Pkc53E	protein kinase C	down	2.39	3.42E-03	CG6622-RB	FBgn0003091	48311	1897.81	793.84
1633825_at	CG14221	---	down	2.38	6.60E-03	CG14221-RA	FBgn0031042	32961	130.59	54.77
1640521_at	CG6361	---	down	2.37	8.82E-04	CG6361-RA	FBgn0030925	32831	889.85	375.02
1636148_at	CG6553	---	down	2.36	2.08E-02	CG6553-RA	FBgn0033880	36537	173.81	73.52
1626345_at	IM4	Immune induced molecule 4	down	2.36	1.95E-03	CG15231-RA	FBgn0040653	50126	25186.23	10676.04
1637862_at	CG6654	---	down	2.35	2.59E-04	CG6654-RA	FBgn0038301	41831	578.48	245.94
1639397_at	CG9676 /// spheroide	--- /// CG9675	down	2.35	3.22E-02	CG9676-RA	0030773 /// FBgn003	32647 /// 32648	5916.70	2521.45
1627529_at	CG32726	---	down	2.33	3.99E-03	CG32726-RA	FBgn0052726	318175	1212.40	519.76
1623080_at	CG18410	---	down	2.32	1.30E-03	CG18410-RA	FBgn0039226	42953	1058.95	456.69
1637800_at	CG3347	---	down	2.31	4.29E-02	CG3347-RA	FBgn0031513	33538	79.49	34.35
1640594_at	CG3011 /// DsecCG3011	--- /// ---	down	2.31	6.53E-04	CG3011-RA	0029823 /// FBgn026	31524	5284.52	2287.84
1623932_at	Ent3	Equilibrative nucleoside transporte	down	2.30	3.76E-04	CG11010-RA	FBgn0036319	39461	1112.11	483.21
1626665_at	CG7408	---	down	2.30	4.08E-02	CG7408-RB	FBgn0036765	39991	132.04	57.46
1628272_a_at	CG12466 /// CG40486	--- /// ---	down	2.29	1.13E-03	CG40486-RB	0031206 /// FBgn006	3355162	1793.18	781.91
1623900_a_at	CG14935 /// DsecCG14935	--- /// ---	down	2.29	9.82E-03	CG14935-RA	0032382 /// FBgn026	34598	2766.28	1206.62
1637439_at	---	---	down	2.29	2.42E-02	CG14709-RA	---	---	77.18	33.68
1630365_at	CG33330 /// CG3563	--- /// ---	down	2.29	1.08E-02	CG3563-RA	0053330 /// FBgn026	2768667 /// 41788	220.23	96.35
1632087_at	CG9928	---	down	2.28	2.51E-03	CG9928-RA	FBgn0032472	34704	13456.85	5896.76
1634048_a_at	bgm	bubblegum	down	2.28	1.71E-02	CG4501-RB	FBgn0027348	44117	2268.40	994.15
1628264_a_at	RecQ5	Drosophila homolog of RecQ	down	2.28	1.34E-02	CG4879-RA	FBgn0027375	39594	348.90	153.33
1636217_at	CG30503 /// kappaB-Ras	--- /// kappaB-Ras	down	2.27	5.62E-03	CG30503-RA	0040513 /// FBgn005	246656 /// 35667	1846.04	813.13
1630844_at	CG15293	---	down	2.26	6.45E-03	CG15293-RA	FBgn0028526	34828	12527.89	5539.25
1633293_at	CG10157	---	down	2.26	2.69E-02	CG10157-RA	FBgn0039099	42788	3556.57	1573.12
1638325_at	Hn	Henna	down	2.26	1.86E-02	CG7399-RA	FBgn0001208	38871	13143.79	5818.79
1633936_a_at	sca	scabrous	down	2.25	1.98E-03	CG17579-RA	FBgn0003326	36411	175.64	77.91
1628963_at	CG4716 /// DsimGD10957	--- /// CG4716	down	2.24	3.10E-03	CG4716-RA	0033820 /// FBgn006	36471	15458.02	6898.18
1627251_s_at	CG42343	CG12564	down	2.24	1.19E-02	CG12565-RA	FBgn0259245	33125	51.83	23.16
1640488_a_at	CG9510 /// CG9515	--- /// ---	down	2.23	4.80E-02	CG33085-RA	0032076 /// FBgn003	3771738 /// 3771965	2924.12	1308.77
1631523_at	CG10824	---	down	2.23	6.05E-03	CG10824-RA	FBgn0038865	42512	626.86	281.24
1638078_at	Side	similar to Deadpan	down	2.23	2.93E-02	CG10446-RA	FBgn0032741	35168	845.02	379.37
1632772_at	CG15203	---	down	2.22	4.85E-03	CG15203-RA	FBgn0030261	32038	446.27	200.58
1627086_at	CG14871	---	down	2.22	5.37E-03	CG14871-RA	FBgn0038343	41890	557.92	251.68
1639019_s_at	CG33470 /// IM10	--- /// Immune induced molecule 1	down	2.22	4.05E-03	Dm.2R.12921.0	0033835 /// FBgn005	2768831 /// 36486	25738.64	11615.07
1633815_at	CG31191	---	down	2.21	3.32E-03	CG31191-RA	FBgn0051191	42477	1216.30	549.30
1636333_a_at	mthl2	Mth-like 2	down	2.21	2.02E-02	CG17795-RA	FBgn0035623	38636	176.33	79.76
1639079_at	Adgf-D	Adenosine deaminase-related gro	down	2.21	5.25E-05	CG9621-RA	FBgn0038172	41679	2140.90	969.70
1631382_at	CG13085	---	down	2.20	8.08E-03	CG13085-RA	FBgn0032780	35219	112.66	51.27
1628101_s_at	CG32581 /// CG8974	--- /// ---	down	2.20	1.14E-04	CG32581-RA	0030693 /// FBgn005	318098 /// 32532	4361.61	1985.36
1638305_at	Mip	drostatin-B2	down	2.19	2.61E-03	CG6456-RA	FBgn0036713	39933	905.65	412.99
1640199_at	Hcs	CG14670	down	2.19	4.08E-03	CG14670-RA	FBgn0037332	40659	758.27	345.78
1626641_s_at	glob1	Hemoglobin	down	2.19	1.79E-04	CG9734-RC	FBgn0027657	41930	11562.38	5276.42
1638366_at	---	---	down	2.19	1.59E-02	HDC20114	---	---	261.90	119.77
1632431_s_at	Ance	anon-fast-evolving-3D10	down	2.19	1.51E-04	CG8827-RA	FBgn0012037	34805	2030.71	929.09
1629046_a_at	CG4716 /// DsimGD10957	--- /// CG4716	down	2.18	4.13E-03	CG4716-RB	0033820 /// FBgn006	36471	10248.18	4699.37
1638862_at	CG15553	---	down	2.18	2.18E-02	CG15553-RA	FBgn0039817	43677	96.05	44.07
1631606_at	CG14285	---	down	2.18	7.72E-03	CG14285-RA	FBgn0038674	42281	181.24	83.26
1638414_a_at	Bmcp	Bmcp	down	2.15	9.89E-03	CG7314-RA	FBgn0036199	39322	953.60	443.21
1634406_at	CG32982	---	down	2.14	3.16E-03	CG32982-RB	FBgn0052982	34237	289.43	135.12
1637872_at	Spat	serine pyruvate aminotransferase	down	2.14	1.41E-03	CG3926-RA	FBgn0014031	31587	2121.94	991.67
1633905_at	RpL28	Ribosomal protein L28	down	2.14	8.84E-04	CG12740-RC	FBgn0035422	38397	189.65	88.69
1634106_at	CG11686	---	down	2.14	1.11E-05	CG11686-RA	FBgn0040551	50024	1557.46	728.62
1623327_at	---	---	down	2.13	9.04E-03	S.C2R001709	---	---	4201.17	1969.51

1628986_at	gp210	gp210	down	2.13	1.70E-02	CG7897-RA	FBgn0033039	35512	603.71	283.29
1635558_s_at	CG17724 /// seq	--- /// sequoia	down	2.12	2.08E-03	LD12453	0028991 /// FBgn003	246682 /// 3772396	579.35	272.83
1636762_a_at	scf	supercoiling factor	down	2.12	2.31E-02	CG9148-RA	FBgn0025682	38145	1579.48	744.48
1625856_at	dm	diminutive	down	2.12	1.89E-04	CG10798-RA	FBgn0000472	31310	1899.89	897.35
1635191_at	Tsp42Ed	tetraspanin 42E	down	2.11	2.81E-03	CG12846-RA	FBgn0029507	35613	1169.17	552.85
1638675_at	CG10680	---	down	2.11	1.81E-03	CG10680-RA	FBgn0032836	35282	7349.01	3479.55
1627007_at	CG4306	---	down	2.11	1.82E-02	CG4306-RA	FBgn0036787	40017	750.41	356.22
1632980_at	CG3902	---	down	2.10	5.99E-03	CG3902-RA	FBgn0036824	40059	4562.31	2168.92
1634237_at	nimC3	lethal (2) 34Fa	down	2.10	1.43E-02	CG16880-RA	FBgn0001967	48772	156.49	74.42
1625954_at	CG2681	---	down	2.10	2.86E-02	CG2681-RA	FBgn0024997	31258	307.09	146.35
1639273_s_at	vri	vrille	down	2.10	2.41E-04	CG14029-RC	FBgn0016076	33759	2488.53	1186.28
1631040_at	sowah	sosondowah	down	2.10	1.38E-03	CG10632-RA	FBgn0036302	39438	616.32	293.82
1623459_at	Spn4	neuroserpin	down	2.09	2.69E-02	CG9453-RC	FBgn0028985	49805	1181.48	564.45
1636407_at	gol	goliath	down	2.09	2.07E-03	CG2679-RB	FBgn0004919	38006	3713.87	1776.02
1637422_at	CG3246	---	down	2.09	2.61E-03	CG3246-RA	FBgn0031538	33564	1886.71	903.35
1627915_a_at	Gapdh2	G3-P dehydrogenase	down	2.08	1.12E-02	CG8893-RA	FBgn0001092	32545	28732.43	13795.62
1633254_at	CG7787	---	down	2.08	5.74E-03	CG7787-RA	FBgn0032020	34142	559.74	268.81
1627191_a_at	ena	enabled	down	2.07	6.87E-03	CG15112-RC	FBgn0000578	37201	691.83	333.98
1628572_s_at	stops	suppressor (rdgB) 69	down	2.06	5.51E-03	CG31006-RB	FBgn0086704	43683	2482.12	1203.33
1636210_at	CG10191	---	down	2.06	1.32E-02	CG10191-RA	FBgn0036354	39502	70.59	34.26
1632437_at	CR31846	---	down	2.06	4.23E-02	CG31846-RA	FBgn0051846	318979	630.58	306.16
1630468_at	CG15247	---	down	2.06	3.31E-02	CG15247-RA	FBgn0030156	31919	113.82	55.32
1626416_a_at	l(2)efl	lethal (2) essential for life	down	2.05	1.47E-05	CG4533-RB	FBgn0011296	37744	6398.84	3116.64
1628847_a_at	CG17167	---	down	2.05	4.16E-02	CG40146-RA	FBgn0039941	3354991	174.08	85.01
1641071_at	CG8343	Incilarin-like	down	2.05	7.60E-03	CG8343-RA	FBgn0040502	35544	25905.78	12662.54
1631534_at	sfl	diet-wingless	down	2.04	1.01E-02	CG8339-RA	FBgn0020251	38736	214.79	105.13
1636974_at	dlp	Dally-like	down	2.04	3.05E-04	CG32146-RA	FBgn0041604	39596	1425.73	698.72
1640379_s_at	CG34424	---	down	2.04	2.53E-02	CG11079-RA	FBgn0085453	37699	1245.78	611.26
1637491_s_at	CG3835	---	down	2.04	8.98E-03	CG3835-RB	FBgn0023507	31184	2962.31	1453.94
1639654_at	CG3603	---	down	2.02	2.72E-02	CG3603-RA	FBgn0029648	31289	529.68	262.22
1624373_at	Crg-1	Circadianly Regulated Gene	down	2.01	1.00E-02	CG32788-RB	FBgn0021738	43900	229.34	113.85
1626888_at	CG9426	---	down	2.01	6.74E-03	CG9426-RA	FBgn0032485	34719	866.46	430.26
1631228_a_at	sPLA2	secretory Phospholipase A2	down	2.01	4.95E-02	CG11124-RA	FBgn0033170	35665	917.12	456.56
1634184_a_at	jp	junctophilin	down	2.01	4.15E-02	CG4405-RB	FBgn0032129	34274	589.00	293.30
1640206_at	CG4660	---	down	2.00	2.23E-03	CG4660-RA	FBgn0029839	31542	1177.40	587.34
1631127_at	CG10424	---	down	2.00	2.91E-02	CG10424-RA	FBgn0036848	40085	378.50	188.90
1633963_a_at	nAcRalpha-30D	Dalpha6	down	2.00	1.36E-03	CG4128-RA	FBgn0032151	34304	2527.28	1266.12
1632100_s_at	sina	anon-fast-evolving-2H7	down	2.00	8.57E-03	CG9949-RA	FBgn0003410	39884	1089.75	546.16
1634436_at	Ast-C	drostatin-C	down	1.98	1.53E-03	CG14919-RA	FBgn0032336	34537	3667.62	1854.87
1633191_at	CG7650	---	down	1.96	4.00E-02	CR_tc_AT27057	FBgn0036519	39694	125.66	63.97
1625613_at	CG11899 /// DsimGD21426	--- /// CG11899	down	1.96	2.91E-03	CG11899-RA	0014427 /// FBgn008	46391	3229.48	1644.55
1640906_at	CG32523 /// DsecCG32523	--- /// ---	down	1.96	1.53E-02	CG32523-RA	0052523 /// FBgn026	318071	1951.42	994.50
1636994_at	CG14712	---	down	1.96	2.15E-02	CG14712-RA	FBgn0037924	41397	202.65	103.35
1640590_at	CG32195	---	down	1.96	2.12E-02	CG32195-RA	FBgn0052195	317907	307.01	156.57
1628173_at	CG17189	---	down	1.96	2.66E-02	CG17189-RA	FBgn0039485	43263	1003.82	512.21
1624595_a_at	Rbp2	anon-fast-evolving-1B9	down	1.96	5.85E-03	CG4429-RA	FBgn0010256	49809	326.28	166.85
1627752_s_at	SK	small conductance calcium-activat	down	1.96	6.37E-04	CG10706-RA	FBgn0029761	31456	1372.30	701.94
1634702_at	CG7227	---	down	1.95	2.35E-02	CG7227-RA	FBgn0031970	34088	446.96	229.23
1641343_at	Crz	corazonin	down	1.95	3.70E-03	CG3302-RA	FBgn0013767	41742	893.28	458.25
1636174_at	GstD9	Glutathione S transferase D9	down	1.95	6.29E-03	CG10091-RA	FBgn0038020	41502	957.96	491.50
1631110_at	CG10257	---	down	1.95	4.89E-03	CG10257-RA	FBgn0033985	36671	654.35	335.72
1631964_at	CG18262	---	down	1.95	9.01E-04	CG18262-RA	FBgn0030012	31745	147.26	75.57
1625563_s_at	CG14253	---	down	1.95	1.30E-03	CG14253-RB	FBgn0039467	43243	326.67	167.72
1634382_at	CG31675	---	down	1.94	6.28E-03	CG31675-RA	FBgn0051675	318879	255.48	131.44
1626659_at	CG4980	---	down	1.94	3.52E-02	CG4980-RA	FBgn0039558	43348	126.91	65.35
1640458_at	CG11211	---	down	1.94	8.52E-03	CG11211-RA	FBgn0033067	35545	6010.34	3095.80
1638281_at	CG7448	---	down	1.94	4.74E-02	S.C3L002796	FBgn0037143	40440	91.52	47.20
1626574_at	CG9935	---	down	1.94	6.12E-04	CG9935-RA	FBgn0039916	43806	1972.14	1017.49
1634041_at	CG31548	---	down	1.93	3.24E-03	CG31548-RA	FBgn0051548	318794	2001.81	1037.03
1624020_at	mod(mdg4)	Modifier67.2	down	1.92	5.59E-03	CG32491-RO	FBgn0002781	49228	235.09	122.47
1624395_at	CG31102	---	down	1.92	1.10E-02	CG31102-RA	FBgn0051102	326118	571.41	298.17
1632958_a_at	CG42365	---	down	1.91	2.35E-02	CG15675-RA	FBgn0259711	37469	390.64	204.44
1633863_at	---	---	down	1.91	2.47E-02	S.C3R003559	---	---	42.47	22.23
1638669_at	CG11395	---	down	1.91	3.67E-03	CG11395-RA	FBgn0034200	36937	3954.71	2072.41
1631772_a_at	MTA1-like	MTA-like	down	1.91	1.84E-03	CG2244-RA	FBgn0027951	40693	300.98	157.76
1622949_at	peb	hindsight	down	1.91	1.27E-03	CG12212-RA	FBgn0003053	31391	681.57	357.44
1627837_at	CG5527	---	down	1.91	1.54E-02	CG5527-RA	FBgn0039564	43356	432.17	226.66
1636998_at	sca	scabrous	down	1.91	1.35E-02	CG17579-RB	FBgn0003326	36411	320.94	168.41
1629222_at	Ahcy13	AdoHcyase	down	1.91	7.65E-03	CG11654-RA	FBgn0014455	32471	1751.84	919.50
1629240_at	Mst84Dc	Male-specific RNA 84Dc	down	1.90	3.21E-02	CG17945-RA	FBgn0004174	40887	65.12	34.22
1631784_at	CG13663	---	down	1.90	1.36E-02	CG13663-RA	FBgn0039291	43029	313.74	164.99
1631722_at	---	---	down	1.90	1.25E-02	GM02743	---	---	507.92	267.51
1625745_at	CG31643	---	down	1.90	1.62E-02	CG31643-RA	FBgn0051643	318867	184.60	97.35
1626384_at	CG12182	---	down	1.89	6.77E-03	CG12182-RA	FBgn0028504	38324	157.70	83.60
1633558_s_at	CG14997	---	down	1.89	7.57E-03	CG14997-RB	FBgn0035515	38514	806.55	427.74
1628451_at	SuUR	Suppressor of Under-Replication	down	1.88	3.39E-02	CG7869-RA	FBgn0025355	45739	230.56	122.40
1629771_at	Cyp12a5	Cyp12a5	down	1.88	3.27E-02	CG11821-RA	FBgn0038680	42293	272.37	144.66
1635362_at	CG13654	---	down	1.88	3.38E-02	CG13654-RA	FBgn0039290	43028	254.59	135.60
1637602_at	Edem1	CG3810	down	1.88	1.46E-02	CG3810-RC	FBgn0023511	31154	1003.08	534.36
1627465_at	Mes4	NF-YC-like	down	1.88	4.75E-03	CG11301-RA	FBgn0034726	37560	527.95	281.30
1640478_at	CG31777	---	down	1.86	1.70E-02	CG31777-RA	FBgn0051777	261617	1010.35	541.88
1625819_at	sno	Strawberry Notch	down	1.86	2.96E-02	CG1903-RB	FBgn0005410	32273	2400.06	1287.50
1624911_at	Cyp28d2	Cyp28d2	down	1.86	1.47E-02	CG6081-RA	FBgn0031688	33748	183.07	98.25
1640327_at	CG6023	---	down	1.86	1.10E-03	CG6023-RA	FBgn0030912	32814	393.99	211.80
1624440_at	---	---	down	1.85	3.89E-02	AM01482	---	---	377.60	203.73
1632752_at	Ast	drostatin-A4	down	1.85	3.06E-03	CG13633-RA	FBgn0015591	42947	1082.09	584.31
1624010_a_at	CG9062	---	down	1.85	1.04E-02	CG9062-RA	FBgn0033607	36199	176.75	95.53
1637154_at	CG7470	glutamate 5-kinase	down	1.85	3.74E-02	CG7470-RA	FBgn0037146	40443	3779.82	2045.46
1637626_at	CG15412	---	down	1.84	5.73E-04	CG15412-RA	FBgn0031528	33553	263.90	143.09
1632146_a_at	CG5508	---	down	1.84	2.15E-02	CG5508-RA	FBgn0027579	43350	3090.70	1677.90
1631650_a_at	spirit	CG2056	down	1.84	4.19E-02	CG2056-RB	FBgn0030051	31797	239.92	130.31
1634306_at	Klp98A	Kinesin-like protein at 98A	down	1.84	1.74E-02	CG5658-RA	FBgn0004387	43310	1107.84	601.97
1637130_at	CG30159	---	down	1.84	1.06E-03	CG30159-RA	FBgn0050159	35611	1397.72	760.02
1623091_at	CG12644	---	down	1.84	1.81E-02	CG12644-RA	FBgn0040940	50414	123.00	66.89
1640599_at	ade5	AIR-carboxylase-SAICAR syntheta	down	1.84	3.38E-03	CG3989-RA	FBgn0020513	32228	13873.97	7545.81
1641654_a_at	Lk6	Lk6	down	1.84	1.56E-03	CG17342-RA	FBgn0017581	44672	9988.57	5441.84
1628331_at	Ca-beta	calcium channel beta subunit	down	1.83	7.22E-03	CG6320-RB	FBgn0259822	34557	768.36	418.74
1633957_s_at	CG5316	---	down	1.83	2.34E-03	CG5316-RC	FBgn0038704	42322	787.33	429.62
1625997_s_at	---	---	down	1.83	1.06E-02	GH03753	---	---	15868.96	8662.46
1638114_at	CG13022	---	down	1.83	1.14E-02	CG13022-RA	FBgn0036679	39896	317.73	173.50
1640075_a_at	path	pathetic	down	1.83	2.42E-02	CG3424-RA	FBgn0036007	39106	3595.18	1963.47
1631315_at	CG12730	---	down	1.83	4.43E-03	CG12730-RA	FBgn0029771	31466	1821.89	995.90
1627742_at	Bmcp	Bmcp	down	1.83	4.31E-02	CG7314-RB	FBgn0036199	39322	543.15	296.95
1640720_a_at	CG14872	---	down	1.83	4.21E-02	CG14872-RB	FBgn0038346	41893	1611.09	881.34
1625699_at	---	---	down	1.83	1.71E-02	S.C3L001350	---	---	72.83	39.89
1636059_at	CG9689	---	down	1.82	2.34E-03	CG9689-RA	FBgn0030159	31922	1562.40	856.51
1639997_s_at	---	---	down	1.82	1.12E-02	CT39116	---	---	311.50	170.99
1624189_at	CG12236	---	down	1.82	2.06E-03	CG12236-RB	FBgn0029822	31523	800.19	439.26
1632245_a_at	Spn4	neuroserpin	down	1.82	9.66E-03	CG9453-RB	FBgn0028985	49805	1667.44	916.07
1637300_at	CG8229	---	down	1.82	1.25E-04	CG8229-RA	FBgn0033356	35898	1187.97	654.23
1632404_at	rtet	tetracycline resistance	down	1.82	1.33E-02	CG5760-RA	FBgn0028468	42500	231.22	127.34
1624405_a_at	CG7059	---	down	1.82	3.17E-02	CG7059-RA	FBgn0038957	42626	819.75	451.47
1638230_at	CG3160	---	down	1.82	6.09E-03	CG3160-RA	FBgn0029789	31487	329.31	181.38
1626965_at	CG31743 /// CG34313	--- /// ---	down	1.82	5.84E-03	CG31743-RA	0032618 /// FBgn008	326156 /// 5740766	2369.35	1305.38
1627925_at	spict	CG12292	down	1.81	5.13E-03	CG12292-RA	FBgn0032451	34681	1581.00	871.31
1627167_a_at	CG40485	---	down	1.81	3.91E-04	CG40485-RA	FBgn0069973	3355165	3542.86	1953.33
1627242_at	l(2)efl	lethal (2) essential for life	down	1.81	1.02E-03	CG4533-RA	FBgn0011296	37744	15695.22	8659.02
1626684_at	CG7607	---	down	1.81	8.17E-03	CG7607-RA	FBgn0036145	39263	2125.86	1175.16
1630036_at	CG13594	---	down	1.80	1.83E-03	CG13594-RA	FBgn0035041	37940	448.79	248.73
1623008_at	CG5565 /// DsimCG5565	--- /// ---	down	1.80	5.24E-03	CG5565-RA	0031335 /// FBgn008	33323	44.08	24.49
1638683_at	---	---	down	1.80	4.11E-03	HDC17231	---	---	142.74	79.39
1623569_at	CG14906 /// CG14907	open reading frame 2 /// ---	down	1.79	1.63E-02	CG14907-RA	0015351 /// FBgn003	42030 /// 42031	100.18	56.07
1626881_at	CG8907	---	down	1.79	3.04E-02	CG8907-RA	FBgn0038466	42050	91.48	51.20
1634873_at	---	---	down	1.79	3.36E-02	LD33458	---	---	127.93	71.63
1623643_s_at	Idgf3	Imaginal disc growth factor 3	down	1.78	6.85E-03	CG4559-RA	FBgn0020414	34981	4527.54	2536.88
1639457_at	CG32284	---	down	1.78	4.81E-02	CG32284-RA	FBgn0052284	317956	89.29	50.09
1638204_s_at	cav	anonymous fast evolving gene IG!	down	1.78	2.29E-02	CG6219-RE	FBgn0026257	42881	73.31	41.14
1640768_at	CG13965	---	down	1.78	3.52E-03	CG13965-RA	FBgn0032834	35280	592.22	332.55
1641284_a_at	CG6995	---	down	1.78	4.54E-03	CG6995-RA	FBgn0039229	42958	1087.69	611.09
1624497_at	---	---	down	1.78	1.78E-04	CR31541-RA	---	---	864.59	486.31
1636616_at	Or63a	Odorant receptor 63a	down	1.78	3.82E-02	CG9969-RA	FBgn0035382	38354	195.41	110.06
1639136_s_at	psq	pipsqueak	down	1.78	1.63E-02	CG2368-RB	FBgn0004399	36118	402.67	226.81
1633857_at	CG13659	---	down	1.77	1.13E-02	CG13659-RA	FBgn0039319	43059	117.04	65.98
1631497_at	Tsp42Eo	tetraspanin 42E	down	1.77	2.14E-02	CG12838-RA	FBgn0033136	35625	147.44	83.14
1637367_at	CG10799	---	down	1.77	3.78E-03	CG10799-RA	FBgn0033821	36472	3282.34	1851.03
1625428_at	CG13707	---	down	1.77	3.65E-03	CG13707-RA	FBgn0035578	38583	351.57	198.30
1639941_at	CG15317	---	down	1.77	4.29E-02	CG15317-RA	FBgn0030137	31898	170.45	96.25
1634753_at	CG5327	---	down	1.77	1.15E-02	CG5327-RA	FBgn0034363	37138	49.79	28.11
1628719_at	cry	cryptochrome	down	1.77	2.09E-02	CG3772-RA	FBgn0025680	42305	1491.74	842.50
1635284_a_at	HDAC4	HDAC4	down	1.77	1.35E-02	CG1770-RA	FBgn0041210	32278	978.39	552.63
1635165_a_at	CG12090 /// Ir62a	--- /// CG33971	down	1.77	5.96E-04	CG12090-RA	0035227 /// FBgn005	38176 /// 3885628	1543.70	873.73
1631723_at	CG5498	transcript 2	down	1.77	9.27E-03	CG5498-RB	FBgn0027565	40250	743.96	421.41
1638696_a_at	CG3552	---	down	1.76	2.00E-02	CG3552-RA	FBgn0035999	39097	572.88	324.83
1636040_at	CG10527	Farnesoic acid O-methyltransferas	down	1.76	6.57E-03	CG10527-RA	FBgn0034583	37393	5303.74	3009.00
1635488_at	CG31660	---	down	1.76	1.49E-03	CG31660-RB	FBgn0051660	33690	885.48	503.18
1632067_at	Ect4	Ect4	down	1.76	1.18E-02	CG7915-RB	FBgn0085402	38895	1548.58	880.00
1633011_at	crp	cropped	down	1.76	2.81E-03	CG7664-RA	FBgn0001994	34956	248.98	141.86
1627795_at	Ir	Drosophila inward rectifier	down	1.75	2.14E-03	CG6747-RA	FBgn0039061	42742	693.90	395.80
1623517_at	G4962 /// DmauCG4962 /// DsimCG496	--- /// --- /// ---	down	1.75	2.83E-03	CG4962-RA	/// FBgn0260188 ///	39793	3181.07	1814.87
1639729_s_at	---	---	down	1.75	4.31E-02	Transposon.35	---	---	2645.47	1509.33
1640303_a_at	pst	pastrel	down	1.75	4.41E-03	CG8588-RA	FBgn0035770	38818	8902.24	5089.60
1640276_a_at	Flo	flotillin-1	down	1.75	2.02E-02	CG8200-RA	FBgn0024754	36726	1896.59	1085.67
1624159_at	Cyp9h1	Cyp9h1	down	1.74	1.69E-03	CG17577-RA	FBgn0033775	36412	282.18	161.78
1637290_at	Dsk	sulfakinin	down	1.74	1.86E-02	CG18090-RA	FBgn0000500	45845	2508.79	1441.54
1638171_s_at	CG1753	---	down	1.74	9.79E-03	CG1753-RA	FBgn0031148	33081	636.97	366.02
1635858_s_at	Nhe2	Nhe2	down	1.74	1.31E-02	CG9256-RA	FBgn0040297	35376	86.70	49.84
1634856_at	Hdc	histidine decarboxylase	down	1.73	1.24E-02	CG3454-RA	FBgn0005619	36076	3049.43	1758.66
1627666_at	CG10979	---	down	1.73	2.30E-02	CG10979-RA	FBgn0037379	40720	182.95	105.68
1633036_s_at	CG32495 /// GS	--- /// Glutathione Synthetase	down	1.73	7.56E-03	CG32495-RA	0030882 /// FBgn005	318053 /// 32775	1599.63	924.84
1637853_a_at	Hk	Hyperkinetic	down	1.73	1.68E-02	CG32688-RB	FBgn0001203	31955	1714.56	992.79
1632332_at	CG5447	---	down	1.73	2.89E-03	CG5447-RA	FBgn0039427	43192	553.09	320.30
1629875_a_at	Cyp4d1	Cytochrome P450 4D1	down	1.72	2.73E-02	CG3656-RB	FBgn0005670	31188	121.08	70.21
1639489_at	CG3700 /// DmirCG3700 /// MED23	--- /// --- /// Mediator complex subu	down	1.72	3.16E-02	CG3700-RA	/// FBgn0034796 ///	37639 /// 37640	708.67	411.17
1626187_at	Brf	Brf	down	1.72	2.63E-03	CG31256-RA	FBgn0038499	42087	405.71	235.48
1628080_at	GIIIspla2	phospholipase A[[2]]	down	1.72	7.83E-03	CG1583-RA	FBgn0030013	31747	3193.09	1857.47
1628474_at	CG16712	---	down	1.72	1.70E-04	CG16712-RA	FBgn0031561	33592	27065.08	15753.20
1630886_at	SelR	methionine-R-sulfoxide reductase	down	1.71	1.80E-02	CG6584-RD	FBgn0037847	41309	594.77	347.26
1623923_a_at	gl	glass	down	1.71	1.17E-02	CG7672-RB	FBgn0004618	42210	561.40	328.92
1624332_s_at	CG4098 /// CR18217	--- /// ---	down	1.71	2.58E-02	CG4098-RA	0036646 /// FBgn003	39854 /// 8674109	324.75	190.34
1640924_a_at	CG3558	---	down	1.70	1.51E-03	CG3558-RA	FBgn0025681	48421	1535.51	900.96
1623265_at	CG10513	---	down	1.70	1.66E-02	CG10513-RA	FBgn0039311	43051	612.45	359.43
1637714_a_at	Ance-3	Ance-3	down	1.70	3.52E-03	CG31766-RA	FBgn0032536	34808	930.89	547.75
1633492_at	hgo	homogentisate 1,2-dioxygenase	down	1.70	2.29E-03	CG4779-RA	FBgn0040211	34552	733.26	431.62
1624957_a_at	Tequila	Tequila	down	1.70	4.00E-02	CG4821-RD	FBgn0023479	39048	4943.12	2909.86
1639192_at	gammaTub37C	gamma tubulin	down	1.70	2.11E-02	CG17566-RA	FBgn0010097	35199	87.83	51.75
1640631_at	CG32809	---	down	1.70	4.46E-02	CG32809-RD	FBgn0023531	31121	567.16	334.60
1641259_at	Chit	chitinase-like	down	1.69	3.40E-02	CG5210-RA	FBgn0013763	36868	4435.42	2618.30
1629115_s_at	scrib	Scribble	down	1.69	2.94E-03	CG5462-RA	FBgn0261263	44448	323.27	190.93
1626301_at	Idgf3	Imaginal disc growth factor 3	down	1.69	9.88E-03	CG4559-RA	FBgn0020414	34981	179.49	106.22

1631355_at	mRpS28	mitochondrial ribosomal protein S2	down	1.69	6.07E-04	CG5497-RA	FBgn0034361	37136	661.89	392.03
1623565_at	CG17278	---	down	1.69	4.63E-02	CG17278-RA	FBgn0046763	50050	1492.72	884.64
1632317_at	CG3036	---	down	1.68	1.18E-03	CG3036-RA	FBgn0031645	33695	7094.24	4216.27
1625170_at	CG8001	---	down	1.68	5.94E-03	CG8001-RA	FBgn0035268	38224	357.32	212.46
1637376_at	CG30108 /// CG30109	--- /// ---	down	1.68	2.81E-02	CG30108-RA	0050108 /// FBgn005	246460 /// 246461	78.60	46.79
1641265_at	tty	tweety	down	1.68	3.57E-02	CG1693-RA	FBgn0015558	33109	585.89	349.10
1630380_at	CG3837	---	down	1.68	2.41E-02	CG3837-RA	FBgn0038279	41809	655.31	390.49
1623665_at	---	---	down	1.68	3.64E-02	CG32716-RA	---	---	280.86	167.41
1641069_at	CG10703	---	down	1.67	3.13E-02	CG10703-RA	FBgn0037881	41350	307.86	183.84
1637264_at	ncd	non-claret disjunctional	down	1.67	3.35E-02	CG7831-RA	FBgn0002924	43517	85.84	51.32
1624599_at	CG34331	---	down	1.67	1.37E-02	CG12656-RA	FBgn0085360	33011	1388.20	829.97
1630285_at	RhoGAP100F	RhoGAP100F	down	1.67	1.52E-02	CG1976-RA	FBgn0039883	43758	1424.39	854.94
1627653_at	l(1)G0469	lethal (1) G0469	down	1.67	3.43E-02	CG32597-RA	FBgn0040153	32371	198.45	119.16
1623277_at	cwo	clockwork orange	down	1.66	6.69E-03	CG17100-RA	FBgn0259938	44669	1634.55	982.04
1629793_at	CG12772	---	down	1.66	2.70E-02	CG12772-RA	FBgn0030055	31803	497.56	298.94
1633959_s_at	CG30345 /// CG9766 /// DsimCG9766	--- /// --- /// ---	down	1.66	2.12E-02	Transposon.10	/// FBgn0050345 ///	246553 /// 40539	1650.34	991.83
1625837_at	CG17244	---	down	1.66	2.21E-02	CG17244-RA	FBgn0039031	42709	2215.71	1331.81
1624053_at	CG33967	---	down	1.66	4.67E-02	CG7552-RA	FBgn0053967	41783	266.99	160.51
1624586_a_at	Prat2	Phosphoribosylamidotransferase 2	down	1.66	4.16E-03	CG10078-RB	FBgn0041194	38753	2793.04	1680.33
1640062_at	CG34319	---	down	1.66	1.36E-02	S.CX001111	FBgn0085348	5740276	296.17	178.34
1638368_at	CG31674	---	down	1.66	2.11E-02	CG31674-RA	FBgn0051674	318878	206.95	124.64
1641534_at	CG11842	---	down	1.66	5.49E-04	CG11842-RA	FBgn0039629	43431	2531.30	1526.31
1628309_at	CG5126	---	down	1.66	1.27E-02	CG5126-RA	FBgn0031320	33306	244.58	147.48
1632809_at	CG14762	gp150-like	down	1.66	4.19E-05	CG14762-RA	FBgn0033250	35768	7208.20	4349.78
1624543_s_at	---	---	down	1.66	3.33E-04	Transposon.32	---	---	2191.45	1323.28
1640975_at	Lcp65Ag2	Lcp65Ag2	down	1.65	2.98E-02	CG10534-RA	FBgn0020637	38702	213.67	129.35
1634854_at	CG16989	---	down	1.65	7.76E-03	CG16989-RA	FBgn0025621	31026	244.49	148.32
1635548_s_at	CG30287 /// CG34166	--- /// ---	down	1.64	1.04E-02	CG15281-RA	0050287 /// FBgn008	246530 /// 5740596	4090.30	2489.60
1626377_at	CG8358	---	down	1.64	1.45E-02	CG8358-RA	FBgn0037727	41172	236.46	144.02
1625624_at	CG14105	---	down	1.64	1.00E-03	CG14105-RA	FBgn0036359	39507	460.02	280.55
1631227_at	CG5793	---	down	1.64	2.44E-02	CG5793-RA	FBgn0038858	42505	3423.97	2090.53
1638001_a_at	CG8889	---	down	1.64	2.18E-04	CG8889-RA	FBgn0259985	36285	5452.40	3329.09
1625046_at	Rbm13	CG10648	down	1.64	1.81E-02	CG10648-RA	FBgn0030067	31818	121.96	74.56
1624070_at	RpS9	Ribosomal protein S9	down	1.64	1.03E-02	CG3395-RB	FBgn0010408	39108	898.17	549.29
1629795_at	PTP-ER	Protein tyrosine phosphatase-ERK	down	1.63	7.49E-03	CG9856-RA	FBgn0016641	37461	1252.61	766.43
1634241_at	CG4860	---	down	1.63	2.75E-02	CG4860-RA	FBgn0037999	41480	278.65	171.00
1629518_at	CG2493 /// DsecGM23324	--- /// ---	down	1.63	2.92E-02	CG2493-RA	0032864 /// FBgn017	35316	880.54	540.45
1640822_s_at	qkr54B	quaking related 54B	down	1.63	3.16E-02	CG4816-RA	FBgn0022987	36966	687.94	422.35
1628856_at	---	---	down	1.63	2.20E-02	CG31181-RA	---	---	348.96	214.54
1629898_s_at	CG10737	Gene 1	down	1.63	9.81E-04	CG10737-RD	FBgn0034420	37202	1150.87	707.97
1625514_s_at	olf186-F	lethal (2) k11505	down	1.63	2.37E-02	CG11430-RB	FBgn0041585	37040	927.67	570.76
1626240_s_at	CG6236	---	down	1.63	3.75E-03	CG6236-RB	FBgn0038318	41857	275.53	169.55
1641131_at	CG5621	---	down	1.62	2.81E-02	CG5621-RA	FBgn0038840	42476	283.35	174.47
1629044_at	trbl	tribbles	down	1.62	3.56E-03	CG5408-RA	FBgn0028978	43999	224.56	138.34
1630514_at	Gprk1	rhodopsin kinase	down	1.62	1.72E-02	CG40129-RA	FBgn0260798	3355013	1576.28	971.78
1635575_at	---	---	down	1.62	4.85E-02	CG40032-RA	---	---	91.10	56.17
1624481_a_at	CG31534	---	down	1.62	2.12E-02	CG31534-RA	FBgn0051534	40585	3017.02	1861.55
1628618_at	CG9240	---	down	1.62	1.42E-02	CG9240-RA	FBgn0030669	32505	1532.42	946.30
1631931_s_at	Sdc	Syndecan	down	1.62	1.03E-02	CG10497-RC	FBgn0010415	37447	6471.47	3997.72
1629492_s_at	CG31626	---	down	1.61	3.49E-02	CG31626-RA	FBgn0051626	318859	191.22	118.42
1626552_at	CG3568	---	down	1.61	3.93E-03	CG3568-RA	FBgn0029710	31383	238.25	147.72
1625756_at	CG6656	---	down	1.61	2.31E-04	CG6656-RA	FBgn0038912	42576	2075.98	1289.94
1639346_at	frj	Farjavit	down	1.61	5.94E-03	CG9526-RA	FBgn0031815	33900	1730.06	1077.05
1627825_at	CG13305	---	down	1.60	5.34E-04	CG13305-RA	FBgn0035921	38992	489.55	305.22
1635227_at	ImpL3	lactic DH	down	1.60	2.26E-02	CG10160-RA	FBgn0001258	45880	330.84	206.30
1640032_a_at	CG3814 /// TTLL3B	--- /// CG11201	down	1.60	3.15E-02	CG3814-RB	0025692 /// FBgn003	33946 /// 36418	161.42	100.73
1623171_s_at	lilli	lilliputian	down	1.60	1.07E-03	GM02019	FBgn0041111	33496	189.76	118.43
1623266_at	CG14180	---	down	1.60	6.27E-03	CG14180-RA	FBgn0036003	39101	141.87	88.66
1625250_at	CG5802	anon-fast-evolving-1H6	down	1.60	8.87E-03	CG5802-RA	FBgn0250820	42510	1492.94	932.96
1623669_at	CG16836	---	down	1.60	1.25E-02	CG16836-RA	FBgn0040735	50208	13521.71	8454.45
1628684_at	Teh1	tipE homolog 1	down	1.60	1.04E-02	CG12806-RA	FBgn0037766	41215	142.91	89.37
1635183_at	Spn43Ab	Serine protease inhibitor 2	down	1.60	2.28E-02	CG1865-RA	FBgn0024293	45040	7366.35	4606.94
1636934_at	zormin	D-Titin	down	1.60	3.11E-02	CG32307-RA	FBgn0052311	2769001	239.12	149.73
1638445_a_at	SPoCk	Secretory Pathway Calcium atpas	down	1.60	4.36E-03	CG32451-RA	FBgn0052451	40495	6897.95	4322.80
1626545_at	pHCl	pHCl	down	1.60	2.75E-02	Dm.3L.31789.0	FBgn0036542	39730	189.29	118.65
1634939_at	CG13077	---	down	1.59	2.86E-03	CG13077-RA	FBgn0032810	35252	3006.72	1885.25
1624103_at	CG7246	---	down	1.59	3.85E-02	CG7246-RA	FBgn0030081	31833	121.26	76.06
1623241_s_at	CG30104	---	down	1.59	2.01E-02	CG30104-RB	FBgn0050104	246458	395.04	247.91
1640562_at	CG30033	---	down	1.59	8.80E-03	CG30033-RB	FBgn0050033	246406	2001.03	1256.17
1640042_at	CG12299	---	down	1.59	1.52E-02	CG12299-RA	FBgn0032295	34483	916.14	575.17
1628567_at	CG13565	---	down	1.59	1.74E-02	CG13565-RA	FBgn0034935	37806	992.92	624.10
1631151_at	CG4951	---	down	1.59	1.70E-02	CG4951-RA	FBgn0039563	43355	146.04	91.80
1634789_at	CG30151	---	down	1.59	3.37E-02	CG30151-RA	FBgn0050151	246484	531.21	334.00
1632748_at	CG15084	---	down	1.59	7.89E-03	CG15084-RA	FBgn0034402	37178	141.93	89.46
1625218_at	CG11399	---	down	1.58	3.72E-03	CG11399-RB	FBgn0037021	40295	462.24	291.75
1639785_s_at	dpld /// wech	dappled /// dappled	down	1.58	3.37E-02	CG1624-RC	0015930 /// FBgn025	44653 /// 7411854	441.64	278.83
1637361_a_at	CG12179	---	down	1.58	8.48E-03	CG12179-RB	FBgn0025388	31393	71.34	45.06
1626315_at	Rip11	lethal (1) G0003	down	1.58	6.19E-03	CG6606-RA	FBgn0027335	32850	2029.46	1282.06
1630410_at	CG32163	---	down	1.58	7.87E-03	CG32163-RA	FBgn0052163	317891	402.09	254.31
1635870_at	CG1628	---	down	1.58	2.26E-02	CG1628-RB	FBgn0030218	31990	495.59	313.55
1634699_a_at	CG11919 /// CG18003	--- /// ---	down	1.58	2.86E-02	CG30019-RB	0033564 /// FBgn006	3771779 /// 3772165	1005.06	635.96
1635792_a_at	Ela	elastin-like	down	1.58	4.07E-02	CG7021-RA	FBgn0013949	42970	1559.47	986.77
1639048_a_at	CG8229	---	down	1.58	8.62E-03	CG8229-RA	FBgn0033356	35898	814.96	515.69
1628320_at	CG9471	biliverdin reductase	down	1.58	7.59E-04	CG9471-RA	FBgn0037749	41197	1272.51	805.54
1634235_at	CG34388	CG14842	down	1.58	1.03E-02	CG14843-RA	FBgn0085417	5740700	215.13	136.20
1631633_a_at	GNBP2	Gram-negative bacteria binding pr	down	1.58	1.09E-03	CG4144-RB	FBgn0040322	40033	563.86	357.03
1632200_s_at	Edem1	CG3810	down	1.58	2.69E-03	CG3810-RB	FBgn0023511	31154	338.71	214.69
1636749_at	---	---	down	1.58	2.43E-02	Transposon.99	---	---	111.76	70.85
1629493_at	U26	U26	down	1.58	2.90E-03	CG13401-RA	FBgn0027780	34175	435.21	276.17
1637749_at	D2R	Dopamine 2-like receptor	down	1.57	2.85E-02	CG17004-RB	FBgn0053517	33007	713.91	453.80
1626113_at	CG4080	---	down	1.57	1.18E-02	CG4080-RA	FBgn0035983	39079	719.51	457.59
1628357_at	CG6142	---	down	1.57	1.38E-02	CG6142-RA	FBgn0039415	43178	92.01	58.52
1628677_at	Pkcdelta	protein kinase C	down	1.57	3.32E-03	CG10524-RA	FBgn0259680	32191	475.35	302.49
1635416_at	CG31100	---	down	1.57	2.70E-03	CG31100-RA	FBgn0051100	41075	714.91	455.17
1632684_s_at	CG4575 /// gt	--- /// giant	down	1.57	8.06E-03	CG4575-RA	0001150 /// FBgn002	31227 /// 31637	174.59	111.23
1626339_at	garz	gartenzwerg	down	1.57	1.84E-02	CG8487-RB	FBgn0033714	36337	1086.42	692.58
1627219_at	nAcRbeta-64B	AChR protein of Drosophila	down	1.57	6.09E-03	CG11348-RB	FBgn0000038	38545	3578.70	2283.88
1624285_at	CG3088	---	down	1.57	4.20E-03	CG3088-RA	FBgn0036015	39115	15432.29	9852.85
1635518_at	nonA	no-on-transient A	down	1.57	4.87E-02	CG4211-RA	FBgn0004227	32603	596.42	380.84
1629423_at	ics	Ras suppressor-1	down	1.57	6.54E-04	CG9031-RA	FBgn0028546	34774	1438.37	918.51
1640217_at	CG30154	---	down	1.57	4.43E-02	CG30154-RA	FBgn0050154	246487	859.08	548.78
1626473_a_at	fon	CG15825	down	1.56	5.07E-03	CG15825-RB	FBgn0032773	35211	11388.36	7278.37
1634631_at	CG3105	PAS kinase	down	1.56	8.77E-03	CG3105-RA	FBgn0034950	37824	755.67	483.26
1625686_s_at	su(r)	Drosophila Rhythmically expresse	down	1.56	5.60E-03	CG2194-RB	FBgn0086450	31858	954.09	611.01
1628688_at	CG12054	---	down	1.56	2.85E-02	CG12054-RA	FBgn0039831	43694	979.82	628.47
1632636_at	CG2145	---	down	1.56	2.43E-02	CG2145-RA	FBgn0030251	32027	4072.99	2613.11
1636953_a_at	---	---	down	1.56	2.20E-03	CG32171-RB	---	---	7662.60	4922.50
1625611_at	nimB4	CG33115	down	1.56	6.88E-05	CG33115-RA	FBgn0028542	34814	301.33	193.63
1635360_at	CG10830	---	down	1.56	3.33E-03	CG10830-RA	FBgn0038839	42475	3163.31	2033.44
1627434_at	CG5741	---	down	1.55	2.89E-02	CG5741-RA	FBgn0035937	39018	770.66	496.62
1625481_a_at	retn	dead ringer	down	1.55	1.33E-04	CG5403-RA	FBgn0004795	45976	1007.74	649.96
1629347_at	klu	klumphuss	down	1.55	1.99E-02	CG12296-RA	FBgn0013469	39228	173.86	112.26
1639857_at	CG32068	---	down	1.55	8.61E-03	CG32068-RA	FBgn0052068	326189	2393.62	1547.09
1630842_s_at	CG32640 /// CG32641	--- /// ---	down	1.55	1.23E-02	CG32641-RA	0052640 /// FBgn005	318135 /// 318136	1320.70	853.99
1627899_at	---	---	down	1.55	1.77E-02	RE31004	---	---	198.69	128.50
1629776_a_at	Esyt2	---	down	1.55	8.59E-03	CG6643-RA	FBgn0039208	42929	2514.23	1626.66
1637823_at	CG1434	---	down	1.55	2.47E-02	CG1434-RA	FBgn0030554	32377	417.76	270.30
1626988_at	CG6805	---	down	1.55	4.08E-03	CG6805-RA	FBgn0034179	36911	1190.48	770.46
1635713_at	CG13185	---	down	1.54	5.26E-03	CG13185-RA	FBgn0033661	36268	284.32	184.09
1639232_s_at	SP1029	SP1029	down	1.54	4.93E-02	CG11956-RA	FBgn0040282	53473	927.27	600.69
1624986_at	CG9836	---	down	1.54	2.71E-03	CG9836-RA	FBgn0037637	41059	8121.09	5262.75
1639611_at	CG8709	---	down	1.54	6.21E-03	CG8709-RA	FBgn0033269	35790	2091.21	1356.64
1639968_at	Ciao1	Ciao1	down	1.54	1.46E-02	CG12797-RA	FBgn0033972	36655	1041.60	675.76
1631420_at	CG9509	---	down	1.54	3.04E-02	CG9509-RA	FBgn0030594	32420	60.61	39.36
1635382_at	dlg1	Discslarge	down	1.54	2.22E-03	CG1725-RF	FBgn0001624	32083	2620.86	1703.12
1631128_s_at	CG9016	---	down	1.54	3.19E-02	CG9016-RA	FBgn0031751	33822	117.07	76.11
1638074_at	CG6673	---	down	1.54	1.94E-02	CG6673-RA	FBgn0035906	38974	504.39	327.99
1635729_a_at	Ptp61F	protein tyrosine phosphatase	down	1.54	3.01E-02	CG9181-RC	FBgn0003138	38160	125.28	81.47
1630457_s_at	by	tensin	down	1.54	3.78E-02	CR_tc_RE65113	FBgn0000244	41144	710.91	462.49
1635862_at	CG33120	---	down	1.54	3.22E-03	CG33120-RA	FBgn0053120	326260	439.37	285.84
1637125_at	CG2004	---	down	1.53	3.18E-02	CG2004-RA	FBgn0030060	31809	1328.13	865.71
1638193_at	CG5612	---	down	1.53	1.96E-02	CG5612-RA	FBgn0039529	43316	652.64	425.44
1632341_at	CG13559	---	down	1.53	7.45E-03	CG13559-RA	FBgn0034870	37720	127.57	83.18
1627590_at	CG9336	---	down	1.53	3.44E-04	CG9336-RA	FBgn0032897	35355	8374.54	5472.14
1627060_s_at	Gel	gelsolin	down	1.53	4.61E-03	CG1106-RB	FBgn0010225	46008	6273.89	4104.50
1640974_a_at	CG33143	---	down	1.53	4.52E-03	CG33143-RC	FBgn0053143	37583	1144.95	749.21
1634296_s_at	CG17646	---	down	1.53	1.95E-02	CG17646-RB	FBgn0031362	33354	1908.78	1249.51
1626052_at	MP1	Melanization Protease 1	down	1.53	5.35E-03	CG1102-RA	FBgn0027930	40541	1811.02	1186.59
1626780_s_at	CG4678	---	down	1.53	6.32E-03	CG4678-RB	FBgn0030778	32652	2021.36	1324.43
1630704_at	---	---	down	1.53	7.46E-03	HDC20453	---	---	74.13	48.59
1629637_at	CG13794	---	down	1.53	3.33E-02	CG13794-RA	FBgn0031936	34048	964.00	632.05
1625620_a_at	CG14425 /// Sxl	--- /// sex-lethal	down	1.52	2.64E-02	CG33070-RC	0003659 /// FBgn002	3771983 /// 3772180	2001.80	1313.25
1628187_s_at	CG9691	---	down	1.52	7.76E-05	CG9691-RA	FBgn0030160	31923	14574.06	9563.38
1628009_at	fas	faint sausage	down	1.52	2.56E-02	CG17716-RA	FBgn0000633	36521	981.44	645.18
1640898_at	DNaseII	acid DNase	down	1.52	4.83E-02	CG7780-RA	FBgn0000477	48228	1445.42	950.57
1635223_at	CG30285 /// rap	--- /// fizzy-related	down	1.52	4.22E-03	CG30285-RA	0003200 /// FBgn005	246528 /// 45922	1325.18	872.36
1632003_a_at	Ptp99A	Protein tyrosine phosphatase 99A	down	1.52	1.48E-02	CG2005-RB	FBgn0004369	43469	143.02	94.19
1625065_s_at	SK	small conductance calcium-activat	down	1.52	2.22E-02	CG10706-RC	FBgn0029761	31456	2956.69	1948.62
1626766_s_at	daw	dActivin2	down	1.52	3.71E-03	CG16987-RB	FBgn0031461	33474	831.62	548.16
1640202_at	Idgf2	Imaginal disc growth factor 2	down	1.52	2.79E-03	CG4475-RA	FBgn0020415	34979	4585.62	3024.44
1640366_at	CG5608	---	down	1.52	2.68E-02	CG5608-RA	FBgn0038058	41543	394.20	260.08
1629319_at	CG10428	---	down	1.52	6.58E-04	CG10428-RA	FBgn0032724	35150	761.50	502.45
1641120_at	CG13506	---	down	1.51	3.36E-03	CG13506-RA	FBgn0034723	37556	2284.14	1508.08
1632019_s_at	CG9008	---	down	1.51	2.85E-02	CG9008-RA	FBgn0028540	34779	1292.56	853.59
1636848_at	CG6024	---	down	1.51	1.51E-02	CG6024-RA	FBgn0036202	39325	1272.62	840.98
1624932_at	Obp49a	Odorant-binding protein 49a	down	1.51	4.02E-02	CG30052-RA	FBgn0050052	36399	5026.95	3322.84
1638453_at	CG9849	---	down	1.51	1.93E-04	CG9849-RA	FBgn0034803	37647	856.75	566.46
1640395_a_at	dos	Suppressor of sevenless-S11	down	1.51	1.14E-02	CG1044-RA	FBgn0016794	38321	516.18	341.41
1637307_at	CG31694	---	down	1.51	2.28E-02	CG31694-RA	FBgn0051694	33489	4007.81	2651.29
1623682_a_at	Cam	calmodulin	down	1.51	1.20E-02	CG8472-RA	FBgn0000253	36329	28954.78	19154.57
1625873_at	CG15661	---	down	1.51	3.96E-02	CG15661-RA	FBgn0034605	37421	786.54	520.35
1625146_a_at	RN-tre	tre oncogene-related protein	down	1.51	3.69E-02	CG8085-RA	FBgn0020620	36554	138.22	91.62
1641018_s_at	CG8468	---	down	1.51	1.22E-02	CG8468-RC	FBgn0033913	36577	2553.41	1693.00
1626805_s_at	gish	gilgamesh	down	1.51	8.73E-04	CG6963-RD	FBgn0250823	49701	1940.93	1288.86
1628774_a_at	boi	CG32796	down	1.51	3.47E-02	CG32796-RB	FBgn0040388	31229	126.53	84.03
1623869_at	CtBP	C-terminal Binding Protein	down	1.51	4.74E-02	S.C3R001251	FBgn0020496	41602	280.63	186.39
1636950_at	CG11077	---	down	1.51	2.49E-03	CG11077-RA	FBgn0039930	43831	1269.42	843.26
1630441_at	CG12716	---	down	1.50	2.86E-02	CG12716-RA	FBgn0030439	32248	49.53	32.93
1638334_at	CG13168	---	down	1.50	2.13E-02	CG13168-RA	FBgn0033705	36327	79.37	52.78
1638509_at	5PtaseI	5PtaseI	down	1.50	3.33E-04	CG31110-RA	FBgn0259178	326119	1199.50	798.30
1628005_at	chinmo	Chronologically inappropriate mor	down	1.50	4.05E-03	CG31666-RC	FBgn0086758	33343	1038.49	691.42
1629064_at	ewg	erect wing	down	1.50	7.86E-03	CG3114-RA	FBgn0005427	30975	1351.53	900.69
