# Supplementary material for: TDP-43 prevents retrotransposon activation in the Drosophila motor system through regulation of Dicer-2 activity
Source: BMC Biol. 2020 Jul 3;18:82. doi: 10.1186/s12915-020-00816-1 (PMC7334854; doi:10.1186/s12915-020-00816-1)
Supplement: Supplementary file 3 — Additional file 3 : Fig. S1. a Real time quantitative PCR of blastopia, burdock and springer transcript levels normalized on Rpl11 (housekeeping) in w1118 - tbphΔ23,elav-GAL4/tbphΔ23; UAS-GFP/+ and tbphΔ23,elav-GAL4/tbphΔ23,UAS-TBPH. (2biological replicates, with 3 technical replicates for each), error bars SEM. b Microarray results of downregulated TEs in TBPH mutants: the fold change of TEs was reported for both tbph mutant alleles (Δ23 and Δ142) referred to w1118; TEs family and class were also indicated. [file 12915_2020_816_MOESM3_ESM.docx]

**Additional file 3 Fig. S1**

**
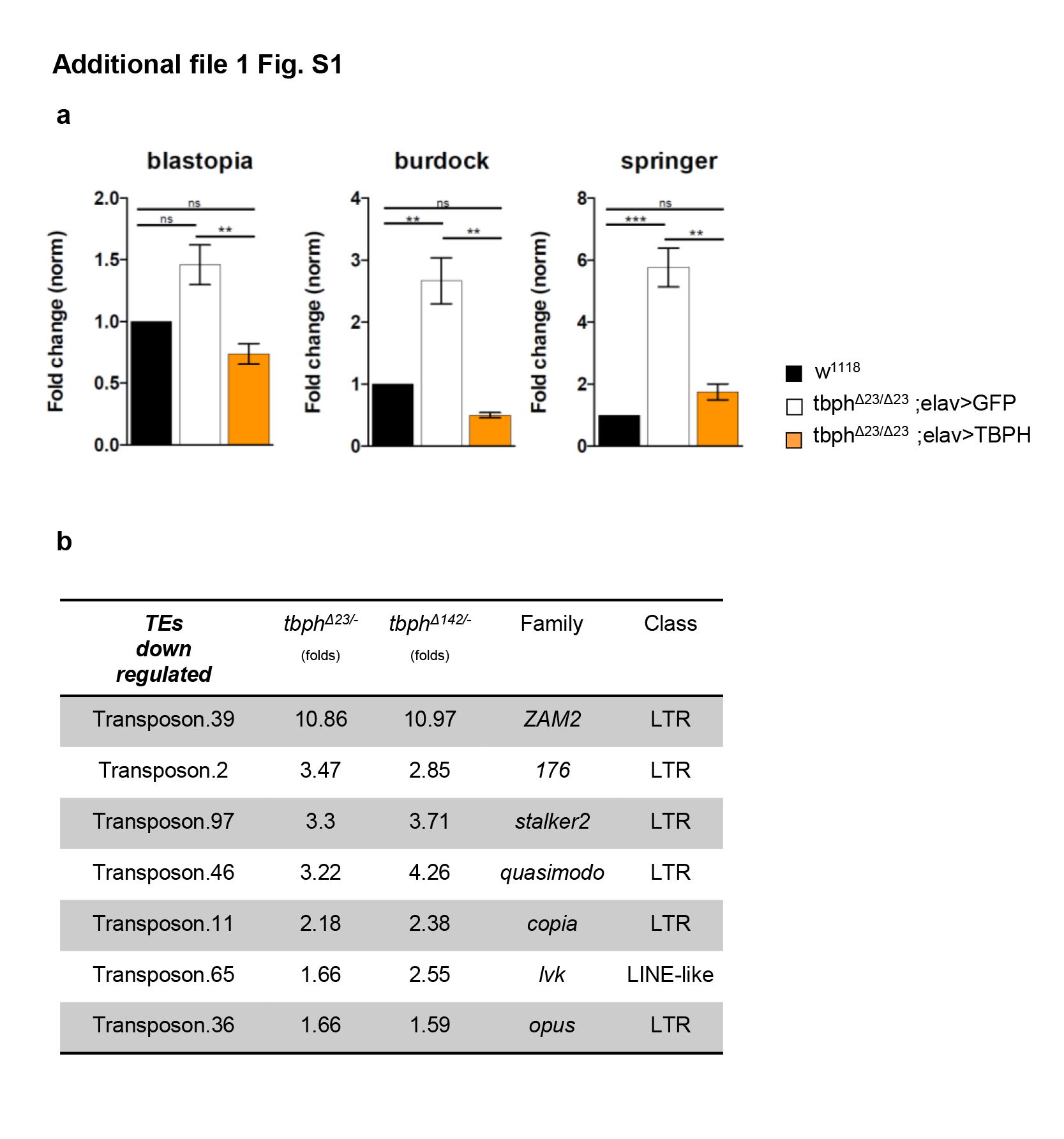
**

**Additional file 3 Fig. S1**

**a** Real time quantitative PCR of *blastopia*, *burdock* and *springer* transcript levels normalized on *Rpl11* (housekeeping) in *w*^1118^ - tbph^Δ23^,elav-GAL4/tbph^Δ23^; UAS-GFP/+ and tbph^Δ23^,elav-GAL4/tbph^Δ23^,UAS-TBPH. (2biological replicates, with 3 technical replicates for each), error bars SEM. **b** Microarray results of downregulated TEs in TBPH mutants: the fold change of TEs was reported for both tbph mutant alleles (Δ23 and Δ142) referred to *w*^1118^; TEs family and class were also indicated.
