# Supplementary material for: TDP-43 prevents retrotransposon activation in the Drosophila motor system through regulation of Dicer-2 activity
Source: BMC Biol. 2020 Jul 3;18:82. doi: 10.1186/s12915-020-00816-1 (PMC7334854; doi:10.1186/s12915-020-00816-1)
Supplement: Supplementary file 4 — Additional file 4 : Fig. S2. a Number of peristaltic waves of Ctrl (w1118) and Δtb (tbphΔ23/ tbphΔ23) fed with NRTIs drugs (D) compared to vehicle only (V). n=20. b Number of peristaltic waves of Ctrl (w1118), Δtb-GFP-IR (tbphΔ23 /tbphΔ23; Repo-GAL4/UAS-GFP-IR) and Δtb-gypsy-IR3 (tbphΔ23/tbphΔ23; Repo-GAL4/UAS-gypsy-IR3). n=20. ns=not significant, ***p<0.001 calculated by one-way ANOVA, error bars SEM. [file 12915_2020_816_MOESM4_ESM.docx]

**Additional file 4 Fig. S2**

**
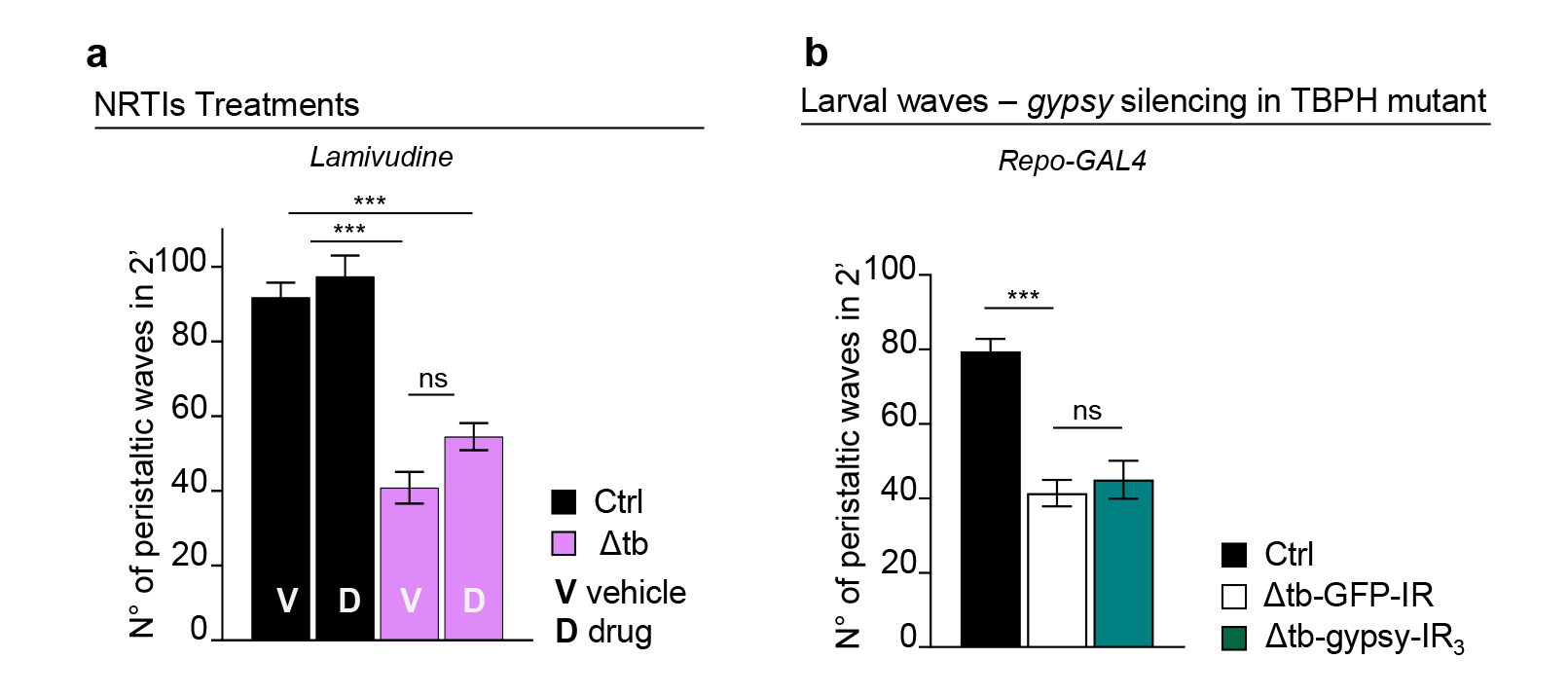
**

**Additional file 4 Fig. S2**

**a** Number of peristaltic waves of Ctrl (*w*^1118^) and Δtb (tbph^Δ23^/ tbph^Δ23^) fed with NRTIs drugs (D) compared to vehicle only (V). *n*=20. **b** Number of peristaltic waves of Ctrl (*w*^1118^), Δtb-GFP-IR (tbph^Δ23^ /tbph^Δ23^; Repo-GAL4/UAS-GFP-IR) and Δtb-gypsy-IR_3_ (tbph^Δ23^/tbph^Δ23^; Repo-GAL4/UAS-gypsy-IR_3_). *n*=20. ns=not significant, ***p<0.001 calculated by one-way ANOVA, error bars SEM.
