# Supplementary material for: TDP-43 prevents retrotransposon activation in the Drosophila motor system through regulation of Dicer-2 activity
Source: BMC Biol. 2020 Jul 3;18:82. doi: 10.1186/s12915-020-00816-1 (PMC7334854; doi:10.1186/s12915-020-00816-1)
Supplement: Supplementary file 5 — Additional file 5 : Fig. S3. Real time PCR of Dicer-2 (Dcr-2), Argonaute 2 (Ago2), Pasha, Piwi, Loquacious (Loq) and Homeless transcript levels normalized on Rpl11 (housekeeping) in adult heads of w1118, tbphΔ23/tbphΔ23 and tbphΔ142/tbphΔ142. n=2, error bars SEM. [file 12915_2020_816_MOESM5_ESM.docx]

**Additional file 5 Fig. S3**

**
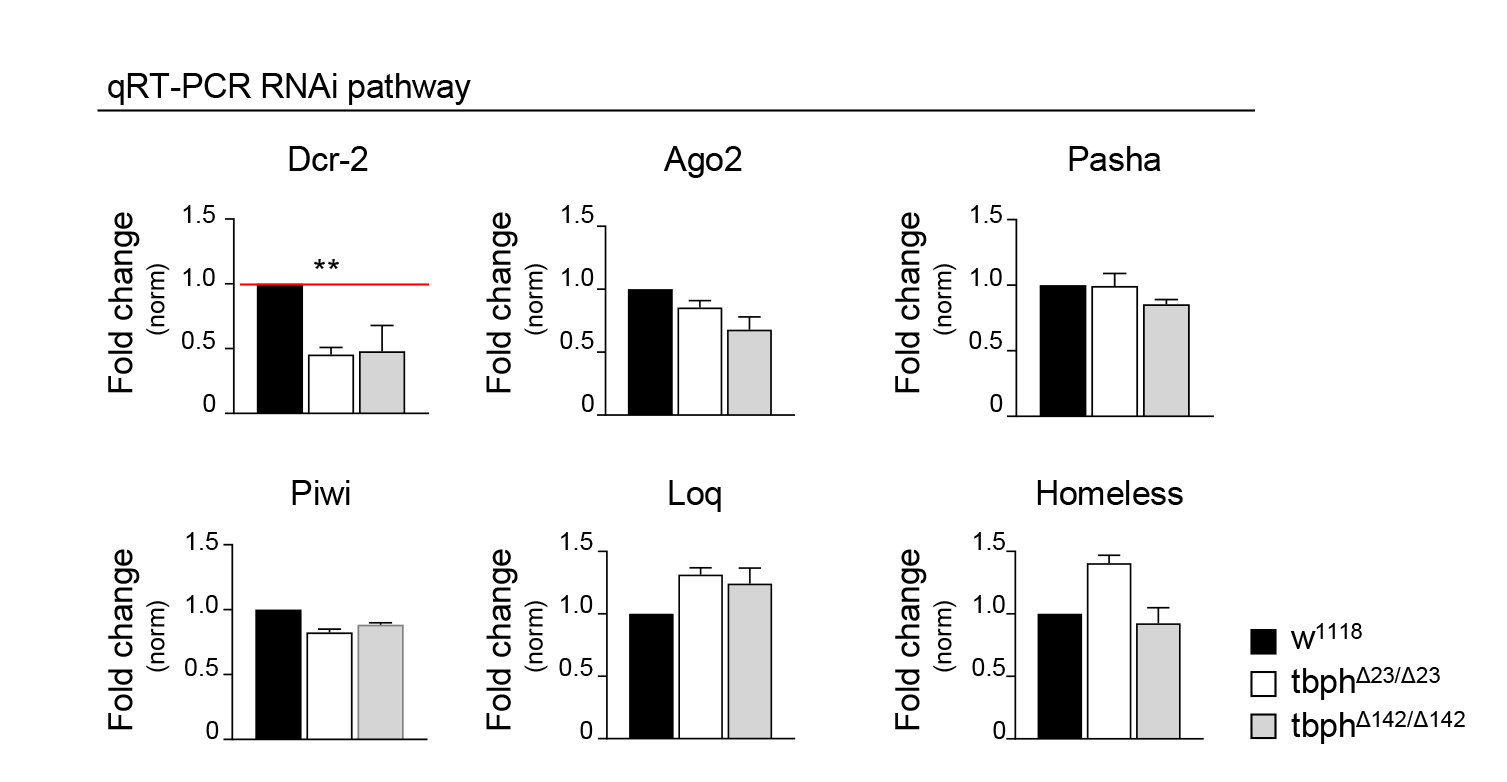
**

**Additional file 5 Fig. S3**

Real time PCR of *Dicer-2* (*Dcr-2), Argonaute 2 (Ago2), Pasha, Piwi, Loquacious (Loq)* and *Homeless* transcript levels normalized on *Rpl11* (housekeeping) in adult heads of *w*^1118^, tbph^Δ23^/tbph^Δ23^ and tbph^Δ142^/tbph^Δ142^. *n*=2, error bars SEM.
