# Supplementary material for: TDP-43 prevents retrotransposon activation in the Drosophila motor system through regulation of Dicer-2 activity
Source: BMC Biol. 2020 Jul 3;18:82. doi: 10.1186/s12915-020-00816-1 (PMC7334854; doi:10.1186/s12915-020-00816-1)
Supplement: Supplementary file 6 — Additional file 6. : Individual data values. [file 12915_2020_816_MOESM6_ESM.pdf]

Figure 1-Individual Data Values

b	qRT-PCR - Adult brains		
	**accord**		
	w^{1118}	$tbph^{\Delta23/\Delta23}$;elav>GFP	$tbph^{\Delta23/\Delta23}$;elav>TBPH
exp1	1.00	4.13	1.10
exp2	1.00	3.85	0.95
exp3	1.00	4.05	1.05
average	1.00	4.01	1.03
	**gypsy**		
	w^{1118}	$tbph^{\Delta23/\Delta23}$;elav>GFP	$tbph^{\Delta23/\Delta23}$;elav>TBPH
exp1	1.00	2.80	0.86
exp2	1.00	1.65	1.82
exp3	1.00	2.10	0.90
average	1.00	2.18	1.19

normalized values

c	WB - Adult brains			
	anti-ENV			
	lane 1	lane 2	lane 3	lane 4
exp1	21	100	162	61
exp2	26	100	166	49
average	23.5	100	164	55

normalized values

Figure 3-Individual Data Values

a **RNA silencing machinery is impaired in TBPH mutants**

	lane 1	lane 2	lane 3	lane 4
exp1	100	44	99	87
exp2	100	26	93	45
mean	100	35	96	66

normalized values

b **Dcr-2 levels in TBPH mutant**

	w^{1118}	$tbph^{\Delta23/\Delta23}$
exp1	100	44
exp2	100	79
exp3	100	48
mean	100	57

normalized values

c **Dcr-2 is reduced in TBPH mutant**

	w^{1118}	$tbph^{\Delta23/\Delta23}$
exp1	100	57
exp2	100	64
exp3	100	64
exp4	100	52
mean	100	59

normalized values

d **SH-SY5Y (human neuroblastoma)**

Dicer	lane 1	lane 2
exp1	100	80
exp2	100	47
exp3	100	48
mean	100	58

TDP-43	lane 1	lane 2
exp1	100	32
exp2	100	54
exp3	100	12
mean	100	33

normalized values

e **Co-IP - Adult heads**

Protein-mRNA

dicer-2	Elav>TBPH	Elav>TBPH-RBD^{mut}
exp1	4.6	1
exp2	3	1
mean	3.8	1

syntaxin	Elav>TBPH	Elav>TBPH-RBD^{mut}
exp1	8.1	1
exp2	2.7	1
mean	5.4	1

rpl11	Elav>TBPH	Elav>TBPH-RBD^{mut}
exp1	1.4	1
exp2	1.5	1
mean	1.45	1

normalized values
